# Genome-wide association analysis for chronic venous disease identifies *EFEMP1* and *KCNH8* as susceptibility loci

**DOI:** 10.1038/srep45652

**Published:** 2017-04-04

**Authors:** Eva Ellinghaus, David Ellinghaus, Petra Krusche, Aljoscha Greiner, Claudia Schreiber, Susanna Nikolaus, Christian Gieger, Konstantin Strauch, Wolfgang Lieb, Philip Rosenstiel, Norbert Frings, Andreas Fiebig, Stefan Schreiber, Andre Franke

**Affiliations:** 1 Institute of Clinical Molecular Biology, Christian-Albrechts-University of Kiel, 24105 Kiel, Germany; 2 Capio Mosel-Eifel-Clinic, 56864 Bad Bertrich, Germany; 3 Department of General Medicine, University Hospital Schleswig-Holstein, Campus Kiel, 24105 Kiel, Germany; 4 Institute of Genetic Epidemiology, Helmholtz Zentrum München, German Research Center for Environmental Health, 85764 Neuherberg, Germany; 5 Institute of Medical Informatics, Biometry and Epidemiology, Chair of Genetic Epidemiology, Ludwig-Maximilians-Universität, 80539 Munich, Germany; 6 PopGen Biobank, Institute of Epidemiology, University Hospital Schleswig-Holstein, 24105 Kiel, Germany

## Abstract

Chronic venous disease (CVD) is a multifactorial condition representing one of the most common disorders among populations of Western countries. The heritability of about 17% suggests genetic risk factors in CVD etiology. However, so far the genetic causes are unknown. We undertook the hitherto first genome-wide association study (GWAS) for CVD, analyzing more than 1.93 M SNPs in 4,942 German individuals, followed by replication in two independent German data sets. The combined analysis of discovery and replication stages (2,269 cases and 7,765 controls) yielded robust associations within the two genes *EFEMP1* and *KCNH8* (rs17278665, rs727139 with *P* < 5 × 10^−8^), and suggestive association within gene *SKAP2* (rs2030136 with *P* < 5 × 10^−7^). Association signals of rs17278665 and rs727139 reside in regions of low linkage disequilibrium containing no other genes. Data from the ENCODE and Roadmap Epigenomics projects show that tissue specific marks overlap with the variants. SNPs rs17278665 and rs2030136 are known eQTLs. Our study demonstrates that GWAS are a valuable tool to study the genetic component of CVD. With our approach, we identified two novel genome-wide significant susceptibility loci for this common disease. Particularly, the extracellular matrix glycoprotein EFEMP1 is promising for future functional studies due to its antagonistic role in vessel development and angiogenesis.

CVD is a complex venous pathology and defined as “morphological and functional abnormalities of the venous system of long duration manifested either by symptoms and/or signs indicating the need for investigation and/or care”^1^. Prevalence of CVD in Germany and the United States was estimated at 31%^2^ and 29%^2^, respectively. The term CVD encompasses patients with primary varicose veins (PVV) and chronic venous insufficiency (CVI). According to the CEAP guidelines (Comprehensive Classification System for Chronic Venous Disorders), absence of visible or palpable signs of venous disease is classified as C0 whereas the presence of telangiectasia or reticular veins leads to C1 classification. Varicose veins are distinguished from reticular veins by a diameter of at least 3 mm so that PVV patients who have dilated subcutaneous veins (≥3 mm Ø) but no signs of edema or skin changes are classified as C2. Patients with CVI can be classified as either C3 (edema), C4 (skin changes without ulceration), C5 (healed venous ulcer) or C6 (active venous ulcer)^3^,^4^. Exemplary photos for C1 to C6 are shown in Fig. 1. CVD has a major socioeconomic impact. The financial burden of CVD treatment on the health-care system is immense with an estimated cost of US$3 billion per year in the USA and up to 2% of the total health-care budget of all Western countries^5^,^6^. Despite the frequency of CVD and its considerably high impact on health-care budgets and on patients’ quality of life the underlying etiology and pathophysiology are still poorly understood. Many factors may increase risk for CVD, including age, female gender, pregnancy, hormonal changes, obesity and standing occupation and predispose individuals to the dilatation, elongation and tortuosity of the saphenous vein and its tributaries^7^. In addition, a genetic component has been proposed for many years, supported by reports on familial clustering and twin studies^8^,^9^,^10^. The narrow-sense heritability (proportion of the phenotypic variance that is explained by additive genetic variance) of PVV and CVD has recently been estimated to equal 18.5% and 17.3%, respectively, in a large sample of affected nuclear families from Germany^4^. These heritability estimates suggest the existence of susceptibility genes, necessitating large systematic association studies. So far, no disease association has been confirmed. To further our understanding of the genetic etiology of CVD, we undertook the hitherto first genome-wide association study (GWAS) for this multifactorial condition comprising 10,034 individuals in total.

## Results

To identify common variants that confer susceptibility to CVD, we performed genome-wide SNP genotyping of 323 unrelated German C2, C3 and C4 CVD cases and 4,619 healthy German control individuals employing Affymetrix SNP arrays (panel A, Supplementary Table 1). Principal component analysis supported European ancestry for all cases and controls (Methods, Supplementary Figure 1). To facilitate the analysis of the four data sets genotyped on different SNP chips and to increase the genomic coverage of our study by predicting untyped markers, SNP genotype imputation was performed. For imputation and subsequent statistical analysis, we used data sets that passed stringent quality control filters (Methods). In total, 1,934,349 quality-controlled autosomal-imputed SNP markers were available for association analysis (Supplementary Figure 2). Assuming a frequency of the disease-associated allele of at least 20% in controls, our screening panel had 79% power to detect a variant with an odds ratio of 1.3 or higher at the 0.001 significance level, which was the value used to define regions for attempting replication in a larger sample set. For stage one replication, we selected 70 SNPs based on their *P*-value rankings in the discovery stage and support by other SNPs in linkage disequilibrium (Methods, Supplementary Table 2). The replication sample of stage one consisted of 1,258 independent CVD cases and 1,925 controls from Germany (panel B, Supplementary Table 1). For stage two replication analysis, we selected three SNPs that replicated at a significance level of *P* < 0.05/70 = 7.14 × 10^−4^ (based on Bonferroni correction) in replication stage one for TaqMan^®^ genotyping (Supplementary Table 3) in an additional German panel comprising 688 CVD cases and 1,221 control individuals (panel C, Supplementary Table 1). We used a significance level of 7.14 × 10^−4^ for the statistical association analysis in the replication panels (panel B and C; Supplementary Table 1) based on Bonferroni correction (Methods). All three SNPs were significant in a meta-analysis of the combined replication data (panel B and C) after adjusting for multiple testing (*P*_Repl1&2_ = 2.59 × 10^−5^ for rs17278665; *P*_Repl1&2_ = 3.39 × 10^−8^ for rs727139; *P*_Repl1&2_ = 6.84 × 10^−5^ for rs2030136; Table 1). In a combined meta-analysis of the discovery and replication samples (2,269 CVD cases and 7,765 controls) SNPs rs17278665 (*P*_combined_ = 1.74 × 10^−8^) and rs727139 (*P*_combined_ = 5.42 × 10^−11^) reached genome-wide significance (*P* < 5 × 10^−8^ Table 1). To assess the robustness of our results, the association analyses were also carried out with adjustments made for sex, age at ascertainment and body mass index (BMI). No notably different results were obtained in the sex-, age- and BMI-adjusted analyses (Supplementary Table 4).

When checking for potential coding SNPs being highly correlated (r^2^ > 0.8 in 1000 Genomes European samples) with the three lead SNPs (Methods), we identified only one further intronic SNP at the *EFEMP1* and *KCNH8* loci, respectively. At the *SKAP2* locus 163 intronic variants, one 5’ UTR variant, five variants upstream and one variant downstream of *SKAP2* correlate with the lead SNP. However, no coding variants in high LD were detected. 31 of these non-coding LD SNPs, all located at the *SKAP2* locus on chromosome 7, were predicted to be deleterious by the fathmm^11^ algorithm (with values above 0.5), and 23 variants with a more stringent cutoff value above 0.8, respectively (Supplementary Table 5). The CADD^12^ method assigned scaled C scores of 10 or greater for 27 variants at the *SKAP2* locus, indicating that these variants are among the highest 10% of all scores, whereas five variants were assigned a scaled C score above 15, a suggested cutoff value on the likelihood of deleteriousness to identify potentially pathogenic variants (Supplementary Table 5). Of these five variants, four variants (rs10266759, rs3801828, rs3801808, rs3801814) had a fathmm score greater 0.8, strongly confirming their potential deleterious effect (Supplementary Table 5).

**Figure 2:**
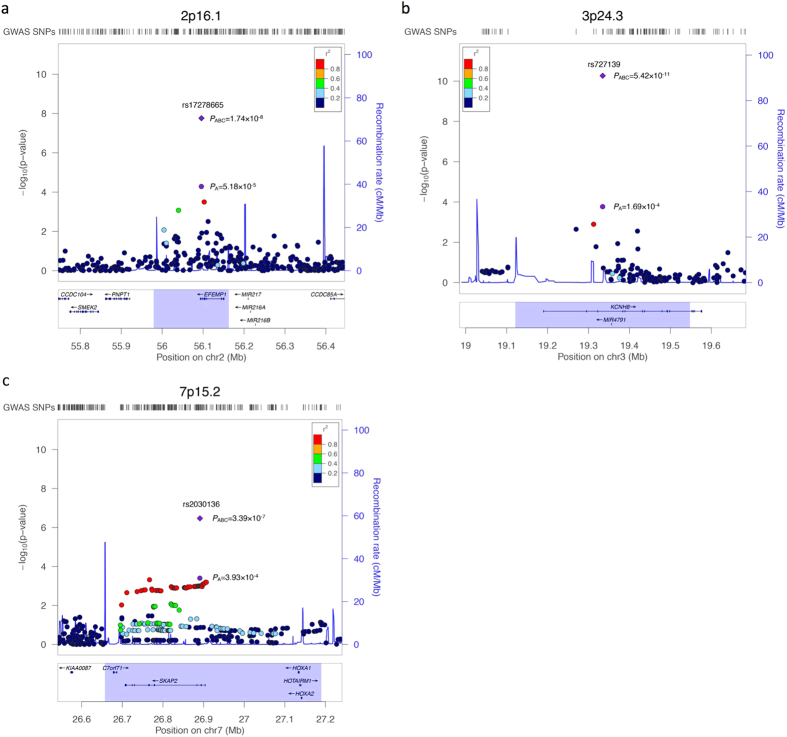
Regional association plots of loci with strongest evidence of association with CVD in combined sample. Shown are the negative log_10_ (genomic control adjusted) *P-*values from the discovery analysis of 323 unrelated German CVD cases and 4,619 healthy control individuals (panel A, Supplementary Table 1) with regard to the physical location of markers. The intronic SNPs (**a**) rs17278665, (**b**) rs727139, and (**c**) rs2030136 were genotyped in the two replication panels and therefore the combined *P*-value of panels A, B and C (*P*_ABC_) is indicated for these three lead SNPs (purple diamond) in addition to the *P*-value of panel A (*P*_A_), whereas for the surrounding SNPs in each plot only the respective *P*-values of panel A are available. Loci 2p16.1 and 3p24.3 are genome-wide significant risk loci (*P* < 5 × 10^−8^) in the combined analysis of discovery and replication stages (2,269 CVD cases and 7,765 controls). **Purple circle:** index SNP with *P*-value of panel A; **Purple diamond**: index SNP with combined *P*-value of panel A, B and C; **filled circles:** analyzed SNPs with the fill color corresponding to the strength of linkage disequilibrium (r^2^) with the lead SNP (for color coding see legend in the upper right corner of each plot); **blue line:** recombination intensity (cM/Mb); **highlighted region:** association boundaries for each index SNP (see Methods and Table 1). Positions and gene annotations are according to NCBI’s build 37 (hg19).

The intronic SNP rs17278665 at 2p16.1 maps to the last intron of the gene *EFEMP1* (epidermal growth factor-containing, fibulin-like extracellular matrix protein 1; also known as *FBNL*), which encodes the extracellular matrix protein fibulin-3. The association signal resides in a region of low linkage disequilibrium containing no other genes (Fig. 2a), pointing out the possibility of a direct involvement of *EFEMP1* in disease development. The odds ratio for carriership of the minor risk allele G of rs17278665, as estimated from replication samples, was 1.26 (95% CI = 1.13–1.41). The variant is a known local eQTL for *EFEMP1 (P*_eQTL_ = 3.45 × 10^−7^ in lung tissue and *P*_eQTL_ = 2.25 × 10^−6^ in skin)^13^,^14^ (Methods, Supplementary Table 6) with the minor allele being associated with decreased expression^14^ (Supplementary Figure 3a). Although classified as benign, the Mutation Taster^15^ prediction tool reports the minor allele to increase a donor splice site and as a consequence, the potential loss of the protein region that mediates interaction with TIMP3, located partially downstream of the altered splice site (Supplementary Table 5). *EFEMP1* is highly expressed in endothelial cells like Telomerase-immortalized microvascular endothelial cells (RNA: 1866 FPKM, protein: 8931)^16^, human umbilical vein endothelial cells (HUVECs; 7680 RPKM), human dermal blood endothelial cells (5896 RPKM) and human brain microvascular endothelial cells (4650 RPKM)^17^ (Supplementary Table 7). Data from the ENCODE^17^ and Roadmap Epigenomics^18^ projects imply that in HUVECs and mesenchymal tissue the variant overlaps with an HMM-predicted enhancer that is classified as a genic enhancer (15-state core model) and transcribed 3’ enhancer (25-state model), respectively (Supplementary Table 8).

The other genome-wide significantly associated SNP rs727139 at 3p24.3 (OR_Repl1&2_ = 0.75, 95% CI = 0.68–0.83) is located within the third intron of the *KCNH8* gene without any other genes contained between the association boundaries (Fig. 2b). *KCNH8* encodes a potassium voltage-gated channel. The Mutation Taster^15^ tool reports the minor allele of the variant to cause the generation of a new donor splice site, and the potential loss of several protein domains downstream of the altered splice site (Supplementary Table 5). According to the ENCODE^17^ data, *KCNH8* is almost exclusively expressed in CD20^+^ B cells (13.1 RPKM) and embryonic stem cells (6.6 RPKM) (Supplementary Table 7). In the Roadmap Epigenomics^18^ data, there is a cluster of promoter and enhancer activity in embryonic stem cells and induced pluripotent stem cells (iPSCs) overlapping with the variant. The 15-state core model classified it as enhancer and flanking active transcription start site whereas the 25-state core model classified it as active enhancer. In addition, a DNase hypersensitivity data peak overlaps with rs727139 in iPSCs (Supplementary Table 8).

SNP rs2030136 is located within the third intron of the *SKAP2* gene (src kinase associated phosphoprotein 2; 7p15.2) and showed highly significant association in the combined analysis (*P*_combined_ = 3.39 × 10^−7^) (Fig. 2c). The gene product of *SKAP2* is potentially involved in leukocyte adhesion processes and couples the B-cell receptor in B-cells to integrin activation^19^. According to the 1000 Genomes Project EUR reference^20^, SNP rs2030136 is in perfect LD (r^2^ = 1.0) with SNPs rs7804356 and rs10486483 that were previously reported to be associated with type I diabetes^21^ and Crohn’s disease^22^, respectively. SNP rs2030136 is a known local eQTL of *SKAP2 (P*_eQTL_ = 4.99 × 10^−141^)^23^ and *HOXA5 (P*_eQTL_ = 4.69 × 10^−7^)^14^ (Supplementary Table 6), a gene that blocks angiogenesis, with the minor allele being associated with increased expression (Supplementary Figure 3b). In addition, this SNP is in high LD (r^2^ > 0.8) with 168 other known eQTL SNPs for *SKAP2* and *HOXA5* (Supplementary Table 6). Although *SKAP2* is expressed ubiquitously in human tissues^24^, it is expressed stronger in human renal cortical epithelial cells (31 RPKM), lymphoblastoid cells (18 RPKM) and human dermal blood endothelial cells (17 RPKM) according to the ENCODE data (Supplementary Table 7).

## Discussion

We performed to our knowledge the first genome-wide association study for CVD and identified three novel CVD susceptibility loci. The analysis of our combined sample of 2,296 CVD cases and 7,765 controls implicates genetic variants in the genes *EFEMP1, KCNH8* and *SKAP2* in CVD susceptibility for the first time. The gene product of *EFEMP1*, fibulin-3, is an extracellular matrix glycoprotein, and belongs to the six-member fibulin family. The protein family is phylogenetically conserved and shows a high conservation of orthologues among different species. A single missense mutation in the *EFEMP1* gene causes the autosomal dominant maculopathy Malattia Leventinese/Doyne honeycomb retinal dystrophy in all patients and families so far reported^25^. Efemp1−/− mice showed reduced fertility, premature aging, had a reduced life span and other phenotypes. Histologic analysis of these mice revealed a marked reduction of elastic fibers in fascia throughout the body^26^ whereas mice that lack fibulin-1 die at birth from defects associated with endothelial cell abnormalities^27^. Fibulin-3 is most homologous to fibulin-4 and -5, but also shows a strong homology to members of the fibrillin family^28^. Mutations in fibulin-5 have been identified in patients with cutis laxa^29^,^30^ and mutations in fibrillin I cause Marfan syndrome, a disorder of connective tissue with manifestations involving the skeletal, ocular and cardiovascular systems^31^. There is evidence that fibulin-3 and -5 antagonize vessel development *in vivo* and fibulin-3 and -5 both repress the expression of the matrix metalloproteinases MMP-2 and MMP-3 while they induce the expression of the tissue inhibitors of metalloproteinases TIMP-1, TIMP-3 and TSP-1 in endothelial cells^32^. In varicose veins the expression of MMP1, -2, -3, -7, -9 and -13, and TIMP-1 and -3 is increased^33^,^34^,^35^. Both groups of enzymes (MMPs and TIMPs) regulate homoeostasis of the extracellular matrix with MMPs being involved in degradation of the extracellular matrix and TIMPs influencing vascular remodeling^34^,^36^. An imbalance between these proteins may lead to changes in the vessel wall and, eventually, to CVD. Because *EFEMP1* is expressed in disease-relevant tissue (Supplementary Table 7), we hypothesize that genetic variation in *EFEMP1* could influence the expression of certain MMPs and TIMPs which might result in defects in cellular and extracellular matrix components, causing an altered vein elasticity. However, fine-mapping studies are needed to identify the actual causal variant(s) and its/their functional consequence(s).

*KCNH8* is a member of the human Elk K^+^ channel gene family. Voltage-gated potassium channels represent the most complex class of voltage-gated ion channels from both functional and structural standpoints. Their diverse known functions include regulating neurotransmitter release, heart rate, insulin secretion, neuronal excitability, epithelial electrolyte transport and smooth muscle contraction. Interestingly, several studies have demonstrated the involvement of ion channels in venous dilatation and varicose vein formation^37^. For example, Raffetto *et al*. described MMP-2 induced venous dilatation via hyperpolarization and activation of K^+^ channels. Among several possible mechanisms by which MMP-2 could activate K^+^ channels, they suggested that as a result of collagen degradation by the protease activity of MMP-2, soluble Arg-Gly-Asp (RGD) tripeptide can bind to smooth muscle α_v_β_3_ integrin receptors and lead to activation of K^+^ channels, venous hyperpolarization and relaxation^38^. This is further supported by an observed partial inhibition of vasodilatation with inhibitors of voltage-gated, ATP-sensitive and inward rectifying K^+^ channels^39^. Whether the herein identified marker rs727139 is tagging causative variant(s) at the *KCNH8* locus and what role the protein itself might play in disease etiology needs to be elucidated in the future.

The gene product of *SKAP2* is an adaptor protein that is thought to be involved in the Src signaling pathway and to regulate proper activation of the immune system. Among its related super-pathways is “cell junction organization”. Variants at the *SKAP2* gene locus have previously been implicated in type 1 diabetes^40^ and in Crohn’s disease^22^. *SKAP2* is expressed broadly in lymphoid and myeloid cells and plays an adhesive/migratory role in B cells and dendritic cells. It interacts with the immune inhibitory receptor protein SIRPα (Signal Regulatory Protein-α) and drives the transduction of integrin-evoked signals which cause cytoskeletal reorganization required for macrophage migration^41^. Skap2- and Sirpα-deficient mice are resistant to experimental autoimmune encephalomyelitis and collagen-induced arthritis, two models for the human diseases multiple sclerosis and rheumatoid arthritis that have integrin-dependent components. These findings imply an important role for *SKAP2* in inflammation, particularly in integrin-dependent and macrophage-centric processes like atherosclerosis and inflammatory bowel disease^41^. A key aspect of CVD is inflammation, particularly caused by macrophages and monocytes, resulting from persistently increased venous pressure which is sensed by the endothelial cells and followed by recruitment of leukocytes, endothelial attachment, and finally, initiation of an inflammatory cascade^42^. Accordingly, *SKAP2* is an interesting candidate gene for functional studies in CVD development. The minor allele of SNP rs2030136 is strongly associated with increased expression of *SKAP2* and *HOXA5*. Furthermore, there are 168 other known eQTL SNPs for *SKAP2* and *HOXA5* in high LD. *HOXA5* is abundantly expressed in quiescent blood vessels, whereas its expression is diminished or absent in active angiogenic endothelial cells^43^. The *HOXA5* gene blocks angiogenesis by up-regulating expression of anti-angiogenic genes like Thrombospondin-2 and down-regulating many pro-angiogenic genes such as vascular endothelial growth factor receptor 2 (*VEGFR2)* and hypoxia inducible factor-1 alpha (*HIF1α)*^43^. VEGFR-2 mediates most of the known cellular responses to VEGF, which plays a central role in maintaining vascular integrity and reactivity. The proangiogenic cytokine VEGF and its upstream transcription factor HIF-1α are important for new vessel formation such as vasa vasora^44^,^45^.

Identification of genetic susceptibility factors in the etiology of chronic venous disease is fundamental for future translational and clinical research in order to understand disease mechanisms and to be able to translate knowledge of involved genes into better prevention and/or treatment of patients. The three risk variants newly identified here collectively account for 2.0% of the total variance in disease liability (heritability) for CVD, indicating that more powerful GWAS studies including larger sample sets are required not only to confirm the herein reported loci but to detect potential additional causal variants. Fine-mapping and resequencing efforts together with extensive functional studies may help to specify the contribution of the respective genes to overall disease susceptibility and to study the mechanisms that are altered through the risk variants.

## Methods

### Study population

To narrow down the wide spectrum of clinical severity of CVD, we included only patients with primary varicose veins with and without signs of edema and/or skin changes (C2-C4) but without healed or active venous ulcer (C5/C6). The prevalence of the different CVD grades (C2-C4) in the overall study population is presented in Supplementary Table 9. Personal characteristics of the CVD patients and healthy control individuals can be found in Supplementary Tables 10 and 11, respectively. In all patients, the primary varicose veins affected the superficial great saphenous vein (vena saphena magna), frequently accompanied by additional varicosities of the short saphenous vein (vena saphena parva). Patients with any signs of acute deep vein thrombosis were excluded. The samples were organized in panels that corresponded to the successive steps of the present study. All individual panels (A-C in Supplementary Table 1) were independent from each other. All patients were from Germany, recruited at the Capio Mosel-Eifel-Clinic (Bad Bertrich, Germany). Ascertainment criteria for CVD are based on the CEAP classification system, which was developed in 1994 and subsequently revised^3^. Its key elements are clinical severity, etiology or cause, anatomy and pathophysiology.

4,374 German healthy control individuals of panel A-C were obtained from the PopGen biobank^46^. 3,391 German healthy controls of panel A were selected from the KORA S3 and S4 survey, an independent population-based sample from the general population living in the region of Augsburg in southern Germany^47^.

Genomic DNA was prepared from blood samples using the Invisorb Blood Giga Kit (Invitec, Berlin, Germany). All DNA samples were quality-checked on agarose gels.

Written, informed consent was obtained from all study participants and the Ethics Committee of the Medical Faculty of the CAU Kiel approved all protocols. All methods carried out were in accordance with the approved study protocols.

### SNP genotyping for discovery sample and quality control

Genotyping of GWAS cases and PopGen controls - which was part of the German GWAS initiative funded by the National Genome Research Network (NGFN) - was performed as a service project by Affymetrix. All experimental steps were carried out according to standard protocols. SNPs that had > 5% missing data in cases or controls, a minor allele frequency <1% or deviated from Hardy-Weinberg equilibrium (exact *P* < 10^−4^ in controls) were excluded using the PLINK software version 1.07^48^. We excluded individuals with more than 5% missing genotypes (i.e. call rate < 95%), individuals from each pair of unexpected duplicates or relatives, as well as outlier individuals with average marker heterozygosities of ± 5 s.d. away from the sample mean. Pair-wise percentage identity-by-state (IBS) values were computed for all individuals and all QCed markers using PLINK. ‘Extreme outliers’ were detected by comparing the distribution of the pair-wise IBS values for each individual with the combined IBS distribution of the entire study population. Two types of “outliers” were detected: 1) Individuals less related to the entire study population than expected were defined as those for whom > 60% of the IBS values were smaller than the median minus three times the interquartile range (3 × IQR) of the population distribution. In this case, the individual was removed from the population. 2) Individuals with a close cognate (relative) in the population were defined as those who had at least one observed IBS value above the median plus 3 × IQR. In this case, the member of the cognate pair with the lower call rate was removed from the population. ‘Extreme outliers’ were calculated using R 2.15.2^49^.

These quality control measures left genotype data of a set of 302,457 autosomal overlapping SNPs in 323 CVD samples and 4,619 control samples for inclusion in the discovery phase (panel A, Supplementary Table 1). These samples were tested for population stratification using the principal components stratification method, as implemented in EIGENSTRAT^50^. Principal component analysis revealed no population outliers (Supplementary Figure 1).

### Genome-wide genotype imputation and association analysis

As input for the imputation only genotyped SNPs that passed quality control were used. SNP genotype imputation was performed using the MACH version 1.10.16^51^ and 690 HapMap3 reference haplotypes from the CEU, TSI, MXL and GIH^52^ as well as 120 HapMap II CEU phased haplotypes cohorts to predict missing autosomal genotypes *in-silico*^53^. Including samples from diverse populations such as Mexican (MXL) and Indian (GIH) can provide significant improvement in imputation for low-frequency variants (MAF < 5%)^54^. For downstream analyses, we included only SNPs that were imputed with high confidence (estimated r^2^ between imputed and true genotypes >0.8) and had a MAF ≥1%. We further excluded markers deviating from HWE in controls after imputation (*P* < 10^−6^). In total, 1,934,349 quality-controlled autosomal genotyped or imputed SNP markers were available for association analysis (Supplementary Figure 2). To take imputation uncertainty into account, phenotypic association was tested for allele dosage data using MACH2DAT’s logistic regression framework for dosage data^51^. The allelic dosage is the weighted sum of the genotype class probabilities. To control potentially confounding effects due to population stratification, we adjusted for the top five eigenvectors (indicating statistical significance by Tracy-Widom statistics) from EIGENSTRAT in the regression analysis. In addition, genomic control correction was applied to results by correction of χ^2^ test statistics using inflation factor λ = 1.156 and multiplying SNP standard errors by the square root of the inflation factor in order to minimize artificial differences in allele frequencies due to different genotyping platforms (see quantile-quantile plot in Supplementary Figure 4).

### Replication genotyping

Since only a few variants showed a substantial deviation from the expected distribution of genome-wide *P*-values in the tail of the distribution, we genotyped in stage one the 70 most strongly associated SNPs with *P* < 10^−3^ from each associated locus by means of PLINK’s clumping procedure (using settings *P*_1_ < 0.001, *P*_2_ < 0.05, r^2^ ≥ 0.8, kb = 250) in an independent German panel of 1,258 CVD cases and 1,925 control individuals (panel B; Supplementary Table 1), using the Sequenom^®^ iPlex platform and TaqMan^®^ technology from Life Technologies. Adjustment for multiple testing was performed by Bonferroni correction for 70 SNPs (association results of 70 SNPs; Supplementary Table 2). In stage two, we selected 3 SNPs that replicated at *P* < 0.05/70 = 7.14 × 10^−4^ in stage one for TaqMan^®^ genotyping in an additional German panel comprising 688 CVD cases and 1,221 control individuals (panel C; Supplementary Table 1, association results of 3 SNPs; Supplementary Table 3). Individual samples with >5% missing data were excluded from the downstream analyses. SNPs that had >3% missing data or deviated from Hardy-Weinberg equilibrium (exact *P* < 10^−4^ in controls) were excluded. *P*-values for allele-based tests were calculated using PLINK. The fixed-effects meta-analysis function of PLINK was then used to obtain *P*-values for the combined replication data set (panel B-C) and the combined discovery-replication data set (panel A-C). We used the commonly accepted threshold of 5 × 10^−8^ for joint *P-*values to define genome-wide significance.

### Annotation of association boundaries & regional association plots

Linkage disequilibrium regions (association boundaries) around lead SNPs from Table 1 were defined by extending in both directions a distance of 0.1 centimorgans (cM). For each locus, candidate genes within regions are listed in the column ‘Key gene’ and more details are listed in the legend of Table 1. Regional association plots of Fig. 2 were generated using Locuszoom^55^.

### Annotation of associations to other phenotypes

Overlaps with other phenotypes were annotated with the NHGRI GWAS-catalog^56^ (http://www.genome.gov/gwastudies/, date of access Dec-1-2015). All known associations with *P* < 5 × 10^−8^ with any disease or primary phenotype were included. For each susceptibility locus with association boundaries defined in Table 1, we annotated all phenotypes that had at least one associated SNP within the region. We also checked whether the hit SNP in the NHGRI GWAS catalogue was the same as, or in high LD with (r^2^ > 0.8) the CVD lead SNP.

### Functional annotation of associated variants

All SNPs in high LD (r^2^ > 0.8) with the three lead variants at CVD associated loci were identified using the SNP Annotation and Proxy Search^57^ (SNAP) online tool (version 2.2; https://www.broadinstitute.org/mpg/snap/index.php). The three non-coding lead SNPs and all variants in high LD (171 non-coding SNPs) were annotated using the following online tools that aim to predict the functional impact of non-coding sequence variants by obtaining information about sequence conservation (conservation metrics like GERP, phastCons and phyloP), chromatin state and protein binding annotation from the Roadmap Epigenomics^18^ and ENCODE^17^ projects, the effect of SNPs on regulatory motifs, on expression from eQTL studies and on splice sites (Supplementary Tables 5, 6, 8): Combined Annotation-Dependent Depletion (CADD)^12^ v1.3 (GRCh37/hg19; http://cadd.gs.washington.edu/home), fathmm^11^ v2.3 (http://fathmm.biocompute.org.uk/index.html), MutationTaster^15^ (NCBI build 37, Ensemble 69; http://www.mutationtaster.org) and HaploReg^58^ v4.1 (http://www.broadinstitute.org/mammals/haploreg/haploreg.php). The tool CADD uses the Ensembl Variant Effect Predictor (VEP^59^), data from the ENCODE^17^ project and information from UCSC genome browser tracks for annotation. The output of MutationTaster includes ‘regulatory features’ like histone modification sites, open chromatin or transcription factor binding sites from the Ensembl Regulation database (http://www.ensembl.org/info/genome/funcgen/index.html) and ‘splice sites’ to show possible changes in splice sites using the locally installed third party splice site prediction program NNSplice^60^. For the ‘splice site’ results, MutationTaster determines the position of the splice site change (increased/decreased or gained/lost splice site) relative to intron/exon borders. Only a loss/decrease of a splice site that occurs at an intron/exon (or reverse) border is shown, whereas such a change distant from any intron/exon border is ignored. For a significant finding the confidence score for the gain of a new splice site has to be greater than 0.3 and for an increase in an existing splice site greater than 10%. The online tool HaploReg annotates variants by their effect on regulatory motifs by using a library of position weight matrices collected from TRANSFAC, JASPAR, protein-binding microarray and ENCODE ChIP-seq experiments^61^ and extracts functional annotations from the ENCODE^17^ and Roadmap Epigenomics^18^ projects, including a chromatin 15-state and 25-state core model learned on five and 12 core chromatin marks, respectively. The chromatin state learning was performed to capture significant combinatorial interactions between different chromatin marks in their spatial context with a tool based on a multivariate Hidden Markov Model (HMM)^18^

### Expression quantitative trait loci (eQTL)

eQTL data was obtained from the genotype-tissue expression (GTEx) project^14^ (http://www.gtexportal.org/), the GEUVADIS^62^ data browser (http://www.ebi.ac.uk/Tools/geuvadis-das/) and 11 other studies using HaploReg^58^ v4.1 (see Supplementary Table 6).

### Gene expression

Information about *EFEMP1, KCNH8* and *SKAP2* gene expression (Supplementary Table 7) was obtained from the publicly available database *THE HUMAN PROTEIN ATLAS*^16^ (http://www.proteinatlas.org/) and the ENCODE^17^ expression data (http://promoter.bx.psu.edu/ENCODE/search_human.php). *THE HUMAN PROTEIN ATLAS*^16^ provides quantitative data on gene expression levels (RNA expression in fragments per kilobase of exon model per million mapped reads (FPKM) and protein expression as antibody staining intensity with values from 0 to 9000) for 46 different human cell lines and 44 human tissues. The ENCODE^17^ project provides gene expression data of 58 human cell types consisting of 108 datasets (RNA expression in reads per kilobase per million mapped reads (RPKM)).

### Variance explained and heritability of single SNPs

The proportion of variance explained by each association signal per population was calculated using a liability threshold model^63^ assuming a disease prevalence of 0.313 for CVD and log-additive disease risk from replication stage1&2 samples (panel B-C).

## Additional Information

**How to cite this article:** Ellinghaus, E. *et al*. Genome-wide association analysis for chronic venous disease identifies *EFEMP1* and *KCNH8* as susceptibility loci. *Sci. Rep.* **7**, 45652; doi: 10.1038/srep45652 (2017).

**Publisher's note:** Springer Nature remains neutral with regard to jurisdictional claims in published maps and institutional affiliations.

## Supplementary Material

Supplementary Information

## Figures and Tables

**Figure 1:**
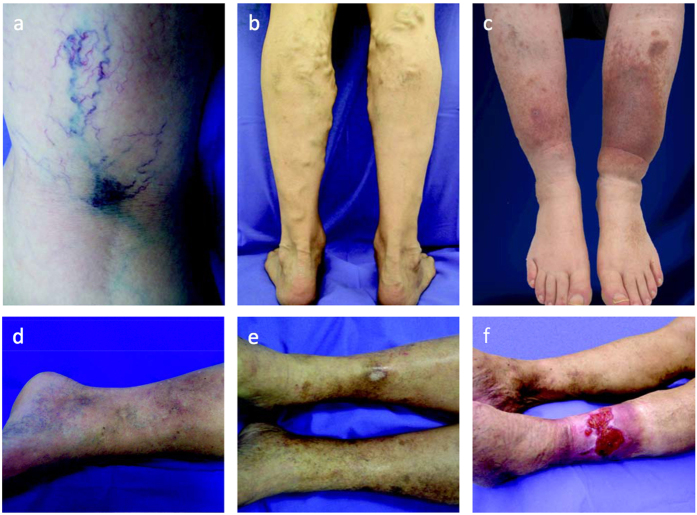
Exemplary photographs for a spectrum of clinical manifestations of chronic venous disease ranging from C1 to C6. (**a**) Spider veins, reticular veins; C1. (**b**) Varicose veins affecting the short saphenous vein; C2. (**c)** Edema due to varicose veins; C3. (**d**) Changes in skin and subcutaneous tissue, dermatosclerosis; C4. **(e)** Healed venous ulcer and Atrophie blanche; C5. **(f)** Active venous ulcer; C6. Photographs by courtesy of Dr. P. Krusche, Capio Mosel-Eifel-Clinic, Bad Bertrich.

**Table 1:** Loci with strongest evidence of association with CVD in combined sample.

Chr	Association boundaries (kb)	dbSNP id^a^	A1	A2	AF_ca_	AF_co_	Key gene (N additional in locus)	Discover GWAS (323/4,619)		Replication stage 1&2(1,946/3,146)		Discovery&Replication (2,269/7,765)		Associations (P < 5 × 10^−8^) to other traits
								*P*	OR (95% CI)	*P*	OR (95% CI)	*P*	OR (95% CI)	
2p16.1	55979–56163	**rs17278665**	G	C	0.17	0.15	***EFEMP1*** (0)	5.18 × 10^−5^	1.59 (1.28–1.98)	2.59 × 10^−5^	1.26 (1.13–1.41)	1.74 × 10^−8^	1.32 (1.19–1.45)	H, O, FVC
3p24.3	19121–19548	**rs727139**	G	A	0.16	0.21	***KCNH8*** (0)	1.69 × 10^−4^	0.63 (0.50–0.80)	3.39 × 10^−8^	0.75 (0.68–0.83)	5.42 × 10^−11^	0.73 (0.66–0.80)	—
7p15.2	26657–27190	rs2030136	C	T	0.26	0.24	*SKAP2* (9)	3.93 × 10^−4^	1.48 (1.20–1.83)	6.84 × 10^−5^	1.21 (1.10–1.33)	3.39 × 10^−7^	1.25 (1.15–1.36)	T1D*, CD*

We used a significance level of 0.05/70 = 7.14 × 10^−4^ for the statistical association analysis in replication panels (panel B and C; Supplementary Table 1) based on Bonferroni correction. All three SNPs replicated. Loci 2p16.1 and 3p24.3 are genome-wide significant risk loci (*P* < 5 × 10^−8^) for CVD.

**Chr**: chromosome; **Association boundaries (kb)**: association boundaries for each index SNP in kb (see Methods) with genomic positions retrieved from NCBI’s dbSNP build v138 (genome build hg19); **dbSNP id:** rs ID of index SNP; **A1:** minor allele; **A2:** major allele; **AF**_**ca**_**/AF**_**co**_: allele frequency of A1 estimated from replication stage1&2 (panel B-C) in cases and controls, respectively; **Key gene:** candidate gene in the region; for 7p15.2, 10 genes are covered by association boundaries: *C7orf71, HOTAIRM1, HOXA1, HOXA2, HOXA3, HOXA4, HOXA5, HOXA6, HOXA*-*AS3* and *SKAP2.* **P** and **OR (95% CI):** *P*-value and corresponding odds ratio and 95% confidence interval with respect to minor allele. For each panel, numbers of CVD cases/controls are displayed in parentheses. **Associations (*****P***** **<** 5 × 10**^**−8**^**) to other traits:** Overlaps with other disease phenotypes (listed if anywhere within association boundaries, * = known genome-wide significant risk SNP in perfect LD (*r*^2^ = |*D*′| = 1) with CVD hit SNP, see also Methods): CD = Crohn’s disease (rs10486483), FVC = forced vital capacity (rs1430193), H = Height (rs1367226, rs3791679, rs3791675), O = optic disc morphology (rs1346786), T1D = Type 1 diabetes (rs7804356). ^a^SNPs attaining genome-wide significance and their notable nearby genes are indicated in bold.
