# Supplementary material for: Genome-wide association analysis for chronic venous disease identifies EFEMP1 and KCNH8 as susceptibility loci
Source: Sci Rep. 2017 Apr 4;7:45652. doi: 10.1038/srep45652 (PMC5379489; doi:10.1038/srep45652)
Supplement: Supplementary Information [file srep45652-s1.pdf]

SUPPLEMENTARY INFORMATION

for

Genome-wide association analysis for chronic venous disease identifies *EFEMP1* and *KCNH8* as susceptibility loci

Eva Ellinghaus, PhD[1,¶]; David Ellinghaus, PhD[1,¶,*]; Petra Krusche, MD [2]; Aljoscha Greiner, MD[2]; Claudia Schreiber, MD[3]; Susanna Nikolaus, MD[3]; Christian Gieger, PhD[4]; Konstantin Strauch, PhD[4,5]; Wolfgang Lieb, MD[6]; Philip Rosenstiel, MD[1]; Norbert Frings, MD[2]; Andreas Fiebig, PhD[1]; Stefan Schreiber, MD[1,3]; Andre Franke, PhD[1]

1 Institute of Clinical Molecular Biology, Christian-Albrechts-University of Kiel, 24105 Kiel, Germany
2 Capio Mosel-Eifel-Clinic, 56864 Bad Bertrich, Germany
3 Department of General Medicine, University Hospital Schleswig-Holstein, Campus Kiel, 24105 Kiel, Germany
4 Institute of Genetic Epidemiology, Helmholtz Zentrum München, German Research Center for Environmental Health, 85764 Neuherberg, Germany
5 Institute of Medical Informatics, Biometry and Epidemiology, Chair of Genetic Epidemiology, Ludwig-Maximilians-Universität, 80539 Munich, Germany
6 PopGen Biobank, Institute of Epidemiology, University Hospital Schleswig-Holstein, 24105 Kiel, Germany

¶These authors contributed equally to this work
*To whom correspondence should be addressed:

Dr. David Ellinghaus
Institute of Clinical Molecular Biology
Christian-Albrechts-University of Kiel
University Hospital Schleswig Holstein · Campus Kiel
Rosalind-Franklin-Str. 12 · 24105 Kiel, Germany
Delivery address: Am Botanischen Garten 11, 24118 Kiel, Germany
Phone: +49 431 500 15131
Fax: +49 431 500 15178
eMail: d.ellinghaus@ikmb.uni-kiel.de

Supplementary Tables

Supplementary Table 1. Case/control panels used in the analysis.

Panel A: German discovery panel (GWAS) after quality control. ‘Group’ refers to the scientific group that provided samples.

Country	Group	No. Cases (C2, C3, C4)	No. Controls	Platform
Germany	Capio Mosel-Eifel-Clinic	323	-	Affymetrix 5.0
	CAU Kiel & PopGen	-	1228	Affymetrix 6.0
	KORA S3 (Helmholtz Center Munich)	-	1773	Affymetrix 500k Set
	KORA S4 (Helmholtz Center Munich)	-	1618	Affymetrix 6.0
Total		323	4619	

Panel B: Stage one replication in German population after quality control.

Country	Group	No. Cases (C2, C3, C4)	No. Controls	Platform
Germany	Capio Mosel-Eifel-Clinic	1258	-	Sequenom & TaqMan
	CAU Kiel & PopGen	-	1925	

Panel C: Stage two replication in German population after quality control.

Country	Group	No. Cases (C2, C3, C4)	No. Controls	Platform
Germany	Capio Mosel-Eifel-Clinic	688	-	TaqMan
	CAU Kiel & PopGen	-	1221	

Supplementary Table 2. Results of replication stage one genotyping and association testing of 70 SNPs in an independent German panel of 1,258 CVD cases and 1,925 controls (panel B, Supplementary Table 1).

CHR	BP	SNP	P_{GWAS}	A1	A2	AF_{cases}	$AF_{controls}$	P	OR	95% CI
1	50,336,554	rs4285748	1.82×10^{-4}	T	C	0.05	0.04	0.46	1.10	0.86-1.39
1	76,441,522	rs17097780	2.37×10^{-7}	T	A	0.09	0.08	0.27	1.11	0.92-1.33
2	53,885,058	rs11682721	1.08×10^{-4}	G	A	0.4	0.42	0.17	0.93	0.84-1.03
2	56,095,994	rs17278665	5.18×10^{-5}	G	C	0.18	0.14	8.47×10^{-5}	1.32	1.15-1.51
2	106,373,169	rs13028719	9.23×10^{-4}	C	T	0.28	0.28	0.48	0.96	0.86-1.08
2	128,309,469	rs12464981	3.05×10^{-4}	G	C	0.41	0.40	0.27	1.06	0.96-1.17
2	142,027,067	rs3856359	3.62×10^{-4}	T	A	0.08	0.07	0.09	1.18	0.98-1.43
2	154,163,944	rs1347927	7.65×10^{-5}	G	A	0.39	0.35	3.29×10^{-3}	1.17	1.05-1.30
2	218,588,976	rs6436007	1.33×10^{-7}	A	G	0.10	0.10	0.42	1.07	0.91-1.27
2	223,658,198	rs11689692	1.04×10^{-4}	A	T	0.13	0.13	0.81	1.02	0.88-1.18
3	19,334,695	rs727139	1.69×10^{-4}	G	A	0.18	0.22	4.54×10^{-5}	0.77	0.67-0.87
3	65,638,580	rs1499496	8.85×10^{-5}	C	T	0.07	0.07	0.72	1.04	0.85-1.27
3	73,379,546	rs12637430	1.13×10^{-4}	C	T	0.23	0.21	0.10	1.11	0.98-1.25
4	30,949,606	rs4692493	8.89×10^{-4}	A	T	0.47	0.46	0.76	1.02	0.92-1.12
4	107,359,768	rs6819528	7.29×10^{-5}	T	G	0.47	0.50	0.05	0.90	0.82-1.00
4	110,499,958	rs13118855	1.18×10^{-4}	G	A	0.10	0.11	0.10	0.87	0.74-1.03
4	110,571,210	rs1029453	2.42×10^{-4}	A	C	0.10	0.12	0.07	0.86	0.73-1.01
5	26,963,586	rs2127426	1.03×10^{-7}	C	G	0.17	0.17	0.45	0.95	0.83-1.09
5	64,495,058	rs1895431	4.90×10^{-4}	T	C	0.32	0.33	0.38	0.95	0.86-1.06
5	91,049,451	rs331385	6.09×10^{-5}	G	A	0.45	0.42	0.01	1.15	1.03-1.27
5	115,800,559	rs10077720	4.94×10^{-5}	A	G	0.10	0.08	0.01	1.26	1.06-1.51
5	127,684,411	rs11241956	1.97×10^{-4}	G	A	0.31	0.34	4.64×10^{-3}	0.86	0.77-0.95
5	171,128,464	rs6555969	3.88×10^{-4}	T	C	0.33	0.33	0.60	1.03	0.92-1.15
6	2,623,930	rs9503233	5.24×10^{-4}	A	G	0.21	0.21	0.45	0.95	0.84-1.08
6	33,020,842	rs3128953	1.20×10^{-12}	A	G	0.24	0.22	0.07	1.12	0.99-1.27
6	55,347,790	rs9396099	8.13×10^{-4}	T	C	0.26	0.23	1.93×10^{-3}	1.20	1.07-1.35
7	24,005,443	rs17148598	3.91×10^{-7}	G	A	0.08	0.08	0.57	0.95	0.78-1.14
7	26,891,016	rs2030136	3.93×10^{-4}	C	T	0.26	0.22	6.98×10^{-5}	1.27	1.13-1.43
7	31,445,598	rs1860793	5.99×10^{-4}	C	T	0.34	0.36	0.04	0.89	0.80-0.99
7	47,445,812	rs834601	3.95×10^{-4}	C	T	0.49	0.50	0.32	0.95	0.86-1.05
7	52,104,548	rs10278047	1.76×10^{-4}	T	C	0.22	0.25	3.03×10^{-3}	0.83	0.74-0.94
7	77,686,838	rs6958027	3.43×10^{-4}	T	G	0.44	0.42	0.30	1.06	0.95-1.17
7	77,868,102	rs10953499	6.86×10^{-5}	A	G	0.17	0.19	0.07	0.89	0.78-1.01
7	83,134,459	rs11982066	4.28×10^{-4}	C	T	0.41	0.43	0.12	0.92	0.83-1.02
7	115,708,814	rs12532028	8.00×10^{-6}	A	C	0.10	0.09	0.58	1.05	0.88-1.24
7	153,638,248	rs6973850	1.59×10^{-7}	T	C	0.12	0.11	0.36	1.08	0.92-1.26
8	59,857,097	rs16924316	1.58×10^{-4}	C	T	0.12	0.13	0.20	0.90	0.77-1.05

8	73,409,704	rs12547029	1.25×10^{-4}	T	C	0.42	0.42	0.91	0.99	0.90-1.10
8	73,419,786	rs1346958	5.39×10^{-5}	A	C	0.38	0.38	0.93	1.01	0.91-1.11
8	114,888,083	rs12334329	3.06×10^{-7}	A	G	0.13	0.13	0.95	1.01	0.87-1.17
8	133,247,788	rs4736558	7.37×10^{-4}	T	A	0.24	0.24	0.98	1.00	0.89-1.12
9	8,117,601	rs4742453	7.97×10^{-4}	A	G	0.12	0.12	0.44	1.06	0.91-1.24
9	22,740,232	rs7872081	5.30×10^{-6}	A	G	0.06	0.05	0.18	1.16	0.94-1.44
9	28,299,438	rs7855717	1.30×10^{-4}	C	T	0.29	0.30	0.55	0.97	0.87-1.08
9	71,198,014	rs17479566	3.11×10^{-5}	T	C	0.20	0.23	3.71×10^{-3}	0.83	0.74-0.94
10	6,677,767	rs10795331	7.79×10^{-5}	T	C	0.46	0.47	0.53	0.97	0.88-1.07
10	95,116,029	rs787629	1.20×10^{-4}	A	C	0.27	0.27	0.78	1.02	0.91-1.14
10	128,526,537	rs10794128	4.66×10^{-5}	C	T	0.35	0.33	0.03	1.13	1.01-1.25
11	63,948,354	rs4980525	5.14×10^{-4}	C	T	0.46	0.47	0.55	0.97	0.88-1.07
11	104,140,745	rs7950244	4.33×10^{-5}	C	A	0.07	0.07	0.24	0.89	0.73-1.08
12	27,031,461	rs7306179	5.20×10^{-4}	A	T	0.38	0.39	0.23	0.94	0.85-1.04
12	101,637,505	rs10778104	1.56×10^{-4}	A	C	0.40	0.37	0.06	1.10	1.00-1.22
12	106,299,118	rs7957583	8.97×10^{-4}	A	T	0.38	0.40	0.16	0.93	0.84-1.03
12	114,713,898	rs7962292	9.98×10^{-4}	T	C	0.35	0.35	0.82	1.01	0.91-1.13
13	19,875,086	rs547308	1.40×10^{-4}	C	T	0.41	0.41	0.94	1.00	0.90-1.10
13	24,208,829	rs7987909	6.30×10^{-4}	G	A	0.11	0.11	0.85	0.98	0.84-1.16
15	30,137,080	rs1403253	1.03×10^{-4}	G	A	0.15	0.13	0.13	1.12	0.97-1.29
15	70,817,223	rs8033107	3.11×10^{-4}	T	C	0.23	0.21	0.21	1.08	0.96-1.22
17	5,391,690	rs2309399	9.81×10^{-4}	A	G	0.43	0.44	0.45	0.96	0.87-1.07
17	68,256,295	rs236562	3.02×10^{-5}	G	A	0.41	0.42	0.39	0.96	0.86-1.06
18	3,656,488	rs1791398	8.30×10^{-4}	G	A	0.21	0.21	0.43	0.95	0.84-1.08
18	5,620,114	rs12455403	6.81×10^{-6}	C	T	0.18	0.18	0.55	0.96	0.84-1.10
18	28,619,690	rs276931	9.36×10^{-4}	G	A	0.31	0.33	0.15	0.92	0.83-1.03
19	3,709,018	rs570896	6.17×10^{-4}	G	C	0.31	0.31	0.92	1.01	0.90-1.12
19	16,337,588	rs10469462	1.04×10^{-4}	C	T	0.17	0.20	0.02	0.86	0.75-0.98
19	16,343,847	rs12984337	6.79×10^{-5}	T	A	0.28	0.28	0.92	0.99	0.89-1.11
19	16,368,528	rs4808478	2.98×10^{-5}	A	C	0.26	0.28	0.08	0.90	0.81-1.01
20	2,100,348	rs6112914	9.70×10^{-4}	A	G	0.20	0.20	0.48	0.96	0.84-1.08
20	61,018,265	rs6061531	4.34×10^{-7}	C	T	0.14	0.14	0.59	1.04	0.90-1.20
21	39,284,835	rs2836050	2.18×10^{-5}	T	C	0.20	0.20	0.73	1.02	0.90-1.16

CHR: chromosome; **BP:** position (NCBI build 37/hg19 coordinates) of SNP marker; **SNP:** SNP rs-id (if available from dbSNP130); **P_{GWAS}**: *P*-value in the initial genome-wide phase; **A1:** minor allele; **A2:** major allele; **AF_{cases}**: allele frequency of minor allele A1 in cases; **$AF_{controls}$**: allele frequency of minor allele A1 in controls; **P:** *P*-value; **OR:** corresponding odds ratio and **95% CI:** 95% confidence interval with respect to minor allele.

Supplementary Table 3. Results of replication stage two genotyping and association testing of 3 SNPs in an independent German panel of 688 CVD cases and 1,221 controls (panel C, Supplementary Table 1).

CHR	BP	SNP	P_{GWAS}	A1	A2	AF_{cases}	$AF_{controls}$	P	OR	95% CI
2	56,095,994	rs17278665	5.18×10^{-5}	G	C	0.17	0.15	0.07	1.18	0.99-1.41
3	19,334,695	rs727139	1.69×10^{-4}	G	A	0.16	0.21	1.53×10^{-4}	0.72	0.60-0.85
7	26,891,016	rs2030136	3.93×10^{-4}	C	T	0.26	0.24	0.17	1.11	0.95-1.30

CHR: chromosome; **BP:** position (NCBI build 37/hg19 coordinates) of SNP marker; **SNP:** SNP rs-id (if available from dbSNP130); $\mathbf{P_{GWAS}}$: *P*-value in the initial genome-wide phase; **A1:** minor allele; **A2:** major allele; $\mathbf{AF_{cases}}$: allele frequency of minor allele A1 in cases; $\mathbf{AF_{controls}}$: allele frequency of minor allele A1 in controls; **P:** *P*-value; **OR:** corresponding odds ratio and **95% CI:** 95% confidence interval with respect to minor allele.

Supplementary Table 4. Results of association testing of 3 SNPs in panel B and C with adjustments made for sex, age at ascertainment and body mass index (BMI) if available. No notably different results were obtained in the sex-, age- and BMI-adjusted analyses.

					panel B					panel B & C				
					no adjustment			BMI* adjusted		no adjustment			sex & age adjusted	
CHR	BP	SNP	A1	A2	P	OR	95% CI	P	OR	P	OR	95% CI	P	OR
2	56,095,994	rs17278665	G	C	8.47×10^{-5}	1.32	1.15-1.51	7.81×10^{-5}	1.32	2.59×10^{-5}	1.26	1.13-1.41	4.54×10^{-6}	1.32
3	19,334,695	rs727139	G	A	4.54×10^{-5}	0.77	0.67-0.87	6.33×10^{-5}	0.77	3.39×10^{-8}	0.75	0.68-0.83	4.99×10^{-8}	0.74
7	26,891,016	rs2030136	C	T	6.98×10^{-5}	1.27	1.13-1.43	2.18×10^{-4}	1.25	6.84×10^{-5}	1.21	1.10-1.33	2.53×10^{-5}	1.24

CHR: chromosome; **BP:** position (NCBI build 37/hg19 coordinates) of SNP marker; **SNP:** SNP rs-id (if available from dbSNP130); **A1:** minor allele; **A2:** major allele; **P/OR:** *P*-value, corresponding odds ratio and **95% CI:** 95% confidence interval with respect to minor allele.

*BMI was available for the cases of panel B and C, but only for the controls of panel B.

Supplementary Table 5. Functional annotation of three newly identified disease associations and all variants in high LD (r^2>0.8) with these variants. All variants were annotated using CADD[1] v1·3 (GRCh37/hg19;), fathmm[2] v2·3, MutationTaster[3] (NCBI build 37, Ensemble 69) and HaploReg[4] v4·1 to determine position annotations, including the closest gene, the effect on SNPs on splice sites and functional consequences (**Methods**).

	CADD																											FATHMM		MutationTaster						HaploReg				
SNP	CHR	BP	Ref	Anc	Alt	Type	AnnoType	Consequence	GC	CpG	priPhCons	mamPhCons	verPhCons	priPhyloP	mamPhyloP	verPhyloP	GerpN	GerpS	GerpRS	GerpRSpval	GeneName	Intron	Grantham	PolyPhenVal	SIFTval	RawScore	PHRED	Non-Coding score	Non-Coding Groups	pred_index	probability	features	splice site effect	splice site gDNA position	splice site score	Sequence constraint by GERP	Sequence constraint by SiPhy	NHGRI/EBI GWAS hits	RefSeq genes	dbSNP func annot
rs17278665	2	56,095,994	C	C	G	SNV	Transcript	INTRONIC	0.38	0.03	0.041	0.000	0.000	0.645	-0.305	-0.313	2.99	-1.36	.	.	EFEMP1	10/10	.	.	.	0.18	4.42	0.1742	ABC	polymorphism_automatic	1	splicing affected; protein features affected	Donor increased	55276	wt: 0.25 / mu: 0.73	.	.	.	EFEMP1	intronic
rs11899888	2	56,102,744	A	A	G	SNV	RegulatoryFeature	REGULATORY	0.40	0.01	0.018	0.000	0.000	-0.332	-0.171	-0.316	4.76	-2.52	.	.	.	.	.	.	.	-0.21	1.03	0.17565	ALL	polymorphism_automatic	1	.	.	.	.	.	.	.	EFEMP1	intronic
rs13064845	3	19,312,697	G	G	A	SNV	Transcript	INTRONIC	0.34	0.00	0.012	0.000	0.000	-0.817	-2.761	-2.743	4	-8	.	.	KCNH8	2/15	.	.	.	-0.25	0.85	0.05848	AD	polymorphism_automatic	1	.	.	.	.	.	.	.	KCNH8	intronic
rs727139	3	19,334,695	A	A	G	SNV	Transcript	INTRONIC	0.46	0.01	0.002	0.002	0.003	-0.499	0.122	0.182	3.31	0.676	.	.	KCNH8	3/15	.	.	.	-0.14	1.48	0.16699	AB	polymorphism_automatic	1	splicing affected; protein features affected	Donor gained	144744	0.88	.	.	.	KCNH8	intronic
rs212838	7	26,698,194	T	C	C	SNV	Intergenic	INTERGENIC	0.31	0.01	0.501	0.843	0.131	0.587	0.478	0.073	6.08	0.843	7306.1	0	.	.	.	.	.	0.14	4.03	0.36135	ALL			.	.	.	.	yes	yes	.	8.5kb 3' of SKAP2	.
rs3757654	7	26,711,104	C	C	T	SNV	Transcript	INTRONIC	0.30	0.01	0.043	0.002	0.010	-0.201	0.077	0.035	4.29	0.523	.	.	SKAP2	11/12	.	.	.	0.01	2.68	0.15937	A	polymorphism_automatic	1	.	.	.	.	.	.	.	SKAP2	intronic
rs212848	7	26,722,155	G	T	T	SNV	Transcript	INTRONIC	0.42	0.03	0.001	0.000	0.000	0.530	0.019	0.066	4.11	0.167	.	.	SKAP2	11/12	.	.	.	-0.22	1.00	0.09511	ABC	polymorphism_automatic	1	splicing affected; protein features affected	Donor gained	312704	0.5	.	.	.	SKAP2	intronic
rs212850	7	26,724,279	T	G	G	SNV	RegulatoryFeature	REGULATORY	0.34	0.03	0.093	0.000	0.000	0.651	0.450	-0.008	6.17	0.31	2222.5	1.8613e-243	.	.	.	.	.	-0.30	0.68	0.16952	ADB	polymorphism_automatic	1	.	.	.	.	.	.	.	SKAP2	intronic
rs212856	7	26,729,205	T	C	C	SNV	RegulatoryFeature	REGULATORY	0.42	0.00	0.995	0.997	0.770	0.651	1.435	0.914	5.39	4.51	5881	0	.	.	.	.	.	0.88	9.97	0.87582	ABC	polymorphism_automatic	0.00982381	.	.	.	.	yes	yes	.	SKAP2	intronic
rs212857	7	26,730,200	C	A	A	SNV	Transcript	INTRONIC	0.36	0.01	0.001	0.001	0.001	0.307	0.313	0.332	0.245	0.245	.	.	SKAP2	9/12	.	.	.	-0.13	1.49	0.0263	ADB	polymorphism_automatic	1	.	.	.	.	.	.	.	SKAP2	intronic
rs4719877	7	26,736,220	A	G	G	SNV	Transcript	INTRONIC	0.30	0.00	0.002	0.000	0.000	-1.023	-0.646	-0.651	3.75	-2.77	.	.	SKAP2	9/12	.	.	.	-0.42	0.35	0.07557	A	polymorphism_automatic	1	splicing affected; protein features affected	Acc increased	298634	wt: 0.25 / mu: 0.35	.	.	.	SKAP2	intronic
rs6461962	7	26,746,304	A	T	T	SNV	Transcript	INTRONIC	0.30	0.00	0.019	0.932	0.997	-0.464	0.001	0.547	5.06	1.37	2536.7	2.51131e-83	SKAP2	9/12	.	.	.	-0.17	1.25	0.91529	AB	polymorphism_automatic	8.80665E-09	.	.	.	.	.	yes	.	SKAP2	intronic
rs6972378	7	26,753,074	C	T	T	SNV	Transcript	INTRONIC	0.30	0.00	0.003	0.001	0.001	0.447	0.232	0.257	2.14	0.851	.	.	SKAP2	9/12	.	.	.	-0.02	2.42	0.03575	A	polymorphism_automatic	1	splicing affected; protein features affected	Acc increased	281788	wt: 0.57 / mu: 0.82	.	.	.	SKAP2	intronic
rs6951065	7	26,756,162	G	A	A	SNV	Transcript	INTRONIC	0.26	0.00	0.015	0.000	0.000	0.374	-0.036	-0.016	2.32	-0.177	.	.	SKAP2	9/12	.	.	.	-0.01	2.46	0.06862	A	polymorphism_automatic	1	splicing affected; protein features affected	Acc gained	278707	0.33	.	.	.	SKAP2	intronic
rs6964817	7	26,756,424	G	C	C	SNV	Transcript	INTRONIC	0.36	0.01	0.017	0.000	0.000	0.455	-0.195	-0.157	1.97	-0.933	.	.	SKAP2	9/12	.	.	.	-0.41	0.36	0.06764	A	polymorphism_automatic	1	splicing affected; protein features affected	Donor gained	278434	0.85	.	.	.	SKAP2	intronic
rs2106905	7	26,766,851	T	C	C	SNV	Transcript	INTRONIC	0.38	0.00	0.016	0.006	0.551	-0.150	0.209	0.419	5.18	0.731	.	.	SKAP2	6/12	.	.	.	0.19	4.59	0.55736	A	polymorphism_automatic	0.877647	splicing affected; protein features affected	Acc increased	268001	wt: 0.47 / mu: 0.59	.	.	.	SKAP2	intronic
rs6461963	7	26,772,491	G	A	A	SNV	Transcript	INTRONIC	0.36	0.01	0.017	0.000	0.000	0.473	0.824	0.509	4.52	1.91	.	.	SKAP2	6/12	.	.	.	0.15	4.14	0.05875	A	polymorphism_automatic	1	.	.	.	.	.	.	.	SKAP2	intronic
rs4722630	7	26,773,364	G	T	T	SNV	Transcript	INTRONIC	0.23	0.01	0.452	0.182	0.337	0.478	1.548	1.618	2.86	2.86	.	.	SKAP2	6/12	.	.	.	0.89	9.98	0.12369	A	polymorphism_automatic	0.999981	splicing affected; protein features affected	Donor gained	261491	0.72	.	.	.	SKAP2	intronic
rs6971397	7	26,782,564	C	T	T	SNV	Transcript	INTRONIC	0.26	0.03	0.015	0.000	0.000	0.455	-0.371	-0.314	2.47	-1.7	.	.	SKAP2	4/12	.	.	.	-0.06	2.01	0.05496	A	polymorphism_automatic	1	splicing affected; protein features affected	Donor gained	252296	0.42	.	.	.	SKAP2	intronic
rs727450	7	26,787,642	G	A	A	SNV	Transcript	INTRONIC	0.31	0.03	0.018	0.001	0.001	0.478	1.531	1.606	2.82	2.82	.	.	SKAP2	4/12	.	.	.	0.15	4.13	0.04876	A	polymorphism_automatic	1	splicing affected; protein features affected	Acc increased	247228	wt: 0.42 / mu: 0.53	.	.	.	SKAP2	intronic
rs10281261	7	26,789,472	T	C	C	SNV	Transcript	INTRONIC	0.32	0.00	0.076	0.000	0.000	0.587	-0.314	-0.301	2.18	-1.11	.	.	SKAP2	4/12	.	.	.	-0.01	2.45	0.0667	A	polymorphism_automatic	1	splicing affected; protein features affected	Donor gained	245386	0.98	.	.	.	SKAP2	intronic
rs7457076	7	26,793,215	C	T	T	SNV	Transcript	INTRONIC	0.28	0.00	0.184	0.056	0.123	0.520	0.764	0.823	3.58	2.44	.	.	SKAP2	4/12	.	.	.	0.49	7.34	0.07073	A	polymorphism_automatic	0.999999	splicing affected; protein features affected	Donor increased	241636	wt: 0.73 / mu: 0.86	.	.	.	SKAP2	intronic
rs2189017	7	26,795,073	A	G	G	SNV	Transcript	INTRONIC	0.29	0.00	0.088	0.001	0.002	-0.366	-0.816	-0.810	3.49	-3.31	.	.	SKAP2	4/12	.	.	.	0.10	3.65	0.06186	A	polymorphism_automatic	1	splicing affected; protein features affected	Acc increased	239783	wt: 0.29 / mu: 0.35	.	.	.	SKAP2	intronic
rs2189019	7	26,798,581	G	A	A	SNV	Transcript	INTRONIC	0.30	0.04	0.067	0.002	0.000	0.525	0.316	0.009	4.85	0.997	.	.	SKAP2	4/12	.	.	.	0.28	5.53	0.09056	AB	polymorphism_automatic	1	.	.	.	.	.	.	.	SKAP2	intronic
rs73061894	7	26,802,085	T	T	C	SNV	Transcript	INTRONIC	0.34	0.03	0.007	0.000	0.000	0.452	-0.533	-0.456	2.94	-2.25	.	.	SKAP2	4/12	.	.	.	-0.02	2.39	0.0953	A	polymorphism_automatic	1	splicing affected; protein features affected	Donor increased	232768	wt: 0.26 / mu: 0.94	.	.	.	SKAP2	intronic
rs117217399	7	26,805,212	T	T	C	SNV	Transcript	INTRONIC	0.38	0.03	0.084	0.084	0.100	0.157	0.159	0.169	0.118	0.118	.	.	SKAP2	4/12	.	.	.	-0.01	2.52	0.08193	A			.	.	.	.	.	.	.	SKAP2	intronic
rs10486478	7	26,819,465	C	C	T	SNV	Transcript	INTRONIC	0.30	0.00	0.012	0.013	0.019	-0.995	0.374	0.382	3.45	1.49	.	.	SKAP2	4/12	.	.	.	-0.15	1.37	0.14807	A	polymorphism_automatic	1	splicing affected; protein features affected	Donor increased	215389	wt: 0.51 / mu: 0.62	.	.	.	SKAP2	intronic
rs3801850	7	26,820,577	T	T	C	SNV	Transcript	INTRONIC	0.30	0.01	0.001	0.000	0.000	-1.154	0.001	0.159	4.45	0.586	.	.	SKAP2	4/12	.	.	.	-0.31	0.65	0.16597	A	polymorphism_automatic	1	splicing affected; protein features affected	Donor increased	214276	wt: 0.43 / mu: 0.98	.	.	.	SKAP2	intronic
rs12536560	7	26,822,876	G	G	C	SNV	Transcript	INTRONIC	0.44	0.01	0.021	0.000	0.000	0.462	-0.425	-0.375	2.04	-1.44	.	.	SKAP2	4/12	.	.	.	-0.30	0.66	0.09685	A	polymorphism_automatic	1	splicing affected; protein features affected	Donor gained	211982	0.82	.	.	.	SKAP2	intronic
rs73065504	7	26,825,984	A	A	G	SNV	Transcript	INTRONIC	0.35	0.00	0.056	0.000	0.000	-0.359	-0.805	-0.700	2.75	-4.26	.	.	SKAP2	4/12	.	.	.	-0.23	0.93	0.08031	A	polymorphism_automatic	1	.	.	.	.	.	.	.	SKAP2	intronic
rs10267950	7	26,826,757	T	T	A	SNV	Transcript	INTRONIC	0.36	0.01	0.020	0.058	0.098	0.530	1.970	2.069	3.88	3.88	.	.	SKAP2	4/12	.	.	.	0.69	8.77	0.42381	AB	polymorphism_automatic	0.877096	splicing affected; protein features affected	Donor increased	208095	wt: 0.26 / mu: 0.89	.	.	.	SKAP2	intronic
rs10272303	7	26,827,815	T	T	C	SNV	Transcript	INTRONIC	0.28	0.01	0.296	0.001	0.002	-0.352	0.658	0.717	3.35	2.19	.	.	SKAP2	4/12	.	.	.	0.43	6.88	0.18775	AB	polymorphism_automatic	1	splicing affected; protein features affected	Donor increased	207043	wt: 0.60 / mu: 0.99	.	.	.	SKAP2	intronic
rs3801846	7	26,828,653	C	T	T	SNV	Transcript	INTRONIC	0.34	0.00	0.173	0.000	0.001	-0.487	-0.020	0.113	4.56	0.572	.	.	SKAP2	4/12	.	.	.	0.33	5.94	0.13535	A	polymorphism_automatic	1	.	.	.	.	.	.	.	SKAP2	intronic
rs1327988	7	26,828,965	G	T	C	SNV	Transcript	INTRONIC	0.34	0.00	0.357	0.000	0.001	0.457	-0.137	-0.170	5.37	-1.94	.	.	SKAP2	4/12	.	.	.	0.57	7.94	0.1602	A	polymorphism_automatic	1	splicing affected; protein features affected	Acc gained	205893	0.53	.	.	.	SKAP2	intronic
rs3801844	7	26,829,759	A	G	G	SNV	Transcript	INTRONIC	0.42	0.00	0.950	1.000	1.000	0.651	1.409	2.851	5.69	4.81	4327.8	0	SKAP2	4/12	.	.	.	1.80	14.97	0.95174	A	polymorphism_automatic	2.94712E-16	splicing affected; protein features affected	Donor gained	205104	0.47	yes	yes	.	SKAP2	intronic
rs3801843	7	26,830,201	T	C	C	SNV	Transcript	INTRONIC	0.26	0.00	0.031	0.000	0.000	0.645	-0.231	-0.462	4.86	-0.98	4327.8	0	SKAP2	4/12	.	.	.	-1.00	0.02	0.14345	A	polymorphism_automatic	1	splicing affected; protein features affected	Donor gained	204652	0.67	yes	.	.	SKAP2	intronic
rs3801842	7	26,830,263	C	C	T	SNV	Transcript	INTRONIC	0.28	0.01	0.820	0.003	0.000	-0.386	-0.324	-0.263	4.72	-2.82	4327.8	0	SKAP2	4/12	.	.	.	0.58	8.03	0.08324	A	polymorphism_automatic	1	splicing affected; protein features affected	Donor increased	204591<br>204597	wt: 0.83 / mu: 0.96<br>wt: 0.50 / mu: 0.80	yes	.	.	SKAP2	intronic
rs73065517	7	26,830,555	T	C	C	SNV	Transcript	INTRONIC	0.29	0.01	0.012	0.001	0.007	-0.144	-0.442	-0.443	3.64	-2.02	4327.8	0	SKAP2	4/12	.	.	.	-1.25	0.01	0.11067	A	polymorphism_automatic	1	splicing affected; protein features affected	Donor gained	204298	0.94	.	.	.	SKAP2	intronic
rs28733208	7	26,830,825	G	A	A	SNV	Transcript	INTRONIC	0.28	0.00	0.100	0.000	0.000	-1.528	-0.108	-0.064	3.27	-0.612	.	.	SKAP2	4/12	.	.	.	-0.12	1.58	0.05497	A	polymorphism_automatic	1	.	.	.	.	.	.	.	SKAP2	intronic
rs73065521	7	26,831,409	C	C	A	SNV	Transcript	INTRONIC	0.35	0.01	0.002	0.000	0.000	0.557	-0.597	-0.487	3.1	-3.07	.	.	SKAP2	4/12	.	.	.	0.01	2.65	0.10977	A	polymorphism_automatic	1	splicing affected; protein features affected	Acc gained<br>Acc increased	203455<br>203456	0.39<br>wt: 0.54 / mu: 0.89	.	.	.	SKAP2	intronic
rs3801840	7	26,832,986	A	A	G	SNV	Transcript	INTRONIC	0.42	0.00	0.019	0.000	0.000	-1.154	-0.304	-0.356	5.25	-1.12	.	.	SKAP2	4/12	.	.	.	-0.17	1.27	0.15948	AD	polymorphism_automatic	1	splicing affected; protein features affected	Donor gained	201872	0.64	.	.	.	SKAP2	intronic
rs1327989	7	26,833,563	C	T	T	SNV	Transcript	INTRONIC	0.37	0.00	0.071	0.014	0.001	0.478	0.186	0.013	3.98	-0.236	.	.	SKAP2	4/12	.	.	.	0.27	5.39	0.04862	A	polymorphism_automatic	1	splicing affected; protein features affected	Acc gained	201298	0.58	.	.	.	SKAP2	intronic
rs6964094	7	26,834,380	C	C	G	SNV	Transcript	INTRONIC	0.51	0.01	0.161	0.158	0.190	0.307	0.302	0.311	0.235	0.235	.	.	SKAP2	4/12	.	.	.	0.23	4.95	0.14294	A	polymorphism_automatic	0.999998	splicing affected; protein features affected	Donor gained	200479	0.35	.	.	.	SKAP2	intronic

rs6956088	7	26,835,727	T	A	A	SNV	Transcript	INTRONIC	0.27	0.03	0.043	0.050	0.076	0.455	0.466	0.492	0.559	0.559	.	.	SKAP2	4/12	.	.	.	0.34	6.05	0.04897	A	polymorphism_automatic	1	splicing affected; protein features affected	Acc increased<br>Acc gained	199139<br>199141	wt: 0.65 / mu: 0.94<br>0.52	.	.	.	SKAP2	intronic
rs10276471	7	26,837,940	C	G	G	SNV	Transcript	INTRONIC	0.37	0.00	0.020	0.020	0.023	0.198	0.195	0.202	0.158	0.158	.	.	SKAP2	4/12	.	.	.	-0.24	0.89	0.03267	AB	polymorphism_automatic	1	.	.	.	.	.	.	.	SKAP2	intronic
rs4722641	7	26,838,677	C	C	G	SNV	RegulatoryFeature	REGULATORY	0.58	0.01	0.038	0.037	0.040	0.177	0.174	0.181	0.14	0.14	.	.	.	.	.	.	.	0.05	3.06	0.07605	ADB	polymorphism_automatic	1	splicing affected; protein features affected	Acc increased<br>Acc gained	196188<br>196193	wt: 0.29 / mu: 0.65<br>0.44	.	.	.	SKAP2	intronic
rs10225924	7	26,839,954	C	C	T	SNV	Transcript	INTRONIC	0.35	0.03	0.211	0.213	0.264	0.164	0.162	0.169	0.13	0.13	.	.	SKAP2	4/12	.	.	.	0.40	6.63	0.17155	AB	polymorphism_automatic	0.999998	splicing affected; protein features affected	Acc increased	194908	wt: 0.69 / mu: 0.79	.	.	.	SKAP2	intronic
rs10229837	7	26,840,652	G	G	A	SNV	Transcript	INTRONIC	0.30	0.03	0.040	0.040	0.049	0.164	0.162	0.169	0.13	0.13	.	.	SKAP2	4/12	.	.	.	-0.12	1.60	0.07505	AB	polymorphism_automatic	1	splicing affected; protein features affected	Donor gained<br>Acc increased	194200<br>194216	0.34<br>wt: 0.54 / mu: 0.74	.	.	.	SKAP2	intronic
rs12532395	7	26,841,702	G	G	A	SNV	Transcript	INTRONIC	0.36	0.01	0.081	0.082	0.106	-1.360	-1.338	-1.277	0.149	-0.298	.	.	SKAP2	4/12	.	.	.	-0.42	0.35	0.05934	AB	polymorphism_automatic	1	.	.	.	.	.	.	.	SKAP2	intronic
rs12539719	7	26,844,852	A	A	T	SNV	Transcript	INTRONIC	0.30	0.00	0.011	0.054	0.078	0.450	1.222	1.284	2.12	2.12	.	.	SKAP2	4/12	.	.	.	-0.13	1.54	0.23969	A	polymorphism_automatic	0.999988	.	.	.	.	.	.	.	SKAP2	intronic
rs4722644	7	26,845,386	C	C	A	SNV	Transcript	INTRONIC	0.34	0.00	0.002	0.000	0.001	-0.529	-0.021	-0.002	2.04	0.0268	.	.	SKAP2	4/12	.	.	.	-0.13	1.53	0.08827	A	polymorphism_automatic	1	splicing affected; protein features affected	Donor increased	189478	wt: 0.36 / mu: 0.56	.	.	.	SKAP2	intronic
rs12333525	7	26,847,762	C	C	T	SNV	Transcript	INTRONIC	0.34	0.00	0.054	0.688	0.724	0.432	0.877	0.858	1.01	1.01	.	.	SKAP2	4/12	.	.	.	0.42	6.75	0.70205	A	polymorphism_automatic	0.0117238	splicing affected; protein features affected	Donor gained	187093	0.78	.	.	.	SKAP2	intronic
rs10244986	7	26,849,558	C	C	A	SNV	Transcript	INTRONIC	0.33	0.00	0.077	0.002	0.003	-1.133	-1.272	-1.274	0.6	-1.2	.	.	SKAP2	4/12	.	.	.	-0.20	1.09	0.03895	A	polymorphism_automatic	1	splicing affected; protein features affected	Acc gained	185312	0.5	.	.	.	SKAP2	intronic
rs73067435	7	26,850,195	T	T	C	SNV	Transcript	INTRONIC	0.32	0.01	0.156	0.112	0.164	0.334	0.340	0.362	0.361	0.361	.	.	SKAP2	4/12	.	.	.	0.16	4.28	0.12835	A	polymorphism_automatic	0.999998	splicing affected; protein features affected	Acc increased	184663	wt: 0.25 / mu: 0.45	.	.	.	SKAP2	intronic
rs73067437	7	26,850,313	T	T	C	SNV	Transcript	INTRONIC	0.39	0.01	0.034	0.034	0.039	0.166	0.169	0.181	0.158	0.158	.	.	SKAP2	4/12	.	.	.	-0.28	0.75	0.06705	A	polymorphism_automatic	1	splicing affected; protein features affected	Donor increased<br>Donor gained	184540<br>184545	wt: 0.36 / mu: 0.98<br>0.99	.	.	.	SKAP2	intronic
rs57135974	7	26,850,699	C	A	A	SNV	Transcript	INTRONIC	0.33	0.01	0.470	0.006	0.007	0.478	0.044	0.063	2.63	0.194	.	.	SKAP2	4/12	.	.	.	1.12	11.34	0.03564	A	polymorphism_automatic	0.999983	splicing affected; protein features affected	Donor gained	184153	0.84	.	.	.	SKAP2	intronic
rs58760954	7	26,850,776	C	T	T	SNV	Transcript	INTRONIC	0.40	0.01	0.003	0.005	0.006	0.478	0.012	0.043	3.83	0.0715	.	.	SKAP2	4/12	.	.	.	0.09	3.53	0.05557	A	polymorphism_automatic	0.999992	splicing affected; protein features affected	Donor increased	184086	wt: 0.43 / mu: 0.52	.	.	.	SKAP2	intronic
rs73067443	7	26,851,074	A	A	G	SNV	Transcript	INTRONIC	0.40	0.00	0.032	0.000	0.000	0.478	-0.695	-0.887	4.65	-4.91	.	.	SKAP2	4/12	.	.	.	0.28	5.53	0.08395	A	polymorphism_automatic	1	splicing affected; protein features affected	Donor increased	183786	wt: 0.34 / mu: 0.85	.	.	.	SKAP2	intronic
rs3801837	7	26,851,361	A	G	G	SNV	Transcript	INTRONIC	0.30	0.00	0.032	0.007	0.001	-0.347	0.669	0.391	5.13	2.17	.	.	SKAP2	4/12	.	.	.	-0.03	2.31	0.06382	A	polymorphism_automatic	1	.	.	.	.	.	.	.	SKAP2	intronic
rs3801836	7	26,851,400	C	T	T	SNV	Transcript	INTRONIC	0.28	0.00	0.817	0.000	0.000	0.398	-1.033	-0.762	4.71	-5.24	.	.	SKAP2	4/12	.	.	.	1.74	14.64	0.07101	A	polymorphism_automatic	1	splicing affected; protein features affected	Acc increased	183462	wt: 0.55 / mu: 0.73	yes	.	.	SKAP2	intronic
rs3801834	7	26,852,062	T	T	C	SNV	Transcript	INTRONIC	0.30	0.01	0.348	0.937	0.641	0.474	0.848	1.346	5.33	4.16	1877.1	6.78654e-148	SKAP2	4/12	.	.	.	0.83	9.67	0.84755	A	polymorphism_automatic	0.14345	splicing affected; protein features affected	Donor gained	182796	0.45	yes	yes	.	SKAP2	intronic
rs3801833	7	26,852,212	C	T	T	SNV	Transcript	INTRONIC	0.26	0.04	0.447	0.001	0.000	0.525	0.080	-0.033	5.63	-0.0368	.	.	SKAP2	4/12	.	.	.	1.05	10.93	0.10483	A	polymorphism_automatic	0.999999	splicing affected; protein features affected	Donor increased<br>Acc gained	182645<br>182650	wt: 0.65 / mu: 0.85<br>0.36	.	.	.	SKAP2	intronic
rs3801832	7	26,852,280	C	T	T	SNV	Transcript	INTRONIC	0.24	0.04	0.106	0.001	0.000	0.488	0.417	0.653	5.56	3.21	.	.	SKAP2	4/12	.	.	.	0.34	6.09	0.08593	A	polymorphism_automatic	1	splicing affected; protein features affected	Donor increased	182577	wt: 0.60 / mu: 0.76	.	.	.	SKAP2	intronic
rs7802715	7	26,852,720	C	C	A	SNV	Transcript	INTRONIC	0.24	0.00	0.005	0.000	0.000	-0.353	0.010	-0.022	5.87	1.31	5799	0	SKAP2	4/12	.	.	.	-1.33	0.00	0.22493	A	polymorphism_automatic	1	.	.	.	.	.	.	.	SKAP2	intronic
rs7785815	7	26,852,728	T	A	A	SNV	Transcript	INTRONIC	0.25	0.00	0.037	0.002	0.001	-0.261	0.181	0.547	5.87	0.802	5799	0	SKAP2	4/12	.	.	.	-0.82	0.04	0.11139	A	polymorphism_automatic	1	.	.	.	.	.	.	.	SKAP2	intronic
rs114978183	7	26,852,740	A	C	T	SNV	Transcript	INTRONIC	0.25	0.00	0.043	0.000	0.000	-0.155	-0.080	-0.123	5.61	-0.463	5799	0	SKAP2	4/12	.	.	.	-1.28	0.01	0.21721	AB	polymorphism_automatic	1	splicing affected; protein features affected	Donor increased	182120	wt: 0.44 / mu: 0.68	.	.	.	SKAP2	intronic
rs115453073	7	26,852,741	A	A	T	SNV	Transcript	INTRONIC	0.25	0.00	0.049	0.001	0.000	-0.295	0.488	0.247	5.61	3.23	5799	0	SKAP2	4/12	.	.	.	-1.25	0.01	0.23737	AB	polymorphism_automatic	1	.	.	.	.	.	.	.	SKAP2	intronic
rs7785832	7	26,852,760	T	T	C	SNV	Transcript	INTRONIC	0.28	0.00	0.637	0.992	0.902	0.530	0.167	-0.046	5.87	0.327	5799	0	SKAP2	4/12	.	.	.	0.58	8.00	0.93457	AB	polymorphism_automatic	2.15872E-05	splicing affected; protein features affected	Donor gained	182098	0.56	yes	yes	.	SKAP2	intronic
rs17315334	7	26,852,860	G	G	A	SNV	Transcript	INTRONIC	0.34	0.00	0.922	0.993	0.999	0.651	0.437	0.574	5.78	2.98	5799	0	SKAP2	4/12	.	.	.	1.35	12.54	0.96885	AB	polymorphism_automatic	3.37534E-09	.	.	.	.	yes	yes	.	SKAP2	intronic
rs73067456	7	26,852,998	G	A	A	SNV	Transcript	INTRONIC	0.30	0.01	0.000	0.000	0.000	-0.269	0.135	-0.077	5.66	0.506	5799	0	SKAP2	4/12	.	.	.	-1.36	0.00	0.16542	AB	polymorphism_automatic	1	splicing affected; protein features affected	Acc increased	181871	wt: 0.58 / mu: 0.82	.	.	.	SKAP2	intronic
rs3801831	7	26,853,470	T	C	C	SNV	RegulatoryFeature	REGULATORY	0.30	0.01	0.985	1.000	1.000	0.587	2.619	4.215	5.28	5.28	5799	0	.	.	.	.	.	0.91	10.11	0.95807	AB	polymorphism_automatic	1.07106E-23	splicing affected; protein features affected	Donor gained	181388	0.73	yes	yes	.	SKAP2	intronic
rs3823938	7	26,853,741	A	A	G	SNV	Transcript	INTRONIC	0.24	0.01	0.964	0.997	0.588	0.439	0.512	0.382	5.62	2.04	5799	0	SKAP2	4/12	.	.	.	1.19	11.70	0.75588	AB	polymorphism_automatic	0.0433415	splicing affected; protein features affected	Donor increased	181117	wt: 0.41 / mu: 0.63	yes	yes	.	SKAP2	intronic
rs3823936	7	26,853,830	A	G	G	SNV	Transcript	INTRONIC	0.30	0.00	0.993	1.000	1.000	0.587	1.539	2.007	5.68	4.81	5799	0	SKAP2	4/12	.	.	.	1.63	14.00	0.904	A	polymorphism_automatic	1.74692E-12	splicing affected; protein features affected	Donor increased	181024	wt: 0.66 / mu: 0.91	yes	yes	.	SKAP2	intronic
rs3823934	7	26,853,934	T	T	C	SNV	Transcript	INTRONIC	0.37	0.00	0.977	1.000	0.998	0.530	1.120	0.605	5.77	4.63	5799	0	SKAP2	4/12	.	.	.	1.35	12.52	0.96867	AD	polymorphism_automatic	7.00975E-08	splicing affected; protein features affected	Donor increased	180919	wt: 0.51 / mu: 0.98	yes	yes	.	SKAP2	intronic
rs3823933	7	26,853,966	T	C	C	SNV	Transcript	INTRONIC	0.36	0.00	0.959	0.995	0.996	-0.144	0.170	0.640	5.77	2.09	5799	0	SKAP2	4/12	.	.	.	1.24	11.98	0.96503	AD	polymorphism_automatic	2.05969E-07	splicing affected; protein features affected	Donor increased	180887	wt: 0.42 / mu: 0.97	yes	yes	.	SKAP2	intronic
rs3823932	7	26,853,980	T	T	C	SNV	Transcript	INTRONIC	0.34	0.00	0.957	0.996	0.990	0.530	1.120	0.435	5.77	3.39	5799	0	SKAP2	4/12	.	.	.	1.38	12.70	0.96687	AD	polymorphism_automatic	3.60127E-07	splicing affected; protein features affected	Donor gained	180873	0.84	yes	yes	.	SKAP2	intronic
rs3823931	7	26,854,026	T	C	C	SNV	Transcript	INTRONIC	0.32	0.00	0.045	0.000	0.005	-0.927	-0.904	-0.850	0.694	-1.39	5799	0	SKAP2	4/12	.	.	.	-1.70	0.00	0.0689	A	polymorphism_automatic	1	.	.	.	.	.	.	.	SKAP2	intronic
rs10262414	7	26,854,266	C	T	T	SNV	Transcript	INTRONIC	0.26	0.00	0.028	0.001	0.000	-0.267	0.346	0.302	5.03	1.37	5799	0	SKAP2	4/12	.	.	.	-1.24	0.01	0.09174	AD	polymorphism_automatic	1	.	.	.	.	.	.	.	SKAP2	intronic
rs12532199	7	26,854,375	A	A	G	SNV	Transcript	INTRONIC	0.25	0.01	0.350	0.999	0.863	0.525	2.132	1.158	4.77	3.63	.	.	SKAP2	4/12	.	.	.	1.36	12.58	0.94221	A	polymorphism_automatic	2.26626E-09	splicing affected; protein features affected	Donor increased	180488	wt: 0.51 / mu: 0.78	.	yes	.	SKAP2	intronic
rs12537323	7	26,854,388	C	T	T	SNV	Transcript	INTRONIC	0.27	0.01	0.021	0.000	0.000	0.525	-0.449	-0.402	4.67	-3.96	.	.	SKAP2	4/12	.	.	.	0.14	3.99	0.1039	A	polymorphism_automatic	1	.	.	.	.	.	.	.	SKAP2	intronic
rs28617235	7	26,854,671	A	A	G	SNV	Transcript	INTRONIC	0.25	0.00	0.028	0.000	0.000	0.525	-0.187	-0.143	4.96	-0.716	.	.	SKAP2	4/12	.	.	.	0.00	2.57	0.19236	A	polymorphism_automatic	1	splicing affected; protein features affected	Donor gained	180188	0.46	.	.	.	SKAP2	intronic
rs12532796	7	26,854,828	A	A	G	SNV	Transcript	INTRONIC	0.32	0.01	0.073	0.001	0.000	0.374	-0.361	-0.285	5.61	-3.37	.	.	SKAP2	4/12	.	.	.	0.33	6.01	0.17325	A	polymorphism_automatic	1	splicing affected; protein features affected	Donor increased	180026	wt: 0.24 / mu: 0.58	.	.	.	SKAP2	intronic
rs3801830	7	26,854,870	A	A	G	SNV	Transcript	INTRONIC	0.30	0.01	0.312	0.000	0.000	0.473	-0.351	-0.010	5.55	-1.21	359.7	5.42336e-13	SKAP2	4/12	.	.	.	0.60	8.14	0.18177	A	polymorphism_automatic	1	splicing affected; protein features affected	Acc increased<br>Donor increased	179981<br>179984	wt: 0.53 / mu: 0.68<br>wt: 0.53 / mu: 0.66	.	.	.	SKAP2	intronic
rs3801829	7	26,854,996	G	G	C	SNV	Transcript	INTRONIC	0.34	0.00	0.026	0.006	0.000	0.567	0.089	0.037	4.9	1.1	359.7	5.42336e-13	SKAP2	4/12	.	.	.	-0.26	0.80	0.18684	A	polymorphism_automatic	1	splicing affected; protein features affected	Donor gained	179862	0.6	.	.	.	SKAP2	intronic
rs10266759	7	26,855,282	G	G	A	SNV	Transcript	INTRONIC	0.43	0.03	0.985	1.000	1.000	0.581	2.886	2.250	5.77	5.77	1218.2	3.54978e-180	SKAP2	4/12	.	.	.	2.52	19.60	0.93415	ADC	polymorphism_automatic	1.79076E-18	splicing affected; protein features affected	Acc increased	179583	wt: 0.78 / mu: 0.87	yes	yes	.	SKAP2	intronic
rs3801828	7	26,855,284	C	A	A	SNV	Transcript	INTRONIC	0.42	0.03	0.970	1.000	1.000	0.473	1.099	0.956	5.77	4.61	1218.2	3.54978e-180	SKAP2	4/12	.	.	.	2.33	18.36	0.88818	ADC	polymorphism_automatic	1.98924E-11	splicing affected; protein features affected	Acc gained<br>Acc increased	179580<br>179583	0.76<br>wt: 0.78 / mu: 0.94	yes	yes	.	SKAP2	intronic

rs6948584	7	26,855,732	G	A	A	SNV	Transcript	INTRONIC	0.29	0.01	0.123	0.001	0.001	0.249	0.107	0.132	1.74	0.435	.	.	SKAP2	4/12	.	.	.	0.23	4.97	0.1036	A	polymorphism_automatic	1	splicing affected; protein features affected	Donor increased	179120	wt: 0.89 / mu: 0.99	.	.	.	SKAP2	intronic
rs6948657	7	26,855,943	C	C	T	SNV	Transcript	INTRONIC	0.28	0.00	0.018	0.003	0.004	0.581	0.913	0.554	5.77	2.85	.	.	SKAP2	4/12	.	.	.	0.15	4.10	0.20995	A	polymorphism_automatic	1	splicing affected; protein features affected	Donor gained	178911	0.93	.	.	.	SKAP2	intronic
rs73067472	7	26,855,959	T	T	C	SNV	Transcript	INTRONIC	0.28	0.00	0.409	0.000	0.001	0.473	-0.439	-0.020	5.77	-1.83	.	.	SKAP2	4/12	.	.	.	0.93	10.28	0.20433	A	polymorphism_automatic	1	.	.	.	.	.	.	.	SKAP2	intronic
rs3823930	7	26,856,525	T	T	C	SNV	Transcript	INTRONIC	0.36	0.00	0.163	0.000	0.000	0.473	-1.132	-1.060	4.11	-5.83	619.1	3.6682e-10	SKAP2	4/12	.	.	.	0.19	4.60	0.11132	A	polymorphism_automatic	1	splicing affected; protein features affected	Donor gained	178333	0.84	.	.	.	SKAP2	intronic
rs3823929	7	26,856,600	T	C	C	SNV	Transcript	INTRONIC	0.40	0.00	0.006	0.000	0.000	-0.334	-0.547	-0.306	4.77	-2.87	619.1	3.6682e-10	SKAP2	4/12	.	.	.	-0.16	1.33	0.12304	A	polymorphism_automatic	1	splicing affected; protein features affected	Acc gained	178268	0.31	yes	.	.	SKAP2	intronic
rs3801827	7	26,856,626	C	T	T	SNV	Transcript	INTRONIC	0.35	0.00	0.503	0.002	0.003	-0.782	-0.744	-0.486	4.27	-3.63	619.1	3.6682e-10	SKAP2	4/12	.	.	.	0.87	9.90	0.05907	A	polymorphism_automatic	1	splicing affected; protein features affected	Donor gained	178229	0.47	yes	.	.	SKAP2	intronic
rs3801826	7	26,856,720	C	C	T	SNV	Transcript	INTRONIC	0.30	0.00	0.263	0.000	0.000	0.581	-0.753	-0.544	4.87	-2.83	619.1	3.6682e-10	SKAP2	4/12	.	.	.	0.59	8.12	0.12099	A	polymorphism_automatic	1	splicing affected; protein features affected	Donor increased	178131	wt: 0.21 / mu: 0.33	.	.	.	SKAP2	intronic
rs3801825	7	26,856,959	T	T	A	SNV	Transcript	INTRONIC	0.30	0.01	0.211	0.890	0.927	0.478	0.916	0.981	4.06	2.92	.	.	SKAP2	4/12	.	.	.	1.31	12.34	0.94993	A	polymorphism_automatic	1.0035E-06	splicing affected; protein features affected	Acc gained	177902	0.9	.	yes	.	SKAP2	intronic
rs73067475	7	26,857,120	C	T	T	SNV	Transcript	INTRONIC	0.30	0.00	0.043	0.002	0.002	0.455	0.703	0.766	3.61	2.39	.	.	SKAP2	4/12	.	.	.	0.17	4.31	0.05087	A	polymorphism_automatic	1	splicing affected; protein features affected	Donor increased	177739	wt: 0.67 / mu: 0.97	.	.	.	SKAP2	intronic
rs73067476	7	26,857,240	A	A	G	SNV	Transcript	INTRONIC	0.25	0.00	0.016	0.001	0.002	0.530	1.607	1.684	3	3	.	.	SKAP2	4/12	.	.	.	0.06	3.18	0.24848	A	polymorphism_automatic	0.999742	splicing affected; protein features affected	Donor increased	177611	wt: 0.26 / mu: 0.52	.	.	.	SKAP2	intronic
rs115447617	7	26,857,751	T	C	C	SNV	Transcript	INTRONIC	0.37	0.03	0.013	0.000	0.000	0.300	-0.273	-0.253	0.562	-0.517	.	.	SKAP2	4/12	.	.	.	-0.28	0.74	0.0333	A	.	.	.	.	.	.	.	.	.	SKAP2	intronic
rs111373218	7	26,859,152	G	G	A	SNV	Transcript	INTRONIC	0.40	0.03	0.004	0.004	0.005	-1.360	-1.338	-1.277	0.149	-0.298	.	.	SKAP2	4/12	.	.	.	-0.66	0.09	0.0343	A	polymorphism_automatic	1	splicing affected; protein features affected	Donor gained	175700	0.55	.	.	.	SKAP2	intronic
rs17315775	7	26,860,134	T	C	C	SNV	Transcript	INTRONIC	0.30	0.01	0.001	0.000	0.000	-0.150	0.065	0.060	3.17	0.378	.	.	SKAP2	4/12	.	.	.	-0.12	1.60	0.08043	A	polymorphism_automatic	1	splicing affected; protein features affected	Donor gained	174719	0.91	.	.	.	SKAP2	intronic
rs28404962	7	26,860,324	T	T	C	SNV	Transcript	INTRONIC	0.28	0.01	0.792	0.010	0.016	0.525	-0.277	-0.230	3.78	-1.61	.	.	SKAP2	4/12	.	.	.	1.68	14.28	0.12747	A	polymorphism_automatic	1	splicing affected; protein features affected	Acc increased	174534	wt: 0.29 / mu: 0.66	.	.	.	SKAP2	intronic
rs13438513	7	26,860,633	G	C	C	SNV	Transcript	INTRONIC	0.29	0.01	0.037	0.000	0.000	-0.896	-1.587	-1.432	4.46	-7.77	.	.	SKAP2	4/12	.	.	.	-0.43	0.33	0.05604	A	polymorphism_automatic	0.999956	.	.	.	.	.	.	.	SKAP2	intronic
rs13438514	7	26,860,688	G	A	A	SNV	Transcript	INTRONIC	0.30	0.01	0.025	0.000	0.001	0.374	-0.222	-0.166	3.93	-1.27	.	.	SKAP2	4/12	.	.	.	0.05	3.05	0.07063	A	polymorphism_automatic	1	.	.	.	.	.	.	.	SKAP2	intronic
rs55739485	7	26,860,741	T	C	C	SNV	Transcript	INTRONIC	0.29	0.01	0.027	0.357	0.516	0.581	2.457	2.444	3.89	3.89	.	.	SKAP2	4/12	.	.	.	0.37	6.39	0.35712	A	polymorphism_automatic	0.04167	splicing affected; protein features affected	Donor gained	174112	0.96	.	.	.	SKAP2	intronic
rs3801823	7	26,861,031	T	T	C	SNV	Transcript	INTRONIC	0.32	0.00	0.109	0.020	0.038	0.478	2.286	2.378	4.59	4.59	640.1	1.8881e-26	SKAP2	4/12	.	.	.	0.40	6.60	0.40566	A	polymorphism_automatic	0.0481642	splicing affected; protein features affected	Acc gained	173837	0.4	yes	.	.	SKAP2	intronic
rs3801822	7	26,861,201	T	C	C	SNV	Transcript	INTRONIC	0.36	0.01	0.012	0.966	0.977	-0.236	0.718	0.703	4.24	2.4	640.1	1.8881e-26	SKAP2	4/12	.	.	.	0.32	5.90	0.92022	A	polymorphism_automatic	3.73786E-09	splicing affected; protein features affected	Donor gained	173652	0.54	yes	yes	.	SKAP2	intronic
rs3801821	7	26,861,206	C	T	T	SNV	Transcript	INTRONIC	0.35	0.01	0.013	0.976	0.984	0.478	0.933	0.994	4.24	3.05	640.1	1.8881e-26	SKAP2	4/12	.	.	.	0.58	8.03	0.91725	A	polymorphism_automatic	7.64236E-08	splicing affected; protein features affected	Donor increased	173655	wt: 0.55 / mu: 0.92	yes	yes	.	SKAP2	intronic
rs3801820	7	26,861,353	A	G	G	SNV	Transcript	INTRONIC	0.38	0.01	0.305	0.409	0.494	0.538	0.177	0.196	4.28	0.967	.	.	SKAP2	4/12	.	.	.	0.96	10.46	0.50263	A	polymorphism_automatic	0.0368784	splicing affected; protein features affected	Donor increased	173503	wt: 0.27 / mu: 0.46	.	.	.	SKAP2	intronic
rs7785689	7	26,861,609	G	A	A	SNV	Transcript	INTRONIC	0.26	0.00	0.013	0.013	0.017	0.434	0.205	0.224	1.9	0.703	.	.	SKAP2	4/12	.	.	.	-0.15	1.37	0.04861	AD	polymorphism_automatic	0.999999	.	.	.	.	.	.	.	SKAP2	intronic
rs7785711	7	26,861,640	G	G	A	SNV	Transcript	INTRONIC	0.28	0.00	0.077	0.000	0.000	0.384	-1.093	-1.032	1.36	-2.72	.	.	SKAP2	4/12	.	.	.	-0.04	2.25	0.09753	AD	polymorphism_automatic	1	.	.	.	.	.	.	.	SKAP2	intronic
rs7785798	7	26,861,943	A	A	G	SNV	Transcript	INTRONIC	0.31	0.00	0.267	0.292	0.412	-0.489	-0.438	-0.368	0.593	-1.1	.	.	SKAP2	4/12	.	.	.	0.30	5.72	0.27419	A	polymorphism_automatic	0.999708	splicing affected; protein features affected	Donor increased	172916	wt: 0.78 / mu: 0.92	.	.	.	SKAP2	intronic
rs7785918	7	26,861,959	C	C	T	SNV	Transcript	INTRONIC	0.30	0.00	0.002	0.002	0.004	0.581	0.575	0.584	0.593	0.593	.	.	SKAP2	4/12	.	.	.	-0.11	1.66	0.07049	A	polymorphism_automatic	1	splicing affected; protein features affected	Donor increased	172898	wt: 0.78 / mu: 0.90	.	.	.	SKAP2	intronic
rs12540045	7	26,863,174	C	C	G	SNV	Transcript	INTRONIC	0.44	0.00	0.031	0.032	0.045	0.399	0.394	0.405	0.351	0.351	.	.	SKAP2	4/12	.	.	.	-0.04	2.18	0.07512	A	polymorphism_automatic	1	splicing affected; protein features affected	Acc gained	171696	0.44	.	.	.	SKAP2	intronic
rs10248500	7	26,863,294	A	C	C	SNV	Transcript	INTRONIC	0.42	0.00	0.000	0.000	0.000	-1.604	-1.640	-1.616	0.351	-0.702	.	.	SKAP2	4/12	.	.	.	-0.75	0.05	0.01647	AD	polymorphism_automatic	1	splicing affected; protein features affected	Acc gained	171564	0.76	.	.	.	SKAP2	intronic
rs17315929	7	26,864,173	C	T	T	SNV	Transcript	INTRONIC	0.36	0.00	0.046	0.002	0.004	0.393	-0.069	-0.051	2.25	-0.369	.	.	SKAP2	4/12	.	.	.	-0.01	2.47	0.05298	A	polymorphism_automatic	1	splicing affected; protein features affected	Donor increased	170681	wt: 0.52 / mu: 0.99	.	.	.	SKAP2	intronic
rs10227673	7	26,865,299	G	A	A	SNV	Transcript	INTRONIC	0.27	0.00	0.006	0.002	0.002	0.365	0.499	0.521	0.531	0.531	.	.	SKAP2	4/12	.	.	.	-0.14	1.43	0.02645	A	polymorphism_automatic	1	splicing affected; protein features affected	Acc increased	169569	wt: 0.65 / mu: 0.84	.	.	.	SKAP2	intronic
rs7805766	7	26,865,610	C	G	G	SNV	Transcript	INTRONIC	0.44	0.00	0.464	0.027	0.041	0.457	0.128	0.127	2.6	0.548	.	.	SKAP2	4/12	.	.	.	0.96	10.42	0.06133	A	polymorphism_automatic	0.999999	splicing affected; protein features affected	Donor gained	169251	0.48	.	.	.	SKAP2	intronic
rs17375272	7	26,866,497	C	C	T	SNV	Transcript	INTRONIC	0.25	0.01	0.003	0.007	0.010	-1.596	-1.305	-1.293	3.32	-3.34	372.5	9.05291e-09	SKAP2	4/12	.	.	.	-0.48	0.25	0.08045	A	polymorphism_automatic	0.99999	.	.	.	.	.	.	.	SKAP2	intronic
rs3801819	7	26,866,698	T	T	G	SNV	Transcript	INTRONIC	0.28	0.01	0.051	0.000	0.001	0.398	-0.141	-0.094	3.39	-0.803	372.5	9.05291e-09	SKAP2	4/12	.	.	.	0.16	4.21	0.12713	A	polymorphism_automatic	1	.	.	.	.	yes	.	.	SKAP2	intronic
rs61354389	7	26,868,343	C	T	T	SNV	Transcript	INTRONIC	0.49	0.07	0.046	0.006	0.007	0.157	0.402	0.419	0.431	0.431	.	.	SKAP2	4/12	.	.	.	-0.08	1.91	0.01906	A	polymorphism_automatic	1	splicing affected; protein features affected	Donor gained	166518	0.47	.	.	.	SKAP2	intronic
rs10225700	7	26,869,880	C	T	T	SNV	Transcript	INTRONIC	0.39	0.01	0.051	0.000	0.000	0.148	-0.481	-0.409	2.24	-2.1	.	.	SKAP2	4/12	.	.	.	0.04	3.01	0.04276	A	polymorphism_automatic	0.999999	.	.	.	.	.	.	.	SKAP2	intronic
rs10241234	7	26,869,887	T	T	A	SNV	Transcript	INTRONIC	0.38	0.01	0.071	0.000	0.000	0.158	-0.245	-0.185	2.28	-1.25	.	.	SKAP2	4/12	.	.	.	0.56	7.87	0.07827	A	polymorphism_automatic	1	splicing affected; protein features affected	Donor gained<br>Acc gained	164965<br>164978	0.35<br>0.43	.	.	.	SKAP2	intronic
rs10225904	7	26,869,888	G	G	A	SNV	Transcript	INTRONIC	0.38	0.01	0.070	0.000	0.000	0.188	-0.253	-0.244	2.28	-0.92	.	.	SKAP2	4/12	.	.	.	0.08	3.38	0.07892	A	polymorphism_automatic	1	.	.	.	.	.	.	.	SKAP2	intronic
rs28550029	7	26,872,581	C	T	T	SNV	Transcript	INTRONIC	0.47	0.03	0.004	0.000	0.000	-0.658	-0.849	-0.753	2.72	-4.06	.	.	SKAP2	4/12	.	.	.	-0.15	1.40	0.04785	AB	polymorphism_automatic	1	splicing affected; protein features affected	Donor increased	162274	wt: 0.20 / mu: 0.85	.	.	.	SKAP2	intronic
rs114236648	7	26,874,639	A	G	G	SNV	Transcript	INTRONIC	0.38	0.01	0.072	0.071	0.088	0.176	0.173	0.179	0.109	0.109	.	.	SKAP2	4/12	.	.	.	-0.02	2.40	0.03193	A	.	.	.	.	.	.	.	.	.	SKAP2	intronic
rs12534899	7	26,877,189	T	C	C	SNV	Transcript	INTRONIC	0.52	0.03	0.053	0.056	0.071	0.299	0.294	0.303	0.225	0.225	.	.	SKAP2	4/12	.	.	.	0.02	2.79	0.04385	AB	polymorphism_automatic	1	splicing affected; protein features affected	Acc increased	157677	wt: 0.67 / mu: 0.80	.	.	.	SKAP2	intronic
rs12534921	7	26,877,210	T	C	C	SNV	Transcript	INTRONIC	0.49	0.03	0.038	0.075	0.096	0.299	0.294	0.303	0.225	0.225	.	.	SKAP2	4/12	.	.	.	0.01	2.69	0.04904	AB	polymorphism_automatic	1	.	.	.	.	.	.	.	SKAP2	intronic
rs73069507	7	26,877,536	A	G	G	SNV	Transcript	INTRONIC	0.42	0.00	0.005	0.000	0.000	-0.310	-0.524	-0.502	2.89	-2.21	.	.	SKAP2	4/12	.	.	.	-0.17	1.24	0.05896	AB	polymorphism_automatic	1	splicing affected; protein features affected	Acc increased	157318	wt: 0.49 / mu: 0.62	.	.	.	SKAP2	intronic
rs73069510	7	26,877,567	T	T	C	SNV	Transcript	INTRONIC	0.39	0.00	0.066	0.997	0.999	0.473	1.477	1.557	2.7	2.7	.	.	SKAP2	4/12	.	.	.	0.71	8.88	0.95445	AB	polymorphism_automatic	3.80573E-12	splicing affected; protein features affected	Donor increased	157286	wt: 0.28 / mu: 0.99	.	yes	.	SKAP2	intronic
rs3801816	7	26,881,177	G	A	A	SNV	Transcript	INTRONIC	0.36	0.01	0.994	1.000	1.000	0.478	2.286	3.682	5.68	5.68	5863.9	0	SKAP2	4/12	.	.	.	1.73	14.55	0.8965	AC	polymorphism_automatic	3.42867E-23	.	.	.	.	yes	yes	.	SKAP2	intronic
rs3801815	7	26,881,292	T	C	C	SNV	Transcript	INTRONIC	0.38	0.00	0.983	0.999	1.000	0.587	0.370	1.344	5.58	2.73	5863.9	0	SKAP2	4/12	.	.	.	1.49	13.24	0.88127	AC	polymorphism_automatic	4.3743E-14	splicing affected; protein features affected	Acc increased	153563	wt: 0.51 / mu: 0.70	yes	yes	.	SKAP2	intronic

rs3801814	7	26,881,302	T	G	G	SNV	Transcript	INTRONIC	0.36	0.00	0.958	1.000	1.000	0.587	2.777	4.367	5.58	5.58	5863.9	0	SKAP2	4/12	.	.	.	1.85	15.27	0.90021	AC	polymorphism_automatic	1.38808E-25	splicing affected; protein features affected	Acc gained<br>Acc increased	153559<br>153562	0.60<br>wt: 0.22 / mu: 0.81	yes	yes	.	SKAP2	intronic
rs12535784	7	26,882,067	G	A	A	SNV	Transcript	INTRONIC	0.23	0.00	0.219	0.001	0.002	0.379	1.415	0.800	3.15	2.01	.	.	SKAP2	4/12	.	.	.	0.50	7.47	0.06588	AB	polymorphism_automatic	1	splicing affected; protein features affected	Donor increased	152788	wt: 0.22 / mu: 0.40	.	.	.	SKAP2	intronic
rs4719882	7	26,882,252	A	G	G	SNV	Transcript	INTRONIC	0.27	0.01	0.054	0.000	0.000	-0.393	-0.102	-0.363	4.94	-1.58	.	.	SKAP2	4/12	.	.	.	0.06	3.22	0.15122	AB	polymorphism_automatic	1	.	.	.	.	.	.	.	SKAP2	intronic
rs1982995	7	26,884,135	A	A	C	SNV	Transcript	INTRONIC	0.32	0.01	0.031	0.000	0.000	0.450	-0.914	-0.784	5.03	-4.56	.	.	SKAP2	3/12	.	.	.	0.02	2.74	0.16163	AB	polymorphism_automatic	1	splicing affected; protein features affected	Acc gained	150723	0.8	.	.	.	SKAP2	intronic
rs10236221	7	26,884,671	A	T	T	SNV	Transcript	INTRONIC	0.34	0.01	0.011	0.000	0.000	-0.377	-2.114	-1.942	4.18	-8.36	.	.	SKAP2	3/12	.	.	.	-0.29	0.69	0.05804	A	polymorphism_automatic	1	.	.	.	.	.	.	.	SKAP2	intronic
rs10270187	7	26,885,885	C	G	G	SNV	Transcript	INTRONIC	0.23	0.00	0.001	0.000	0.000	-0.494	-0.482	-0.446	0.556	-0.946	.	.	SKAP2	3/12	.	.	.	-0.40	0.40	0.07207	A	polymorphism_automatic	1	splicing affected; protein features affected	Acc gained<br>Acc increased	148976<br>148981	0.56<br>wt: 0.64 / mu: 0.85	.	.	.	SKAP2	intronic
rs10227529	7	26,886,309	T	A	A	SNV	Transcript	INTRONIC	0.44	0.05	0.004	0.000	0.000	0.249	-0.031	-0.196	1.3	-0.0897	.	.	SKAP2	3/12	.	.	.	0.04	2.96	0.03394	AB	polymorphism_automatic	0.999999	splicing affected; protein features affected	Acc increased<br>Acc gained	148552<br>148554	wt: 0.69 / mu: 0.98<br>0.71	.	.	.	SKAP2	intronic
rs3801813	7	26,887,069	T	T	C	SNV	Transcript	INTRONIC	0.37	0.03	0.033	0.930	0.875	-0.267	0.184	0.140	6.06	0.653	3108.9	0	SKAP2	3/12	.	.	.	-0.38	0.43	0.93633	AB	polymorphism_automatic	3.17609E-08	.	.	.	.	yes	yes	.	SKAP2	intronic
rs3801812	7	26,887,615	C	C	T	SNV	Transcript	INTRONIC	0.30	0.01	0.883	0.727	0.000	0.645	0.435	0.240	6	2.23	3108.9	0	SKAP2	3/12	.	.	.	1.30	12.26	0.23834	AB	polymorphism_automatic	1	splicing affected; protein features affected	Donor gained	147244	0.8	yes	yes	.	SKAP2	intronic
rs10272190	7	26,887,942	T	T	A	SNV	Transcript	INTRONIC	0.32	0.00	0.001	0.000	0.000	-1.130	-1.568	-1.423	1.68	-3.36	.	.	SKAP2	3/12	.	.	.	-0.12	1.57	0.07308	AB	polymorphism_automatic	1	splicing affected; protein features affected	Acc gained	146922	0.81	.	.	.	SKAP2	intronic
rs10227231	7	26,888,074	A	A	G	SNV	Transcript	INTRONIC	0.36	0.03	0.071	0.012	0.015	0.525	-0.079	-0.050	1.16	-0.271	.	.	SKAP2	3/12	.	.	.	0.15	4.13	0.09482	AB	polymorphism_automatic	1	splicing affected; protein features affected	Donor increased	146790	wt: 0.23 / mu: 0.34	.	.	.	SKAP2	intronic
rs10272457	7	26,888,155	T	C	C	SNV	Transcript	INTRONIC	0.37	0.01	0.005	0.001	0.001	-0.255	-0.241	-0.206	0.685	-0.659	.	.	SKAP2	3/12	.	.	.	-0.37	0.47	0.05915	AB	polymorphism_automatic	1	splicing affected; protein features affected	Donor gained	146698	0.82	.	.	.	SKAP2	intronic
rs10272603	7	26,888,277	T	C	C	SNV	Transcript	INTRONIC	0.30	0.00	0.000	0.000	0.000	-0.838	-0.460	-0.458	3.36	-1.89	.	.	SKAP2	3/12	.	.	.	-0.31	0.63	0.05997	AB	polymorphism_automatic	1	splicing affected; protein features affected	Acc increased	146581	wt: 0.50 / mu: 0.75	.	.	.	SKAP2	intronic
rs6461979	7	26,888,918	T	T	C	SNV	Transcript	INTRONIC	0.27	0.00	0.024	0.001	0.001	0.369	0.343	0.391	4	1.58	.	.	SKAP2	3/12	.	.	.	0.06	3.22	0.17888	A	polymorphism_automatic	1	splicing affected; protein features affected	Donor gained	145935	0.96	.	.	.	SKAP2	intronic
rs17376018	7	26,889,862	A	G	G	SNV	Transcript	INTRONIC	0.38	0.05	0.008	0.000	0.000	-0.199	-0.991	-0.972	3.52	-4.28	.	.	SKAP2	3/12	.	.	.	-0.01	2.47	0.09935	ALL	polymorphism_automatic	1	.	.	.	.	.	.	.	SKAP2	intronic
rs4722647	7	26,890,446	G	C	C	SNV	Transcript	INTRONIC	0.28	0.01	0.000	0.000	0.000	-0.304	-0.744	-0.693	3.28	-3.53	.	.	SKAP2	3/12	.	.	.	-0.58	0.14	0.13342	ALL	polymorphism_automatic	1	.	.	.	.	.	.	.	SKAP2	intronic
rs3934268	7	26,890,572	G	C	C	SNV	Transcript	INTRONIC	0.27	0.01	0.000	0.000	0.000	-2.359	-0.733	-0.712	3.92	-3.84	.	.	SKAP2	3/12	.	.	.	-0.84	0.04	0.11558	AB	polymorphism_automatic	1	splicing affected; protein features affected	Acc gained	144286	0.38	.	.	.	SKAP2	intronic
rs2031811	7	26,890,672	T	T	C	SNV	Transcript	INTRONIC	0.31	0.01	0.021	0.001	0.001	0.492	0.019	0.058	2.63	0.203	.	.	SKAP2	3/12	.	.	.	-0.07	2.00	0.14149	AB	polymorphism_automatic	1	splicing affected; protein features affected	Acc increased	144197	wt: 0.65 / mu: 0.83	.	.	.	SKAP2	intronic
rs2030136	7	26,891,016	T	T	C	SNV	Transcript	INTRONIC	0.52	0.04	0.072	0.030	0.035	0.166	-0.360	-0.350	0.539	-0.696	.	.	SKAP2	3/12	.	.	.	-0.01	2.50	0.06226	AB	polymorphism_automatic	1	.	.	.	.	.	.	.	SKAP2	intronic
rs7804356	7	26,891,665	T	T	C	SNV	Transcript	INTRONIC	0.37	0.01	0.150	0.000	0.000	0.530	-0.345	-0.418	4.45	-3.61	.	.	SKAP2	3/12	.	.	.	0.36	6.29	0.1563	AB	polymorphism_automatic	1	splicing affected; protein features affected	Donor gained	143193	0.74	.	.	19430480,Type 1 diabetes,5E-9	SKAP2	intronic
rs7804620	7	26,891,831	T	T	G	SNV	Transcript	INTRONIC	0.30	0.01	0.051	0.000	0.000	0.450	-0.426	-0.489	4.1	-1.8	.	.	SKAP2	3/12	.	.	.	0.23	5.02	0.14792	AB	polymorphism_automatic	1	splicing affected; protein features affected	Acc gained	143032	0.73	.	.	0	SKAP2	intronic
rs73069533	7	26,892,325	G	G	A	SNV	Transcript	INTRONIC	0.28	0.01	0.009	0.224	0.094	0.587	0.317	0.094	5.24	1.41	1135.2	3.74694e-72	SKAP2	3/12	.	.	.	-0.02	2.40	0.24818	AB	polymorphism_automatic	0.997691	splicing affected; protein features affected	Acc increased	142544	wt: 0.50 / mu: 0.76	yes	yes	0	SKAP2	intronic
rs10486483	7	26,892,440	G	A	A	SNV	Transcript	INTRONIC	0.28	0.01	0.299	0.355	0.171	0.379	2.032	2.809	5.04	5.04	1135.2	3.74694e-72	SKAP2	3/12	.	.	.	0.71	8.92	0.11794	AB	polymorphism_automatic	0.999172	splicing affected; protein features affected	Donor increased	142410	wt: 0.39 / mu: 0.55	.	.	23128233,Crohn's disease,3E-8	SKAP2	intronic
rs3801810	7	26,892,531	G	G	A	SNV	Transcript	INTRONIC	0.25	0.03	0.011	0.132	0.000	-0.470	0.401	0.060	5.21	-0.0556	.	.	SKAP2	3/12	.	.	.	-0.06	2.01	0.1642	AB	polymorphism_automatic	1	splicing affected; protein features affected	Acc increased	142330	wt: 0.22 / mu: 0.27	.	.	.	SKAP2	intronic
rs3823928	7	26,893,317	T	C	C	SNV	Transcript	INTRONIC	0.32	0.00	0.006	0.000	0.000	0.462	-0.095	-0.089	4.74	-1.19	.	.	SKAP2	3/12	.	.	.	0.23	4.96	0.2257	ABC	polymorphism_automatic	1	splicing affected; protein features affected	Donor gained	141536	0.87	.	.	.	SKAP2	intronic
rs7791513	7	26,893,357	A	C	C	SNV	RegulatoryFeature	REGULATORY	0.39	0.00	0.187	0.001	0.001	0.651	0.212	0.126	4.56	0.62	.	.	.	.	.	.	.	0.24	5.14	0.18384	ABC	polymorphism_automatic	0.999992	.	.	.	.	.	.	.	SKAP2	intronic
rs3213853	7	26,893,524	T	T	C	SNV	RegulatoryFeature	REGULATORY	0.34	0.03	0.027	0.003	0.001	0.530	0.177	1.006	4.61	2.02	.	.	.	.	.	.	.	0.12	3.81	0.2963	ALL	polymorphism_automatic	0.999956	splicing affected; protein features affected	Acc increased	141344	wt: 0.30 / mu: 0.41	.	.	.	SKAP2	intronic
rs6461980	7	26,894,123	T	T	G	SNV	Transcript	INTRONIC	0.32	0.00	0.024	0.001	0.000	0.530	-0.419	-0.462	4.05	-3.84	.	.	SKAP2	2/12	.	.	.	0.18	4.43	0.17919	AB	polymorphism_automatic	1	.	.	.	.	.	.	.	SKAP2	intronic
rs12536145	7	26,894,860	A	T	T	SNV	RegulatoryFeature	REGULATORY	0.30	0.00	0.024	0.047	0.560	0.398	0.491	1.122	5.95	3.56	1710.5	1.04531e-163	.	.	.	.	.	-0.48	0.24	0.36641	ALL	polymorphism_automatic	0.995936	splicing affected; protein features affected	Donor increased	140004	wt: 0.81 / mu: 0.90	yes	.	.	SKAP2	intronic
rs10242572	7	26,895,065	T	C	C	SNV	RegulatoryFeature	REGULATORY	0.38	0.00	0.001	0.000	0.000	-1.147	-0.369	-0.389	3.5	-1.68	.	.	.	.	.	.	.	-0.48	0.24	0.12772	AB	polymorphism_automatic	0.999999	splicing affected; protein features affected	Donor increased	139793	wt: 0.56 / mu: 0.98	.	.	.	SKAP2	intronic
rs10256250	7	26,895,080	A	A	T	SNV	RegulatoryFeature	REGULATORY	0.40	0.00	0.009	0.001	0.001	0.455	-0.125	0.025	5.22	-0.332	.	.	.	.	.	.	.	-0.26	0.82	0.19558	AB	polymorphism_automatic	1	splicing affected; protein features affected	Donor increased	139774<br>139779	wt: 0.43 / mu: 0.97	.	.	.	SKAP2	intronic
rs3801809	7	26,895,456	G	G	A	SNV	RegulatoryFeature	REGULATORY	0.26	0.00	0.005	0.000	0.000	-0.140	0.115	0.134	3.98	0.718	.	.	.	.	.	.	.	-0.14	1.48	0.17281	AB	polymorphism_automatic	1	splicing affected; protein features affected	Acc increased	139406	wt: 0.60 / mu: 0.77	.	.	.	SKAP2	intronic
rs3801808	7	26,896,555	T	T	A	SNV	RegulatoryFeature	REGULATORY	0.27	0.00	0.922	1.000	1.000	0.457	0.869	1.187	4.53	3.38	559.2	2.58832e-45	.	.	.	.	.	1.89	15.55	0.96911	AB	polymorphism_automatic	9.08982E-13	splicing affected; protein features affected	Donor increased	138296	wt: 0.68 / mu: 0.75	.	yes	.	SKAP2	intronic
rs3801807	7	26,897,439	T	T	C	SNV	RegulatoryFeature	REGULATORY	0.42	0.03	0.113	0.621	0.683	0.530	1.025	1.105	4.57	3.38	446	6.01797e-37	.	.	.	.	.	0.74	9.11	0.73209	ALL	polymorphism_automatic	2.17774E-05	splicing affected; protein features affected	Donor gained	137419	0.92	yes	yes	.	SKAP2	intronic
rs3801805	7	26,897,640	A	A	G	SNV	Transcript	INTRONIC	0.48	0.09	0.310	0.727	0.818	0.487	0.049	0.093	4.21	0.323	.	.	SKAP2	1/12	.	.	.	1.54	13.51	0.75579	ALL	polymorphism_automatic	0.00144294	.	.	.	.	.	yes	.	SKAP2	intronic
rs10486485	7	26,900,356	C	C	A	SNV	Transcript	INTRONIC	0.36	0.01	0.888	0.001	0.000	0.651	0.717	0.528	4.9	2.05	.	.	SKAP2	1/12	.	.	.	2.09	16.80	0.22824	ABC	polymorphism_automatic	1	splicing affected; protein features affected	Donor increased	134498	wt: 0.47 / mu: 0.69	.	.	.	SKAP2	intronic
rs3801803	7	26,901,740	T	T	C	SNV	Transcript	INTRONIC	0.27	0.00	0.026	0.000	0.000	-0.350	-0.364	-0.292	3.76	-2.22	.	.	SKAP2	1/12	.	.	.	-0.15	1.36	0.16241	AB	polymorphism_automatic	1	.	.	.	.	.	.	.	SKAP2	intronic
rs73069539	7	26,903,661	C	C	T	SNV	RegulatoryFeature	REGULATORY	0.50	0.05	0.004	0.000	0.000	0.651	-1.455	-1.384	2.84	-5.43	.	.	.	.	.	.	.	0.84	9.73	0.40417	ALL	polymorphism_automatic	0.999996	splicing affected; protein features affected	Donor gained	131196	0.42	.	.	.	SKAP2	intronic
rs17323934	7	26,904,330	C	C	G	SNV	RegulatoryFeature	REGULATORY	0.67	0.13	0.006	0.000	0.000	0.651	0.357	0.653	5.81	2.76	.	.	.	.	.	.	.	0.69	8.75	0.50418	ALL	polymorphism_automatic	0.999742	splicing affected; protein features affected	Donor increased	130533	wt: 0.75 / mu: 0.90	.	.	.	SKAP2	5'-UTR
rs73069540	7	26,904,770	C	C	T	SNV	RegulatoryFeature	REGULATORY	0.54	0.15	0.054	0.000	0.000	0.581	0.040	-0.092	4.96	-0.685	.	.	.	.	.	.	.	0.93	10.27	0.58346	ALL	.	.	.	.	.	.	.	.	.	428bp 5' of SKAP2	.
rs73069541	7	26,904,928	A	A	G	SNV	RegulatoryFeature	REGULATORY	0.36	0.01	0.167	0.000	0.001	-0.267	-0.803	-0.702	4.47	-4.49	.	.	.	.	.	.	.	0.49	7.32	0.25792	ABC	.	.	.	.	.	.	.	.	.	586bp 5' of SKAP2	.
rs12540388	7	26,905,731	A	A	T	SNV	Intergenic	UPSTREAM	0.40	0.07	0.004	0.004	0.004	0.000	-0.000	0.000	0	0	.	.	SKAP2	.	.	.	.	-0.67	0.08	0.06388	ADB	.	.	.	.	.	.	.	.	.	1.4kb 5' of SKAP2	.
rs2391362	7	26,907,123	T	T	C	SNV	Intergenic	UPSTREAM	0.39	0.00	0.002	0.002	0.003	-0.697	-0.619	-0.523	0.489	-0.979	.	.	SKAP2	.	.	.	.	-0.55	0.17	0.04944	A	.	.	.	.	.	.	.	.	.	2.8kb 5' of SKAP2	.
rs76438067	7	26,911,904	C	C	T	SNV	Transcript	INTRONIC	0.30	0.03	0.002	0.000	0.000	-2.281	-1.018	-1.006	1.14	-2.29	.	.	SKAP2	2/6	.	.	.	-0.63	0.10	0.06265	AB	.	.	.	.	.	.	.	.	.	7.6kb 5' of SKAP2	.

SNP: SNP rs ID; **CHR:** Chromosome; **BP:** position (NCBI build 37/hg19 coordinates) of SNP marker; **Ref:** reference allele; **Anc**: ancestral allele; **Alt:** observed allele; **Type**: event type ("SNV": single nucleotide variant, 'DEL': deletion, 'INS': insertion); **AnnoType**: 'CodingTranscript', 'Intergenic', 'MotifFeature', 'NonCodingTranscript', 'RegulatoryFeature', 'Transcript'; **Consequence**: '3PRIME_UTR', '5PRIME_UTR', 'DOWNSTREAM', 'INTERGENIC', 'INTRONIC', 'NON_SYNONYMOUS', 'SYNONYMOUS', 'REGULATORY', 'STOP_GAINED', 'STOP_LOST', 'SPLICE_SITE', 'CANONCAL_SPLICE', 'UPSTREAM', 'NONCODING_CHANGE'; **GC**: percent GC in a window of +/- 75 bp; **CpG**: percent CpG in a window of +/-75 bp; **prPhCons**: primate PhastCons conservation score (excl. human); **mamPhCons**: mammalian PhastCons conservation score (excl. human); **verPhCons**: vertebrate PhastCons conservation score (excl. human); **priPhyloP**: primate PhyloP score (excl. human); **mamPhyloP**: mammalian PhyloP score (excl. human); **verPhyloP**: vertebrate PhyloP score (excl. human); **GerpN**: neutral evolution score defined by GERP++ (GERP: Genomic Evolutionary Rate Profiling); **GerpS**: rejected substitution score defined by GERP++; **GerpRS**: Gerp element score; **GerpRSpval**: Gerp element *P*-value ; **GeneName**: name of gene provided in ENSEMBL annotation; **Intron**: intron number/total number of introns; **Grantham**: Grantham score; **PolyPhenVal**: PolyPhen score; **SIFTval**: SIFT score; **RawScore**: raw CADD score. Raw values have relative meaning, with higher values indicating that a variant is more likely not to be observed and therefore more likely to have deleterious effects; **PHRED**: PHRED-like (-10×log10(rank/total)) scaled C-score. A scaled C-score of ≥10 indicates a prediction of the 10% most deleterious substitutions that you can do to the human genome, a score of ≥20 indicates the 1% most deleterious; **Non-Coding score**: predictions are given as *P*-values [0-1], values >0.5 are predicted to be deleterious, values <0.5 are predicted to be neutral/benign; **Non-Coding Groups**: non-coding predictor (for positions in intergenic regions, introns or non-coding genes) using a subset of 4 of these feature groups: A (46-Way Sequence Conservation based on multiple sequence alignment scores, at the nucleotide level, of 46 vertebrate genomes compared to the human genome), B (Histone Modifications based on ChIP-Seq peak calls for histone modifications), C (Transcription Factor Binding Sites (TFBS PeakSeq) based on PeakSeq peak calls for various transcription factors), D (Open Chromatin based on DNase-Seq peak calls); **pred_index**: 'disease causing': probably deleterious, 'disease causing automatic': known to be deleterious, 'polymorphism': probably harmless, 'polymorphism automatic': known to be harmless; **probability**: probability of the prediction, i.e. a value close to 1 indicates a high security of the prediction.; **features**: summary of

affected features; **splice site - effect**: donor/acceptor splice site increased/decreased/ gained/lost; **splice site - gDNA position**: gDNA position of splice site; **splice site - score**: confidence score for wild-type (wt) and/or mutated (mu) splice site as generated by NNSplice[5]; **Sequence constraint by GERP**: 'yes' indicates that the region is called as constrained (vs. non-constrained) by the mammalian conservation algorithm GERP; **Sequence constraint by SiPhy**: 'yes' indicates that the region is called as constrained (vs. non-constrained) by the mammalian conservation algorithm SiPhy-omega; **NHGRI/EBI GWAS hits**: GWAS results (PMID, phenotype, *P*-value) obtained from the NHGRI catalog (accessed October 31, 2015); **RefSeq genes**: RefSeq genes from the UCSC Genome Browser; **dbSNP func annot**: annotations of functional consequences from dbSNP.

Supplementary Table 6. List of known eQTLs for the three newly identified disease associations and all variants in high LD (r^2>0.8) with these variants. All variants were annotated using HaploReg[4] v4·1 to identify SNPs with an effect on gene expression (see **Methods**).

			HaploReg		
SNP	CHR	BP	eQTL resource id*	Selected eQTL hits	P-val
rs17278665	2	56,095,994	GTEx2015_v6	Skin Sun Exposed Lower leg, EFEMP1	2.25×10-6
			Hao2012	Lung, EFEMP1	3.45×10-7
rs11899888	2	56,102,744	.	.	.
rs13064845	3	19,312,697	.	.	.
rs727139	3	19,334,695	.	.	.
rs212838	7	26,698,194	GTEx2015_v6	Thyroid, HOXA5	2.26×10-5
			GTEx2015_v6	Whole Blood, SKAP2	4.26×10-7
			Lappalainen2013	Lymphoblastoid EUR exonlevel, ENSG00000005020.8	8.82×10-7
			Westra2013	Whole Blood, SKAP2	2.23×10-122
rs3757654	7	26,711,104	GTEx2015_v6	Thyroid, HOXA5	1.20×10-5
			GTEx2015_v6	Whole Blood, SKAP2	3.47×10-7
			Lappalainen2013	Lymphoblastoid EUR exonlevel, ENSG00000005020.8	3.45×10-6
			Westra2013	Whole Blood, SKAP2	4.57×10-119
rs212848	7	26,722,155	GTEx2015_v6	Thyroid, HOXA5	1.28×10-5
			GTEx2015_v6	Whole Blood, SKAP2	3.41×10-7
			Lappalainen2013	Lymphoblastoid EUR exonlevel, ENSG00000005020.8	1.44×10-6
rs212850	7	26,724,279	GTEx2015_v6	Thyroid, HOXA5	1.38×10-5
			GTEx2015_v6	Whole Blood, SKAP2	3.80×10-7
			Lappalainen2013	Lymphoblastoid EUR exonlevel, ENSG00000005020.8	1.37×10-6
			Westra2013	Whole Blood, SKAP2	9.41×10-123
rs212856	7	26,729,205	GTEx2015_v6	Thyroid, HOXA5	1.41×10-5
			GTEx2015_v6	Whole Blood, SKAP2	3.89×10-7
			Lappalainen2013	Lymphoblastoid EUR exonlevel, ENSG00000005020.8	1.34×10-6
rs212857	7	26,730,200	GTEx2015_v6	Thyroid, HOXA5	1.23×10-5
			GTEx2015_v6	Whole Blood, SKAP2	7.76×10-7
			Lappalainen2013	Lymphoblastoid EUR exonlevel, ENSG00000005020.8	1.38×10-6
			Westra2013	Whole Blood, SKAP2	2.28×10-122
rs4719877	7	26,736,220	GTEx2015_v6	Thyroid, HOXA5	1.31×10-5
			GTEx2015_v6	Whole Blood, SKAP2	3.44×10-7
			Lappalainen2013	Lymphoblastoid EUR exonlevel, ENSG00000005020.8	1.16×10-6
			Westra2013	Whole Blood, SKAP2	1.87×10-122
rs6461962	7	26,746,304	GTEx2015_v6	Thyroid, HOXA5	1.41×10-5
			GTEx2015_v6	Whole Blood, SKAP2	6.87×10-7
			Lappalainen2013	Lymphoblastoid EUR exonlevel, ENSG00000005020.8	1.89×10-6
			Westra2013	Whole Blood, SKAP2	6.70×10-123
rs6972378	7	26,753,074	GTEx2015_v6	Thyroid, HOXA5	1.38×10-5
			GTEx2015_v6	Whole Blood, SKAP2	5.77×10-7
			Lappalainen2013	Lymphoblastoid EUR exonlevel, ENSG00000005020.8	1.75×10-6
			Westra2013	Whole Blood, SKAP2	2.32×10-123
rs6951065	7	26,756,162	GTEx2015_v6	Thyroid, HOXA5	1.27×10-5
			GTEx2015_v6	Whole Blood, SKAP2	3.51×10-7
			Lappalainen2013	Lymphoblastoid EUR exonlevel, ENSG00000005020.8	1.80×10-6
			Westra2013	Whole Blood, SKAP2	6.91×10-124
rs6964817	7	26,756,424	GTEx2015_v6	Thyroid, HOXA5	1.27×10-5
			GTEx2015_v6	Whole Blood, SKAP2	3.49×10-7
			Lappalainen2013	Lymphoblastoid EUR exonlevel, ENSG00000005020.8	1.67×10-6
			Westra2013	Whole Blood, SKAP2	6.91×10-124
rs2106905	7	26,766,851	GTEx2015_v6	Thyroid, HOXA5	1.22×10-5
			GTEx2015_v6	Whole Blood, SKAP2	2.44×10-7
			Lappalainen2013	Lymphoblastoid EUR exonlevel, ENSG00000005020.8	1.41×10-6
			Westra2013	Whole Blood, SKAP2	1.82×10-120
rs6461963	7	26,772,491	GTEx2015_v6	Thyroid, HOXA5	1.22×10-5
			GTEx2015_v6	Whole Blood, SKAP2	2.43×10-7
			Lappalainen2013	Lymphoblastoid EUR exonlevel, ENSG00000005020.8	1.77×10-6
			Westra2013	Whole Blood, SKAP2	2.30×10-126

rs4722630	7	26,773,364	GTEx2015_v6	Thyroid, HOXA5	1.22×10-5
			GTEx2015_v6	Whole Blood, SKAP2	2.43×10-7
			Lappalainen2013	Lymphoblastoid EUR exonlevel, ENSG00000005020.8	2.19×10-6
			Westra2013	Whole Blood, SKAP2	3.60×10-126
rs6971397	7	26,782,564	GTEx2015_v6	Thyroid, HOXA5	1.18×10-5
			GTEx2015_v6	Whole Blood, SKAP2	1.41×10-7
			Lappalainen2013	Lymphoblastoid EUR exonlevel, ENSG00000005020.8	1.01×10-6
			Westra2013	Whole Blood, SKAP2	3.60×10-126
rs727450	7	26,787,642	GTEx2015_v6	Thyroid, HOXA5	9.08×10-6
			GTEx2015_v6	Whole Blood, SKAP2	3.25×10-7
			Lappalainen2013	Lymphoblastoid EUR exonlevel, ENSG00000005020.8	1.50×10-6
			Westra2013	Whole Blood, SKAP2	5.16×10-126
rs10281261	7	26,789,472	GTEx2015_v6	Thyroid, HOXA5	1.11×10-5
			GTEx2015_v6	Whole Blood, SKAP2	1.37×10-7
			Lappalainen2013	Lymphoblastoid EUR exonlevel, ENSG00000005020.8	1.45×10-6
			Westra2013	Whole Blood, SKAP2	5.16×10-126
rs7457076	7	26,793,215	GTEx2015_v6	Thyroid, HOXA5	9.39×10-6
			GTEx2015_v6	Whole Blood, SKAP2	1.27×10-7
			Lappalainen2013	Lymphoblastoid EUR exonlevel, ENSG00000005020.8	1.32×10-6
			Westra2013	Whole Blood, SKAP2	6.55×10-126
rs2189017	7	26,795,073	GTEx2015_v6	Thyroid, HOXA5	8.16×10-6
			GTEx2015_v6	Whole Blood, SKAP2	1.23×10-7
			Lappalainen2013	Lymphoblastoid EUR exonlevel, ENSG00000005020.8	1.37×10-6
			Westra2013	Whole Blood, SKAP2	6.07×10-126
rs2189019	7	26,798,581	GTEx2015_v6	Thyroid, HOXA5	7.90×10-6
			GTEx2015_v6	Whole Blood, SKAP2	1.23×10-7
			Lappalainen2013	Lymphoblastoid EUR exonlevel, ENSG00000005020.8	1.60×10-6
			Westra2013	Whole Blood, SKAP2	6.07×10-126
rs73061894	7	26,802,085	GTEx2015_v6	Thyroid, HOXA5	7.48×10-6
			GTEx2015_v6	Whole Blood, SKAP2	1.21×10-7
			Lappalainen2013	Lymphoblastoid EUR exonlevel, ENSG00000005020.8	1.04×10-6
rs117217399	7	26,805,212	Lappalainen2013	Lymphoblastoid EUR exonlevel, ENSG00000005020.8	1.34×10-6
rs10486478	7	26,819,465	GTEx2015_v6	Thyroid, HOXA5	1.62×10-6
			GTEx2015_v6	Whole Blood, SKAP2	3.69×10-7
			Lappalainen2013	Lymphoblastoid EUR exonlevel, ENSG00000005020.8	1.61×10-7
			Westra2013	Whole Blood, SKAP2	3.98×10-149
rs3801850	7	26,820,577	GTEx2015_v6	Thyroid, HOXA5	3.64×10-7
			GTEx2015_v6	Whole Blood, SKAP2	6.89×10-6
			Lappalainen2013	Lymphoblastoid EUR exonlevel, ENSG00000005020.8	2.93×10-8
			Westra2013	Whole Blood, SKAP2	2.36×10-149
rs12536560	7	26,822,876	GTEx2015_v6	Thyroid, HOXA5	3.64×10-7
			GTEx2015_v6	Whole Blood, SKAP2	7.14×10-6
			Lappalainen2013	Lymphoblastoid EUR exonlevel, ENSG00000005020.8	3.78×10-8
			Westra2013	Whole Blood, SKAP2	1.91×10-149
rs73065504	7	26,825,984	GTEx2015_v6	Thyroid, HOXA5	9.16×10-7
			GTEx2015_v6	Whole Blood, SKAP2	1.19×10-7
			Lappalainen2013	Lymphoblastoid EUR exonlevel, ENSG00000005020.8	3.73×10-8
rs10267950	7	26,826,757	GTEx2015_v6	Thyroid, HOXA5	3.64×10-7
			GTEx2015_v6	Whole Blood, SKAP2	1.04×10-5
			Lappalainen2013	Lymphoblastoid EUR exonlevel, ENSG00000005020.8	3.48×10-8
			Westra2013	Whole Blood, SKAP2	1.51×10-149
rs10272303	7	26,827,815	GTEx2015_v6	Thyroid, HOXA5	3.64×10-7
			GTEx2015_v6	Whole Blood, SKAP2	1.04×10-5
			Lappalainen2013	Lymphoblastoid EUR exonlevel, ENSG00000005020.8	3.10×10-8
rs3801846	7	26,828,653	GTEx2015_v6	Thyroid, HOXA5	3.92×10-7
			Lappalainen2013	Lymphoblastoid EUR exonlevel, ENSG00000005020.8	3.44×10-8
			Westra2013	Whole Blood, SKAP2	1.11×10-149

rs1327988	7	26,828,965	GTEx2015_v6	Thyroid, HOXA5	3.64×10-7
			Lappalainen2013	Lymphoblastoid EUR exonlevel, ENSG00000005020.8	3.18×10-8
			Westra2013	Whole Blood, SKAP2	8.95×10-150
rs3801844	7	26,829,759	GTEx2015_v6	Thyroid, HOXA5	3.64×10-7
			Lappalainen2013	Lymphoblastoid EUR exonlevel, ENSG00000005020.8	3.16×10-8
			Westra2013	Whole Blood, SKAP2	8.95×10-150
rs3801843	7	26,830,201	GTEx2015_v6	Thyroid, HOXA5	3.64×10-7
			Lappalainen2013	Lymphoblastoid EUR exonlevel, ENSG00000005020.8	3.34×10-8
			Westra2013	Whole Blood, SKAP2	8.95×10-150
rs3801842	7	26,830,263	GTEx2015_v6	Thyroid, HOXA5	3.64×10-7
			Lappalainen2013	Lymphoblastoid EUR exonlevel, ENSG00000005020.8	3.07×10-8
			Westra2013	Whole Blood, SKAP2	8.95×10-150
rs73065517	7	26,830,555	GTEx2015_v6	Thyroid, HOXA5	3.64×10-7
			Lappalainen2013	Lymphoblastoid EUR exonlevel, ENSG00000005020.8	4.16×10-8
rs28733208	7	26,830,825	GTEx2015_v6	Thyroid, HOXA5	3.64×10-7
			Lappalainen2013	Lymphoblastoid EUR exonlevel, ENSG00000005020.8	3.98×10-8
rs73065521	7	26,831,409	GTEx2015_v6	Thyroid, HOXA5	3.64×10-7
			Lappalainen2013	Lymphoblastoid EUR exonlevel, ENSG00000005020.8	3.14×10-8
rs3801840	7	26,832,986	GTEx2015_v6	Thyroid, HOXA5	3.64×10-7
			GTEx2015_v6	Whole Blood, SKAP2	1.04×10-5
			Lappalainen2013	Lymphoblastoid EUR exonlevel, ENSG00000005020.8	3.76×10-8
			Westra2013	Whole Blood, SKAP2	1.41×10-150
rs1327989	7	26,833,563	GTEx2015_v6	Thyroid, HOXA5	3.52×10-7
			Lappalainen2013	Lymphoblastoid EUR exonlevel, ENSG00000005020.8	3.33×10-8
			Westra2013	Whole Blood, SKAP2	1.14×10-150
rs6964094	7	26,834,380	GTEx2015_v6	Thyroid, HOXA5	4.10×10-7
			GTEx2015_v6	Whole Blood, SKAP2	1.17×10-6
			Lappalainen2013	Lymphoblastoid EUR exonlevel, ENSG00000005020.8	3.18×10-8
			Westra2013	Whole Blood, SKAP2	5.02×10-147
rs6956088	7	26,835,727	GTEx2015_v6	Thyroid, HOXA5	3.07×10-7
			Lappalainen2013	Lymphoblastoid EUR exonlevel, ENSG00000005020.8	4.27×10-8
rs10276471	7	26,837,940	GTEx2015_v6	Thyroid, HOXA5	2.56×10-7
			Lappalainen2013	Lymphoblastoid EUR exonlevel, ENSG00000005020.8	1.58×10-8
rs4722641	7	26,838,677	GTEx2015_v6	Thyroid, HOXA5	1.32×10-6
			GTEx2015_v6	Whole Blood, SKAP2	7.60×10-6
			Lappalainen2013	Lymphoblastoid EUR exonlevel, ENSG00000005020.8	1.35×10-8
rs10225924	7	26,839,954	GTEx2015_v6	Thyroid, HOXA5	2.19×10-7
			GTEx2015_v6	Whole Blood, SKAP2	1.03×10-5
			Lappalainen2013	Lymphoblastoid EUR exonlevel, ENSG00000005020.8	1.50×10-8
rs10229837	7	26,840,652	GTEx2015_v6	Thyroid, HOXA5	5.88×10-7
			GTEx2015_v6	Whole Blood, SKAP2	9.85×10-6
			Lappalainen2013	Lymphoblastoid EUR exonlevel, ENSG00000005020.8	1.39×10-8
rs12532395	7	26,841,702	GTEx2015_v6	Thyroid, HOXA5	1.92×10-7
			GTEx2015_v6	Whole Blood, SKAP2	1.03×10-5
			Lappalainen2013	Lymphoblastoid EUR exonlevel, ENSG00000005020.8	1.32×10-8
rs12539719	7	26,844,852	GTEx2015_v6	Thyroid, HOXA5	3.77×10-7
			GTEx2015_v6	Whole Blood, SKAP2	9.70×10-8
			Lappalainen2013	Lymphoblastoid EUR exonlevel, ENSG00000005020.8	4.02×10-8
rs4722644	7	26,845,386	GTEx2015_v6	Thyroid, HOXA5, 4.66927614117146e-07	7.47×10-6
			GTEx2015_v6	Whole Blood, SKAP2	7.47×10-6
			Lappalainen2013	Lymphoblastoid EUR exonlevel, ENSG00000005020.8	1.56×10-8
rs12333525	7	26,847,762	GTEx2015_v6	Thyroid, HOXA5	4.67×10-7
			Lappalainen2013	Lymphoblastoid EUR exonlevel, ENSG00000005020.8	1.07×10-8
rs10244986	7	26,849,558	GTEx2015_v6	Thyroid, HOXA5	4.67×10-7
			GTEx2015_v6	Whole Blood, SKAP2	7.58×10-6
			Lappalainen2013	Lymphoblastoid EUR exonlevel, ENSG00000005020.8	1.49×10-8

rs73067435	7	26,850,195	GTEx2015_v6	Thyroid, HOXA5	4.67×10-7
			GTEx2015_v6	Whole Blood, SKAP2	7.45×10-6
			Lappalainen2013	Lymphoblastoid EUR exonlevel, ENSG00000005020.8	1.49×10-8
rs73067437	7	26,850,313	GTEx2015_v6	Thyroid, HOXA5	3.26×10-7
			GTEx2015_v6	Whole Blood, SKAP2	1.18×10-7
			Lappalainen2013	Lymphoblastoid EUR exonlevel, ENSG00000005020.8	1.50×10-8
rs57135974	7	26,850,699	GTEx2015_v6	Thyroid, HOXA5	4.67×10-7
			GTEx2015_v6	Whole Blood, SKAP2	7.35×10-6
			Lappalainen2013	Lymphoblastoid EUR exonlevel, ENSG00000005020.8	1.46×10-8
rs58760954	7	26,850,776	GTEx2015_v6	Thyroid, HOXA5	4.67×10-7
			GTEx2015_v6	Whole Blood, SKAP2	7.33×10-6
			Lappalainen2013	Lymphoblastoid EUR exonlevel, ENSG00000005020.8	1.26×10-8
rs73067443	7	26,851,074	GTEx2015_v6	Thyroid, HOXA5	4.67×10-7
			GTEx2015_v6	Whole Blood, SKAP2	7.27×10-6
			Lappalainen2013	Lymphoblastoid EUR exonlevel, ENSG00000005020.8	1.47×10-8
rs3801837	7	26,851,361	GTEx2015_v6	Thyroid, HOXA5	4.67×10-7
			GTEx2015_v6	Whole Blood, SKAP2	7.23×10-6
			Lappalainen2013	Lymphoblastoid EUR exonlevel, ENSG00000005020.8	1.21×10-8
rs3801836	7	26,851,400	GTEx2015_v6	Thyroid, HOXA5	5.37×10-7
			GTEx2015_v6	Whole Blood, SKAP2	3.01×10-6
			Lappalainen2013	Lymphoblastoid EUR exonlevel, ENSG00000005020.8	1.27×10-8
rs3801834	7	26,852,062	GTEx2015_v6	Thyroid, HOXA5	4.77×10-7
			Lappalainen2013	Lymphoblastoid EUR exonlevel, ENSG00000005020.8	1.22×10-8
rs3801833	7	26,852,212	GTEx2015_v6	Thyroid, HOXA5	4.67×10-7
			GTEx2015_v6	Whole Blood, SKAP2	7.17×10-6
			Lappalainen2013	Lymphoblastoid EUR exonlevel, ENSG00000005020.8	1.58×10-8
rs3801832	7	26,852,280	GTEx2015_v6	Thyroid, HOXA5	4.67×10-7
			GTEx2015_v6	Whole Blood, SKAP2	7.14×10-6
			Lappalainen2013	Lymphoblastoid EUR exonlevel, ENSG00000005020.8	1.46×10-8
rs7802715	7	26,852,720	GTEx2015_v6	Thyroid, HOXA5	5.52×10-6
			GTEx2015_v6	Whole Blood, SKAP2	5.16×10-6
			Lappalainen2013	Lymphoblastoid EUR exonlevel, ENSG00000005020.8	1.00×10-8
rs7785815	7	26,852,728	GTEx2015_v6	Thyroid, HOXA5	5.52×10-6
			Lappalainen2013	Lymphoblastoid EUR exonlevel, ENSG00000005020.8	1.17×10-8
rs114978183	7	26,852,740	.	.	.
rs115453073	7	26,852,741	GTEx2015_v6	Thyroid, HOXA5	5.45×10-6
			GTEx2015_v6	Whole Blood, SKAP2	6.61×10-6
			Lappalainen2013	Lymphoblastoid EUR exonlevel, ENSG00000005020.8	1.85×10-8
rs7785832	7	26,852,760	GTEx2015_v6	Thyroid, HOXA5	4.69×10-7
			GTEx2015_v6	Whole Blood, SKAP2	6.48×10-6
			Lappalainen2013	Lymphoblastoid EUR exonlevel, ENSG00000005020.8	1.95×10-8
			Westra2013	Whole Blood, SKAP2	1.08×10-153
rs17315334	7	26,852,860	GTEx2015_v6	Thyroid, HOXA5	4.69×10-7
			GTEx2015_v6	Whole Blood, SKAP2	6.47×10-6
			Lappalainen2013	Lymphoblastoid EUR exonlevel, ENSG00000005020.8	2.02×10-8
rs73067456	7	26,852,998	GTEx2015_v6	Thyroid, HOXA5	4.67×10-7
			GTEx2015_v6	Whole Blood, SKAP2	7.00×10-6
			Lappalainen2013	Lymphoblastoid EUR exonlevel, ENSG00000005020.8	1.48×10-8
rs3801831	7	26,853,470	GTEx2015_v6	Thyroid, HOXA5	4.67×10-7
			GTEx2015_v6	Whole Blood, SKAP2	6.98×10-6
			Lappalainen2013	Lymphoblastoid EUR exonlevel, ENSG00000005020.8	4.27×10-8
			Westra2013	Whole Blood, SKAP2	1.48×10-153
rs3823938	7	26,853,741	GTEx2015_v6	Thyroid, HOXA5	4.67×10-7
			GTEx2015_v6	Whole Blood, SKAP2	6.97×10-6
			Lappalainen2013	Lymphoblastoid EUR exonlevel, ENSG00000005020.8	7.89×10-8

rs3823936	7	26,853,830	GTEx2015_v6	Thyroid, HOXA5	4.67×10-7
			GTEx2015_v6	Whole Blood, SKAP2	6.97×10-6
			Lappalainen2013	Lymphoblastoid EUR exonlevel, ENSG00000005020.8	6.67×10-8
			Westra2013	Whole Blood, SKAP2	1.48×10-153
rs3823934	7	26,853,934	GTEx2015_v6	Thyroid, HOXA5	3.07×10-7
			GTEx2015_v6	Whole Blood, SKAP2	1.18×10-7
			Lappalainen2013	Lymphoblastoid EUR exonlevel, ENSG00000005020.8	7.81×10-8
			Westra2013	Whole Blood, SKAP2	1.19×10-153
rs3823933	7	26,853,966	GTEx2015_v6	Thyroid, HOXA5	4.67×10-7
			GTEx2015_v6	Whole Blood, SKAP2	6.97×10-6
			Lappalainen2013	Lymphoblastoid EUR exonlevel, ENSG00000005020.8	5.93×10-8
			Westra2013	Whole Blood, SKAP2	1.19×10-153
rs3823932	7	26,853,980	GTEx2015_v6	Thyroid, HOXA5	4.67×10-7
			GTEx2015_v6	Whole Blood, SKAP2	6.97×10-6
			Lappalainen2013	Lymphoblastoid EUR exonlevel, ENSG00000005020.8	7.33×10-8
			Westra2013	Whole Blood, SKAP2	1.19×10-153
rs3823931	7	26,854,026	GTEx2015_v6	Thyroid, HOXA5	4.67×10-7
			GTEx2015_v6	Whole Blood, SKAP2	6.97×10-6
			Lappalainen2013	Lymphoblastoid EUR exonlevel, ENSG00000005020.8	6.35×10-8
rs10262414	7	26,854,266	GTEx2015_v6	Thyroid, HOXA5	4.67×10-7
			GTEx2015_v6	Whole Blood, SKAP2	2.65×10-6
			Lappalainen2013	Lymphoblastoid EUR exonlevel, ENSG00000005020.8	6.76×10-8
			Westra2013	Whole Blood, SKAP2	8.07×10-154
rs12532199	7	26,854,375	GTEx2015_v6	Thyroid, HOXA5	4.67×10-7
			GTEx2015_v6	Whole Blood, SKAP2	6.97×10-6
			Lappalainen2013	Lymphoblastoid EUR exonlevel, ENSG00000005020.8	5.98×10-8
rs12537323	7	26,854,388	GTEx2015_v6	Thyroid, HOXA5	4.68×10-7
			GTEx2015_v6	Whole Blood, SKAP2	6.96×10-6
			Lappalainen2013	Lymphoblastoid EUR exonlevel, ENSG00000005020.8	5.99×10-8
			Westra2013	Whole Blood, SKAP2	1.08×10-153
rs28617235	7	26,854,671	GTEx2015_v6	Thyroid, HOXA5	4.67×10-7
			GTEx2015_v6	Whole Blood, SKAP2	6.97×10-6
			Lappalainen2013	Lymphoblastoid EUR exonlevel, ENSG00000005020.8	6.40×10-8
rs12532796	7	26,854,828	GTEx2015_v6	Thyroid, HOXA5	3.07×10-7
			GTEx2015_v6	Whole Blood, SKAP2	1.18×10-7
			Lappalainen2013	Lymphoblastoid EUR exonlevel, ENSG00000005020.8	6.76×10-8
			Westra2013	Whole Blood, SKAP2	1.08×10-153
rs3801830	7	26,854,870	GTEx2015_v6	Thyroid, HOXA5	4.74×10-7
			GTEx2015_v6	Whole Blood, SKAP2	6.98×10-6
			Lappalainen2013	Lymphoblastoid EUR exonlevel, ENSG00000005020.8	6.15×10-8
			Westra2013	Whole Blood, SKAP2	4.83×10-150
rs3801829	7	26,854,996	GTEx2015_v6	Thyroid, HOXA5	4.67×10-7
			GTEx2015_v6	Whole Blood, SKAP2	6.97×10-6
			Lappalainen2013	Lymphoblastoid EUR exonlevel, ENSG00000005020.8	6.01×10-8
rs10266759	7	26,855,282	GTEx2015_v6	Thyroid, HOXA5	4.67×10-7
			GTEx2015_v6	Whole Blood, SKAP2	6.97×10-6
			Lappalainen2013	Lymphoblastoid EUR exonlevel, ENSG00000005020.8	4.99×10-8
rs3801828	7	26,855,284	GTEx2015_v6	Thyroid, HOXA5	4.67×10-7
			GTEx2015_v6	Whole Blood, SKAP2	6.97×10-6
			Lappalainen2013	Lymphoblastoid EUR exonlevel, ENSG00000005020.8	6.41×10-8
			Westra2013	Whole Blood, SKAP2	1.08×10-153
rs6948584	7	26,855,732	GTEx2015_v6	Thyroid, HOXA5	4.36×10-7
			GTEx2015_v6	Whole Blood, SKAP2	7.38×10-6
			Lappalainen2013	Lymphoblastoid EUR exonlevel, ENSG00000005020.8	1.55×10-8

rs6948657	7	26,855,943	GTEx2015_v6	Thyroid, HOXA5	4.43×10-7
			GTEx2015_v6	Whole Blood, SKAP2	6.72×10-6
			Lappalainen2013	Lymphoblastoid EUR exonlevel, ENSG00000005020.8	1.52×10-8
			Westra2013	Whole Blood, SKAP2	1.08×10-153
rs73067472	7	26,855,959	GTEx2015_v6	Thyroid, HOXA5	2.76×10-7
			GTEx2015_v6	Whole Blood, SKAP2	1.14×10-7
			Lappalainen2013	Lymphoblastoid EUR exonlevel, ENSG00000005020.8	1.46×10-8
rs3823930	7	26,856,525	GTEx2015_v6	Thyroid, HOXA5	4.43×10-7
			GTEx2015_v6	Whole Blood, SKAP2	6.72×10-6
			Lappalainen2013	Lymphoblastoid EUR exonlevel, ENSG00000005020.8	1.29×10-8
rs3823929	7	26,856,600	GTEx2015_v6	Thyroid, HOXA5	3.85×10-7
			GTEx2015_v6	Whole Blood, SKAP2	3.84×10-6
			Lappalainen2013	Lymphoblastoid EUR exonlevel, ENSG00000005020.8	1.51×10-8
rs3801827	7	26,856,626	GTEx2015_v6	Thyroid, HOXA5	4.43×10-7
			GTEx2015_v6	Whole Blood, SKAP2	6.72×10-6
			Lappalainen2013	Lymphoblastoid EUR exonlevel, ENSG00000005020.8	1.61×10-8
rs3801826	7	26,856,720	GTEx2015_v6	Thyroid, HOXA5	4.43×10-7
			GTEx2015_v6	Whole Blood, SKAP2	6.72×10-6
			Lappalainen2013	Lymphoblastoid EUR exonlevel, ENSG00000005020.8	1.49×10-8
rs3801825	7	26,856,959	GTEx2015_v6	Thyroid, HOXA5	4.43×10-7
			GTEx2015_v6	Whole Blood, SKAP2	6.72×10-6
			Lappalainen2013	Lymphoblastoid EUR exonlevel, ENSG00000005020.8	1.35×10-8
			Westra2013	Whole Blood, SKAP2	8.56×10-154
rs73067475	7	26,857,120	GTEx2015_v6	Thyroid, HOXA5	4.43×10-7
			GTEx2015_v6	Whole Blood, SKAP2	6.72×10-6
			Lappalainen2013	Lymphoblastoid EUR exonlevel, ENSG00000005020.8	1.41×10-8
rs73067476	7	26,857,240	GTEx2015_v6	Thyroid, HOXA5	4.43×10-7
			GTEx2015_v6	Whole Blood, SKAP2	6.72×10-6
			Lappalainen2013	Lymphoblastoid EUR exonlevel, ENSG00000005020.8	1.43×10-8
rs115447617	7	26,857,751	Lappalainen2013	Lymphoblastoid EUR exonlevel, ENSG00000005020.8	1.54×10-8
rs111373218	7	26,859,152	GTEx2015_v6	Thyroid, HOXA5	1.03×10-5
			Lappalainen2013	Lymphoblastoid EUR exonlevel, ENSG00000005020.8	2.46×10-9
rs17315775	7	26,860,134	GTEx2015_v6	Thyroid, HOXA5	4.43×10-7
			GTEx2015_v6	Whole Blood, SKAP2	6.71×10-6
			Lappalainen2013	Lymphoblastoid EUR exonlevel, ENSG00000005020.8	1.37×10-8
			Westra2013	Whole Blood, SKAP2	8.56×10-154
rs28404962	7	26,860,324	GTEx2015_v6	Thyroid, HOXA5	4.43×10-7
			GTEx2015_v6	Whole Blood, SKAP2	6.71×10-6
			Lappalainen2013	Lymphoblastoid EUR exonlevel, ENSG00000005020.8	1.51×10-8
rs13438513	7	26,860,633	GTEx2015_v6	Thyroid, HOXA5	4.43×10-7
			GTEx2015_v6	Whole Blood, SKAP2	6.71×10-6
			Lappalainen2013	Lymphoblastoid EUR exonlevel, ENSG00000005020.8	1.84×10-8
			Westra2013	Whole Blood, SKAP2	8.56×10-154
rs13438514	7	26,860,688	GTEx2015_v6	Thyroid, HOXA5	4.45×10-7
			GTEx2015_v6	Whole Blood, SKAP2	6.53×10-6
			Lappalainen2013	Lymphoblastoid EUR exonlevel, ENSG00000005020.8	1.67×10-8
			Westra2013	Whole Blood, SKAP2	8.56×10-154
rs55739485	7	26,860,741	GTEx2015_v6	Thyroid, HOXA5	4.42×10-7
			GTEx2015_v6	Whole Blood, SKAP2	6.72×10-6
			Lappalainen2013	Lymphoblastoid EUR exonlevel, ENSG00000005020.8	1.53×10-8
rs3801823	7	26,861,031	GTEx2015_v6	Thyroid, HOXA5	4.32×10-7
			GTEx2015_v6	Whole Blood, SKAP2	8.96×10-6
			Lappalainen2013	Lymphoblastoid EUR exonlevel, ENSG00000005020.8	1.66×10-8
rs3801822	7	26,861,201	GTEx2015_v6	Thyroid, HOXA5	4.42×10-7
			GTEx2015_v6	Whole Blood, SKAP2	6.72×10-6
			Lappalainen2013	Lymphoblastoid EUR exonlevel, ENSG00000005020.8	2.09×10-8

rs3801821	7	26,861,206	GTEx2015_v6	Thyroid, HOXA5	4.42×10-7
			GTEx2015_v6	Whole Blood, SKAP2	6.72×10-6
			Lappalainen2013	Lymphoblastoid EUR exonlevel, ENSG00000005020.8	1.98×10-8
			Westra2013	Whole Blood, SKAP2	8.56×10-154
rs3801820	7	26,861,353	GTEx2015_v6	Thyroid, HOXA5	4.42×10-7
			GTEx2015_v6	Whole Blood, SKAP2	6.72×10-6
			Lappalainen2013	Lymphoblastoid EUR exonlevel, ENSG00000005020.8	1.51×10-8
			Westra2013	Whole Blood, SKAP2	8.56×10-154
rs7785689	7	26,861,609	GTEx2015_v6	Thyroid, HOXA5	4.42×10-7
			GTEx2015_v6	Whole Blood, SKAP2	6.72×10-6
			Lappalainen2013	Lymphoblastoid EUR exonlevel, ENSG00000005020.8	1.41×10-8
rs7785711	7	26,861,640	GTEx2015_v6	Thyroid, HOXA5	4.29×10-7
			GTEx2015_v6	Whole Blood, SKAP2	7.32×10-6
			Lappalainen2013	Lymphoblastoid EUR exonlevel, ENSG00000005020.8	1.33×10-8
rs7785798	7	26,861,943	GTEx2015_v6	Thyroid, HOXA5	4.42×10-7
			GTEx2015_v6	Whole Blood, SKAP2	6.72×10-6
			Lappalainen2013	Lymphoblastoid EUR exonlevel, ENSG00000005020.8	1.71×10-8
rs7785918	7	26,861,959	GTEx2015_v6	Thyroid, HOXA5	4.35×10-7
			GTEx2015_v6	Whole Blood, SKAP2	6.93×10-6
			Lappalainen2013	Lymphoblastoid EUR exonlevel, ENSG00000005020.8	1.59×10-8
rs12540045	7	26,863,174	GTEx2015_v6	Thyroid, HOXA5	4.43×10-7
			GTEx2015_v6	Whole Blood, SKAP2	6.71×10-6
			Lappalainen2013	Lymphoblastoid EUR exonlevel, ENSG00000005020.8	1.43×10-8
rs10248500	7	26,863,294	GTEx2015_v6	Thyroid, HOXA5	4.43×10-7
			GTEx2015_v6	Whole Blood, SKAP2	6.71×10-6
			Lappalainen2013	Lymphoblastoid EUR exonlevel, ENSG00000005020.8	1.53×10-8
rs17315929	7	26,864,173	GTEx2015_v6	Thyroid, HOXA5	4.43×10-7
			GTEx2015_v6	Whole Blood, SKAP2	6.65×10-6
			Lappalainen2013	Lymphoblastoid EUR exonlevel, ENSG00000005020.8	5.25×10-8
			Westra2013	Whole Blood, SKAP2	5.78×10-154
rs10227673	7	26,865,299	GTEx2015_v6	Thyroid, HOXA5	4.43×10-7
			GTEx2015_v6	Whole Blood, SKAP2	6.71×10-6
			Lappalainen2013	Lymphoblastoid EUR exonlevel, ENSG00000005020.8	1.15×10-8
rs7805766	7	26,865,610	GTEx2015_v6	Thyroid, HOXA5	4.43×10-7
			GTEx2015_v6	Whole Blood, SKAP2	1.11×10-5
			Lappalainen2013	Lymphoblastoid EUR exonlevel, ENSG00000005020.8	1.16×10-8
			Westra2013	Whole Blood, SKAP2	5.37×10-159
rs17375272	7	26,866,497	GTEx2015_v6	Thyroid, HOXA5	2.97×10-7
			Lappalainen2013	Lymphoblastoid EUR exonlevel, ENSG00000005020.8	1.30×10-8
rs3801819	7	26,866,698	GTEx2015_v6	Thyroid, HOXA5	2.76×10-7
			GTEx2015_v6	Whole Blood, SKAP2	1.14×10-7
			Lappalainen2013	Lymphoblastoid EUR exonlevel, ENSG00000005020.8	1.39×10-8
			Westra2013	Whole Blood, SKAP2	1.94×10-154
rs61354389	7	26,868,343	GTEx2015_v6	Thyroid, HOXA5	4.43×10-7
			GTEx2015_v6	Whole Blood, SKAP2	6.71×10-6
			Lappalainen2013	Lymphoblastoid EUR exonlevel, ENSG00000005020.8	1.10×10-8
rs10225700	7	26,869,880	GTEx2015_v6	Thyroid, HOXA5	2.26×10-6
			GTEx2015_v6	Whole Blood, SKAP2	8.05×10-6
			Lappalainen2013	Lymphoblastoid EUR exonlevel, ENSG00000005020.8	3.62×10-8
rs10241234	7	26,869,887	GTEx2015_v6	Thyroid, HOXA5	3.78×10-6
			GTEx2015_v6	Whole Blood, SKAP2	3.39×10-6
			Lappalainen2013	Lymphoblastoid EUR exonlevel, ENSG00000005020.8	4.69×10-8
rs10225904	7	26,869,888	GTEx2015_v6	Thyroid, HOXA5	3.78×10-6
			GTEx2015_v6	Whole Blood, SKAP2	3.39×10-6
			Lappalainen2013	Lymphoblastoid EUR exonlevel, ENSG00000005020.8	4.34×10-8

rs28550029	7	26,872,581	GTEx2015_v6	Thyroid, HOXA5	4.43×10^{-7}
			GTEx2015_v6	Whole Blood, SKAP2	6.71×10^{-6}
			Lappalainen2013	Lymphoblastoid EUR exonlevel, ENSG00000005020.8	1.40×10^{-8}
rs114236648	7	26,874,639	Lappalainen2013	Lymphoblastoid EUR exonlevel, ENSG00000005020.8	2.20×10^{-8}
rs12534899	7	26,877,189	GTEx2015_v6	Thyroid, HOXA5	4.43×10^{-7}
			GTEx2015_v6	Whole Blood, SKAP2	6.71×10^{-6}
			Lappalainen2013	Lymphoblastoid EUR exonlevel, ENSG00000005020.8	1.46×10^{-8}
			Westra2013	Whole Blood, SKAP2	7.96×10^{-155}
rs12534921	7	26,877,210	GTEx2015_v6	Thyroid, HOXA5	4.43×10^{-7}
			GTEx2015_v6	Whole Blood, SKAP2	6.71×10^{-6}
			Lappalainen2013	Lymphoblastoid EUR exonlevel, ENSG00000005020.8	2.10×10^{-8}
rs73069507	7	26,877,536	GTEx2015_v6	Thyroid, HOXA5	4.43×10^{-7}
			GTEx2015_v6	Whole Blood, SKAP2	6.71×10^{-6}
			Lappalainen2013	Lymphoblastoid EUR exonlevel, ENSG00000005020.8	2.30×10^{-8}
rs73069510	7	26,877,567	GTEx2015_v6	Thyroid, HOXA5	3.32×10^{-7}
			Lappalainen2013	Lymphoblastoid EUR exonlevel, ENSG00000005020.8	1.86×10^{-8}
rs3801816	7	26,881,177	GTEx2015_v6	Thyroid, HOXA5	4.57×10^{-7}
			GTEx2015_v6	Whole Blood, SKAP2	6.99×10^{-6}
			Lappalainen2013	Lymphoblastoid EUR exonlevel, ENSG00000005020.8	9.22×10^{-9}
			Westra2013	Whole Blood, SKAP2	5.64×10^{-155}
rs3801815	7	26,881,292	GTEx2015_v6	Thyroid, HOXA5	4.76×10^{-7}
			GTEx2015_v6	Whole Blood, SKAP2	2.22×10^{-6}
			Lappalainen2013	Lymphoblastoid EUR exonlevel, ENSG00000005020.8	1.90×10^{-8}
			Westra2013	Whole Blood, SKAP2	5.64×10^{-155}
rs3801814	7	26,881,302	GTEx2015_v6	Thyroid, HOXA5	4.67×10^{-7}
			GTEx2015_v6	Whole Blood, SKAP2	2.65×10^{-6}
			Lappalainen2013	Lymphoblastoid EUR exonlevel, ENSG00000005020.8	1.74×10^{-8}
			Westra2013	Whole Blood, SKAP2	4.73×10^{-155}
rs12535784	7	26,882,067	GTEx2015_v6	Thyroid, HOXA5	4.83×10^{-7}
			GTEx2015_v6	Whole Blood, SKAP2	6.05×10^{-6}
			Lappalainen2013	Lymphoblastoid EUR exonlevel, ENSG00000005020.8	1.60×10^{-8}
			Westra2013	Whole Blood, SKAP2	7.01×10^{-155}
rs4719882	7	26,882,252	GTEx2015_v6	Thyroid, HOXA5	3.04×10^{-7}
			GTEx2015_v6	Whole Blood, SKAP2	6.58×10^{-6}
			Lappalainen2013	Lymphoblastoid EUR exonlevel, ENSG00000005020.8	8.99×10^{-7}
rs1982995	7	26,884,135	GTEx2015_v6	Thyroid, HOXA5	3.48×10^{-7}
			GTEx2015_v6	Whole Blood, SKAP2	5.50×10^{-6}
			Lappalainen2013	Lymphoblastoid EUR exonlevel, ENSG00000005020.8	1.51×10^{-8}
rs10236221	7	26,884,671	GTEx2015_v6	Thyroid, HOXA5	4.87×10^{-7}
			GTEx2015_v6	Whole Blood, SKAP2	5.81×10^{-6}
			Lappalainen2013	Lymphoblastoid EUR exonlevel, ENSG00000005020.8	1.44×10^{-8}
rs10270187	7	26,885,885	GTEx2015_v6	Thyroid, HOXA5	4.97×10^{-7}
			GTEx2015_v6	Whole Blood, SKAP2	7.15×10^{-6}
			Lappalainen2013	Lymphoblastoid EUR exonlevel, ENSG00000005020.8	1.85×10^{-8}
			Westra2013	Whole Blood, SKAP2	7.01×10^{-155}
rs10227529	7	26,886,309	GTEx2015_v6	Thyroid, HOXA5	5.53×10^{-7}
			GTEx2015_v6	Whole Blood, SKAP2	7.09×10^{-6}
			Lappalainen2013	Lymphoblastoid EUR exonlevel, ENSG00000005020.8	1.79×10^{-7}
rs3801813	7	26,887,069	GTEx2015_v6	Thyroid, HOXA5	4.19×10^{-7}
			GTEx2015_v6	Whole Blood, SKAP2	6.20×10^{-6}
			Lappalainen2013	Lymphoblastoid EUR exonlevel, ENSG00000005020.8	7.77×10^{-9}
			Westra2013	Whole Blood, SKAP2	8.72×10^{-155}
rs3801812	7	26,887,615	GTEx2015_v6	Thyroid, HOXA5	4.97×10^{-7}
			GTEx2015_v6	Whole Blood, SKAP2	7.13×10^{-6}
			Lappalainen2013	Lymphoblastoid EUR exonlevel, ENSG00000005020.8	1.62×10^{-8}
			Westra2013	Whole Blood, SKAP2	1.08×10^{-154}

rs10272190	7	26,887,942	GTEx2015_v6	Thyroid, HOXA5	4.97×10-7
			GTEx2015_v6	Whole Blood, SKAP2	7.12×10-6
			Lappalainen2013	Lymphoblastoid EUR exonlevel, ENSG00000005020.8	1.58×10-8
			Westra2013	Whole Blood, SKAP2	1.08×10-154
rs10227231	7	26,888,074	GTEx2015_v6	Thyroid, HOXA5	9.43×10-7
			GTEx2015_v6	Whole Blood, SKAP2	1.07×10-6
			Lappalainen2013	Lymphoblastoid EUR exonlevel, ENSG00000005020.8	1.26×10-8
			Westra2013	Whole Blood, SKAP2	1.08×10-154
rs10272457	7	26,888,155	GTEx2015_v6	Thyroid, HOXA5	4.97×10-7
			GTEx2015_v6	Whole Blood, SKAP2	7.11×10-6
			Lappalainen2013	Lymphoblastoid EUR exonlevel, ENSG00000005020.8	1.51×10-8
			Westra2013	Whole Blood, SKAP2	1.08×10-154
rs10272603	7	26,888,277	GTEx2015_v6	Thyroid, HOXA5	4.97×10-7
			GTEx2015_v6	Whole Blood, SKAP2	7.11×10-6
			Lappalainen2013	Lymphoblastoid EUR exonlevel, ENSG00000005020.8	1.65×10-8
rs6461979	7	26,888,918	GTEx2015_v6	Thyroid, HOXA5	2.92×10-7
			GTEx2015_v6	Whole Blood, SKAP2	1.51×10-7
			Lappalainen2013	Lymphoblastoid EUR exonlevel, ENSG00000005020.8	1.49×10-8
			Westra2013	Whole Blood, SKAP2	1.35×10-154
rs17376018	7	26,889,862	GTEx2015_v6	Thyroid, HOXA5	3.35×10-7
			GTEx2015_v6	Whole Blood, SKAP2	1.03×10-5
			Lappalainen2013	Lymphoblastoid EUR exonlevel, ENSG00000005020.8	1.41×10-8
rs4722647	7	26,890,446	GTEx2015_v6	Thyroid, HOXA5	4.45×10-7
			GTEx2015_v6	Whole Blood, SKAP2	6.36×10-6
			Lappalainen2013	Lymphoblastoid EUR exonlevel, ENSG00000005020.8	1.10×10-8
rs3934268	7	26,890,572	GTEx2015_v6	Thyroid, HOXA5	4.68×10-7
			GTEx2015_v6	Whole Blood, SKAP2	6.78×10-6
			Lappalainen2013	Lymphoblastoid EUR exonlevel, ENSG00000005020.8	1.37×10-8
rs2031811	7	26,890,672	GTEx2015_v6	Thyroid, HOXA5	4.67×10-7
			GTEx2015_v6	Whole Blood, SKAP2	6.78×10-6
			Lappalainen2013	Lymphoblastoid EUR exonlevel, ENSG00000005020.8	1.31×10-8
			Westra2013	Whole Blood, SKAP2	1.35×10-154
rs2030136	7	26,891,016	GTEx2015_v6	Thyroid, HOXA5	4.69×10-7
			Lappalainen2013	Lymphoblastoid EUR exonlevel, ENSG00000005020.8	8.30×10-9
			Westra2013	Whole Blood, SKAP2	4.99×10-141
rs7804356	7	26,891,665	GTEx2015_v6	Thyroid, HOXA5	4.67×10-7
			GTEx2015_v6	Whole Blood, SKAP2	6.78×10-6
			Lappalainen2013	Lymphoblastoid EUR exonlevel, ENSG00000005020.8	9.69×10-9
			Westra2013	Whole Blood, SKAP2	6.51×10-155
rs7804620	7	26,891,831	GTEx2015_v6	Thyroid, HOXA5	8.76×10-7
			Lappalainen2013	Lymphoblastoid EUR exonlevel, ENSG00000005020.8	1.18×10-8
rs73069533	7	26,892,325	GTEx2015_v6	Thyroid, HOXA5	2.75×10-7
			GTEx2015_v6	Whole Blood, SKAP2	1.43×10-7
			Lappalainen2013	Lymphoblastoid EUR exonlevel, ENSG00000005020.8	1.64×10-8
rs10486483	7	26,892,440	GTEx2015_v6	Thyroid, HOXA5	4.67×10-7
			GTEx2015_v6	Whole Blood, SKAP2	6.75×10-6
			Lappalainen2013	Lymphoblastoid EUR exonlevel, ENSG00000005020.8	1.69×10-8
			Westra2013	Whole Blood, SKAP2	1.35×10-154
rs3801810	7	26,892,531	GTEx2015_v6	Thyroid, HOXA5	4.59×10-7
			GTEx2015_v6	Whole Blood, SKAP2	8.97×10-6
			Lappalainen2013	Lymphoblastoid EUR exonlevel, ENSG00000005020.8	1.69×10-8
			Westra2013	Whole Blood, SKAP2	1.35×10-154
rs3823928	7	26,893,317	GTEx2015_v6	Thyroid, HOXA5	4.68×10-7
			GTEx2015_v6	Whole Blood, SKAP2	6.74×10-6
			Lappalainen2013	Lymphoblastoid EUR exonlevel, ENSG00000005020.8	1.44×10-8

rs7791513	7	26,893,357	GTEx2015_v6	Thyroid, HOXA5	5.40×10-7
			GTEx2015_v6	Whole Blood, SKAP2	6.40×10-6
			Lappalainen2013	Lymphoblastoid EUR exonlevel, ENSG00000005020.8	1.42×10-8
			Westra2013	Whole Blood, SKAP2	1.35×10-154
rs3213853	7	26,893,524	GTEx2015_v6	Thyroid, HOXA5	4.68×10-7
			GTEx2015_v6	Whole Blood, SKAP2	6.74×10-6
			Lappalainen2013	Lymphoblastoid EUR exonlevel, ENSG00000005020.8	1.32×10-8
rs6461980	7	26,894,123	GTEx2015_v6	Thyroid, HOXA5	4.68×10-7
			GTEx2015_v6	Whole Blood, SKAP2	6.70×10-6
			Lappalainen2013	Lymphoblastoid EUR exonlevel, ENSG00000005020.8	1.40×10-8
rs12536145	7	26,894,860	GTEx2015_v6	Thyroid, HOXA5	4.69×10-7
			GTEx2015_v6	Whole Blood, SKAP2	6.22×10-6
			Lappalainen2013	Lymphoblastoid EUR exonlevel, ENSG00000005020.8	1.31×10-8
rs10242572	7	26,895,065	GTEx2015_v6	Thyroid, HOXA5	4.70×10-7
			GTEx2015_v6	Whole Blood, SKAP2	6.83×10-6
			Lappalainen2013	Lymphoblastoid EUR exonlevel, ENSG00000005020.8	2.09×10-8
			Westra2013	Whole Blood, SKAP2	9.34×10-143
rs10256250	7	26,895,080	GTEx2015_v6	Thyroid, HOXA5	4.70×10-7
			GTEx2015_v6	Whole Blood, SKAP2	6.07×10-6
			Lappalainen2013	Lymphoblastoid EUR exonlevel, ENSG00000005020.8	1.53×10-8
			Westra2013	Whole Blood, SKAP2	1.35×10-154
rs3801809	7	26,895,456	GTEx2015_v6	Thyroid, HOXA5	4.73×10-7
			GTEx2015_v6	Whole Blood, SKAP2	5.56×10-6
			Lappalainen2013	Lymphoblastoid EUR exonlevel, ENSG00000005020.8	1.29×10-8
rs3801808	7	26,896,555	GTEx2015_v6	Thyroid, HOXA5	4.92×10-7
			GTEx2015_v6	Whole Blood, SKAP2	4.36×10-6
			Lappalainen2013	Lymphoblastoid EUR exonlevel, ENSG00000005020.8	3.77×10-8
			Westra2013	Whole Blood, SKAP2	2.11×10-150
rs3801807	7	26,897,439	GTEx2015_v6	Thyroid, HOXA5	4.91×10-7
			GTEx2015_v6	Whole Blood, SKAP2	4.34×10-6
			Lappalainen2013	Lymphoblastoid EUR exonlevel, ENSG00000005020.8	1.32×10-8
			Westra2013	Whole Blood, SKAP2	1.16×10-154
rs3801805	7	26,897,640	GTEx2015_v6	Thyroid, HOXA5	1.63×10-6
			GTEx2015_v6	Whole Blood, SKAP2	2.40×10-6
			Lappalainen2013	Lymphoblastoid EUR exonlevel, ENSG00000005020.8	1.81×10-8
			Westra2013	Whole Blood, SKAP2	1.35×10-154
rs10486485	7	26,900,356	GTEx2015_v6	Thyroid, HOXA5	1.05×10-6
			GTEx2015_v6	Whole Blood, SKAP2	5.74×10-7
			Lappalainen2013	Lymphoblastoid EUR exonlevel, ENSG00000005020.8	3.82×10-8
			Westra2013	Whole Blood, SKAP2	7.16×10-155
rs3801803	7	26,901,740	GTEx2015_v6	Thyroid, HOXA5	8.85×10-7
			GTEx2015_v6	Whole Blood, SKAP2	1.41×10-6
			Lappalainen2013	Lymphoblastoid EUR exonlevel, ENSG00000005020.8	2.58×10-8
			Westra2013	Whole Blood, SKAP2	7.16×10-155
rs73069539	7	26,903,661	GTEx2015_v6	Thyroid, HOXA5	4.23×10-7
			GTEx2015_v6	Whole Blood, SKAP2	6.23×10-9
			Lappalainen2013	Lymphoblastoid EUR exonlevel, ENSG00000005020.8	5.06×10-8
rs17323934	7	26,904,330	GTEx2015_v6	Thyroid, HOXA5	7.33×10-7
			GTEx2015_v6	Whole Blood, SKAP2	9.46×10-9
			Lappalainen2013	Lymphoblastoid EUR exonlevel, ENSG00000005020.8	3.67×10-9
			Westra2013	Whole Blood, SKAP2	1.58×10-161
rs73069540	7	26,904,770	GTEx2015_v6	Thyroid, HOXA5	3.72×10-7
			GTEx2015_v6	Whole Blood, SKAP2	5.40×10-9
			Lappalainen2013	Lymphoblastoid EUR exonlevel, ENSG00000005020.8	2.54×10-9
rs73069541	7	26,904,928	GTEx2015_v6	Thyroid, HOXA5	3.71×10-7
			GTEx2015_v6	Whole Blood, SKAP2	5.29×10-9
			Lappalainen2013	Lymphoblastoid EUR exonlevel, ENSG00000005020.8	2.24×10-9

rs12540388	7	26,905,731	GTEx2015_v6	Thyroid, HOXA5	3.60×10-7
			GTEx2015_v6	Whole Blood, SKAP2	4.85×10-9
			Lappalainen2013	Lymphoblastoid EUR exonlevel, ENSG00000005020.8	3.00×10-9
			Westra2013	Whole Blood, SKAP2	3.49×10-163
rs2391362	7	26,907,123	GTEx2015_v6	Thyroid, HOXA5	3.35×10-7
			GTEx2015_v6	Whole Blood, SKAP2	5.01×10-9
			Lappalainen2013	Lymphoblastoid EUR exonlevel, ENSG00000005020.8	4.56×10-9
			Westra2013	Whole Blood, SKAP2	1.64×10-162
rs76438067	7	26,911,904	GTEx2015_v6	Thyroid, HOXA5	4.30×10-7
			GTEx2015_v6	Whole Blood, SKAP2	2.29×10-9
			Lappalainen2013	Lymphoblastoid EUR exonlevel, ENSG00000005020.8	3.77×10-9

*eQTL resources used by HaploReg

id	pmid	name	source
Montgomery2010	20220756	Transcriptome genetics using second generation sequencing in a Caucasian population	From NCBI eQTL Browser, http://www.ncbi.nlm.nih.gov/projects/gap/eqtl/index.cgi
Schadt2008	18462017	Mapping the genetic architecture of gene expression in human liver	From NCBI eQTL Browser, http://www.ncbi.nlm.nih.gov/projects/gap/eqtl/index.cgi
Gibbs2010	20485568	Abundant quantitative trait loci exist for DNA methylation and gene expression in human brain	From NCBI eQTL Browser, http://www.ncbi.nlm.nih.gov/projects/gap/eqtl/index.cgi
Stranger2008	17873874	Population genomics of human gene expression	From NCBI eQTL Browser, http://www.ncbi.nlm.nih.gov/projects/gap/eqtl/index.cgi
GTEx2015_v6	25954001	The Genotype-Tissue Expression (GTEx) pilot analysis: Multitissue gene regulation in humans	From GTEx Portal, Release v6, from http://www.gtexportal.org/static/datasets/gtex_analysis_v6/single_tissue_eqtl_data/GTEx_Analysis_V6_eQTLs.tar.gz
Li2014	24907074	Expression QTL-based analyses reveal candidate causal genes and loci across five tumor types	From Table S1. cis-eQTL for mRNA were exported from each tab of the Excel file.
Westra2013	24013639	Systematic identification of trans eQTLs as putative drivers of known disease associations	All associations from 2012-12-21-CisAssociationsProbeLevelFDR0.5.txt
Hao2012	23209423	Lung eQTLs to help reveal the molecular underpinnings of asthma	All cis and trans eQTLs from Tables S2A and S2B were combined in the provided Excel and the p value in the field "pvalue.fix" was used
Koopmann2014	24846176	Genome-wide identification of expression quantitative trait loci (eQTLs) in human heart	All associations from Table S1
Fairfax2014	24604202	Innate immune activity conditions the effect of regulatory variants upon monocyte gene expression	All associations from Tables S2B and S3B. Tables were flattened by the four treatments and filtered for "NA" reported as a p-value.
Grundberg2011	21283786	Global Analysis of the Impact of Environmental Perturbation on cis-Regulation of Gene Expression	All associations from Table S6
Lappalainen2013	24037378	Transcriptome and genome sequencing uncovers functional variation in humans	All gene- and exon-level eQTLs from EUR and YRI. Gene-leveQTL genes are translated from ENSG to HGNC names when possible; exons are named by probe name.
Ramasamy2014	25174007	Genetic variability in the regulation of gene expression in ten regions of the human brain	All associations reported in Table S2

SNP: SNP rs ID; **CHR:** Chromosome; **BP:** position (NCBI build 37/hg19 coordinates) of SNP marker; **eQTL resource id**: publication that reports SNP as eQTL; **Selected eQTL hits**: selection of eQTL hits, shown are cell types/tissues and the respective gene; **P-val**: *P*-value of eQTL for the respective gene in the respective cell type/tissue.

Supplementary Table 7. Gene expression of genes *EFEMP1*, *SKAP2* and *KCNH8* across multiple tissues/cell lines from ENCODE data. Expression data was obtained from website http://promoter.bx.psu.edu/ENCODE/search_human.php.

tissues/ cell lines	cell	Description	$RPKM_{EFEMP1}$	$RPKM_{SKAP2}$	$RPKM_{KCNH8}$
A549_cell_longNonPolyA	A549	epithelial cell line derived from a lung carcinoma tissue	17.08	2.83	0.00
A549_cell_longPolyA			42.21	17.01	0.00
A549_cytosol_longPolyA			55.50	5.02	0.00
A549_nucleus_longPolyA			53.59	1.38	0.00
AG04450_cell_longNonPolyA	AG04450	fetal lung fibroblast	93.71	4.50	0.00
AG04450_cell_longPolyA			352.58	13.02	0.00
BJ_cell_longNonPolyA	BJ	skin fibroblast	273.33	1.04	0.00
BJ_cell_longPolyA			1124.11	3.90	0.00
CD20_Plus_cell_longNonPolyA	CD20+	B cells	0.00	9.58	4.77
CD20_Plus_cell_longPolyA			0.00	11.93	13.12
GM12878_cell_longNonPolyA	GM12878	B-lymphocyte, lymphoblastoid	1.73	3.24	0.00
GM12878_cell_longPolyA			0.51	18.12	0.04
GM12878_cytosol_longNonPolyA			0.00	1.20	0.00
GM12878_cytosol_longPolyA			0.84	9.24	0.00
GM12878_nucleolus_total			0.13	2.83	0.00
GM12878_nucleus_longNonPolyA			0.70	4.59	0.05
GM12878_nucleus_longPolyA			0.58	2.74	0.05
H1_hESC_cell_longNonPolyA	H1-hESC	embryonic stem cells	20.87	0.32	6.60
H1_hESC_cell_longPolyA			19.29	0.60	3.09
HAoAF_cell_total	HAoAF	Aortic Adventitial Fibroblasts	216.59	7.72	0.00
HAoEC_cell_total	HAoEC	Aortic Endothelial Cells (thoracic)	565.17	11.85	0.00
HAoSMC_cell_total	HAoSMC	Human Aortic Smooth Muscle Cells	694.76	0.79	0.00
HBdMEC_cell_total	HBdMEC	Human Bladder Microvascular Endothelial Cells	4649.97	11.11	0.00
HBdSMC_cell_total	HBdSMC	Human Bladder Smooth Muscle Cells	269.47	3.17	0.00
HBEpC_cell_total	HBEpC	Human Bronchial Epithelial Cells	36.44	0.93	0.00
HBSMC_cell_total	HBSMC	Human Bronchial Smooth Muscle Cells	281.68	3.90	0.00
HCAEC_cell_total	HCAEC	Human Coronary Artery Endothelial Cells	1332.16	5.90	0.00
HCASMC_cell_total	HCASMC	Human Coronary Artery Smooth Muscle Cells	1929.37	0.43	0.00

HCH_cell_total	HCH	Undifferentiated Chondrocytes	1068.59	1.34	0.00
HCM_cell_total	HCM	Human Cardiac Myocytes	939.91	1.01	0.00
HDBEC_cell_total	HDBEC	Human Dermal Blood Endothelial Cells	5896.16	17.95	0.02
HDLEC_cell_total	HDLEC	Human Dermal Lymphatic Endothelial Cells	1031.65	9.53	0.00
HDMEC_cell_total	HDMEC	Human Dermal Microvascular Endothelial Cells	2030.59	7.00	0.00
HeLa_S3_cell_longNonPolyA	HeLa-S3	cervical carcinoma	0.00	1.24	0.00
HeLa_S3_cell_longPolyA			89.60	4.08	0.00
HeLa_S3_cytosol_longPolyA			95.21	2.90	0.00
HeLa_S3_nucleus_longNonPolyA			19.14	1.62	0.00
HeLa_S3_nucleus_longPolyA			60.50	0.51	0.00
HepG2_cell_longNonPolyA	HepG2	hepatocellular carcinoma	0.00	1.38	0.00
HepG2_cell_longPolyA			0.00	3.49	0.00
HepG2_cytosol_longNonPolyA			0.00	1.48	0.00
HepG2_cytosol_longPolyA			0.00	3.06	0.00
HepG2_nucleus_longNonPolyA			0.22	2.49	0.00
HepG2_nucleus_longPolyA			0.00	0.80	0.00
HFDPC_cell_total	HFDPC	Follicle Dermal Papilla Cells	30.68	3.01	0.00
hMSC_AT_cell_total	hMSC-AT	Undifferentiated Mesenchymal Stem Cells from subcutaneous abdomen adipose tissue	30.54	9.13	0.00
hMSC_BM_cell_total	hMSC-BM	Undifferentiated Mesenchymal Stem Cells from femoral head	7.10	7.27	0.00
hMSC_UC_cell_total	hMSC-UC	Undifferentiated Mesenchymal Stem Cells from matrix (Wharton's Jelly)	6.71	7.42	0.00
HNEpC_cell_total			93.39	4.02	0.00
HOB_cell_total	HOB	Undifferentiated Osteoblasts femoral head	422.49	6.06	0.00
HPAEC_cell_total	HPAEC	Human Pulmonary Artery Endothelial Cells	3247.85	9.84	0.00
HPASMC_cell_total	HPASMC	Human Pulmonary Artery Smooth Muscle Cells	1547.67	4.19	0.00
HPC_PL_cell_total	HPC-PL	Undifferentiated Pericytes	4.98	2.37	0.00
HPF_cell_total	HPF	Human Pulmonary Fibroblasts	258.70	8.53	0.00
HPIEpC_cell_total	HPIEpC	Placental Epithelial Cells amniotic membrane	104.99	4.18	0.00
HPMEC_cell_total	HPMEC	Human Pulmonary Microvascular Endothelial Cells	4270.35	7.02	0.00
HRCEpC_cell_total	HRCEpC	Human Renal Cortical Epithelial Cells	125.07	31.05	0.00
HSaVEC_cell_total	HSaVEC	Saphenous Vein Endothelial Cells from thigh	1336.69	11.39	0.00
HSMM_cell_longNonPolyA	HSMM	Human Skeletal Muscle Myoblast	281.80	0.38	0.00
HSMM_cell_longPolyA			1147.55	2.37	0.00

HTEpC_cell_total	HTEpC	Human Tracheal Epithelial Cells	88.08	4.24	0.00
HTSMC_cell_total	HTSMC	Human Tracheal Smooth Muscle Cells	143.50	7.33	0.00
HUAEC_cell_total	HUAEC	Human Aortic Endothelial Cells	651.03	3.35	0.00
HUASMC_cell_total	HUASMC	Human Umbilical Artery Smooth Muscle Cells	47.92	6.03	0.00
HUtSMC_cell_total	HUtSMC	Human Uterine Smooth Muscle Cells	4.04	3.22	0.00
HUVEC_cell_longNonPolyA	HUVEC	umbilical vein endothelial cells	3868.04	2.53	0.00
HUVEC_cell_longPolyA			7680.14	3.20	0.00
HUVEC_cytosol_longPolyA			7580.66	7.87	0.00
HUVEC_nucleus_longNonPolyA			970.35	2.82	0.00
HUVEC_nucleus_longPolyA			2714.06	2.84	0.00
HVMF_cell_total	HVMF	villous mesenchymal fibroblast cells	6.10	3.42	0.00
HWP_cell_total	HWP	Undifferentiated White Preadipocytes, subcutaneous adipose tissue from abdomen / upper arm	25.77	10.94	0.00
IMR90_cell_longPolyA	IMR90	fetal lung fibroblasts	192.50	6.78	0.00
IMR90_cell_total			123.90	3.79	0.00
IMR90_cytosol_longPolyA			186.03	2.40	0.00
IMR90_nucleus_longPolyA			49.86	0.36	0.00
K562_cell_longNonPolyA	K562	leukemia	0.81	1.53	0.00
K562_cell_longPolyA			4.74	4.43	0.00
K562_chromatin_total			0.37	1.23	0.00
K562_cytosol_longNonPolyA			0.00	0.46	0.00
K562_cytosol_longPolyA			1.40	1.50	0.00
K562_nucleolus_total			0.59	0.38	0.00
K562_nucleoplasm_total			0.41	0.85	0.00
K562_nucleus_longNonPolyA			0.46	0.89	0.00
K562_nucleus_longPolyA			0.28	0.36	0.00
Liver_STL004_Liver_STL011_cell_total		Liver	3.63	1.77	0.06
MCF_7_cell_longNonPolyA	MCF-7	mammary gland, adenocarcinoma	10.68	0.43	0.00
MCF_7_cell_longPolyA			14.85	14.35	0.00
MCF_7_cytosol_longPolyA			20.45	11.04	0.00
MCF_7_nucleus_longPolyA			6.19	3.88	0.03
Monocytes_CD14_Plus_cell_longNonPolyA	Monocytes-CD14+	Monocytes-CD14+ are CD14-positive cells from human leukapheresis production	0.00	6.62	0.00
Monocytes_CD14_Plus_cell_longPolyA			0.00	8.48	0.00
NHDF_cell_total	NHDF	Dermal Fibroblasts from temple / breast	8.06	9.32	0.00

NHEK_cell_longNonPolyA	NHEK	epidermal keratinocytes	95.88	4.13	0.00
NHEK_cell_longPolyA			195.39	12.39	0.00
NHEK_cytosol_longPolyA			96.90	10.77	0.00
NHEK_nucleus_longNonPolyA			26.50	4.34	0.00
NHEK_nucleus_longPolyA			132.83	7.81	0.00
NHEM_f_M2_cell_total	NHEM.f_M2	Epidermal Melanocytes from foreskin	3.77	2.27	0.00
NHEM_M2_cell_total	NHEM_M2	Epidermal Melanocytes (adult) from cheek / temple	2.84	2.66	0.28
NHLF_cell_longNonPolyA	NHLF	Normal Human Lung Fibroblasts	88.40	0.67	0.00
NHLF_cell_longPolyA			416.63	14.80	0.00
SK_N_SH_cell_longPolyA	SK-N-SH	neuroblastoma	1042.44	0.93	0.05
SK_N_SH_cytosol_longPolyA			1098.83	1.46	0.15
SK_N_SH_nucleus_longPolyA			467.35	0.26	0.16
SK_N_SH_RA_cell_longNonPolyA	SK-N-SH_RA	neuroblastoma cell line, treatment: differentiated with retinoic acid	26.23	0.29	1.70
SK_N_SH_RA_cell_longPolyA			91.36	1.96	1.89
SkMC_cell_total	SKMC	skeletal muscle cells	74.16	12.48	0.00

RPKM: reads per kilobase per million mapped reads

Supplementary Table 8. Regulatory features of three newly identified disease associations and all variants in high LD (r^2>0.8) with these variants. All variants were annotated using CADD[1] v1·3 (GRCh37/hg19;), MutationTaster[3] (NCBI build 37, Ensemble 69) and HaploReg[4] v4·1 to determine regulatory features including chromatin state, protein binding annotation and the effect of SNPs on regulatory motifs (**Methods**).

	CADD															MutationTaster	HaploReg							
SNP	CHR	BP	AnnoType	Consequence	EncExp	EncH3K27Ac	EncH3K4Me1	EncH3K4Me3	tOverlapMotifs	motifDist	TFBS	TFBSPeaks	TFBSPeaksMax	minDistTSS	minDistTSE	regulatory features	Chromatin States#	Chromatin States Imputed#	Chromatin Marks#	Promoter histone marks#	Enhancer histone marks#	DNAse#	Proteins bound by ChIP (ENCODE)	Regulatory motifs altered
rs17278665	2	56,095,994	Transcript	INTRONIC	43.90	4.00	8.48	3.00	1	1.00				54363	1999	H3K36me3, Histone, Histone 3 Lysine 36 Tri-Methylation	E025,6_EnhG; E026,6_EnhG; E097,7_Enh; E122,6_EnhG; E129,6_EnhG	E023,11_TxEnh3; E025,11_TxEnh3; E026,10_TxEnh5; E049,11_TxEnh3; E052,11_TxEnh3; E122,11_TxEnh3; E125,11_TxEnh3; E126,11_TxEnh3; E129,11_TxEnh3	E023,H3K4me1_Enh; E025,H3K4me1_Enh; E026,H3K4me1_Enh; E049,H3K4me1_Enh; E052,H3K4me1_Enh; E068,H3K4me1_Enh; E097,H3K4me1_Enh; E122,H3K4me1_Enh; E126,H3K4me1_Enh; E128,H3K4me1_Enh; E129,H3K4me1_Enh; E023,H3K4me3_Pro; E026,H3K4me3_Pro; E023,H3K9ac_Pro; E025,H3K9ac_Pro; E026,H3K9ac_Pro; E052,H3K9ac_Pro; E063,H3K9ac_Pro; E122,H3K9ac_Pro; E026,H3K27ac_Enh; E063,H3K27ac_Enh; E126,H3K27ac_Enh; E129,H3K27ac_Enh	.	FAT, STRM, OVRY, VAS, BONE	.	.	AIRE, CTCF, NRSF, Sin3Ak-20, TCF12
rs11899888	2	56,102,744	RegulatoryFeature	REGULATORY		32.40	20.96	4.00			1	1	25.3025	47613	8749	DNase1, Open Chromatin, DNase1 Hypersensitive Site<br>H3K36me3, Histone, Histone 3 Lysine 36 Tri-Methylation<br>Gene Associated, Regulatory Feature, Gene associated regulatory feature	E023,7_Enh; E025,7_Enh; E026,6_EnhG; E049,7_Enh; E052,7_Enh; E055,7_Enh; E066,7_Enh; E114,7_Enh; E117,7_Enh; E120,6_EnhG; E121,7_Enh; E122,6_EnhG; E125,7_Enh; E126,7_Enh; E128,6_EnhG; E129,6_EnhG	E006,17_EnhW2; E011,19_DNase; E013,18_EnhAc; E017,15_EnhAF; E023,14_EnhA2; E025,13_EnhA1; E026,13_EnhA1; E049,13_EnhA1; E052,13_EnhA1; E055,14_EnhA2; E056,17_EnhW2; E063,14_EnhA2; E065,17_EnhW2; E066,14_EnhA2; E078,19_DNase; E086,19_DNase; E099,19_DNase; E114,15_EnhAF; E117,14_EnhA2; E120,15_EnhAF; E121,14_EnhA2; E122,15_EnhAF; E125,13_EnhA1; E126,15_EnhAF; E128,14_EnhA2; E129,13_EnhA1	E010,H3K4me1_Enh; E013,H3K4me1_Enh; E017,H3K4me1_Enh; E023,H3K4me1_Enh; E025,H3K4me1_Enh; E026,H3K4me1_Enh; E027,H3K4me1_Enh; E028,H3K4me1_Enh; E049,H3K4me1_Enh; E052,H3K4me1_Enh; E055,H3K4me1_Enh; E058,H3K4me1_Enh; E063,H3K4me1_Enh; E065,H3K4me1_Enh; E066,H3K4me1_Enh; E074,H3K4me1_Enh; E099,H3K4me1_Enh; E108,H3K4me1_Enh; E114,H3K4me1_Enh; E117,H3K4me1_Enh; E120,H3K4me1_Enh; E121,H3K4me1_Enh; E122,H3K4me1_Enh; E125,H3K4me1_Enh; E126,H3K4me1_Enh; E128,H3K4me1_Enh; E129,H3K4me1_Enh; E011,H3K27ac_Enh; E017,H3K27ac_Enh; E026,H3K27ac_Enh; E049,H3K27ac_Enh; E055,H3K27ac_Enh; E056,H3K27ac_Enh; E058,H3K27ac_Enh; E063,H3K27ac_Enh; E065,H3K27ac_Enh; E066,H3K27ac_Enh; E095,H3K27ac_Enh; E097,H3K27ac_Enh; E120,H3K27ac_Enh; E121,H3K27ac_Enh; E122,H3K27ac_Enh; E125,H3K27ac_Enh; E126,H3K27ac_Enh; E128,H3K27ac_Enh; E129,H3K27ac_Enh; E023,H3K4me3_Pro; E025,H3K4me3_Pro; E052,H3K4me3_Pro; E125,H3K4me3_Pro; E023,H3K9ac_Pro; E025,H3K9ac_Pro; E026,H3K9ac_Pro; E052,H3K9ac_Pro; E063,H3K9ac_Pro; E122,H3K9ac_Pro; E125,H3K9ac_Pro; E128,H3K9ac_Pro	.	FAT, STRM, MUS, SKIN, LIV, LNG, CRVX, VAS, BRN, BONE	E017;E056;E117;E120;E121;E122;E125;E126;E128	.	Pax-8, SIX5
rs13064845	3	19,312,697	Transcript	INTRONIC		2.00	2.00	3.00						122671	263535		E061,7_Enh; E067,7_Enh	E061,19_DNase	E059,H3K4me1_Enh; E061,H3K4me1_Enh; E067,H3K4me1_Enh; E068,H3K4me1_Enh; E071,H3K4me1_Enh; E072,H3K4me1_Enh; E074,H3K4me1_Enh; E081,H3K4me1_Enh; E082,H3K4me1_Enh; E061,H3K27ac_Enh	.	SKIN, BRN	E059;E081	.	.
rs727139	3	19,334,695	Transcript	INTRONIC		4.36	10.84	3.16						144669	241537	H3K27ac, Histone, Histone 3 Lysine 27 Acetylation	E001,2_TssAFlnk; E002,7_Enh; E003,7_Enh; E004,7_Enh; E008,7_Enh; E014,7_Enh; E015,7_Enh; E016,7_Enh; E018,7_Enh; E019,7_Enh; E020,2_TssAFlnk; E021,7_Enh	E001,14_EnhA2; E002,16_EnhW1; E003,15_EnhAF; E004,14_EnhA2; E008,15_EnhAF; E011,17_EnhW2; E012,17_EnhW2; E014,14_EnhA2; E015,14_EnhA2; E016,14_EnhA2; E018,14_EnhA2; E019,15_EnhAF; E020,14_EnhA2; E021,15_EnhAF; E022,15_EnhAF; E024,18_EnhAc	E001,H3K4me1_Enh; E002,H3K4me1_Enh; E003,H3K4me1_Enh; E004,H3K4me1_Enh; E008,H3K4me1_Enh; E010,H3K4me1_Enh; E014,H3K4me1_Enh; E015,H3K4me1_Enh; E016,H3K4me1_Enh; E018,H3K4me1_Enh; E019,H3K4me1_Enh; E020,H3K4me1_Enh; E021,H3K4me1_Enh; E022,H3K4me1_Enh; E024,H3K4me1_Enh; E001,H3K4me3_Pro; E002,H3K4me3_Pro; E018,H3K4me3_Pro; E020,H3K4me3_Pro; E003,H3K27ac_Enh; E004,H3K27ac_Enh; E008,H3K27ac_Enh; E014,H3K27ac_Enh; E015,H3K27ac_Enh; E016,H3K27ac_Enh; E020,H3K27ac_Enh; E022,H3K27ac_Enh; E004,H3K9ac_Pro; E016,H3K9ac_Pro; E018,H3K9ac_Pro; E020,H3K9ac_Pro	ESC, IPSC	ESC, ESDR, IPSC	E021	.	HNF4, PPAR, RXRA, SP1, YY1
rs212838	7	26,698,194	Intergenic	INTERGENIC		5.00	15.00	4.64			4	4	104.0720	206169	8488		E001,2_TssAFlnk; E003,7_Enh; E007,7_Enh; E009,2_TssAFlnk; E010,2_TssAFlnk; E011,7_Enh; E012,2_TssAFlnk; E013,7_Enh; E014,2_TssAFlnk; E015,7_Enh; E016,2_TssAFlnk; E018,2_TssAFlnk; E019,2_TssAFlnk; E020,2_TssAFlnk; E024,7_Enh; E036,7_Enh; E053,2_TssAFlnk; E054,7_Enh; E070,2_TssAFlnk; E073,7_Enh; E081,7_Enh; E082,1_TssA; E088,12_EnhBiv; E091,7_Enh	E001,14_EnhA2; E002,15_EnhAF; E003,15_EnhAF; E004,15_EnhAF; E005,18_EnhAc; E007,15_EnhAF; E008,15_EnhAF; E009,14_EnhA2; E010,2_PromU; E011,13_EnhA1; E012,2_PromU; E013,15_EnhAF; E014,2_PromU; E015,13_EnhA1; E016,2_PromU; E018,14_EnhA2; E019,2_PromU; E020,13_EnhA1; E021,15_EnhAF; E022,17_EnhW2; E024,15_EnhAF; E053,2_PromU; E054,2_PromU; E067,17_EnhW2; E068,19_DNase; E069,18_EnhAc; E070,14_EnhA2; E071,18_EnhAc; E072,18_EnhAc; E073,18_EnhAc; E074,14_EnhA2; E081,13_EnhA1; E082,13_EnhA1; E083,17_EnhW2; E091,17_EnhW2; E099,18_EnhAc	E001,H3K4me1_Enh; E002,H3K4me1_Enh; E003,H3K4me1_Enh; E007,H3K4me1_Enh; E008,H3K4me1_Enh; E009,H3K4me1_Enh; E010,H3K4me1_Enh; E011,H3K4me1_Enh; E012,H3K4me1_Enh; E013,H3K4me1_Enh; E014,H3K4me1_Enh; E015,H3K4me1_Enh; E016,H3K4me1_Enh; E018,H3K4me1_Enh; E019,H3K4me1_Enh; E020,H3K4me1_Enh; E021,H3K4me1_Enh; E024,H3K4me1_Enh; E035,H3K4me1_Enh; E036,H3K4me1_Enh; E053,H3K4me1_Enh; E054,H3K4me1_Enh; E061,H3K4me1_Enh; E067,H3K4me1_Enh; E069,H3K4me1_Enh; E070,H3K4me1_Enh; E071,H3K4me1_Enh; E073,H3K4me1_Enh; E074,H3K4me1_Enh; E081,H3K4me1_Enh; E082,H3K4me1_Enh; E086,H3K4me1_Enh; E087,H3K4me1_Enh; E088,H3K4me1_Enh; E090,H3K4me1_Enh; E091,H3K4me1_Enh; E092,H3K4me1_Enh; E001,H3K4me3_Pro; E003,H3K4me3_Pro; E009,H3K4me3_Pro; E010,H3K4me3_Pro; E012,H3K4me3_Pro; E014,H3K4me3_Pro; E015,H3K4me3_Pro; E016,H3K4me3_Pro; E018,H3K4me3_Pro; E019,H3K4me3_Pro; E020,H3K4me3_Pro; E053,H3K4me3_Pro; E054,H3K4me3_Pro; E070,H3K4me3_Pro; E074,H3K4me3_Pro; E081,H3K4me3_Pro; E082,H3K4me3_Pro; E001,H3K9ac_Pro; E008,H3K9ac_Pro; E014,H3K9ac_Pro; E015,H3K9ac_Pro; E019,H3K9ac_Pro; E020,H3K9ac_Pro; E073,H3K9ac_Pro; E074,H3K9ac_Pro; E003,H3K27ac_Enh; E007,H3K27ac_Enh; E008,H3K27ac_Enh; E011,H3K27ac_Enh; E012,H3K27ac_Enh; E013,H3K27ac_Enh; E014,H3K27ac_Enh; E015,H3K27ac_Enh; E016,H3K27ac_Enh; E019,H3K27ac_Enh; E020,H3K27ac_Enh; E021,H3K27ac_Enh; E022,H3K27ac_Enh; E061,H3K27ac_Enh; E067,H3K27ac_Enh; E069,H3K27ac_Enh; E071,H3K27ac_Enh; E072,H3K27ac_Enh; E073,H3K27ac_Enh; E074,H3K27ac_Enh; E091,H3K27ac_Enh	ESC, ESDR, IPSC, BRN	ESC, ESDR, BLD, BRN, LNG, PLCNT	E008;E081;E082;E091	ERALPHA_A, FOXA1, GATA3, P300	Foxj2, Irx, Mrg1::Hoxa9, Pou2f2, THAP1, YY1, p53
rs3757654	7	26,711,104	Transcript	INTRONIC		1.12	4.32	3.00						186136	2056	H3K4me1, Histone, Histone 3 Lysine 4 Mono-Methylation	E036,7_Enh; E050,7_Enh; E051,2_TssAFlnk	E035,17_EnhW2; E036,15_EnhAF; E050,14_EnhA2; E051,13_EnhA1	E016,H3K4me1_Enh; E035,H3K4me1_Enh; E036,H3K4me1_Enh; E050,H3K4me1_Enh; E051,H3K4me1_Enh; E087,H3K4me1_Enh; E050,H3K27ac_Enh; E050,H3K4me3_Pro; E051,H3K4me3_Pro	BLD	BLD	.	.	.
rs212848	7	26,722,155	Transcript	INTRONIC	1.91	4.00	25.76	4.00						175085	13107	H3K36me3, Histone, Histone 3 Lysine 36 Tri-Methylation<br>H3K27me3, Histone, Histone 3 Lysine 27 Tri-Methylation	E036,7_Enh; E050,7_Enh; E051,2_TssAFlnk; E063,7_Enh; E077,7_Enh; E088,12_EnhBiv; E102,7_Enh; E109,7_Enh; E110,7_Enh; E122,7_Enh	E033,22_PromP; E035,16_EnhW1; E036,16_EnhW1; E050,14_EnhA2; E051,13_EnhA1; E077,17_EnhW2; E086,17_EnhW2; E088,16_EnhW1; E101,17_EnhW2; E102,17_EnhW2; E110,17_EnhW2; E122,18_EnhAc	E023,H3K4me1_Enh; E030,H3K4me1_Enh; E032,H3K4me1_Enh; E036,H3K4me1_Enh; E050,H3K4me1_Enh; E051,H3K4me1_Enh; E061,H3K4me1_Enh; E063,H3K4me1_Enh; E077,H3K4me1_Enh; E078,H3K4me1_Enh; E084,H3K4me1_Enh; E086,H3K4me1_Enh; E088,H3K4me1_Enh; E102,H3K4me1_Enh; E109,H3K4me1_Enh; E110,H3K4me1_Enh; E122,H3K4me1_Enh; E050,H3K4me3_Pro; E051,H3K4me3_Pro; E063,H3K9ac_Pro; E066,H3K9ac_Pro; E088,H3K9ac_Pro; E102,H3K9ac_Pro; E122,H3K27ac_Enh	BLD	BLD, FAT, GI, LNG, VAS	E007;E088	.	.
rs212850	7	26,724,279	RegulatoryFeature	REGULATORY		16.24	48.08	5.60						172961	15231	DNase1, Open Chromatin, DNase1 Hypersensitive Site<br>H3K4me1, Histone, Histone 3 Lysine 4 Mono-Methylation<br>H3K36me3, Histone, Histone 3 Lysine 36 Tri-Methylation<br>H3K27me3, Histone, Histone 3 Lysine 27 Tri-Methylation<br>Gene Associated, Regulatory Feature, Gene associated regulatory feature	E036,7_Enh; E050,7_Enh; E051,7_Enh; E088,12_EnhBiv; E110,7_Enh; E122,7_Enh	E035,17_EnhW2; E051,18_EnhAc; E063,17_EnhW2; E083,19_DNase; E086,17_EnhW2; E088,18_EnhAc; E122,13_EnhA1	E001,H3K4me1_Enh; E011,H3K4me1_Enh; E013,H3K4me1_Enh; E018,H3K4me1_Enh; E025,H3K4me1_Enh; E036,H3K4me1_Enh; E050,H3K4me1_Enh; E051,H3K4me1_Enh; E061,H3K4me1_Enh; E066,H3K4me1_Enh; E077,H3K4me1_Enh; E078,H3K4me1_Enh; E086,H3K4me1_Enh; E088,H3K4me1_Enh; E092,H3K4me1_Enh; E102,H3K4me1_Enh; E110,H3K4me1_Enh; E111,H3K4me1_Enh; E114,H3K4me1_Enh; E122,H3K4me1_Enh; E023,H3K9ac_Pro; E025,H3K9ac_Pro; E063,H3K9ac_Pro; E066,H3K9ac_Pro; E077,H3K9ac_Pro; E088,H3K9ac_Pro; E102,H3K9ac_Pro; E110,H3K9ac_Pro; E122,H3K9ac_Pro; E050,H3K27ac_Enh; E063,H3K27ac_Enh; E078,H3K27ac_Enh; E089,H3K27ac_Enh; E103,H3K27ac_Enh; E122,H3K27ac_Enh; E051,H3K4me3_Pro; E088,H3K4me3_Pro; E111,H3K4me3_Pro; E122,H3K4me3_Pro	.	BLD, LNG, GI, VAS	E088;E122	.	NRSF, Rhox11
rs212856	7	26,729,205	RegulatoryFeature	REGULATORY		12.60	45.32	9.72			3	3	96.5614	52890	722	H3K9me1, Histone, Histone 3 Lysine 9 mono-methylation<br>DNase1, Open Chromatin, DNase1 Hypersensitive Site<br>H3K4me1, Histone, Histone 3 Lysine 4 Mono-Methylation<br>H3K36me3, Histone, Histone 3 Lysine 36 Tri-Methylation<br>Gene Associated, Regulatory Feature, Gene associated regulatory feature	E011,7_Enh; E017,7_Enh; E023,7_Enh; E028,6_EnhG; E035,7_Enh; E036,7_Enh; E050,2_TssAFlnk; E051,2_TssAFlnk; E063,7_Enh; E077,7_Enh; E078,7_Enh; E080,7_Enh; E084,7_Enh; E085,7_Enh; E086,7_Enh; E088,12_EnhBiv; E090,7_Enh; E097,7_Enh; E100,7_Enh; E102,7_Enh; E106,7_Enh; E108,7_Enh; E109,7_Enh; E114,7_Enh; E116,7_Enh; E118,7_Enh; E119,7_Enh; E122,7_Enh; E123,7_Enh	E023,17_EnhW2; E027,17_EnhW2; E030,17_EnhW2; E031,17_EnhW2; E033,17_EnhW2; E035,15_EnhAF; E036,14_EnhA2; E050,14_EnhA2; E051,13_EnhA1; E063,14_EnhA2; E066,17_EnhW2; E075,17_EnhW2; E076,17_EnhW2; E077,15_EnhAF; E078,15_EnhAF; E080,15_EnhAF; E081,17_EnhW2; E084,15_EnhAF; E085,15_EnhAF; E086,17_EnhW2; E088,14_EnhA2; E089,17_EnhW2; E090,17_EnhW2; E092,17_EnhW2; E095,17_EnhW2; E096,17_EnhW2; E097,17_EnhW2; E101,17_EnhW2; E102,15_EnhAF; E103,17_EnhW2; E109,15_EnhAF; E110,15_EnhAF; E111,17_EnhW2; E113,17_EnhW2; E115,19_DNase; E118,15_EnhAF; E122,18_EnhAc; E123,15_EnhAF; E124,17_EnhW2	E005,H3K4me1_Enh; E010,H3K4me1_Enh; E011,H3K4me1_Enh; E013,H3K4me1_Enh; E017,H3K4me1_Enh; E023,H3K4me1_Enh; E025,H3K4me1_Enh; E028,H3K4me1_Enh; E029,H3K4me1_Enh; E031,H3K4me1_Enh; E032,H3K4me1_Enh; E035,H3K4me1_Enh; E036,H3K4me1_Enh; E050,H3K4me1_Enh; E051,H3K4me1_Enh; E057,H3K4me1_Enh; E058,H3K4me1_Enh; E061,H3K4me1_Enh; E062,H3K4me1_Enh; E063,H3K4me1_Enh; E066,H3K4me1_Enh; E075,H3K4me1_Enh; E077,H3K4me1_Enh; E078,H3K4me1_Enh; E080,H3K4me1_Enh; E084,H3K4me1_Enh; E085,H3K4me1_Enh; E086,H3K4me1_Enh; E088,H3K4me1_Enh; E090,H3K4me1_Enh; E092,H3K4me1_Enh; E095,H3K4me1_Enh; E096,H3K4me1_Enh; E097,H3K4me1_Enh; E100,H3K4me1_Enh; E101,H3K4me1_Enh; E102,H3K4me1_Enh; E103,H3K4me1_Enh; E104,H3K4me1_Enh; E106,H3K4me1_Enh; E108,H3K4me1_Enh; E109,H3K4me1_Enh; E110,H3K4me1_Enh; E113,H3K4me1_Enh; E114,H3K4me1_Enh; E116,H3K4me1_Enh; E117,H3K4me1_Enh; E118,H3K4me1_Enh; E119,H3K4me1_Enh; E122,H3K4me1_Enh; E123,H3K4me1_Enh; E124,H3K4me1_Enh; E007,H3K27ac_Enh; E013,H3K27ac_Enh; E017,H3K27ac_Enh; E032,H3K27ac_Enh; E050,H3K27ac_Enh; E063,H3K27ac_Enh; E075,H3K27ac_Enh; E080,H3K27ac_Enh; E084,H3K27ac_Enh; E085,H3K27ac_Enh; E089,H3K27ac_Enh; E090,H3K27ac_Enh; E092,H3K27ac_Enh; E096,H3K27ac_Enh; E101,H3K27ac_Enh; E106,H3K27ac_Enh; E109,H3K27ac_Enh; E113,H3K27ac_Enh; E118,H3K27ac_Enh; E122,H3K27ac_Enh; E123,H3K27ac_Enh; E023,H3K9ac_Pro; E063,H3K9ac_Pro; E088,H3K9ac_Pro; E124,H3K9ac_Pro; E035,H3K4me3_Pro; E036,H3K4me3_Pro; E050,H3K4me3_Pro; E051,H3K4me3_Pro; E088,H3K4me3_Pro; E114,H3K4me3_Pro; E123,H3K4me3_Pro	BLD	ESDR, LNG, FAT, BRST, BLD, GI, ADRL, KID, MUS, OVRY, LIV, VAS	E084;E085;E123	GATA1	Irf, SMC3
rs212857	7	26,730,200	Transcript	INTRONIC	0.81	2.00	22.68	5.00						51895	274	H3K9me1, Histone, Histone 3 Lysine 9 mono-methylation<br>H3K4me1, Histone, Histone 3 Lysine 4 Mono-Methylation<br>H3K36me3, Histone, Histone 3 Lysine 36 Tri-Methylation	E023,7_Enh; E035,7_Enh; E036,7_Enh; E050,7_Enh; E051,7_Enh; E057,7_Enh; E063,7_Enh; E084,7_Enh; E085,7_Enh; E118,7_Enh; E122,7_Enh; E128,7_Enh	E031,17_EnhW2; E033,17_EnhW2; E035,17_EnhW2; E036,17_EnhW2; E050,14_EnhA2; E051,14_EnhA2; E063,19_DNase; E069,19_DNase; E073,19_DNase; E074,19_DNase; E080,19_DNase; E084,19_DNase; E085,19_DNase; E088,19_DNase; E089,19_DNase; E090,19_DNase; E092,19_DNase; E100,19_DNase; E105,19_DNase; E107,19_DNase; E108,19_DNase; E111,19_DNase; E124,17_EnhW2	E013,H3K27ac_Enh; E050,H3K27ac_Enh; E063,H3K27ac_Enh; E080,H3K27ac_Enh; E084,H3K27ac_Enh; E085,H3K27ac_Enh; E089,H3K27ac_Enh; E090,H3K27ac_Enh; E013,H3K4me1_Enh; E017,H3K4me1_Enh; E018,H3K4me1_Enh; E023,H3K4me1_Enh; E025,H3K4me1_Enh; E028,H3K4me1_Enh; E035,H3K4me1_Enh; E036,H3K4me1_Enh; E050,H3K4me1_Enh; E051,H3K4me1_Enh; E055,H3K4me1_Enh; E057,H3K4me1_Enh; E058,H3K4me1_Enh; E062,H3K4me1_Enh; E063,H3K4me1_Enh; E066,H3K4me1_Enh; E075,H3K4me1_Enh; E077,H3K4me1_Enh; E078,H3K4me1_Enh; E084,H3K4me1_Enh; E085,H3K4me1_Enh; E086,H3K4me1_Enh; E088,H3K4me1_Enh; E092,H3K4me1_Enh; E102,H3K4me1_Enh; E103,H3K4me1_Enh; E109,H3K4me1_Enh; E114,H3K4me1_Enh; E117,H3K4me1_Enh; E118,H3K4me1_Enh; E122,H3K4me1_Enh; E127,H3K4me1_Enh; E128,H3K4me1_Enh; E129,H3K4me1_Enh; E050,H3K4me3_Pro; E051,H3K4me3_Pro; E088,H3K4me3_Pro; E063,H3K9ac_Pro; E088,H3K9ac_Pro; E124,H3K9ac_Pro; E127,H3K9ac_Pro	.	FAT, BLD, SKIN, GI, LIV, VAS, LNG	E088;E089;E090;E092;E097	.	CEBPB
rs4719877	7	26,736,220	Transcript	INTRONIC	0.44	1.00	6.68	2.28						45875	6294		.	.	E010,H3K4me1_Enh; E023,H3K4me1_Enh; E028,H3K4me1_Enh; E036,H3K4me1_Enh; E050,H3K4me1_Enh; E075,H3K4me1_Enh; E101,H3K4me1_Enh; E114,H3K4me1_Enh; E027,H3K9ac_Pro; E038,H3K9ac_Pro; E063,H3K27ac_Enh	.	.	.	.	.
rs6461962	7	26,746,304	Transcript	INTRONIC		3.44	15.56	3.24						35791	16378	H3K4me1, Histone, Histone 3 Lysine 4 Mono-Methylation	E025,7_Enh; E050,7_Enh; E052,7_Enh; E122,7_Enh; E126,7_Enh; E128,7_Enh	E023,17_EnhW2	E017,H3K4me1_Enh; E023,H3K4me1_Enh; E025,H3K4me1_Enh; E028,H3K4me1_Enh; E049,H3K4me1_Enh; E050,H3K4me1_Enh; E051,H3K4me1_Enh; E052,H3K4me1_Enh; E055,H3K4me1_Enh; E058,H3K4me1_Enh; E061,H3K4me1_Enh; E076,H3K4me1_Enh; E078,H3K4me1_Enh; E086,H3K4me1_Enh; E088,H3K4me1_Enh; E092,H3K4me1_Enh; E103,H3K4me1_Enh; E111,H3K4me1_Enh; E114,H3K4me1_Enh; E121,H3K4me1_Enh; E122,H3K4me1_Enh; E125,H3K4me1_Enh; E126,H3K4me1_Enh; E127,H3K4me1_Enh; E128,H3K4me1_Enh; E023,H3K9ac_Pro; E066,H3K9ac_Pro; E122,H3K9ac_Pro; E050,H3K27ac_Enh; E058,H3K27ac_Enh; E063,H3K27ac_Enh; E076,H3K27ac_Enh; E111,H3K27ac_Enh; E119,H3K27ac_Enh	.	FAT, BLD, MUS, VAS, SKIN, LNG	.	.	.
rs6972378	7	26,753,074	Transcript	INTRONIC		4.00	2.00	3.00						29021	23148		.	.	E011,H3K4me1_Enh; E092,H3K4me1_Enh	.	.	.	.	.
rs6951065	7	26,756,162	Transcript	INTRONIC		1.32	2.00	1.04						25933	26236		.	.	0	.	.	.	.	Foxp1, Mef2, Pou3f3
rs6964817	7	26,756,424	Transcript	INTRONIC	0.89	6.00	1.00	7.28						25671	26498		.	.	E050,H3K4me1_Enh	.	.	.	.	EWSR1-FLI1, HDAC2, Irf, NRSF, Pax-4, SP1, TFII-I, ZNF263
rs2106905	7	26,766,851	Transcript	INTRONIC	0.89	3.00	2.00	4.52	2	0.43				15244	343		.	.	E008,H3K4me1_Enh; E018,H3K4me1_Enh; E078,H3K4me1_Enh; E124,H3K27ac_Enh	.	.	.	.	Maf, SRF
rs6461963	7	26,772,491	Transcript	INTRONIC	0.64	1.00	2.00	3.00						9604	5983	H3K36me3, Histone, Histone 3 Lysine 36 Tri-Methylation	.	.	0	.	.	.	.	PLZF
rs4722630	7	26,773,364	Transcript	INTRONIC		1.72	1.00	1.64						8731	6856		.	.	0	.	.	.	.	Hoxa10, Mef2
rs6971397	7	26,782,564	Transcript	INTRONIC		3.00	2.00	2.00						470	3058		.	.	E017,H3K4me1_Enh; E050,H3K4me1_Enh	.	.	E127	.	.
rs727450	7	26,787,642	Transcript	INTRONIC		1.00	3.00	4.00						107129	1456		.	.	E027,H3K9ac_Pro	.	.	.	.	HNF4, Hoxa10, Msx2, Prrx2, p300
rs10281261	7	26,789,472	Transcript	INTRONIC		2.00	1.00	2.32						105299	3286		.	.	0	.	.	.	.	GATA
rs7457076	7	26,793,215	Transcript	INTRONIC		3.96	2.00	2.00						101556	7029		.	.	E007,H3K4me1_Enh; E018,H3K4me1_Enh; E078,H3K4me1_Enh; E007,H3K9ac_Pro; E040,H3K4me3_Pro	.	.	.	.	CEBPB, Cdx2, Dbx2, Homez, Hoxa10, Hoxa9, Hoxb8, Hoxb9, Hoxc10, Hoxc9, Hoxd10, Pdx1, Pou2f2, Pou3f2, Sox
rs2189017	7	26,795,073	Transcript	INTRONIC	0.64	3.00	4.16	2.52						99698	8887		.	.	0	.	.	.	.	Dbx1, Ets, HNF1, Ncx, Nkx6-1, Pax7, Pou2f2, Pou6f1
rs2189019	7	26,798,581	Transcript	INTRONIC		3.00	2.00	2.00						96190	12395	H3K36me3, Histone, Histone 3 Lysine 36 Tri-Methylation	.	.	0	.	.	.	.	Hoxc10
rs73061894	7	26,802,085	Transcript	INTRONIC		2.00	2.00	3.00	2	0.76				92686	15899		.	.	E050,H3K4me1_Enh	.	.	.	.	DMRT2, Irx, Nanog, Pou2f2, Pou3f3, Pou5f1, TATA
rs117217399	7	26,805,212	Transcript	INTRONIC		1.68		2.88						89559	19026		.	.	0	.	.	.	.	AIRE, Hoxb13, Hoxb9, Pax-4, TATA, TBX5
rs10486478	7	26,819,465	Transcript	INTRONIC		2.52	2.00	2.20						75306	33279		.	.	E014,H3K4me1_Enh; E018,H3K4me1_Enh; E061,H3K4me1_Enh; E088,H3K4me1_Enh; E110,H3K4me1_Enh; E027,H3K9ac_Pro	.	.	.	.	Evi-1, Hoxa7, Obox6, Pou6f1
rs3801850	7	26,820,577	Transcript	INTRONIC		3.00	1.00	4.04						74194	34391		E061,7_Enh; E088,7_Enh	.	E010,H3K4me1_Enh; E014,H3K4me1_Enh; E016,H3K4me1_Enh; E018,H3K4me1_Enh; E020,H3K4me1_Enh; E028,H3K4me1_Enh; E061,H3K4me1_Enh; E088,H3K4me1_Enh; E110,H3K4me1_Enh; E027,H3K9ac_Pro	.	SKIN, LNG	.	.	Foxf1, Foxj1, Foxk1, Foxl1, Foxo, Hoxa9
rs12536560	7	26,822,876	Transcript	INTRONIC	0.64	5.68	5.92	2.84						71895	36690	H3K36me3, Histone, Histone 3 Lysine 36 Tri-Methylation<br>H3K27me3, Histone, Histone 3 Lysine 27 Tri-Methylation	E018,7_Enh; E019,7_Enh	.	E001,H3K4me1_Enh; E002,H3K4me1_Enh; E004,H3K4me1_Enh; E008,H3K4me1_Enh; E014,H3K4me1_Enh; E015,H3K4me1_Enh; E016,H3K4me1_Enh; E018,H3K4me1_Enh; E019,H3K4me1_Enh; E020,H3K4me1_Enh; E024,H3K4me1_Enh; E028,H3K4me1_Enh; E058,H3K4me1_Enh; E094,H3K4me1_Enh; E003,H3K27ac_Enh; E016,H3K27ac_Enh; E022,H3K27ac_Enh; E058,H3K27ac_Enh; E014,H3K4me3_Pro; E019,H3K4me3_Pro; E096,H3K4me3_Pro; E020,H3K9ac_Pro; E047,H3K9ac_Pro; E076,H3K9ac_Pro	.	IPSC	E124	.	Evi-1, HP1-site-factor
rs73065504	7	26,825,984	Transcript	INTRONIC	0.64	2.12	2.00	3.00						68787	39798		E010,7_Enh	.	E008,H3K4me1_Enh; E010,H3K4me1_Enh; E014,H3K4me1_Enh; E018,H3K4me1_Enh; E019,H3K4me1_Enh; E020,H3K4me1_Enh; E021,H3K4me1_Enh; E024,H3K4me1_Enh; E070,H3K4me1_Enh; E019,H3K27ac_Enh; E097,H3K27ac_Enh; E027,H3K9ac_Pro; E047,H3K9ac_Pro	.	ESDR	.	.	AIRE, Foxp1, GR, Irx, Zfp105
rs10267950	7	26,826,757	Transcript	INTRONIC		3.48	4.00	4.00						68014	40571		.	.	E008,H3K4me1_Enh; E015,H3K4me1_Enh; E018,H3K4me1_Enh; E019,H3K4me1_Enh; E024,H3K4me1_Enh; E070,H3K4me1_Enh; E027,H3K9ac_Pro; E047,H3K9ac_Pro	.	.	.	.	.
rs10272303	7	26,827,815	Transcript	INTRONIC		3.08	2.08	3.00						66956	41629		.	.	E014,H3K4me1_Enh; E018,H3K4me1_Enh; E019,H3K4me1_Enh; E024,H3K4me1_Enh; E070,H3K4me1_Enh; E027,H3K9ac_Pro; E113,H3K4me3_Pro	.	.	.	.	Hoxb13, Hoxd10
rs3801846	7	26,828,653	Transcript	INTRONIC		2.00	1.00	3.28						66118	42467		E023,7_Enh	.	E023,H3K4me1_Enh; E070,H3K4me1_Enh; E078,H3K4me1_Enh; E027,H3K9ac_Pro	.	FAT	.	.	AP-1, BCL, Dbx2, GATA, GR, HNF1, Hoxd8, Irf, PRDM1, RFX5, STAT, VDR, p300
rs1327988	7	26,828,965	Transcript	INTRONIC		4.00	2.00	5.00	1	1.00				65806	42779		E023,7_Enh	.	E023,H3K4me1_Enh; E025,H3K4me1_Enh; E070,H3K4me1_Enh; E078,H3K4me1_Enh	.	FAT	.	.	Elf3, Elf5, Maf, Nkx2, Nkx3, PU.1
rs3801844	7	26,829,759	Transcript	INTRONIC		3.00	2.00	2.00						65012	43573		E025,7_Enh; E063,7_Enh; E070,7_Enh; E126,7_Enh	E023,19_DNase; E025,19_DNase	E008,H3K27ac_Enh; E023,H3K4me1_Enh; E025,H3K4me1_Enh; E063,H3K4me1_Enh; E070,H3K4me1_Enh; E078,H3K4me1_Enh; E096,H3K4me1_Enh; E126,H3K4me1_Enh	.	FAT, BRN, SKIN	.	.	Cdx
rs3801843	7	26,830,201	Transcript	INTRONIC		2.00	1.00	2.00						64570	44015		.	.	E023,H3K4me1_Enh; E078,H3K4me1_Enh	.	.	.	.	.
rs3801842	7	26,830,263	Transcript	INTRONIC		2.00	2.28	3.00						64508	44077		.	.	E023,H3K4me1_Enh; E078,H3K4me1_Enh	.	.	.	.	Evi-1, Foxa, Foxi1, Foxj2, HDAC2, HNF4, Mef2, p300
rs73065517	7	26,830,555	Transcript	INTRONIC		2.00	1.00	1.00						64216	44369		.	.	E078,H3K4me1_Enh	.	.	.	.	Foxa, Ncx
rs28733208	7	26,830,825	Transcript	INTRONIC		4.00	1.00	2.64						63946	44639		.	.	E078,H3K4me1_Enh	.	.	.	.	Hoxa9, Mef2, PLZF, Pou2f2
rs73065521	7	26,831,409	Transcript	INTRONIC		1.00	3.92	3.32						63362	45223	H3K9me1, Histone, Histone 3 Lysine 9 mono-methylation	.	.	E061,H3K4me1_Enh; E078,H3K4me1_Enh	.	.	.	.	Bcl6b, Ik-2, Sp100
rs3801840	7	26,832,986	Transcript	INTRONIC		4.00	3.84	3.80						61785	46800		E001,7_Enh; E015,7_Enh; E061,7_Enh	E002,17_EnhW2	E001,H3K4me1_Enh; E014,H3K4me1_Enh; E015,H3K4me1_Enh; E016,H3K4me1_Enh; E027,H3K4me1_Enh; E061,H3K4me1_Enh; E078,H3K4me1_Enh; E002,H3K4me3_Pro; E018,H3K9ac_Pro; E027,H3K9ac_Pro; E047,H3K9ac_Pro	.	ESC, SKIN	.	.	Pax-6
rs1327989	7	26,833,563	Transcript	INTRONIC	1.27	2.00	2.00	0.32						61208	47377		E061,7_Enh	.	E016,H3K4me1_Enh; E027,H3K4me1_Enh; E061,H3K4me1_Enh; E018,H3K9ac_Pro; E027,H3K9ac_Pro; E047,H3K9ac_Pro	.	SKIN	.	.	Foxa, Irf
rs6964094	7	26,834,380	Transcript	INTRONIC		6.00	4.88	2.96						60391	48194	H3K36me3, Histone, Histone 3 Lysine 36 Tri-Methylation	.	.	E021,H3K27ac_Enh; E027,H3K9ac_Pro; E098,H3K4me1_Enh; E109,H3K4me1_Enh; E113,H3K4me1_Enh	.	.	.	.	Rad21, SP1
rs6956088	7	26,835,727	Transcript	INTRONIC		2.00	1.00	3.00						59044	49541		.	.	.	.	.	.	.	Dbx1, Fox, Foxf1, Foxi1, Foxj2, Foxp1, Mef2
rs10276471	7	26,837,940	Transcript	INTRONIC		1.08	0.80	2.16						56831	51754		.	.	E028,H3K4me1_Enh; E058,H3K4me1_Enh; E119,H3K4me1_Enh; E079,H3K27ac_Enh	.	.	.	.	Zbtb12
rs4722641	7	26,838,677	RegulatoryFeature	REGULATORY		2.20	5.00	2.00						56094	52491	DNase1, Open Chromatin, DNase1 Hypersensitive Site	E005,7_Enh; E058,7_Enh; E119,7_Enh; E127,7_Enh	E028,19_DNase; E058,19_DNase	E005,H3K4me3_Pro; E024,H3K4me3_Pro; E024,H3K4me1_Enh; E028,H3K4me1_Enh; E058,H3K4me1_Enh; E079,H3K4me1_Enh; E119,H3K4me1_Enh; E127,H3K4me1_Enh; E079,H3K27ac_Enh	.	ESDR, SKIN, BRST	E028;E057;E127	.	BCL, BHLHE40, E2F, Ets, GR, Irf, MZF1::1-4, NRSF, PU.1, Pou2f2, SP1, SRF, STAT, Sin3Ak-20, Sp4, UF1H3BETA
rs10225924	7	26,839,954	Transcript	INTRONIC		3.00	1.48	2.00						54817	53768		.	.	.	.	.	.	.	Foxo, Foxp1, GR, HDAC2, Irf, Mef2, Pou2f2, RREB-1
rs10229837	7	26,840,652	Transcript	INTRONIC		1.00	1.00	1.92						54119	54466		.	.	.	.	.	.	.	p300
rs12532395	7	26,841,702	Transcript	INTRONIC	1.27	3.00	2.00	3.00						53069	55516		.	.	.	.	.	.	.	.
rs12539719	7	26,844,852	Transcript	INTRONIC		1.00	1.00	2.96						49919	58666		.	.	.	.	.	.	.	Duxl, Fox, Jundm2
rs4722644	7	26,845,386	Transcript	INTRONIC		1.04	3.00	2.04						49385	59200		.	.	.	.	.	.	.	AP-1, Bach1, Cphx, Duxl, HNF1, Nkx2

rs12333525	7	26,847,762	Transcript	INTRONIC		2.00	1.00	2.88						47009	61576	H3K36me3, Histone, Histone 3 Lysine 36 Tri-Methylation	.	.	E058,H3K27ac_Enh	.	.	.	.	Barhl1, Barx1, Barx2, Cart1, Dbx1, Dbx2, En-1, Esx1, HNF1, Hmx, Hoxa7, Hoxb4, Hoxb7, Hoxb8, Hoxc6, Hoxd8, Isl2, Lhx4, Msx-1, Ncx, Nkx6-1, Nkx6-2, Pax-6, Pax7, Phox2a, Pou1f1, Pou2f2, Pou3f2, Pou3f4, Pou4f3, Pou6f1, Prrx1, Prrx2, Sox, Vax2, Zfp187
rs10244986	7	26,849,558	Transcript	INTRONIC		2.00	3.44	2.96						45213	63372	H3K36me3, Histone, Histone 3 Lysine 36 Tri-Methylation	.	.	E081,H3K4me1_Enh	.	.	.	.	BATF, Elf3, Elf5, Evi-1, Foxa, HDAC2, STAT
rs73067435	7	26,850,195	Transcript	INTRONIC	0.64	2.20	3.00	4.00						44576	64009	H3K9me1, Histone, Histone 3 Lysine 9 mono-methylation H3K36me3, Histone, Histone 3 Lysine 36 Tri-Methylation	.	.	E081,H3K4me1_Enh	.	.	.	.	Irx, Nkx3, SIX5, Zfp187
rs73067437	7	26,850,313	Transcript	INTRONIC		1.76	1.92	1.00						44458	64127	H3K36me3, Histone, Histone 3 Lysine 36 Tri-Methylation	.	.	.	.	.	.	.	FAC1, Foxa, Foxc1, Foxd3, Foxj2, Foxk1, Foxl1, Foxo, Foxp1, HDAC2, Zfp105, p300
rs57135974	7	26,850,699	Transcript	INTRONIC	0.64	6.04	1.00	2.00						44072	64513	H3K36me3, Histone, Histone 3 Lysine 36 Tri-Methylation	.	.	.	.	.	.	.	Gfi1, TCF4
rs58760954	7	26,850,776	Transcript	INTRONIC		6.00	2.00	3.00						43995	64590		.	.	.	.	.	.	.	Rad21, STAT
rs73067443	7	26,851,074	Transcript	INTRONIC	1.27	3.52	5.00	2.60						43697	64888		.	.	E010,H3K4me1_Enh; E056,H3K27ac_Enh	.	.	.	.	NRSF, RXRA
rs3801837	7	26,851,361	Transcript	INTRONIC		1.00	2.68	1.00						43410	65175		.	.	E010,H3K4me1_Enh; E086,H3K4me1_Enh; E056,H3K27ac_Enh	.	.	E086	.	Evi-1, HDAC2, Hoxa9, Hoxd8, Ncx, PPAR, Pax-5, RXRA, TATA
rs3801836	7	26,851,400	Transcript	INTRONIC		1.00	2.00	1.16						43371	65214		.	.	E010,H3K4me1_Enh; E086,H3K4me1_Enh; E088,H3K4me1_Enh; E056,H3K27ac_Enh	.	.	.	.	Cdx, Foxp1, Pdx1
rs3801834	7	26,852,062	Transcript	INTRONIC		0.80	3.96	3.84						42709	65876		E010,7_Enh; E088,7_Enh	.	E002,H3K4me3_Pro; E009,H3K4me1_Enh; E010,H3K4me1_Enh; E084,H3K4me1_Enh; E086,H3K4me1_Enh; E088,H3K4me1_Enh; E103,H3K4me1_Enh; E114,H3K4me1_Enh	.	ESDR, LNG	.	.	BCL, Ik-1, NF-kappaB, STAT, TAL1, p300
rs3801833	7	26,852,212	Transcript	INTRONIC		2.00	1.00	2.00						42559	66026		E010,7_Enh	.	E002,H3K4me3_Pro; E009,H3K4me1_Enh; E010,H3K4me1_Enh; E084,H3K4me1_Enh; E086,H3K4me1_Enh; E088,H3K4me1_Enh; E103,H3K4me1_Enh; E114,H3K4me1_Enh	.	ESDR	.	.	.
rs3801832	7	26,852,280	Transcript	INTRONIC		2.00	2.00	3.00	1	-0.23				42491	66094		E010,7_Enh	.	E002,H3K4me3_Pro; E009,H3K4me1_Enh; E010,H3K4me1_Enh; E084,H3K4me1_Enh; E086,H3K4me1_Enh; E088,H3K4me1_Enh; E103,H3K4me1_Enh; E110,H3K4me1_Enh; E114,H3K4me1_Enh	.	ESDR	.	.	Foxa, Irf, Pou2f2, Pou3f2, Pou3f3
rs7802715	7	26,852,720	Transcript	INTRONIC		2.00	1.00	2.00						42051	66534		E010,7_Enh; E088,7_Enh	.	E009,H3K4me1_Enh; E010,H3K4me1_Enh; E023,H3K4me1_Enh; E025,H3K4me1_Enh; E078,H3K4me1_Enh; E084,H3K4me1_Enh; E086,H3K4me1_Enh; E088,H3K4me1_Enh; E092,H3K4me1_Enh; E103,H3K4me1_Enh; E110,H3K4me1_Enh; E114,H3K4me1_Enh; E121,H3K4me1_Enh	.	ESDR, LNG	.	.	Pax-4
rs7785815	7	26,852,728	Transcript	INTRONIC	0.64	2.00	1.00	2.00						42043	66542		E010,7_Enh; E088,7_Enh	.	E009,H3K4me1_Enh; E010,H3K4me1_Enh; E023,H3K4me1_Enh; E025,H3K4me1_Enh; E078,H3K4me1_Enh; E084,H3K4me1_Enh; E086,H3K4me1_Enh; E088,H3K4me1_Enh; E092,H3K4me1_Enh; E103,H3K4me1_Enh; E110,H3K4me1_Enh; E114,H3K4me1_Enh; E121,H3K4me1_Enh	.	ESDR, LNG	.	.	HMG-IY, Pax-4
rs114978183	7	26,852,740	Transcript	INTRONIC		2.00	1.00	2.00						42031	66554		E010,7_Enh; E088,7_Enh	.	E009,H3K4me1_Enh; E010,H3K4me1_Enh; E023,H3K4me1_Enh; E025,H3K4me1_Enh; E078,H3K4me1_Enh; E084,H3K4me1_Enh; E086,H3K4me1_Enh; E088,H3K4me1_Enh; E092,H3K4me1_Enh; E103,H3K4me1_Enh; E110,H3K4me1_Enh; E114,H3K4me1_Enh; E121,H3K4me1_Enh	.	ESDR, LNG	.	.	Dbx1, HMG-IY, HNF1, Hoxd8, Mef2, Ncx, Pou2f2, TATA, Zfp105
rs115453073	7	26,852,741	Transcript	INTRONIC		2.00	1.00	2.00						42030	66555		E010,7_Enh; E088,7_Enh	.	E009,H3K4me1_Enh; E010,H3K4me1_Enh; E023,H3K4me1_Enh; E025,H3K4me1_Enh; E078,H3K4me1_Enh; E084,H3K4me1_Enh; E086,H3K4me1_Enh; E088,H3K4me1_Enh; E092,H3K4me1_Enh; E103,H3K4me1_Enh; E110,H3K4me1_Enh; E114,H3K4me1_Enh; E121,H3K4me1_Enh	.	ESDR, LNG	.	.	Dbx1, Dbx2, HNF1, Hoxd8, Ncx, PLZF, Pou2f2, Pou3f2, Sox, TATA
rs7785832	7	26,852,760	Transcript	INTRONIC		2.00	0.32	2.00						42011	66574		E010,7_Enh; E088,7_Enh	.	E009,H3K4me1_Enh; E010,H3K4me1_Enh; E023,H3K4me1_Enh; E025,H3K4me1_Enh; E027,H3K4me1_Enh; E063,H3K4me1_Enh; E078,H3K4me1_Enh; E084,H3K4me1_Enh; E086,H3K4me1_Enh; E088,H3K4me1_Enh; E092,H3K4me1_Enh; E103,H3K4me1_Enh; E110,H3K4me1_Enh; E114,H3K4me1_Enh; E121,H3K4me1_Enh	.	ESDR, LNG	.	.	ELF1, HMG-IY, HNF1, NF-AT, SPIB
rs17315334	7	26,852,860	Transcript	INTRONIC		2.00	2.00	2.76	1	0.50				41911	66674		E009,7_Enh; E010,7_Enh; E061,7_Enh; E084,7_Enh; E088,7_Enh; E118,7_Enh	E010,18_EnhAc	E009,H3K4me1_Enh; E010,H3K4me1_Enh; E023,H3K4me1_Enh; E025,H3K4me1_Enh; E027,H3K4me1_Enh; E061,H3K4me1_Enh; E063,H3K4me1_Enh; E078,H3K4me1_Enh; E084,H3K4me1_Enh; E086,H3K4me1_Enh; E088,H3K4me1_Enh; E092,H3K4me1_Enh; E103,H3K4me1_Enh; E110,H3K4me1_Enh; E114,H3K4me1_Enh; E118,H3K4me1_Enh; E121,H3K4me1_Enh; E129,H3K4me1_Enh; E012,H3K27ac_Enh	.	ESDR, SKIN, GI, LNG, LIV	.	.	Foxa, RFX5
rs73067456	7	26,852,998	Transcript	INTRONIC		2.96	6.88	4.08			1	1	38.8700	41773	66812		E009,7_Enh; E010,7_Enh; E061,7_Enh; E084,7_Enh; E088,7_Enh; E118,7_Enh	E010,18_EnhAc	E009,H3K4me1_Enh; E010,H3K4me1_Enh; E023,H3K4me1_Enh; E025,H3K4me1_Enh; E027,H3K4me1_Enh; E061,H3K4me1_Enh; E063,H3K4me1_Enh; E078,H3K4me1_Enh; E084,H3K4me1_Enh; E086,H3K4me1_Enh; E088,H3K4me1_Enh; E092,H3K4me1_Enh; E103,H3K4me1_Enh; E110,H3K4me1_Enh; E114,H3K4me1_Enh; E118,H3K4me1_Enh; E121,H3K4me1_Enh; E129,H3K4me1_Enh; E012,H3K27ac_Enh; E061,H3K27ac_Enh; E114,H3K4me3_Pro	.	ESDR, SKIN, GI, LNG, LIV	E059	.	HMG-IY, Mef2, TATA, YY1
rs3801831	7	26,853,470	RegulatoryFeature	REGULATORY		4.52	7.00	2.12			1	1	38.8700	41301	67284	DNase1, Open Chromatin, DNase1 Hypersensitive Site	E009,7_Enh; E010,7_Enh; E023,7_Enh; E025,7_Enh; E027,7_Enh; E061,7_Enh; E070,7_Enh; E078,7_Enh; E084,7_Enh; E088,7_Enh; E109,7_Enh; E110,7_Enh; E114,7_Enh	E009,19_DNase; E010,18_EnhAc; E023,16_EnhW1; E025,19_DNase; E063,16_EnhW1; E081,17_EnhW2; E086,17_EnhW2; E088,16_EnhW1; E121,22_PromP	E009,H3K4me1_Enh; E010,H3K4me1_Enh; E023,H3K4me1_Enh; E025,H3K4me1_Enh; E027,H3K4me1_Enh; E061,H3K4me1_Enh; E063,H3K4me1_Enh; E078,H3K4me1_Enh; E081,H3K4me1_Enh; E084,H3K4me1_Enh; E086,H3K4me1_Enh; E088,H3K4me1_Enh; E092,H3K4me1_Enh; E097,H3K4me1_Enh; E103,H3K4me1_Enh; E110,H3K4me1_Enh; E114,H3K4me1_Enh; E118,H3K4me1_Enh; E121,H3K4me1_Enh; E129,H3K4me1_Enh; E012,H3K27ac_Enh; E061,H3K27ac_Enh; E063,H3K27ac_Enh; E084,H3K27ac_Enh; E114,H3K4me3_Pro; E118,H3K4me3_Pro	.	ESDR, FAT, BRST, SKIN, BRN, GI, LNG	E059;E088;E121;E125	.	HDAC2, Hoxa3, Myc
rs3823938	7	26,853,741	Transcript	INTRONIC		2.00	2.68	1.00	2	0.25				41030	67555		E009,7_Enh; E010,7_Enh; E023,7_Enh; E025,7_Enh; E061,7_Enh; E078,7_Enh; E088,7_Enh; E109,7_Enh; E110,7_Enh; E114,7_Enh	E010,18_EnhAc; E086,17_EnhW2	E009,H3K4me1_Enh; E010,H3K4me1_Enh; E023,H3K4me1_Enh; E025,H3K4me1_Enh; E027,H3K4me1_Enh; E061,H3K4me1_Enh; E063,H3K4me1_Enh; E078,H3K4me1_Enh; E081,H3K4me1_Enh; E084,H3K4me1_Enh; E086,H3K4me1_Enh; E088,H3K4me1_Enh; E092,H3K4me1_Enh; E103,H3K4me1_Enh; E110,H3K4me1_Enh; E114,H3K4me1_Enh; E121,H3K4me1_Enh; E129,H3K4me1_Enh; E012,H3K27ac_Enh; E061,H3K27ac_Enh; E084,H3K27ac_Enh; E114,H3K4me3_Pro	.	ESDR, FAT, SKIN, GI, LNG	E088	.	Foxf1, Foxp1, Hoxa13, Hoxa9, TATA
rs3823936	7	26,853,830	Transcript	INTRONIC		2.00	7.44	2.00	1	0.15				40941	67644		E009,7_Enh; E010,7_Enh; E025,7_Enh; E078,7_Enh; E088,7_Enh; E109,7_Enh; E110,7_Enh; E114,7_Enh	E009,17_EnhW2; E010,14_EnhA2; E086,17_EnhW2	E009,H3K4me1_Enh; E010,H3K4me1_Enh; E023,H3K4me1_Enh; E025,H3K4me1_Enh; E027,H3K4me1_Enh; E061,H3K4me1_Enh; E063,H3K4me1_Enh; E078,H3K4me1_Enh; E081,H3K4me1_Enh; E084,H3K4me1_Enh; E086,H3K4me1_Enh; E088,H3K4me1_Enh; E092,H3K4me1_Enh; E103,H3K4me1_Enh; E110,H3K4me1_Enh; E114,H3K4me1_Enh; E121,H3K4me1_Enh; E129,H3K4me1_Enh; E012,H3K27ac_Enh; E061,H3K27ac_Enh; E084,H3K27ac_Enh; E114,H3K4me3_Pro	.	ESDR, FAT, GI, LNG	E088	.	Dbx1, PLZF, Pou2f2, Pou6f1, Sox
rs3823934	7	26,853,934	Transcript	INTRONIC		2.00	8.24	2.00						40837	67748		E009,7_Enh; E010,7_Enh; E025,7_Enh; E078,7_Enh; E088,7_Enh; E109,7_Enh; E110,7_Enh; E114,7_Enh	E009,17_EnhW2; E010,14_EnhA2; E086,17_EnhW2	E009,H3K4me1_Enh; E010,H3K4me1_Enh; E023,H3K4me1_Enh; E025,H3K4me1_Enh; E027,H3K4me1_Enh; E061,H3K4me1_Enh; E063,H3K4me1_Enh; E078,H3K4me1_Enh; E081,H3K4me1_Enh; E084,H3K4me1_Enh; E086,H3K4me1_Enh; E088,H3K4me1_Enh; E092,H3K4me1_Enh; E103,H3K4me1_Enh; E110,H3K4me1_Enh; E114,H3K4me1_Enh; E121,H3K4me1_Enh; E129,H3K4me1_Enh; E012,H3K27ac_Enh; E061,H3K27ac_Enh; E084,H3K27ac_Enh; E114,H3K4me3_Pro	.	ESDR, FAT, GI, LNG	E081;E088;E120;E121	.	HNF1, Hmbox1
rs3823933	7	26,853,966	Transcript	INTRONIC		2.00	9.00	2.00						40805	67780		E009,7_Enh; E010,7_Enh; E025,7_Enh; E078,7_Enh; E088,7_Enh; E109,7_Enh; E110,7_Enh; E114,7_Enh	E009,17_EnhW2; E010,14_EnhA2; E086,17_EnhW2	E009,H3K4me1_Enh; E010,H3K4me1_Enh; E023,H3K4me1_Enh; E025,H3K4me1_Enh; E027,H3K4me1_Enh; E061,H3K4me1_Enh; E063,H3K4me1_Enh; E078,H3K4me1_Enh; E081,H3K4me1_Enh; E084,H3K4me1_Enh; E086,H3K4me1_Enh; E088,H3K4me1_Enh; E092,H3K4me1_Enh; E103,H3K4me1_Enh; E110,H3K4me1_Enh; E114,H3K4me1_Enh; E121,H3K4me1_Enh; E129,H3K4me1_Enh; E012,H3K27ac_Enh; E084,H3K27ac_Enh; E114,H3K4me3_Pro	.	ESDR, FAT, GI, LNG	E081;E088;E120;E121	.	AP-1, Gfi1, Myf, TBX5, Tgif1
rs3823932	7	26,853,980	Transcript	INTRONIC		2.00	6.04	2.00						40791	67794		E009,7_Enh; E010,7_Enh; E025,7_Enh; E078,7_Enh; E088,7_Enh; E109,7_Enh; E110,7_Enh; E114,7_Enh	E009,17_EnhW2; E010,14_EnhA2; E086,17_EnhW2	E009,H3K4me1_Enh; E010,H3K4me1_Enh; E023,H3K4me1_Enh; E025,H3K4me1_Enh; E027,H3K4me1_Enh; E061,H3K4me1_Enh; E063,H3K4me1_Enh; E078,H3K4me1_Enh; E081,H3K4me1_Enh; E084,H3K4me1_Enh; E086,H3K4me1_Enh; E088,H3K4me1_Enh; E092,H3K4me1_Enh; E103,H3K4me1_Enh; E110,H3K4me1_Enh; E114,H3K4me1_Enh; E121,H3K4me1_Enh; E129,H3K4me1_Enh; E012,H3K27ac_Enh; E084,H3K27ac_Enh; E114,H3K4me3_Pro	.	ESDR, FAT, GI, LNG	E081;E088;E120	.	.
rs3823931	7	26,854,026	Transcript	INTRONIC		1.44	4.44	2.00						40745	67840		E009,7_Enh; E010,7_Enh; E025,7_Enh; E078,7_Enh; E088,7_Enh; E114,7_Enh	E009,17_EnhW2; E010,18_EnhAc	E009,H3K4me1_Enh; E010,H3K4me1_Enh; E023,H3K4me1_Enh; E025,H3K4me1_Enh; E061,H3K4me1_Enh; E078,H3K4me1_Enh; E081,H3K4me1_Enh; E084,H3K4me1_Enh; E086,H3K4me1_Enh; E088,H3K4me1_Enh; E092,H3K4me1_Enh; E103,H3K4me1_Enh; E110,H3K4me1_Enh; E114,H3K4me1_Enh; E121,H3K4me1_Enh; E129,H3K4me1_Enh; E012,H3K27ac_Enh; E084,H3K27ac_Enh; E114,H3K4me3_Pro	.	ESDR, FAT, GI, LNG	E088;E120	.	Gfi1, Hdx, Mef2, PLZF
rs10262414	7	26,854,266	Transcript	INTRONIC		4.00	4.28	2.92						40505	68080		.	.	E009,H3K4me1_Enh; E010,H3K4me1_Enh; E023,H3K4me1_Enh; E025,H3K4me1_Enh; E061,H3K4me1_Enh; E078,H3K4me1_Enh; E081,H3K4me1_Enh; E084,H3K4me1_Enh; E088,H3K4me1_Enh; E110,H3K4me1_Enh; E114,H3K4me1_Enh; E121,H3K4me1_Enh; E012,H3K27ac_Enh; E084,H3K27ac_Enh	.	.	.	.	.
rs12532199	7	26,854,375	Transcript	INTRONIC		1.68	3.08	3.96						40396	68189		E009,7_Enh; E010,7_Enh; E078,7_Enh	E009,17_EnhW2; E010,18_EnhAc	E009,H3K4me1_Enh; E010,H3K4me1_Enh; E023,H3K4me1_Enh; E025,H3K4me1_Enh; E061,H3K4me1_Enh; E078,H3K4me1_Enh; E081,H3K4me1_Enh; E084,H3K4me1_Enh; E088,H3K4me1_Enh; E110,H3K4me1_Enh; E114,H3K4me1_Enh; E121,H3K4me1_Enh; E012,H3K27ac_Enh; E084,H3K27ac_Enh	.	ESDR, GI	.	.	Cdc5, HP1-site-factor, Nkx3
rs12537323	7	26,854,388	Transcript	INTRONIC		1.00	3.00	3.00						40383	68202		E009,7_Enh; E010,7_Enh; E078,7_Enh	E009,17_EnhW2; E010,18_EnhAc	E009,H3K4me1_Enh; E010,H3K4me1_Enh; E023,H3K4me1_Enh; E025,H3K4me1_Enh; E061,H3K4me1_Enh; E078,H3K4me1_Enh; E081,H3K4me1_Enh; E084,H3K4me1_Enh; E088,H3K4me1_Enh; E110,H3K4me1_Enh; E114,H3K4me1_Enh; E121,H3K4me1_Enh; E012,H3K27ac_Enh; E084,H3K27ac_Enh	.	ESDR, GI	.	.	.
rs28617235	7	26,854,671	Transcript	INTRONIC		2.00	1.80	3.00						40100	68485		E010,7_Enh; E078,7_Enh	.	E009,H3K4me1_Enh; E010,H3K4me1_Enh; E023,H3K4me1_Enh; E025,H3K4me1_Enh; E061,H3K4me1_Enh; E078,H3K4me1_Enh; E081,H3K4me1_Enh; E084,H3K4me1_Enh; E088,H3K4me1_Enh; E110,H3K4me1_Enh; E114,H3K4me1_Enh; E121,H3K4me1_Enh; E012,H3K27ac_Enh; E084,H3K27ac_Enh	.	ESDR, GI	.	.	Foxl1, Hoxb6
rs12532796	7	26,854,828	Transcript	INTRONIC		1.00	2.60	3.00						39943	68642		E010,7_Enh; E078,7_Enh	.	E009,H3K4me1_Enh; E010,H3K4me1_Enh; E023,H3K4me1_Enh; E025,H3K4me1_Enh; E027,H3K4me1_Enh; E061,H3K4me1_Enh; E078,H3K4me1_Enh; E081,H3K4me1_Enh; E084,H3K4me1_Enh; E088,H3K4me1_Enh; E110,H3K4me1_Enh; E114,H3K4me1_Enh; E121,H3K4me1_Enh; E012,H3K27ac_Enh; E084,H3K27ac_Enh	.	ESDR, GI	E088	.	AFP1, PLZF
rs3801830	7	26,854,870	Transcript	INTRONIC		1.00	2.36	3.00						39901	68684		E010,7_Enh; E078,7_Enh	.	E009,H3K4me1_Enh; E010,H3K4me1_Enh; E023,H3K4me1_Enh; E025,H3K4me1_Enh; E027,H3K4me1_Enh; E061,H3K4me1_Enh; E078,H3K4me1_Enh; E081,H3K4me1_Enh; E084,H3K4me1_Enh; E088,H3K4me1_Enh; E110,H3K4me1_Enh; E114,H3K4me1_Enh; E121,H3K4me1_Enh; E012,H3K27ac_Enh; E084,H3K27ac_Enh	.	ESDR, GI	.	.	AP-1, Myc, Pou2f2
rs3801829	7	26,854,996	Transcript	INTRONIC		2.00	2.00	3.80						39775	68810		E010,7_Enh; E078,7_Enh	.	E009,H3K4me1_Enh; E010,H3K4me1_Enh; E023,H3K4me1_Enh; E025,H3K4me1_Enh; E027,H3K4me1_Enh; E061,H3K4me1_Enh; E078,H3K4me1_Enh; E081,H3K4me1_Enh; E084,H3K4me1_Enh; E088,H3K4me1_Enh; E110,H3K4me1_Enh; E114,H3K4me1_Enh; E121,H3K4me1_Enh; E012,H3K27ac_Enh; E084,H3K27ac_Enh	.	ESDR, GI	.	.	Fox, Foxj1
rs10266759	7	26,855,282	Transcript	INTRONIC		4.00	4.84	3.08						39489	69096		.	.	E009,H3K4me1_Enh; E010,H3K4me1_Enh; E023,H3K4me1_Enh; E025,H3K4me1_Enh; E027,H3K4me1_Enh; E061,H3K4me1_Enh; E078,H3K4me1_Enh; E081,H3K4me1_Enh; E084,H3K4me1_Enh; E088,H3K4me1_Enh; E110,H3K4me1_Enh; E114,H3K4me1_Enh; E121,H3K4me1_Enh; E012,H3K27ac_Enh; E084,H3K27ac_Enh	.	.	E081;E082	P300	NF-AT, NF-AT1, SP1
rs3801828	7	26,855,284	Transcript	INTRONIC		4.00	4.84	3.08						39487	69098		E010,7_Enh; E070,7_Enh; E078,7_Enh	E010,19_DNase; E070,19_DNase	E009,H3K4me1_Enh; E010,H3K4me1_Enh; E023,H3K4me1_Enh; E025,H3K4me1_Enh; E027,H3K4me1_Enh; E061,H3K4me1_Enh; E078,H3K4me1_Enh; E081,H3K4me1_Enh; E084,H3K4me1_Enh; E088,H3K4me1_Enh; E110,H3K4me1_Enh; E114,H3K4me1_Enh; E121,H3K4me1_Enh; E012,H3K27ac_Enh; E084,H3K27ac_Enh	.	ESDR, BRN, GI	E081;E082	P300	SETDB1, SP1
rs6948584	7	26,855,732	Transcript	INTRONIC	0.64	4.72	4.00	6.00						39039	69546		E010,7_Enh; E025,7_Enh	E023,17_EnhW2	E010,H3K4me1_Enh; E023,H3K4me1_Enh; E025,H3K4me1_Enh; E027,H3K4me1_Enh; E063,H3K4me1_Enh; E078,H3K4me1_Enh; E081,H3K4me1_Enh; E084,H3K4me1_Enh; E088,H3K4me1_Enh; E110,H3K4me1_Enh; E047,H3K9ac_Pro	.	ESDR, FAT	E032;E086;E088	.	Foxj1, Foxo, PLZF, Sox
rs6948657	7	26,855,943	Transcript	INTRONIC		3.00	3.00	2.00						38828	69757		E025,7_Enh	E023,17_EnhW2	E010,H3K4me1_Enh; E023,H3K4me1_Enh; E025,H3K4me1_Enh; E063,H3K4me1_Enh; E078,H3K4me1_Enh; E081,H3K4me1_Enh; E084,H3K4me1_Enh; E088,H3K4me1_Enh; E110,H3K4me1_Enh; E047,H3K9ac_Pro	.	FAT	E089	.	Barhl1, Bsx, Esx1, Isx, Msx-1, Msx2, Nkx1-1, Nkx2, Nobox, Pax7, Phox2a, Vax2
rs73067472	7	26,855,959	Transcript	INTRONIC		2.00	3.00	2.88						38812	69773		E025,7_Enh	E023,17_EnhW2	E010,H3K4me1_Enh; E023,H3K4me1_Enh; E025,H3K4me1_Enh; E063,H3K4me1_Enh; E078,H3K4me1_Enh; E084,H3K4me1_Enh; E088,H3K4me1_Enh; E110,H3K4me1_Enh; E047,H3K9ac_Pro	.	FAT	E089	.	.
rs3823930	7	26,856,525	Transcript	INTRONIC		3.00	6.00	5.28						38246	70339		E023,7_Enh	.	E010,H3K4me1_Enh; E023,H3K4me1_Enh; E025,H3K4me1_Enh; E078,H3K4me1_Enh; E084,H3K4me1_Enh; E088,H3K4me1_Enh; E110,H3K4me1_Enh; E047,H3K9ac_Pro; E110,H3K9ac_Pro	.	FAT	.	.	Barx1, Barx2, Dbx2, Hoxa10, Hoxc6, Hoxc9, Hoxd8, Lhx3, Maf, Msx-1, Ncx, Nkx2, Nkx6-1, Nobox, YY1
rs3823929	7	26,856,600	Transcript	INTRONIC	0.64	2.00	5.60	5.76						38171	70414		E023,7_Enh	.	E010,H3K4me1_Enh; E023,H3K4me1_Enh; E025,H3K4me1_Enh; E078,H3K4me1_Enh; E084,H3K4me1_Enh; E088,H3K4me1_Enh; E110,H3K4me1_Enh; E047,H3K9ac_Pro; E110,H3K9ac_Pro	.	FAT	.	.	Gsc, Lhx3, Obox6, Otx2
rs3801827	7	26,856,626	Transcript	INTRONIC		1.64	6.00	4.92						38145	70440		.	.	E010,H3K4me1_Enh; E023,H3K4me1_Enh; E025,H3K4me1_Enh; E078,H3K4me1_Enh; E084,H3K4me1_Enh; E088,H3K4me1_Enh; E110,H3K4me1_Enh; E047,H3K9ac_Pro; E110,H3K9ac_Pro	.	.	.	.	Osf2, RREB-1
rs3801826	7	26,856,720	Transcript	INTRONIC	0.64	2.00	3.00	2.84						38051	70534		.	.	E010,H3K4me1_Enh; E023,H3K4me1_Enh; E025,H3K4me1_Enh; E078,H3K4me1_Enh; E084,H3K4me1_Enh; E088,H3K4me1_Enh; E110,H3K4me1_Enh; E047,H3K9ac_Pro; E110,H3K9ac_Pro	.	.	.	.	CEBPB, Maf, Pou5f1, p300
rs3801825	7	26,856,959	Transcript	INTRONIC		2.00	1.00	2.00	1	-1.00				37812	70773		.	.	E010,H3K4me1_Enh; E023,H3K4me1_Enh; E088,H3K4me1_Enh; E110,H3K4me1_Enh; E047,H3K9ac_Pro	.	.	.	.	Dobox4, Mef2, NF-kappaB, Pou1f1, Pou3f1
rs73067475	7	26,857,120	Transcript	INTRONIC		1.00	3.00	2.00						37651	70934		.	.	E010,H3K4me1_Enh; E047,H3K9ac_Pro	.	.	.	.	Brachyury, FAC1, TBX5
rs73067476	7	26,857,240	Transcript	INTRONIC		1.00	2.00	3.00	1	0.52				37531	71054		.	.	E047,H3K9ac_Pro	.	.	.	.	Nrf-2, Pax-2, ZEB1, p300
rs115447617	7	26,857,751	Transcript	INTRONIC		1.00		1.40						37020	71565		.	.	.	.	.	.	.	ERalpha-a, Pax-4, STAT
rs111373218	7	26,859,152	Transcript	INTRONIC		1.00	2.80	1.00						35619	72966		.	.	.	.	.	.	.	.
rs17315775	7	26,860,134	Transcript	INTRONIC		1.04	3.40	2.00	1	0.46				34637	73948		.	.	E047,H3K9ac_Pro	.	.	.	.	Dmbx1, HNF4, Pou6f1
rs28404962	7	26,860,324	Transcript	INTRONIC		2.00	1.00	2.44						34447	74138	H3K36me3, Histone, Histone 3 Lysine 36 Tri-Methylation	.	.	E047,H3K9ac_Pro	.	.	.	.	Arid5b, Cdx2, Foxp1, HNF1, Hoxc10, Lhx3, Ncx, Pou2f2
rs13438513	7	26,860,633	Transcript	INTRONIC		1.44	3.00	3.00						34138	74447	H3K9me1, Histone, Histone 3 Lysine 9 mono-methylation H3K36me3, Histone, Histone 3 Lysine 36 Tri-Methylation	.	.	.	.	.	.	.	Mef2
rs13438514	7	26,860,688	Transcript	INTRONIC		2.08	3.00	3.00						34083	74502	H3K9me1, Histone, Histone 3 Lysine 9 mono-methylation H3K36me3, Histone, Histone 3 Lysine 36 Tri-Methylation	.	.	.	.	.	.	.	Hoxc10
rs55739485	7	26,860,741	Transcript	INTRONIC		3.00	3.00	2.00	1	0.25				34030	74555	H3K36me3, Histone, Histone 3 Lysine 36 Tri-Methylation	.	.	.	.	.	.	.	GATA, Irf, Mef2, TATA
rs3801823	7	26,861,031	Transcript	INTRONIC	0.44	1.16	1.00	1.00						33740	74845	H3K36me3, Histone, Histone 3 Lysine 36 Tri-Methylation	.	E047,17_EnhW2	E047,H3K4me3_Pro	.	.	.	.	Gfi1, PLZF
rs3801822	7	26,861,201	Transcript	INTRONIC		3.00	3.32	2.00						33570	75015	H3K36me3, Histone, Histone 3 Lysine 36 Tri-Methylation	.	E047,17_EnhW2	E047,H3K4me3_Pro	.	.	.	.	Bbx, Dbx1, Gfi1, Ncx
rs3801821	7	26,861,206	Transcript	INTRONIC		3.00	3.32	2.00						33565	75020	H3K36me3, Histone, Histone 3 Lysine 36 Tri-Methylation	.	E047,17_EnhW2	E047,H3K4me3_Pro	.	.	.	.	.
rs3801820	7	26,861,353	Transcript	INTRONIC	0.64	3.00	4.08	2.00						33418	75167	H3K36me3, Histone, Histone 3 Lysine 36 Tri-Methylation	.	E047,17_EnhW2	E047,H3K4me3_Pro; E065,H3K4me1_Enh	.	.	.	.	Brachyury, Foxa, Mrg1::Hoxa9, Nkx3
rs7785689	7	26,861,609	Transcript	INTRONIC		3.88	2.04	2.00						33162	75423	H3K36me3, Histone, Histone 3 Lysine 36 Tri-Methylation	.	.	E047,H3K4me3_Pro	.	.	.	.	Barhl1, Barx2, Dbx1, Dbx2, En-1, Esx1, GATA, HNF1, Hoxa3, Hoxb4, Hoxd8, Msx-1, Pax-6, Pou2f2, Pou3f2, Prrx1
rs7785711	7	26,861,640	Transcript	INTRONIC		4.00	3.00	2.00						33131	75454	H3K36me3, Histone, Histone 3 Lysine 36 Tri-Methylation	.	.	E047,H3K4me3_Pro	.	.	.	.	Dbx1, Foxa, Foxd3, Foxj2, Foxk1, Foxl1, Foxp1, HDAC2, Hoxd8, Ncx, Nkx3, Pou2f2
rs7785798	7	26,861,943	Transcript	INTRONIC		2.00	1.00	1.00						32828	75757	H3K36me3, Histone, Histone 3 Lysine 36 Tri-Methylation	.	.	E047,H3K4me3_Pro	.	.	.	.	Myc, Pax-4
rs7785918	7	26,861,959	Transcript	INTRONIC	0.64	2.00	1.00	1.00						32812	75773	H3K36me3, Histone, Histone 3 Lysine 36 Tri-Methylation	.	.	E047,H3K4me3_Pro	.	.	.	.	Arid5a, DMRT4, HNF1, Pou2f2, Sox

rs12540045	7	26,863,174	Transcript	INTRONIC		4.00	3.00	2.56						31597	76988	H3K36me3, Histone, Histone 3 Lysine 36 Tri-Methylation	.	.	.	.	.	.	.	.
rs10248500	7	26,863,294	Transcript	INTRONIC	0.89	3.00	2.00	4.00						31477	77108	H3K36me3, Histone, Histone 3 Lysine 36 Tri-Methylation	.	.	.	.	.	.	.	Irf, PRDM1, Pou5f1, Sox, Zfp105
rs17315929	7	26,864,173	Transcript	INTRONIC		1.00	2.00	1.00						30598	77987	H3K36me3, Histone, Histone 3 Lysine 36 Tri-Methylation	.	.	E027,H3K9ac_Pro; E088,H3K4me1_Enh	.	.	.	.	GR, HNF4, PPAR, RXRA, Sox, TCF4, Zbtb12
rs10227673	7	26,865,299	Transcript	INTRONIC		2.00		2.72						29472	79113		.	.	E027,H3K9ac_Pro; E088,H3K4me1_Enh; E110,H3K4me1_Enh	.	.	.	.	Cdx2, Dbx1, Foxp1, HNF1, Hoxa10, Hoxa9, Hoxb13, Hoxb9, Hoxd10, Irf, Ncx, PLZF, Pax-4, Pou2f2, Sox, Zfp105
rs7805766	7	26,865,610	Transcript	INTRONIC	2.54	2.00	1.92	4.40						29161	79424		.	.	E027,H3K9ac_Pro; E088,H3K4me1_Enh; E110,H3K4me1_Enh	.	.	E003	.	AP-1, Cdx, Nkx2
rs17375272	7	26,866,497	Transcript	INTRONIC		1.00	2.00	2.28						28274	80311		.	.	E027,H3K9ac_Pro	.	.	.	.	.
rs3801819	7	26,866,698	Transcript	INTRONIC		2.00	1.00	3.00						28073	80512		.	.	E027,H3K9ac_Pro	.	.	.	.	DMRT1, DMRT2, DMRT3, DMRT4, DMRT5, DMRT7, Mef2, STAT
rs61354389	7	26,868,343	Transcript	INTRONIC	1.91	1.20	2.00	1.08						26428	82157	H3K36me3, Histone, Histone 3 Lysine 36 Tri-Methylation	.	.	.	.	.	.	.	E4F1, XBP-1, p53
rs10225700	7	26,869,880	Transcript	INTRONIC		3.00	1.00	2.00						24891	83694	H3K36me3, Histone, Histone 3 Lysine 36 Tri-Methylation	.	.	.	.	.	.	.	AP-3, Irf, Pax-5
rs10241234	7	26,869,887	Transcript	INTRONIC		3.00	1.00	2.00						24884	83701	H3K36me3, Histone, Histone 3 Lysine 36 Tri-Methylation	.	.	.	.	.	.	.	Irf
rs10225904	7	26,869,888	Transcript	INTRONIC		3.00	1.00	2.00						24883	83702	H3K36me3, Histone, Histone 3 Lysine 36 Tri-Methylation	.	.	.	.	.	.	.	.
rs28550029	7	26,872,581	Transcript	INTRONIC	1.91	2.00	8.00	2.32						22190	86395	H3K36me3, Histone, Histone 3 Lysine 36 Tri-Methylation	E027,7_Enh; E028,7_Enh; E057,7_Enh; E079,7_Enh; E127,7_Enh	.	E002,H3K4me3_Pro; E027,H3K4me1_Enh; E028,H3K4me1_Enh; E057,H3K4me1_Enh; E058,H3K4me1_Enh; E079,H3K4me1_Enh; E119,H3K4me1_Enh; E127,H3K4me1_Enh	.	BRST, SKIN, GI	.	.	PLZF
rs114236648	7	26,874,639	Transcript	INTRONIC		1.00	1.00	1.00						20132	88453		.	.	.	.	.	.	.	EBF
rs12534899	7	26,877,189	Transcript	INTRONIC		3.00	3.72	2.12						17582	91003	H3K36me3, Histone, Histone 3 Lysine 36 Tri-Methylation	.	.	E088,H3K4me3_Pro	.	.	.	.	Smad, TLX1::NFIC
rs12534921	7	26,877,210	Transcript	INTRONIC		3.00	3.00	3.56						17561	91024	H3K36me3, Histone, Histone 3 Lysine 36 Tri-Methylation	.	.	E088,H3K4me3_Pro	.	.	.	.	YY1, Znf143
rs73069507	7	26,877,536	Transcript	INTRONIC		3.00	2.00	4.00						17235	91350	H3K36me3, Histone, Histone 3 Lysine 36 Tri-Methylation	E112,7_Enh	.	E007,H3K9ac_Pro; E033,H3K4me1_Enh	.	THYM	.	.	.
rs73069510	7	26,877,567	Transcript	INTRONIC		3.00	2.00	2.72						17204	91381	H3K36me3, Histone, Histone 3 Lysine 36 Tri-Methylation	E112,7_Enh	.	E007,H3K9ac_Pro; E033,H3K4me1_Enh	.	THYM	.	.	E2F, HDAC2, HNF4, Irf, Pax-5, Pou2f2, Pou3f1, RXRA
rs3801816	7	26,881,177	Transcript	INTRONIC		3.76	2.76	2.00						13594	94991	H3K36me3, Histone, Histone 3 Lysine 36 Tri-Methylation	.	.	E012,H3K27ac_Enh; E025,H3K4me1_Enh; E061,H3K4me1_Enh; E063,H3K4me1_Enh; E077,H3K4me1_Enh; E084,H3K4me1_Enh; E087,H3K4me1_Enh; E102,H3K4me1_Enh; E110,H3K4me1_Enh; E025,H3K9ac_Pro; E066,H3K9ac_Pro; E102,H3K9ac_Pro; E106,H3K4me3_Pro	.	.	.	.	Pou3f2, STAT
rs3801815	7	26,881,292	Transcript	INTRONIC		4.00	2.68	2.00	1	0.12				13479	95106	H3K36me3, Histone, Histone 3 Lysine 36 Tri-Methylation	.	.	E025,H3K4me1_Enh; E061,H3K4me1_Enh; E063,H3K4me1_Enh; E084,H3K4me1_Enh; E102,H3K4me1_Enh; E110,H3K4me1_Enh; E025,H3K9ac_Pro; E066,H3K9ac_Pro; E102,H3K9ac_Pro; E106,H3K4me3_Pro	.	.	.	.	BCL, HDAC2, Irf, PU.1, Pax-5, Sox, p300
rs3801814	7	26,881,302	Transcript	INTRONIC		3.92	3.48	3.00	2	-0.28				13469	95116	H3K36me3, Histone, Histone 3 Lysine 36 Tri-Methylation	.	.	E025,H3K4me1_Enh; E061,H3K4me1_Enh; E063,H3K4me1_Enh; E084,H3K4me1_Enh; E102,H3K4me1_Enh; E110,H3K4me1_Enh; E025,H3K9ac_Pro; E066,H3K9ac_Pro; E102,H3K9ac_Pro; E106,H3K4me3_Pro	.	.	.	.	Arid5a, BCL, Fox, Foxa, Foxd3, Foxi1, Foxj1, Foxj2, Foxl1, HDAC2, HNF1, Irf, Nkx6-2, Pax-4, RXRA, Sox, TATA, p300
rs12535784	7	26,882,067	Transcript	INTRONIC		1.00	1.16	1.00						12704	95881	H3K36me3, Histone, Histone 3 Lysine 36 Tri-Methylation	.	.	E023,H3K4me1_Enh; E025,H3K4me1_Enh; E061,H3K4me1_Enh; E025,H3K9ac_Pro; E066,H3K9ac_Pro	.	.	.	.	Hsf
rs4719882	7	26,882,252	Transcript	INTRONIC		3.00	2.00	2.00						12519	96066	H3K36me3, Histone, Histone 3 Lysine 36 Tri-Methylation	.	.	E023,H3K4me1_Enh; E025,H3K4me1_Enh; E061,H3K4me1_Enh; E023,H3K4me3_Pro; E023,H3K9ac_Pro; E025,H3K9ac_Pro; E066,H3K9ac_Pro	.	.	E090	.	Foxp1, Smad
rs1982995	7	26,884,135	Transcript	INTRONIC		4.00	4.00	3.36	1	-0.26				10636	486		E023,7_Enh	E023,17_EnhW2; E025,16_EnhW1	E023,H3K4me1_Enh; E025,H3K4me1_Enh; E050,H3K4me1_Enh; E061,H3K4me1_Enh; E088,H3K4me1_Enh; E023,H3K4me3_Pro; E023,H3K9ac_Pro; E025,H3K9ac_Pro; E063,H3K9ac_Pro	.	FAT	.	.	ELF1, Egr-1, Ets, Foxp1, NF-kappaB, Spdef, TR4, Znf143
rs10236221	7	26,884,671	Transcript	INTRONIC		3.00	8.00	2.00	1	0.10				10100	1022		.	.	E023,H3K4me1_Enh; E025,H3K4me1_Enh; E050,H3K4me1_Enh; E061,H3K4me1_Enh; E063,H3K4me1_Enh; E088,H3K4me1_Enh; E023,H3K9ac_Pro; E025,H3K9ac_Pro; E063,H3K9ac_Pro; E058,H3K27ac_Enh	.	.	E085;E088	.	HDAC2, Pou2f2
rs10270187	7	26,885,885	Transcript	INTRONIC		3.00	4.72	1.00	1	-0.25				8886	2236	H3K36me3, Histone, Histone 3 Lysine 36 Tri-Methylation	.	.	E025,H3K9ac_Pro; E063,H3K9ac_Pro; E124,H3K9ac_Pro; E116,H3K4me1_Enh	.	.	.	.	Fox, Foxa, Irf, Ncx
rs10227529	7	26,886,309	Transcript	INTRONIC		2.00	2.00	4.52						8462	2660	H3K36me3, Histone, Histone 3 Lysine 36 Tri-Methylation	.	.	E116,H3K4me1_Enh; E124,H3K9ac_Pro	.	.	.	.	Arid3a, CEBPG, Dbx1, Foxa, HNF1, Mef2, Ncx, Pou2f2, TATA, Zfp105
rs3801813	7	26,887,069	Transcript	INTRONIC		1.56	2.00	2.00						7702	3420	H3K36me3, Histone, Histone 3 Lysine 36 Tri-Methylation	.	.	E058,H3K27ac_Enh; E116,H3K4me1_Enh	.	.	.	.	AP-2, CACD, CTCF, E2F, EBF, Glis2, MZF1::1-4, Roaz, Zic
rs3801812	7	26,887,615	Transcript	INTRONIC	0.41	3.00	3.00	3.84						7156	3966	H3K36me3, Histone, Histone 3 Lysine 36 Tri-Methylation	.	.	E058,H3K27ac_Enh; E063,H3K27ac_Enh; E116,H3K4me1_Enh	.	.	.	.	Pax-4
rs10272190	7	26,887,942	Transcript	INTRONIC		2.00	4.68	5.00						6829	4293	H3K36me3, Histone, Histone 3 Lysine 36 Tri-Methylation	.	.	E058,H3K27ac_Enh; E063,H3K27ac_Enh	.	.	.	.	Fox, Foxa, Foxd1, RREB-1
rs10227231	7	26,888,074	Transcript	INTRONIC		2.00	2.00	4.00						6697	4425	H3K36me3, Histone, Histone 3 Lysine 36 Tri-Methylation	.	.	E023,H3K9ac_Pro; E058,H3K27ac_Enh; E063,H3K27ac_Enh	.	.	.	.	SIX5
rs10272457	7	26,888,155	Transcript	INTRONIC		3.00	1.00	2.00						6616	4506	H3K36me3, Histone, Histone 3 Lysine 36 Tri-Methylation	.	.	E023,H3K9ac_Pro; E058,H3K27ac_Enh; E063,H3K27ac_Enh	.	.	.	.	GZF1, Mef2, Nanog, Pou1f1, Pou2f2, Pou3f3, Pou5f1, TATA
rs10272603	7	26,888,277	Transcript	INTRONIC		1.00	2.00	1.00						6494	4628	H3K36me3, Histone, Histone 3 Lysine 36 Tri-Methylation	.	.	E023,H3K9ac_Pro; E058,H3K27ac_Enh; E063,H3K27ac_Enh	.	.	.	.	RXRA
rs6461979	7	26,888,918	Transcript	INTRONIC		2.00	2.00	4.12						5853	5269	H3K36me3, Histone, Histone 3 Lysine 36 Tri-Methylation	.	.	E023,H3K9ac_Pro; E025,H3K9ac_Pro; E026,H3K4me1_Enh	.	.	.	.	Foxk1, Mef2, PLZF, Pou2f2, Zfp105
rs17376018	7	26,889,862	Transcript	INTRONIC		14.56	18.52	8.92	1	0.20	1	1	33.0393	4909	6213	H3K36me3, Histone, Histone 3 Lysine 36 Tri-Methylation	E023,7_Enh; E025,7_Enh; E028,6_EnhG; E049,7_Enh; E058,7_Enh; E123,7_Enh	E023,14_EnhA2; E025,14_EnhA2; E026,17_EnhW2; E027,19_DNase; E028,15_EnhAF; E057,17_EnhW2; E058,16_EnhW1; E119,11_TxEnh3; E123,16_EnhW1; E127,17_EnhW2	E023,H3K4me1_Enh; E025,H3K4me1_Enh; E026,H3K4me1_Enh; E028,H3K4me1_Enh; E049,H3K4me1_Enh; E050,H3K4me1_Enh; E058,H3K4me1_Enh; E123,H3K4me1_Enh; E127,H3K4me1_Enh; E023,H3K4me3_Pro; E025,H3K4me3_Pro; E123,H3K4me3_Pro; E023,H3K9ac_Pro; E025,H3K9ac_Pro; E047,H3K9ac_Pro; E049,H3K9ac_Pro; E123,H3K9ac_Pro; E127,H3K9ac_Pro; E058,H3K27ac_Enh; E063,H3K27ac_Enh; E123,H3K27ac_Enh; E127,H3K27ac_Enh	.	FAT, BRST, STRM, SKIN, BLD	E057;E123	.	CEBPB, p300
rs4722647	7	26,890,446	Transcript	INTRONIC		21.56	9.88	9.28			2	2	43.4171	4325	6797	H3K9me1, Histone, Histone 3 Lysine 9 mono-methylation H3K36me3, Histone, Histone 3 Lysine 36 Tri-Methylation	E025,7_Enh; E123,7_Enh; E127,7_Enh	E023,17_EnhW2; E025,14_EnhA2	E023,H3K4me1_Enh; E025,H3K4me1_Enh; E026,H3K4me1_Enh; E028,H3K4me1_Enh; E049,H3K4me1_Enh; E050,H3K4me1_Enh; E052,H3K4me1_Enh; E058,H3K4me1_Enh; E123,H3K4me1_Enh; E127,H3K4me1_Enh; E023,H3K4me3_Pro; E025,H3K4me3_Pro; E123,H3K4me3_Pro; E023,H3K9ac_Pro; E025,H3K9ac_Pro; E049,H3K9ac_Pro; E123,H3K9ac_Pro; E127,H3K9ac_Pro; E037,H3K27ac_Enh; E050,H3K27ac_Enh; E055,H3K27ac_Enh; E058,H3K27ac_Enh; E061,H3K27ac_Enh; E063,H3K27ac_Enh; E127,H3K27ac_Enh	.	FAT, BLD, SKIN	E123	.	CHD2, Pax-4
rs3934268	7	26,890,572	Transcript	INTRONIC		7.64	7.96	1.64			1	1	35.0807	4199	6923	H3K36me3, Histone, Histone 3 Lysine 36 Tri-Methylation	E025,7_Enh; E123,7_Enh; E127,7_Enh	E023,17_EnhW2; E025,14_EnhA2	E023,H3K4me1_Enh; E025,H3K4me1_Enh; E028,H3K4me1_Enh; E049,H3K4me1_Enh; E050,H3K4me1_Enh; E052,H3K4me1_Enh; E058,H3K4me1_Enh; E123,H3K4me1_Enh; E127,H3K4me1_Enh; E023,H3K4me3_Pro; E023,H3K9ac_Pro; E025,H3K9ac_Pro; E049,H3K9ac_Pro; E123,H3K9ac_Pro; E127,H3K9ac_Pro; E050,H3K27ac_Enh; E055,H3K27ac_Enh; E058,H3K27ac_Enh; E063,H3K27ac_Enh; E127,H3K27ac_Enh	.	FAT, BLD, SKIN	.	.	Foxf1, Foxi1, Foxj1, Foxj2, Foxk1, Foxl1, Foxo, Foxq1, HNF1, Irf, Mef2, PLZF, Sox
rs2031811	7	26,890,672	Transcript	INTRONIC		3.96	7.80	2.00						4099	7023	H3K36me3, Histone, Histone 3 Lysine 36 Tri-Methylation	E123,7_Enh; E127,7_Enh	E023,17_EnhW2	E023,H3K4me1_Enh; E025,H3K4me1_Enh; E028,H3K4me1_Enh; E049,H3K4me1_Enh; E050,H3K4me1_Enh; E052,H3K4me1_Enh; E058,H3K4me1_Enh; E123,H3K4me1_Enh; E127,H3K4me1_Enh; E023,H3K4me3_Pro; E023,H3K9ac_Pro; E025,H3K9ac_Pro; E049,H3K9ac_Pro; E123,H3K9ac_Pro; E127,H3K9ac_Pro; E055,H3K27ac_Enh; E058,H3K27ac_Enh; E063,H3K27ac_Enh; E127,H3K27ac_Enh	.	BLD, SKIN	.	.	Foxp1, Irf, Pax-4, Pou2f2, STAT, YY1
rs2030136	7	26,891,016	Transcript	INTRONIC		5.28	5.64	2.00						3755	7367	H3K36me3, Histone, Histone 3 Lysine 36 Tri-Methylation	.	.	E023,H3K4me1_Enh; E025,H3K4me1_Enh; E028,H3K4me1_Enh; E050,H3K4me1_Enh; E123,H3K4me1_Enh; E023,H3K9ac_Pro; E025,H3K9ac_Pro; E049,H3K9ac_Pro; E058,H3K27ac_Enh; E063,H3K27ac_Enh; E127,H3K27ac_Enh	.	.	.	.	GR, STAT, THAP1, YY1
rs7804356	7	26,891,665	Transcript	INTRONIC		4.00	3.00	2.68	1	0.71				3106	8016	H3K36me3, Histone, Histone 3 Lysine 36 Tri-Methylation H3K27me3, Histone, Histone 3 Lysine 27 Tri-Methylation	.	.	E023,H3K9ac_Pro; E025,H3K9ac_Pro; E049,H3K9ac_Pro; E027,H3K4me1_Enh; E058,H3K27ac_Enh; E061,H3K27ac_Enh	.	.	.	.	GR
rs7804620	7	26,891,831	Transcript	INTRONIC		3.96	3.40	1.92						2940	8182	H3K36me3, Histone, Histone 3 Lysine 36 Tri-Methylation H3K27me3, Histone, Histone 3 Lysine 27 Tri-Methylation	.	.	E023,H3K9ac_Pro; E025,H3K9ac_Pro; E049,H3K9ac_Pro; E102,H3K9ac_Pro; E122,H3K9ac_Pro; E027,H3K4me1_Enh; E058,H3K27ac_Enh; E061,H3K27ac_Enh	.	.	.	.	.
rs73069533	7	26,892,325	Transcript	INTRONIC		3.00	2.00	1.00						2446	8676	H3K36me3, Histone, Histone 3 Lysine 36 Tri-Methylation H3K27me3, Histone, Histone 3 Lysine 27 Tri-Methylation	.	.	E023,H3K9ac_Pro; E025,H3K9ac_Pro; E026,H3K9ac_Pro; E049,H3K9ac_Pro; E066,H3K9ac_Pro; E076,H3K9ac_Pro; E088,H3K9ac_Pro; E101,H3K9ac_Pro; E102,H3K9ac_Pro; E119,H3K9ac_Pro; E122,H3K9ac_Pro; E027,H3K4me1_Enh; E028,H3K4me1_Enh; E078,H3K4me1_Enh; E088,H3K4me1_Enh; E101,H3K4me1_Enh; E114,H3K4me1_Enh; E027,H3K4me3_Pro; E058,H3K27ac_Enh; E127,H3K27ac_Enh	.	.	.	.	Ik-2, Irf
rs10486483	7	26,892,440	Transcript	INTRONIC		2.00	4.00	6.00						2331	8791	H3K36me3, Histone, Histone 3 Lysine 36 Tri-Methylation H3K27me3, Histone, Histone 3 Lysine 27 Tri-Methylation	.	.	E023,H3K9ac_Pro; E025,H3K9ac_Pro; E026,H3K9ac_Pro; E049,H3K9ac_Pro; E066,H3K9ac_Pro; E076,H3K9ac_Pro; E088,H3K9ac_Pro; E101,H3K9ac_Pro; E102,H3K9ac_Pro; E119,H3K9ac_Pro; E122,H3K9ac_Pro; E025,H3K4me1_Enh; E027,H3K4me1_Enh; E028,H3K4me1_Enh; E078,H3K4me1_Enh; E088,H3K4me1_Enh; E101,H3K4me1_Enh; E114,H3K4me1_Enh; E027,H3K4me3_Pro; E058,H3K27ac_Enh; E127,H3K27ac_Enh	.	.	.	.	CDP, FXR, Gfi1, Sox
rs3801810	7	26,892,531	Transcript	INTRONIC		2.00	2.96	5.00						2240	8882	H3K36me3, Histone, Histone 3 Lysine 36 Tri-Methylation H3K27me3, Histone, Histone 3 Lysine 27 Tri-Methylation	.	.	E023,H3K9ac_Pro; E025,H3K9ac_Pro; E026,H3K9ac_Pro; E049,H3K9ac_Pro; E066,H3K9ac_Pro; E076,H3K9ac_Pro; E088,H3K9ac_Pro; E101,H3K9ac_Pro; E102,H3K9ac_Pro; E119,H3K9ac_Pro; E122,H3K9ac_Pro; E025,H3K4me1_Enh; E027,H3K4me1_Enh; E028,H3K4me1_Enh; E078,H3K4me1_Enh; E088,H3K4me1_Enh; E101,H3K4me1_Enh; E103,H3K4me1_Enh; E114,H3K4me1_Enh; E027,H3K4me3_Pro; E058,H3K27ac_Enh; E127,H3K27ac_Enh	.	.	.	.	Arid3a, CDP, Cart1, Foxp1, Hoxa5, Hoxb6, Hoxc6, Maf, Nkx6-1, Pou2f2
rs3823928	7	26,893,317	Transcript	INTRONIC		7.00	11.92	4.96	1	1.00				1454	9668	H3K27me3, Histone, Histone 3 Lysine 27 Tri-Methylation	E025,7_Enh; E027,7_Enh; E028,7_Enh; E057,7_Enh; E058,6_EnhG; E114,7_Enh; E119,7_Enh; E127,7_Enh; E128,7_Enh	E023,17_EnhW2; E027,17_EnhW2; E028,15_EnhAF; E057,17_EnhW2; E058,17_EnhW2; E078,22_PromP; E119,15_EnhAF; E127,14_EnhA2	E012,H3K27ac_Enh; E013,H3K27ac_Enh; E050,H3K27ac_Enh; E058,H3K27ac_Enh; E063,H3K27ac_Enh; E076,H3K27ac_Enh; E078,H3K27ac_Enh; E119,H3K27ac_Enh; E127,H3K27ac_Enh; E023,H3K9ac_Pro; E025,H3K9ac_Pro; E026,H3K9ac_Pro; E049,H3K9ac_Pro; E063,H3K9ac_Pro; E066,H3K9ac_Pro; E076,H3K9ac_Pro; E086,H3K9ac_Pro; E088,H3K9ac_Pro; E101,H3K9ac_Pro; E102,H3K9ac_Pro; E114,H3K9ac_Pro; E119,H3K9ac_Pro; E122,H3K9ac_Pro; E127,H3K9ac_Pro; E025,H3K4me1_Enh; E026,H3K4me1_Enh; E027,H3K4me1_Enh; E028,H3K4me1_Enh; E036,H3K4me1_Enh; E049,H3K4me1_Enh; E050,H3K4me1_Enh; E051,H3K4me1_Enh; E057,H3K4me1_Enh; E058,H3K4me1_Enh; E063,H3K4me1_Enh; E078,H3K4me1_Enh; E088,H3K4me1_Enh; E101,H3K4me1_Enh; E102,H3K4me1_Enh; E103,H3K4me1_Enh; E114,H3K4me1_Enh; E119,H3K4me1_Enh; E127,H3K4me1_Enh; E128,H3K4me1_Enh; E027,H3K4me3_Pro; E051,H3K4me3_Pro; E078,H3K4me3_Pro; E088,H3K4me3_Pro; E101,H3K4me3_Pro; E111,H3K4me3_Pro; E127,H3K4me3_Pro	.	FAT, BRST, SKIN, LNG	E080	.	Elf5, Maf, Mef2, Pax-4, p300
rs7791513	7	26,893,357	RegulatoryFeature	REGULATORY		11.88	15.92	6.44						1414	9708	DNase1, Open Chromatin, DNase1 Hypersensitive Site H3K27me3, Histone, Histone 3 Lysine 27 Tri-Methylation Promoter Associated, Regulatory Feature, Promoter like regulatory feature	E025,7_Enh; E027,7_Enh; E028,7_Enh; E057,7_Enh; E058,6_EnhG; E114,7_Enh; E119,7_Enh; E127,7_Enh; E128,7_Enh	E023,17_EnhW2; E027,17_EnhW2; E028,15_EnhAF; E057,17_EnhW2; E058,17_EnhW2; E078,22_PromP; E119,15_EnhAF; E127,14_EnhA2	E012,H3K27ac_Enh; E013,H3K27ac_Enh; E050,H3K27ac_Enh; E058,H3K27ac_Enh; E063,H3K27ac_Enh; E076,H3K27ac_Enh; E078,H3K27ac_Enh; E119,H3K27ac_Enh; E127,H3K27ac_Enh; E023,H3K4me1_Enh; E025,H3K4me1_Enh; E026,H3K4me1_Enh; E027,H3K4me1_Enh; E028,H3K4me1_Enh; E036,H3K4me1_Enh; E049,H3K4me1_Enh; E050,H3K4me1_Enh; E051,H3K4me1_Enh; E057,H3K4me1_Enh; E058,H3K4me1_Enh; E063,H3K4me1_Enh; E078,H3K4me1_Enh; E086,H3K4me1_Enh; E088,H3K4me1_Enh; E101,H3K4me1_Enh; E102,H3K4me1_Enh; E103,H3K4me1_Enh; E114,H3K4me1_Enh; E119,H3K4me1_Enh; E122,H3K4me1_Enh; E127,H3K4me1_Enh; E128,H3K4me1_Enh; E023,H3K9ac_Pro; E025,H3K9ac_Pro; E026,H3K9ac_Pro; E049,H3K9ac_Pro; E063,H3K9ac_Pro; E066,H3K9ac_Pro; E076,H3K9ac_Pro; E086,H3K9ac_Pro; E088,H3K9ac_Pro; E101,H3K9ac_Pro; E102,H3K9ac_Pro; E114,H3K9ac_Pro; E119,H3K9ac_Pro; E122,H3K9ac_Pro; E127,H3K9ac_Pro; E027,H3K4me3_Pro; E051,H3K4me3_Pro; E078,H3K4me3_Pro; E088,H3K4me3_Pro; E101,H3K4me3_Pro; E111,H3K4me3_Pro; E127,H3K4me3_Pro	.	FAT, BRST, SKIN, LNG	E032;E080	.	UF1H3BETA
rs3213853	7	26,893,524	RegulatoryFeature	REGULATORY	0.44	7.92	18.16	7.56			5	11	150.2530	1247	9875	DNase1, Open Chromatin, DNase1 Hypersensitive Site H3K27me3, Histone, Histone 3 Lysine 27 Tri-Methylation Promoter Associated, Regulatory Feature, Promoter like regulatory feature CTCF, Transcription Factor, CCCTC-binding factor	E025,7_Enh; E027,7_Enh; E028,7_Enh; E051,7_Enh; E057,7_Enh; E058,7_Enh; E088,12_EnhBiv; E106,7_Enh; E114,7_Enh; E119,7_Enh; E122,7_Enh; E127,7_Enh	E005,19_DNase; E006,19_DNase; E007,19_DNase; E009,19_DNase; E011,19_DNase; E012,19_DNase; E013,19_DNase; E017,19_DNase; E021,19_DNase; E022,19_DNase; E023,17_EnhW2; E024,19_DNase; E025,17_EnhW2; E026,19_DNase; E027,17_EnhW2; E028,15_EnhAF; E029,19_DNase; E032,19_DNase; E033,19_DNase; E034,19_DNase; E036,19_DNase; E037,19_DNase; E038,19_DNase; E039,19_DNase; E041,19_DNase; E042,19_DNase; E043,19_DNase; E044,19_DNase; E045,19_DNase; E046,19_DNase; E047,19_DNase; E048,19_DNase; E049,19_DNase; E050,19_DNase; E051,19_DNase; E052,19_DNase; E053,19_DNase; E054,19_DNase; E055,19_DNase; E056,19_DNase; E057,17_EnhW2; E058,17_EnhW2; E059,19_DNase; E061,19_DNase; E062,19_DNase; E063,19_DNase; E065,19_DNase; E066,19_DNase; E067,19_DNase; E068,19_DNase; E069,19_DNase; E070,19_DNase; E071,19_DNase; E072,19_DNase; E073,19_DNase; E074,19_DNase; E075,19_DNase; E076,19_DNase; E077,19_DNase; E078,16_EnhW1; E079,19_DNase; E080,19_DNase; E081,19_DNase; E082,19_DNase; E083,19_DNase; E084,19_DNase; E085,19_DNase; E086,19_DNase; E087,19_DNase; E088,18_EnhAc; E089,19_DNase; E090,19_DNase; E091,19_DNase; E092,19_DNase; E093,19_DNase; E094,19_DNase; E095,19_DNase; E096,19_DNase; E097,19_DNase; E098,19_DNase; E099,19_DNase; E100,19_DNase; E101,19_DNase; E102,19_DNase; E103,19_DNase; E104,19_DNase; E105,19_DNase; E106,19_DNase; E107,19_DNase; E108,19_DNase; E109,19_DNase; E111,19_DNase; E112,19_DNase; E113,19_DNase; E114,19_DNase; E115,19_DNase; E116,19_DNase; E117,19_DNase; E118,19_DNase; E119,14_EnhA2; E120,19_DNase; E121,19_DNase; E122,19_DNase; E123,19_DNase; E125,19_DNase; E126,19_DNase; E127,14_EnhA2; E128,19_DNase; E129,19_DNase	E012,H3K27ac_Enh; E013,H3K27ac_Enh; E050,H3K27ac_Enh; E058,H3K27ac_Enh; E063,H3K27ac_Enh; E076,H3K27ac_Enh; E078,H3K27ac_Enh; E119,H3K27ac_Enh; E127,H3K27ac_Enh; E023,H3K4me1_Enh; E025,H3K4me1_Enh; E026,H3K4me1_Enh; E027,H3K4me1_Enh; E028,H3K4me1_Enh; E036,H3K4me1_Enh; E049,H3K4me1_Enh; E050,H3K4me1_Enh; E051,H3K4me1_Enh; E052,H3K4me1_Enh; E057,H3K4me1_Enh; E058,H3K4me1_Enh; E063,H3K4me1_Enh; E078,H3K4me1_Enh; E084,H3K4me1_Enh; E086,H3K4me1_Enh; E088,H3K4me1_Enh; E101,H3K4me1_Enh; E102,H3K4me1_Enh; E103,H3K4me1_Enh; E110,H3K4me1_Enh; E114,H3K4me1_Enh; E119,H3K4me1_Enh; E122,H3K4me1_Enh; E124,H3K4me1_Enh; E127,H3K4me1_Enh; E128,H3K4me1_Enh; E023,H3K9ac_Pro; E025,H3K9ac_Pro; E026,H3K9ac_Pro; E049,H3K9ac_Pro; E063,H3K9ac_Pro; E066,H3K9ac_Pro; E076,H3K9ac_Pro; E086,H3K9ac_Pro; E088,H3K9ac_Pro; E101,H3K9ac_Pro; E102,H3K9ac_Pro; E110,H3K9ac_Pro; E114,H3K9ac_Pro; E119,H3K9ac_Pro; E122,H3K9ac_Pro; E127,H3K9ac_Pro; E027,H3K4me3_Pro; E051,H3K4me3_Pro; E078,H3K4me3_Pro; E088,H3K4me3_Pro; E101,H3K4me3_Pro; E111,H3K4me3_Pro; E127,H3K4me3_Pro	.	FAT, BRST, BLD, SKIN, LNG, GI, VAS	E006;E007;E017;E028;E029;E032;E033;E034;E046;E050;E051;E055;E056;E057;E059;E080;E081;E082;E083;E084;E086;E088;E089;E090;E092;E093;E097;E100;E119;E120;E122;E123;E124;E127	CTCF, RAD21, SMC3, STAT3, FOXA1	.
rs6461980	7	26,894,123	Transcript	INTRONIC		16.48	10.52	4.08	3	-0.01				648	10474	H3K4me1, Histone, Histone 3 Lysine 4 Mono-Methylation H3K27me3, Histone, Histone 3 Lysine 27 Tri-Methylation	E025,7_Enh; E027,7_Enh; E051,7_Enh; E088,12_EnhBiv; E122,7_Enh; E127,7_Enh	E027,18_EnhAc; E028,18_EnhAc; E050,19_DNase; E051,19_DNase; E063,19_DNase; E068,19_DNase; E069,19_DNase; E071,19_DNase; E073,19_DNase; E074,19_DNase; E076,19_DNase; E078,22_PromP; E080,19_DNase; E083,19_DNase; E086,19_DNase; E088,18_EnhAc; E089,19_DNase; E090,19_DNase; E091,19_DNase; E092,19_DNase; E103,19_DNase; E107,19_DNase; E108,19_DNase; E111,19_DNase; E119,17_EnhW2; E127,17_EnhW2	E023,H3K4me1_Enh; E025,H3K4me1_Enh; E026,H3K4me1_Enh; E027,H3K4me1_Enh; E028,H3K4me1_Enh; E036,H3K4me1_Enh; E049,H3K4me1_Enh; E050,H3K4me1_Enh; E051,H3K4me1_Enh; E057,H3K4me1_Enh; E063,H3K4me1_Enh; E077,H3K4me1_Enh; E078,H3K4me1_Enh; E084,H3K4me1_Enh; E086,H3K4me1_Enh; E088,H3K4me1_Enh; E101,H3K4me1_Enh; E102,H3K4me1_Enh; E103,H3K4me1_Enh; E110,H3K4me1_Enh; E114,H3K4me1_Enh; E122,H3K4me1_Enh; E124,H3K4me1_Enh; E127,H3K4me1_Enh; E128,H3K4me1_Enh; E023,H3K9ac_Pro; E025,H3K9ac_Pro; E026,H3K9ac_Pro; E049,H3K9ac_Pro; E063,H3K9ac_Pro; E066,H3K9ac_Pro; E076,H3K9ac_Pro; E086,H3K9ac_Pro; E088,H3K9ac_Pro; E101,H3K9ac_Pro; E102,H3K9ac_Pro; E110,H3K9ac_Pro; E114,H3K9ac_Pro; E119,H3K9ac_Pro; E122,H3K9ac_Pro; E127,H3K9ac_Pro; E128,H3K9ac_Pro; E026,H3K4me3_Pro; E027,H3K4me3_Pro; E036,H3K4me3_Pro; E051,H3K4me3_Pro; E078,H3K4me3_Pro; E088,H3K4me3_Pro; E101,H3K4me3_Pro; E103,H3K4me3_Pro; E110,H3K4me3_Pro; E111,H3K4me3_Pro; E127,H3K4me3_Pro; E050,H3K27ac_Enh; E058,H3K27ac_Enh; E063,H3K27ac_Enh; E076,H3K27ac_Enh; E078,H3K27ac_Enh; E102,H3K27ac_Enh; E119,H3K27ac_Enh; E127,H3K27ac_Enh	.	FAT, BRST, BLD, LNG, VAS, SKIN	E088;E090;E119	.	Evi-1, GATA, IRC900814

rs12536145	7	26,894,860	RegulatoryFeature	REGULATORY		35.72	14.92	7.44			2	3	68.5835	90	11211	DNase1, Open Chromatin, DNase1 Hypersensitive Site H3K4me1, Histone, Histone 3 Lysine 4 Mono-Methylation H3K4me2, Histone, Histone 3 Lysine 4 Di-Methylation H3K27me3, Histone, Histone 3 Lysine 27 Tri-Methylation H3K27ac, Histone, Histone 3 Lysine 27 Acetylation Promoter Associated, Regulatory Feature, Promoter like regulatory feature	E023,7_Enh; E025,7_Enh; E027,7_Enh; E028,7_Enh; E035,12_EnhBiv; E036,7_Enh; E050,7_Enh; E051,2_TssAFlnk; E057,7_Enh; E058,7_Enh; E078,7_Enh; E086,12_EnhBiv; E088,12_EnhBiv; E102,7_Enh; E114,7_Enh; E119,2_TssAFlnk; E122,7_Enh; E127,7_Enh; E128,7_Enh	E023,14_EnhA2; E025,15_EnhAF; E026,22_PromP; E027,9_TxReg; E028,9_TxReg; E035,12_TxEnhW; E036,10_TxEnh5; E049,18_EnhAc; E050,10_TxEnh5; E051,9_TxReg; E057,10_TxEnh5; E058,9_TxReg; E061,22_PromP; E063,9_TxReg; E066,18_EnhAc; E076,14_EnhA2; E078,9_TxReg; E080,18_EnhAc; E086,23_PromBiv; E087,18_EnhAc; E088,10_TxEnh5; E092,19_DNase; E101,10_TxEnh5; E102,10_TxEnh5; E103,22_PromP; E108,18_EnhAc; E110,4_PromD2; E111,18_EnhAc; E114,18_EnhAc; E117,18_EnhAc; E119,9_TxReg; E122,18_EnhAc; E127,9_TxReg	E012,H3K27ac_Enh; E026,H3K27ac_Enh; E049,H3K27ac_Enh; E050,H3K27ac_Enh; E058,H3K27ac_Enh; E063,H3K27ac_Enh; E075,H3K27ac_Enh; E076,H3K27ac_Enh; E078,H3K27ac_Enh; E080,H3K27ac_Enh; E085,H3K27ac_Enh; E101,H3K27ac_Enh; E102,H3K27ac_Enh; E111,H3K27ac_Enh; E119,H3K27ac_Enh; E122,H3K27ac_Enh; E127,H3K27ac_Enh; E128,H3K27ac_Enh; E023,H3K4me1_Enh; E025,H3K4me1_Enh; E026,H3K4me1_Enh; E027,H3K4me1_Enh; E028,H3K4me1_Enh; E030,H3K4me1_Enh; E036,H3K4me1_Enh; E049,H3K4me1_Enh; E050,H3K4me1_Enh; E051,H3K4me1_Enh; E057,H3K4me1_Enh; E058,H3K4me1_Enh; E061,H3K4me1_Enh; E063,H3K4me1_Enh; E077,H3K4me1_Enh; E078,H3K4me1_Enh; E084,H3K4me1_Enh; E086,H3K4me1_Enh; E088,H3K4me1_Enh; E101,H3K4me1_Enh; E102,H3K4me1_Enh; E103,H3K4me1_Enh; E110,H3K4me1_Enh; E114,H3K4me1_Enh; E122,H3K4me1_Enh; E124,H3K4me1_Enh; E127,H3K4me1_Enh; E128,H3K4me1_Enh; E023,H3K9ac_Pro; E025,H3K9ac_Pro; E026,H3K9ac_Pro; E027,H3K9ac_Pro; E049,H3K9ac_Pro; E063,H3K9ac_Pro; E066,H3K9ac_Pro; E076,H3K9ac_Pro; E086,H3K9ac_Pro; E088,H3K9ac_Pro; E101,H3K9ac_Pro; E102,H3K9ac_Pro; E110,H3K9ac_Pro; E114,H3K9ac_Pro; E119,H3K9ac_Pro; E122,H3K9ac_Pro; E127,H3K9ac_Pro; E128,H3K9ac_Pro; E025,H3K4me3_Pro; E026,H3K4me3_Pro; E027,H3K4me3_Pro; E028,H3K4me3_Pro; E036,H3K4me3_Pro; E046,H3K4me3_Pro; E050,H3K4me3_Pro; E051,H3K4me3_Pro; E078,H3K4me3_Pro; E088,H3K4me3_Pro; E101,H3K4me3_Pro; E102,H3K4me3_Pro; E103,H3K4me3_Pro; E110,H3K4me3_Pro; E111,H3K4me3_Pro; E119,H3K4me3_Pro; E122,H3K4me3_Pro; E127,H3K4me3_Pro	BLD, BRST	FAT, BRST, BLD, SKIN, GI, KID, LNG, VAS	E007;E028;E057;E082;E083;E088;E089;E090;E119;E122	.	Pou1f1, Pou3f3, TATA
rs10242572	7	26,895,065	RegulatoryFeature	REGULATORY		40.32	14.88	17.52						295	11416	DNase1, Open Chromatin, DNase1 Hypersensitive Site H3K4me1, Histone, Histone 3 Lysine 4 Mono-Methylation H3K4me2, Histone, Histone 3 Lysine 4 Di-Methylation H3K27me3, Histone, Histone 3 Lysine 27 Tri-Methylation H3K27ac, Histone, Histone 3 Lysine 27 Acetylation Promoter Associated, Regulatory Feature, Promoter like regulatory feature	0	0	E012,H3K27ac_Enh; E026,H3K27ac_Enh; E049,H3K27ac_Enh; E050,H3K27ac_Enh; E058,H3K27ac_Enh; E063,H3K27ac_Enh; E075,H3K27ac_Enh; E076,H3K27ac_Enh; E078,H3K27ac_Enh; E080,H3K27ac_Enh; E085,H3K27ac_Enh; E101,H3K27ac_Enh; E102,H3K27ac_Enh; E103,H3K27ac_Enh; E111,H3K27ac_Enh; E114,H3K27ac_Enh; E119,H3K27ac_Enh; E122,H3K27ac_Enh; E127,H3K27ac_Enh; E128,H3K27ac_Enh; E017,H3K9ac_Pro; E023,H3K9ac_Pro; E025,H3K9ac_Pro; E026,H3K9ac_Pro; E027,H3K9ac_Pro; E049,H3K9ac_Pro; E063,H3K9ac_Pro; E066,H3K9ac_Pro; E076,H3K9ac_Pro; E086,H3K9ac_Pro; E088,H3K9ac_Pro; E101,H3K9ac_Pro; E102,H3K9ac_Pro; E110,H3K9ac_Pro; E114,H3K9ac_Pro; E119,H3K9ac_Pro; E122,H3K9ac_Pro; E127,H3K9ac_Pro; E128,H3K9ac_Pro; E023,H3K4me1_Enh; E025,H3K4me1_Enh; E026,H3K4me1_Enh; E027,H3K4me1_Enh; E028,H3K4me1_Enh; E030,H3K4me1_Enh; E035,H3K4me1_Enh; E036,H3K4me1_Enh; E049,H3K4me1_Enh; E050,H3K4me1_Enh; E051,H3K4me1_Enh; E057,H3K4me1_Enh; E058,H3K4me1_Enh; E061,H3K4me1_Enh; E063,H3K4me1_Enh; E077,H3K4me1_Enh; E078,H3K4me1_Enh; E084,H3K4me1_Enh; E086,H3K4me1_Enh; E088,H3K4me1_Enh; E101,H3K4me1_Enh; E102,H3K4me1_Enh; E103,H3K4me1_Enh; E110,H3K4me1_Enh; E114,H3K4me1_Enh; E116,H3K4me1_Enh; E122,H3K4me1_Enh; E124,H3K4me1_Enh; E127,H3K4me1_Enh; E128,H3K4me1_Enh; E023,H3K4me3_Pro; E025,H3K4me3_Pro; E026,H3K4me3_Pro; E027,H3K4me3_Pro; E028,H3K4me3_Pro; E036,H3K4me3_Pro; E046,H3K4me3_Pro; E050,H3K4me3_Pro; E051,H3K4me3_Pro; E058,H3K4me3_Pro; E078,H3K4me3_Pro; E088,H3K4me3_Pro; E101,H3K4me3_Pro; E102,H3K4me3_Pro; E103,H3K4me3_Pro; E110,H3K4me3_Pro; E111,H3K4me3_Pro; E114,H3K4me3_Pro; E119,H3K4me3_Pro; E122,H3K4me3_Pro; E127,H3K4me3_Pro	.	.	E007;E028;E057;E086;E088;E089;E090;E092;E119;E122	.	EWSR1-FLI1, SP1, WT1, ZNF263
rs10256250	7	26,895,080	RegulatoryFeature	REGULATORY		40.32	14.88	15.40						310	11431	DNase1, Open Chromatin, DNase1 Hypersensitive Site H3K4me1, Histone, Histone 3 Lysine 4 Mono-Methylation H3K4me2, Histone, Histone 3 Lysine 4 Di-Methylation H3K27me3, Histone, Histone 3 Lysine 27 Tri-Methylation H3K27ac, Histone, Histone 3 Lysine 27 Acetylation Promoter Associated, Regulatory Feature, Promoter like regulatory feature	.	.	E012,H3K27ac_Enh; E026,H3K27ac_Enh; E049,H3K27ac_Enh; E050,H3K27ac_Enh; E058,H3K27ac_Enh; E063,H3K27ac_Enh; E075,H3K27ac_Enh; E076,H3K27ac_Enh; E078,H3K27ac_Enh; E080,H3K27ac_Enh; E085,H3K27ac_Enh; E101,H3K27ac_Enh; E102,H3K27ac_Enh; E103,H3K27ac_Enh; E111,H3K27ac_Enh; E114,H3K27ac_Enh; E119,H3K27ac_Enh; E122,H3K27ac_Enh; E127,H3K27ac_Enh; E128,H3K27ac_Enh; E017,H3K9ac_Pro; E023,H3K9ac_Pro; E025,H3K9ac_Pro; E026,H3K9ac_Pro; E027,H3K9ac_Pro; E049,H3K9ac_Pro; E063,H3K9ac_Pro; E066,H3K9ac_Pro; E076,H3K9ac_Pro; E086,H3K9ac_Pro; E088,H3K9ac_Pro; E101,H3K9ac_Pro; E102,H3K9ac_Pro; E110,H3K9ac_Pro; E114,H3K9ac_Pro; E119,H3K9ac_Pro; E122,H3K9ac_Pro; E127,H3K9ac_Pro; E128,H3K9ac_Pro; E023,H3K4me1_Enh; E025,H3K4me1_Enh; E026,H3K4me1_Enh; E028,H3K4me1_Enh; E030,H3K4me1_Enh; E035,H3K4me1_Enh; E036,H3K4me1_Enh; E049,H3K4me1_Enh; E050,H3K4me1_Enh; E051,H3K4me1_Enh; E057,H3K4me1_Enh; E058,H3K4me1_Enh; E061,H3K4me1_Enh; E063,H3K4me1_Enh; E077,H3K4me1_Enh; E078,H3K4me1_Enh; E084,H3K4me1_Enh; E086,H3K4me1_Enh; E088,H3K4me1_Enh; E101,H3K4me1_Enh; E102,H3K4me1_Enh; E103,H3K4me1_Enh; E110,H3K4me1_Enh; E114,H3K4me1_Enh; E116,H3K4me1_Enh; E122,H3K4me1_Enh; E124,H3K4me1_Enh; E127,H3K4me1_Enh; E128,H3K4me1_Enh; E023,H3K4me3_Pro; E025,H3K4me3_Pro; E026,H3K4me3_Pro; E027,H3K4me3_Pro; E028,H3K4me3_Pro; E036,H3K4me3_Pro; E046,H3K4me3_Pro; E050,H3K4me3_Pro; E051,H3K4me3_Pro; E058,H3K4me3_Pro; E078,H3K4me3_Pro; E088,H3K4me3_Pro; E101,H3K4me3_Pro; E102,H3K4me3_Pro; E103,H3K4me3_Pro; E110,H3K4me3_Pro; E111,H3K4me3_Pro; E114,H3K4me3_Pro; E119,H3K4me3_Pro; E122,H3K4me3_Pro; E127,H3K4me3_Pro	.	.	E007;E028;E057;E086;E088;E089;E090;E092;E119;E122;E127	.	AP-1
rs3801809	7	26,895,456	RegulatoryFeature	REGULATORY		18.96	11.60	14.60						686	11807	DNase1, Open Chromatin, DNase1 Hypersensitive Site H3K4me1, Histone, Histone 3 Lysine 4 Mono-Methylation H3K4me2, Histone, Histone 3 Lysine 4 Di-Methylation H3K27me3, Histone, Histone 3 Lysine 27 Tri-Methylation H3K27ac, Histone, Histone 3 Lysine 27 Acetylation Promoter Associated, Regulatory Feature, Promoter like regulatory feature	E023,7_Enh; E025,1_TssA; E027,1_TssA; E028,1_TssA; E035,1_TssA; E036,1_TssA; E050,7_Enh; E051,1_TssA; E057,7_Enh; E058,2_TssAFlnk; E061,7_Enh; E102,7_Enh; E114,7_Enh; E119,1_TssA; E122,2_TssAFlnk; E127,1_TssA; E128,1_TssA	E023,13_EnhA1; E025,13_EnhA1; E026,22_PromP; E027,2_PromU; E028,3_PromD1; E035,4_PromD2; E036,4_PromD2; E049,18_EnhAc; E050,9_TxReg; E051,9_TxReg; E057,4_PromD2; E058,3_PromD1; E061,22_PromP; E063,9_TxReg; E066,18_EnhAc; E076,14_EnhA2; E078,14_EnhA2; E080,18_EnhAc; E084,22_PromP; E086,22_PromP; E087,18_EnhAc; E088,14_EnhA2; E101,18_EnhAc; E102,18_EnhAc; E103,22_PromP; E108,18_EnhAc; E110,22_PromP; E111,18_EnhAc; E114,18_EnhAc; E117,18_EnhAc; E119,9_TxReg; E122,13_EnhA1; E124,10_TxEnh5; E127,9_TxReg	E012,H3K27ac_Enh; E026,H3K27ac_Enh; E049,H3K27ac_Enh; E050,H3K27ac_Enh; E058,H3K27ac_Enh; E063,H3K27ac_Enh; E075,H3K27ac_Enh; E076,H3K27ac_Enh; E078,H3K27ac_Enh; E080,H3K27ac_Enh; E085,H3K27ac_Enh; E101,H3K27ac_Enh; E102,H3K27ac_Enh; E103,H3K27ac_Enh; E111,H3K27ac_Enh; E114,H3K27ac_Enh; E119,H3K27ac_Enh; E122,H3K27ac_Enh; E127,H3K27ac_Enh; E128,H3K27ac_Enh; E017,H3K9ac_Pro; E019,H3K9ac_Pro; E023,H3K9ac_Pro; E025,H3K9ac_Pro; E026,H3K9ac_Pro; E027,H3K9ac_Pro; E049,H3K9ac_Pro; E063,H3K9ac_Pro; E066,H3K9ac_Pro; E076,H3K9ac_Pro; E086,H3K9ac_Pro; E088,H3K9ac_Pro; E101,H3K9ac_Pro; E102,H3K9ac_Pro; E110,H3K9ac_Pro; E114,H3K9ac_Pro; E119,H3K9ac_Pro; E122,H3K9ac_Pro; E127,H3K9ac_Pro; E128,H3K9ac_Pro; E023,H3K4me1_Enh; E025,H3K4me1_Enh; E026,H3K4me1_Enh; E028,H3K4me1_Enh; E030,H3K4me1_Enh; E035,H3K4me1_Enh; E036,H3K4me1_Enh; E049,H3K4me1_Enh; E050,H3K4me1_Enh; E051,H3K4me1_Enh; E058,H3K4me1_Enh; E061,H3K4me1_Enh; E063,H3K4me1_Enh; E077,H3K4me1_Enh; E078,H3K4me1_Enh; E084,H3K4me1_Enh; E086,H3K4me1_Enh; E088,H3K4me1_Enh; E101,H3K4me1_Enh; E102,H3K4me1_Enh; E103,H3K4me1_Enh; E110,H3K4me1_Enh; E114,H3K4me1_Enh; E116,H3K4me1_Enh; E122,H3K4me1_Enh; E124,H3K4me1_Enh; E128,H3K4me1_Enh; E023,H3K4me3_Pro; E025,H3K4me3_Pro; E026,H3K4me3_Pro; E027,H3K4me3_Pro; E028,H3K4me3_Pro; E035,H3K4me3_Pro; E036,H3K4me3_Pro; E041,H3K4me3_Pro; E046,H3K4me3_Pro; E050,H3K4me3_Pro; E051,H3K4me3_Pro; E057,H3K4me3_Pro; E058,H3K4me3_Pro; E063,H3K4me3_Pro; E078,H3K4me3_Pro; E088,H3K4me3_Pro; E101,H3K4me3_Pro; E102,H3K4me3_Pro; E103,H3K4me3_Pro; E110,H3K4me3_Pro; E111,H3K4me3_Pro; E114,H3K4me3_Pro; E119,H3K4me3_Pro; E122,H3K4me3_Pro; E127,H3K4me3_Pro; E128,H3K4me3_Pro	FAT, BRST, BLD, SKIN, VAS, LNG	FAT, BLD, SKIN, GI, LNG	E028;E057;E088;E090;E119;E122	.	Ets, SIX5, YY1
rs3801808	7	26,896,555	RegulatoryFeature	REGULATORY		22.64	12.32	21.96						685	12906	DNase1, Open Chromatin, DNase1 Hypersensitive Site H3K36me3, Histone, Histone 3 Lysine 36 Tri-Methylation H3K4me2, Histone, Histone 3 Lysine 4 Di-Methylation H3K27me3, Histone, Histone 3 Lysine 27 Tri-Methylation H3K27ac, Histone, Histone 3 Lysine 27 Acetylation Promoter Associated, Regulatory Feature, Promoter like regulatory feature	E018,12_EnhBiv; E023,2_TssAFlnk; E025,2_TssAFlnk; E026,7_Enh; E027,1_TssA; E028,1_TssA; E029,7_Enh; E030,7_Enh; E031,12_EnhBiv; E035,2_TssAFlnk; E036,2_TssAFlnk; E049,7_Enh; E050,2_TssAFlnk; E051,2_TssAFlnk; E058,1_TssA; E061,7_Enh; E063,1_TssA; E078,1_TssA; E080,7_Enh; E088,12_EnhBiv; E101,1_TssA; E102,7_Enh; E111,1_TssA; E114,7_Enh; E116,7_Enh; E119,1_TssA; E122,2_TssAFlnk; E124,7_Enh; E127,7_Enh; E128,1_TssA	E010,23_PromBiv; E015,23_PromBiv; E017,22_PromP; E018,23_PromBiv; E023,9_TxReg; E025,3_PromD1; E026,22_PromP; E027,9_TxReg; E028,9_TxReg; E029,4_PromD2; E030,9_TxReg; E031,4_PromD2; E032,22_PromP; E033,23_PromBiv; E035,9_TxReg; E036,9_TxReg; E041,23_PromBiv; E046,22_PromP; E048,23_PromBiv; E049,22_PromP; E050,3_PromD1; E051,3_PromD1; E055,22_PromP; E056,22_PromP; E057,22_PromP; E058,9_TxReg; E061,14_EnhA2; E063,3_PromD1; E065,22_PromP; E066,18_EnhAc; E067,19_DNase; E068,22_PromP; E071,22_PromP; E072,22_PromP; E074,19_DNase; E075,22_PromP; E076,14_EnhA2; E077,22_PromP; E078,9_TxReg; E080,14_EnhA2; E081,23_PromBiv; E083,23_PromBiv; E084,22_PromP; E085,22_PromP; E086,23_PromBiv; E087,22_PromP; E088,3_PromD1; E091,22_PromP; E092,22_PromP; E093,22_PromP; E095,22_PromP; E096,22_PromP; E099,22_PromP; E101,14_EnhA2; E102,9_TxReg; E103,22_PromP; E106,22_PromP; E108,18_EnhAc; E109,22_PromP; E110,4_PromD2; E111,14_EnhA2; E113,22_PromP; E114,22_PromP; E116,22_PromP; E117,18_EnhAc; E118,22_PromP; E119,9_TxReg; E122,9_TxReg; E124,4_PromD2; E125,22_PromP; E127,3_PromD1; E128,22_PromP; E129,22_PromP	E001,H3K4me1_Enh; E002,H3K4me1_Enh; E003,H3K4me1_Enh; E010,H3K4me1_Enh; E011,H3K4me1_Enh; E012,H3K4me1_Enh; E013,H3K4me1_Enh; E014,H3K4me1_Enh; E015,H3K4me1_Enh; E016,H3K4me1_Enh; E017,H3K4me1_Enh; E018,H3K4me1_Enh; E019,H3K4me1_Enh; E020,H3K4me1_Enh; E023,H3K4me1_Enh; E024,H3K4me1_Enh; E025,H3K4me1_Enh; E026,H3K4me1_Enh; E028,H3K4me1_Enh; E029,H3K4me1_Enh; E030,H3K4me1_Enh; E031,H3K4me1_Enh; E032,H3K4me1_Enh; E035,H3K4me1_Enh; E036,H3K4me1_Enh; E037,H3K4me1_Enh; E039,H3K4me1_Enh; E041,H3K4me1_Enh; E042,H3K4me1_Enh; E043,H3K4me1_Enh; E046,H3K4me1_Enh; E048,H3K4me1_Enh; E049,H3K4me1_Enh; E050,H3K4me1_Enh; E051,H3K4me1_Enh; E056,H3K4me1_Enh; E057,H3K4me1_Enh; E058,H3K4me1_Enh; E061,H3K4me1_Enh; E063,H3K4me1_Enh; E066,H3K4me1_Enh; E071,H3K4me1_Enh; E075,H3K4me1_Enh; E077,H3K4me1_Enh; E078,H3K4me1_Enh; E080,H3K4me1_Enh; E084,H3K4me1_Enh; E085,H3K4me1_Enh; E086,H3K4me1_Enh; E088,H3K4me1_Enh; E092,H3K4me1_Enh; E099,H3K4me1_Enh; E101,H3K4me1_Enh; E102,H3K4me1_Enh; E110,H3K4me1_Enh; E114,H3K4me1_Enh; E116,H3K4me1_Enh; E119,H3K4me1_Enh; E122,H3K4me1_Enh; E124,H3K4me1_Enh; E125,H3K4me1_Enh; E127,H3K4me1_Enh; E128,H3K4me1_Enh; E003,H3K4me3_Pro; E010,H3K4me3_Pro; E015,H3K4me3_Pro; E017,H3K4me3_Pro; E018,H3K4me3_Pro; E019,H3K4me3_Pro; E023,H3K4me3_Pro; E025,H3K4me3_Pro; E026,H3K4me3_Pro; E027,H3K4me3_Pro; E028,H3K4me3_Pro; E031,H3K4me3_Pro; E035,H3K4me3_Pro; E036,H3K4me3_Pro; E040,H3K4me3_Pro; E041,H3K4me3_Pro; E045,H3K4me3_Pro; E046,H3K4me3_Pro; E049,H3K4me3_Pro; E050,H3K4me3_Pro; E051,H3K4me3_Pro; E057,H3K4me3_Pro; E058,H3K4me3_Pro; E061,H3K4me3_Pro; E062,H3K4me3_Pro; E063,H3K4me3_Pro; E066,H3K4me3_Pro; E067,H3K4me3_Pro; E068,H3K4me3_Pro; E072,H3K4me3_Pro; E077,H3K4me3_Pro; E078,H3K4me3_Pro; E080,H3K4me3_Pro; E084,H3K4me3_Pro; E085,H3K4me3_Pro; E086,H3K4me3_Pro; E088,H3K4me3_Pro; E100,H3K4me3_Pro; E101,H3K4me3_Pro; E102,H3K4me3_Pro; E103,H3K4me3_Pro; E104,H3K4me3_Pro; E110,H3K4me3_Pro; E111,H3K4me3_Pro; E114,H3K4me3_Pro; E116,H3K4me3_Pro; E119,H3K4me3_Pro; E122,H3K4me3_Pro; E124,H3K4me3_Pro; E127,H3K4me3_Pro; E128,H3K4me3_Pro; E012,H3K27ac_Enh; E013,H3K27ac_Enh; E017,H3K27ac_Enh; E026,H3K27ac_Enh; E046,H3K27ac_Enh; E049,H3K27ac_Enh; E050,H3K27ac_Enh; E058,H3K27ac_Enh; E061,H3K27ac_Enh; E063,H3K27ac_Enh; E066,H3K27ac_Enh; E075,H3K27ac_Enh; E076,H3K27ac_Enh; E078,H3K27ac_Enh; E079,H3K27ac_Enh; E080,H3K27ac_Enh; E084,H3K27ac_Enh; E085,H3K27ac_Enh; E094,H3K27ac_Enh; E101,H3K27ac_Enh; E102,H3K27ac_Enh; E103,H3K27ac_Enh; E108,H3K27ac_Enh; E111,H3K27ac_Enh; E113,H3K27ac_Enh; E114,H3K27ac_Enh; E119,H3K27ac_Enh; E122,H3K27ac_Enh; E127,H3K27ac_Enh; E128,H3K27ac_Enh; E015,H3K9ac_Pro; E017,H3K9ac_Pro; E019,H3K9ac_Pro; E023,H3K9ac_Pro; E025,H3K9ac_Pro; E026,H3K9ac_Pro; E027,H3K9ac_Pro; E049,H3K9ac_Pro; E063,H3K9ac_Pro; E066,H3K9ac_Pro; E072,H3K9ac_Pro; E075,H3K9ac_Pro; E076,H3K9ac_Pro; E086,H3K9ac_Pro; E088,H3K9ac_Pro; E101,H3K9ac_Pro; E102,H3K9ac_Pro; E103,H3K9ac_Pro; E110,H3K9ac_Pro; E111,H3K9ac_Pro; E114,H3K9ac_Pro; E119,H3K9ac_Pro; E122,H3K9ac_Pro; E127,H3K9ac_Pro; E128,H3K9ac_Pro	FAT, BRST, BLD, SKIN, GI, VAS, LNG	IPSC, STRM, BLD, SKIN, ADRL, LNG, GI	E028;E088;E090;E122	.	GATA
rs3801807	7	26,897,439	RegulatoryFeature	REGULATORY		76.96	26.04	53.92			2	2	44.0918	142	13790	DNase1, Open Chromatin, DNase1 Hypersensitive Site H3K9ac, Histone, Histone 3 Lysine 9 Acetylation H3K4me3, Histone, Histone 3 Lysine 4 Tri-Methylation H3K36me3, Histone, Histone 3 Lysine 36 Tri-Methylation H3K4me2, Histone, Histone 3 Lysine 4 Di-Methylation H3K27me3, Histone, Histone 3 Lysine 27 Tri-Methylation H3K27ac, Histone, Histone 3 Lysine 27 Acetylation H2AZ, Histone, Histone 2A variant Z Promoter Associated, Regulatory Feature, Promoter like regulatory feature	E001,10_TssBiv; E002,10_TssBiv; E003,11_BivFlnk; E005,12_EnhBiv; E006,1_TssA; E008,10_TssBiv; E009,11_BivFlnk; E010,11_BivFlnk; E011,12_EnhBiv; E012,12_EnhBiv; E013,1_TssA; E014,11_BivFlnk; E015,10_TssBiv; E016,11_BivFlnk; E017,2_TssAFlnk; E018,10_TssBiv; E019,11_BivFlnk; E020,10_TssBiv; E021,11_BivFlnk; E022,12_EnhBiv; E023,1_TssA; E024,12_EnhBiv; E025,1_TssA; E026,1_TssA; E027,1_TssA; E028,1_TssA; E029,12_EnhBiv; E030,2_TssAFlnk; E031,2_TssAFlnk; E032,2_TssAFlnk; E034,12_EnhBiv; E035,1_TssA; E036,2_TssAFlnk; E046,11_BivFlnk; E050,2_TssAFlnk; E051,2_TssAFlnk; E053,12_EnhBiv; E055,11_BivFlnk; E056,2_TssAFlnk; E057,1_TssA; E058,1_TssA; E061,1_TssA; E063,2_TssAFlnk; E066,12_EnhBiv; E067,10_TssBiv; E068,10_TssBiv; E070,12_EnhBiv; E071,10_TssBiv; E072,10_TssBiv; E073,10_TssBiv; E075,1_TssA; E077,10_TssBiv; E078,1_TssA; E079,7_Enh; E080,2_TssAFlnk; E081,10_TssBiv; E084,11_BivFlnk; E085,12_EnhBiv; E086,10_TssBiv; E088,11_BivFlnk; E089,12_EnhBiv; E091,12_EnhBiv; E092,11_BivFlnk; E096,1_TssA; E097,12_EnhBiv; E099,12_EnhBiv; E100,10_TssBiv; E101,1_TssA; E102,2_TssAFlnk; E103,1_TssA; [illegible]	E001,2_PromU; E002,23_PromBiv; E003,2_PromU; E004,23_PromBiv; E005,23_PromBiv; E006,3_PromD1; E007,23_PromBiv; E008,2_PromU; E009,23_PromBiv; E010,2_PromU; E011,23_PromBiv; E012,23_PromBiv; E013,2_PromU; E014,2_PromU; E015,2_PromU; E016,2_PromU; E017,3_PromD1; E018,2_PromU; E019,2_PromU; E020,2_PromU; E021,23_PromBiv; E022,23_PromBiv; E023,3_PromD1; E024,2_PromU; E025,3_PromD1; E026,3_PromD1; E027,3_PromD1; E028,3_PromD1; E029,3_PromD1; E030,3_PromD1; E031,3_PromD1; E032,3_PromD1; E033,2_PromU; E034,2_PromU; E035,3_PromD1; E036,3_PromD1; E037,23_PromBiv; E038,23_PromBiv; E039,23_PromBiv; E040,23_PromBiv; E041,23_PromBiv; E042,23_PromBiv; E043,23_PromBiv; E044,23_PromBiv; E045,23_PromBiv; E046,3_PromD1; E047,23_PromBiv; E048,23_PromBiv; E049,3_PromD1; E050,3_PromD1; E051,3_PromD1; E053,23_PromBiv; E054,23_PromBiv; E055,3_PromD1; E056,3_PromD1; E057,3_PromD1; E058,3_PromD1; E059,3_PromD1; E061,3_PromD1; E062,4_PromD2; E063,3_PromD1; E065,22_PromP; E066,3_PromD1; E067,2_PromU; E068,3_PromD1; E069,23_PromBiv; E070,23_PromBiv; E071,3_PromD1; E072,23_PromBiv; E073,2_PromU; E074,23_PromBiv; E075,3_PromD1; E076,3_PromD1; E077,3_PromD1; E078,3_PromD1; E079,3_PromD1; E080,3_PromD1; E081,2_PromU; E082,23_PromBiv; E083,3_PromD1; E084,3_PromD1; E085,3_PromD1; E086,3_PromD1; E087,3_PromD1; E088,3_PromD1; E089,2_PromU; E090,2_PromU; E091,3_PromD1; E092,3_PromD1; E093,23_PromBiv; E094,4_PromD2; E095,4_PromD2; E096,3_PromD1; E097,2_PromU; E098,4_PromD2; E099,3_PromD1; E100,3_PromD1; E101,3_PromD1; E102,3_PromD1; E103,3_PromD1; E104,4_PromD2; E105,23_PromBiv; E106,3_PromD1; E107,3_PromD1; E108,3_PromD1; E109,3_PromD1; E110,3_PromD1; E111,3_PromD1; E112,23_PromBiv; E113,3_PromD1; E114,3_PromD1; E115,23_PromBiv; E116,3_PromD1; E117,4_PromD2; E118,2_PromU; E119,3_PromD1; E120,23_PromBiv; E121,23_PromBiv; E122,3_PromD1; E123,3_PromD1; E124,3_PromD1; E125,3_PromD1; E126,4_PromD2; E127,3_PromD1; E128,3_PromD1; E129,23_PromBiv	E001,H3K4me1_Enh; E002,H3K4me1_Enh; E003,H3K4me1_Enh; E005,H3K4me1_Enh; E006,H3K4me1_Enh; E007,H3K4me1_Enh; E008,H3K4me1_Enh; E009,H3K4me1_Enh; E010,H3K4me1_Enh; E011,H3K4me1_Enh; E012,H3K4me1_Enh; E013,H3K4me1_Enh; E014,H3K4me1_Enh; E015,H3K4me1_Enh; E016,H3K4me1_Enh; E017,H3K4me1_Enh; E018,H3K4me1_Enh; E019,H3K4me1_Enh; E020,H3K4me1_Enh; E021,H3K4me1_Enh; E022,H3K4me1_Enh; E023,H3K4me1_Enh; E024,H3K4me1_Enh; E025,H3K4me1_Enh; E026,H3K4me1_Enh; E027,H3K4me1_Enh; E028,H3K4me1_Enh; E029,H3K4me1_Enh; E030,H3K4me1_Enh; E031,H3K4me1_Enh; E032,H3K4me1_Enh; E034,H3K4me1_Enh; E035,H3K4me1_Enh; E036,H3K4me1_Enh; E039,H3K4me1_Enh; E040,H3K4me1_Enh; E041,H3K4me1_Enh; E042,H3K4me1_Enh; E043,H3K4me1_Enh; E046,H3K4me1_Enh; E047,H3K4me1_Enh; E048,H3K4me1_Enh; E049,H3K4me1_Enh; E050,H3K4me1_Enh; E051,H3K4me1_Enh; E055,H3K4me1_Enh; E056,H3K4me1_Enh; E057,H3K4me1_Enh; E058,H3K4me1_Enh; E061,H3K4me1_Enh; E063,H3K4me1_Enh; E066,H3K4me1_Enh; E071,H3K4me1_Enh; E075,H3K4me1_Enh; E077,H3K4me1_Enh; E078,H3K4me1_Enh; E079,H3K4me1_Enh; E080,H3K4me1_Enh; E084,H3K4me1_Enh; E085,H3K4me1_Enh; E086,H3K4me1_Enh; E088,H3K4me1_Enh; E089,H3K4me1_Enh; E092,H3K4me1_Enh; E093,H3K4me1_Enh; E096,H3K4me1_Enh; E097,H3K4me1_Enh; E099,H3K4me1_Enh; E101,H3K4me1_Enh; E102,H3K4me1_Enh; E104,H3K4me1_Enh; E106,H3K4me1_Enh; E110,H3K4me1_Enh; E111,H3K4me1_Enh; E113,H3K4me1_Enh; E114,H3K4me1_Enh; E116,H3K4me1_Enh; E119,H3K4me1_Enh; E122,H3K4me1_Enh; E123,H3K4me1_Enh; E124,H3K4me1_Enh; E125,H3K4me1_Enh; E127,H3K4me1_Enh; E128,H3K4me1_Enh; E001,H3K4me3_Pro; E002,H3K4me3_Pro; E003,H3K4me3_Pro; E006,H3K4me3_Pro; E008,H3K4me3_Pro; E010,H3K4me3_Pro; E011,H3K4me3_Pro; E012,H3K4me3_Pro; E013,H3K4me3_Pro; E014,H3K4me3_Pro; E015,H3K4me3_Pro; E016,H3K4me3_Pro; E017,H3K4me3_Pro; E018,H3K4me3_Pro; E019,H3K4me3_Pro; E020,H3K4me3_Pro; E021,H3K4me3_Pro; E022,H3K4me3_Pro; E023,H3K4me3_Pro; E024,H3K4me3_Pro; E025,H3K4me3_Pro; E026,H3K4me3_Pro; E027,H3K4me3_Pro; E028,H3K4me3_Pro; E030,H3K4me3_Pro; E031,H3K4me3_Pro; E032,H3K4me3_Pro; E033,H3K4me3_Pro; E034,H3K4me3_Pro; E035,H3K4me3_Pro; E036,H3K4me3_Pro; E041,H3K4me3_Pro; E045,H3K4me3_Pro; E046,H3K4me3_Pro; E049,H3K4me3_Pro; E050,H3K4me3_Pro; E051,H3K4me3_Pro; E055,H3K4me3_Pro; E056,H3K4me3_Pro; E057,H3K4me3_Pro; E058,H3K4me3_Pro; E061,H3K4me3_Pro; E062,H3K4me3_Pro; E063,H3K4me3_Pro; E066,H3K4me3_Pro; E067,H3K4me3_Pro; E068,H3K4me3_Pro; E069,H3K4me3_Pro; E071,H3K4me3_Pro; E072,H3K4me3_Pro; E073,H3K4me3_Pro; E075,H3K4me3_Pro; E077,H3K4me3_Pro; E078,H3K4me3_Pro; E079,H3K4me3_Pro; E080,H3K4me3_Pro; E084,H3K4me3_Pro; E085,H3K4me3_Pro; E086,H3K4me3_Pro; E088,H3K4me3_Pro; E089,H3K4me3_Pro; E091,H3K4me3_Pro; E092,H3K4me3_Pro; E096,H3K4me3_Pro; E100,H3K4me3_Pro; E101,H3K4me3_Pro; E102,H3K4me3_Pro; E103,H3K4me3_Pro; E104,H3K4me3_Pro; E105,H3K4me3_Pro; E107,H3K4me3_Pro; E108,H3K4me3_Pro; E110,H3K4me3_Pro; E111,H3K4me3_Pro; E112,H3K4me3_Pro; E114,H3K4me3_Pro; E116,H3K4me3_Pro; E118,H3K4me3_Pro; E119,H3K4me3_Pro; E122,H3K4me3_Pro; E124,H3K4me3_Pro; E127,H3K4me3_Pro; E128,H3K4me3_Pro; E001,H3K9ac_Pro; E003,H3K9ac_Pro; E006,H3K9ac_Pro; E008,H3K9ac_Pro; E015,H3K9ac_Pro; E017,H3K9ac_Pro; E019,H3K9ac_Pro; E020,H3K9ac_Pro; E023,H3K9ac_Pro; E025,H3K9ac_Pro; E026,H3K9ac_Pro; E027,H3K9ac_Pro; E049,H3K9ac_Pro; E063,H3K9ac_Pro; E066,H3K9ac_Pro; E072,H3K9ac_Pro; E075,H3K9ac_Pro; E076,H3K9ac_Pro; E086,H3K9ac_Pro; E088,H3K9ac_Pro; E101,H3K9ac_Pro; E102,H3K9ac_Pro; E103,H3K9ac_Pro; E110,H3K9ac_Pro; E111,H3K9ac_Pro; E114,H3K9ac_Pro; E116,H3K9ac_Pro; E118,H3K9ac_Pro; E119,H3K9ac_Pro; E122,H3K9ac_Pro; E124,H3K9ac_Pro; E127,H3K9ac_Pro; E128,H3K9ac_Pro; E006,H3K27ac_Enh; E012,H3K27ac_Enh; E013,H3K27ac_Enh; E014,H3K27ac_Enh; [illegible]	ESC, ESDR, LNG, IPSC, FAT, STRM, BRST, BLD, SKIN, BRN, GI, ADRL, KID, MUS, THYM, LIV, VAS	ESDR, IPSC, ESC, BLD, BRN, LIV, GI, MUS, PLCNT, OVRY	E003;E004;E006;E007;E008;E017;E022;E028;E029;E032;E046;E050;E051;E055;E056;E057;E059;E080;E081;E083;E084;E086;E088;E089;E090;E091;E092;E097;E098;E100;E119;E122;E126	.	Pax-4, Zic

rs3801805	7	26,897,640	Transcript	INTRONIC		68.28	24.80	36.92			1	1	44.0918	139	13991	H3K9ac, Histone, Histone 3 Lysine 9 Acetylation H3K4me3, Histone, Histone 3 Lysine 4 Tri-Methylation H3K36me3, Histone, Histone 3 Lysine 36 Tri-Methylation H3K4me2, Histone, Histone 3 Lysine 4 Di-Methylation H3K27me3, Histone, Histone 3 Lysine 27 Tri-Methylation H3K27ac, Histone, Histone 3 Lysine 27 Acetylation H2AZ, Histone, Histone 2A variant Z	E001,10_TssBiv; E002,10_TssBiv; E003,12_EnhBiv; E005,12_EnhBiv; E006,2_TssAFlnk; E008,10_TssBiv; E009,12_EnhBiv; E010,11_BivFlnk; E011,12_EnhBiv; E012,12_EnhBiv; E013,10_TssBiv; E014,11_BivFlnk; E015,10_TssBiv; E016,11_BivFlnk; E017,2_TssAFlnk; E018,10_TssBiv; E019,11_BivFlnk; E020,10_TssBiv; E021,12_EnhBiv; E022,11_BivFlnk; E023,1_TssA; E024,12_EnhBiv; E025,1_TssA; E026,1_TssA; E027,1_TssA; E028,1_TssA; E029,12_EnhBiv; E030,2_TssAFlnk; E031,7_Enh; E032,2_TssAFlnk; E034,12_EnhBiv; E035,2_TssAFlnk; E036,2_TssAFlnk; E046,2_TssAFlnk; E050,2_TssAFlnk; E051,2_TssAFlnk; E055,11_BivFlnk; E056,2_TssAFlnk; E057,1_TssA; E058,1_TssA; E061,2_TssAFlnk; E063,2_TssAFlnk; E067,10_TssBiv; E070,12_EnhBiv; E072,10_TssBiv; E075,2_TssAFlnk; E078,1_TssA; E079,7_Enh; E080,2_TssAFlnk; E084,12_EnhBiv; E085,12_EnhBiv; E086,10_TssBiv; E088,12_EnhBiv; E089,12_EnhBiv; E091,12_EnhBiv; E092,12_EnhBiv; E096,2_TssAFlnk; E097,12_EnhBiv; E099,7_Enh; E100,1_TssA; E101,2_TssAFlnk; E102,2_TssAFlnk; E103,1_TssA; E107,10_TssBiv; E111,1_TssA; E116,1_TssA; E118,1_TssA; E119,2_TssAFlnk; E122,2_TssAFlnk; [illegible]	E001,2_PromU; E002,23_PromBiv; E003,23_PromBiv; E004,23_PromBiv; E005,23_PromBiv; E006,3_PromD1; E007,23_PromBiv; E008,23_PromBiv; E009,23_PromBiv; E010,2_PromU; E011,23_PromBiv; E012,23_PromBiv; E013,2_PromU; E014,2_PromU; E015,2_PromU; E016,2_PromU; E017,3_PromD1; E018,2_PromU; E019,2_PromU; E020,2_PromU; E021,23_PromBiv; E022,23_PromBiv; E023,3_PromD1; E024,23_PromBiv; E025,3_PromD1; E026,4_PromD2; E027,3_PromD1; E028,3_PromD1; E029,4_PromD2; E030,3_PromD1; E031,4_PromD2; E032,3_PromD1; E033,4_PromD2; E034,4_PromD2; E035,3_PromD1; E036,3_PromD1; E037,23_PromBiv; E038,23_PromBiv; E039,23_PromBiv; E040,23_PromBiv; E041,23_PromBiv; E042,23_PromBiv; E043,23_PromBiv; E044,23_PromBiv; E045,23_PromBiv; E046,3_PromD1; E047,23_PromBiv; E048,23_PromBiv; E049,4_PromD2; E050,3_PromD1; E051,3_PromD1; E053,23_PromBiv; E054,23_PromBiv; E055,3_PromD1; E056,3_PromD1; E057,3_PromD1; E058,3_PromD1; E059,4_PromD2; E061,3_PromD1; E062,4_PromD2; E063,3_PromD1; E065,22_PromP; E066,3_PromD1; E067,2_PromU; E068,4_PromD2; E069,23_PromBiv; E070,23_PromBiv; E071,4_PromD2; E072,23_PromBiv; E073,23_PromBiv; E074,23_PromBiv; E075,3_PromD1; E076,4_PromD2; E077,3_PromD1; E078,3_PromD1; E079,3_PromD1; E080,3_PromD1; E081,23_PromBiv; E082,23_PromBiv; E083,4_PromD2; E084,3_PromD1; E085,3_PromD1; E086,3_PromD1; E087,4_PromD2; E088,3_PromD1; E089,2_PromU; E090,23_PromBiv; E091,4_PromD2; E092,3_PromD1; E093,23_PromBiv; E094,4_PromD2; E095,4_PromD2; E096,3_PromD1; E097,2_PromU; E098,4_PromD2; E099,3_PromD1; E100,4_PromD2; E101,3_PromD1; E102,3_PromD1; E103,3_PromD1; E104,4_PromD2; E105,4_PromD2; E106,3_PromD1; E107,4_PromD2; E108,4_PromD2; E109,3_PromD1; E110,3_PromD1; E111,3_PromD1; E112,23_PromBiv; E113,3_PromD1; E114,4_PromD2; E115,23_PromBiv; E116,4_PromD2; E117,4_PromD2; E118,2_PromU; E119,3_PromD1; E120,23_PromBiv; E122,3_PromD1; E123,4_PromD2; E124,3_PromD1; E125,4_PromD2; E126,4_PromD2; E127,3_PromD1; E128,3_PromD1; E129,23_PromBiv	E001,H3K4me1_Enh; E002,H3K4me1_Enh; E003,H3K4me1_Enh; E005,H3K4me1_Enh; E006,H3K4me1_Enh; E007,H3K4me1_Enh; E008,H3K4me1_Enh; E009,H3K4me1_Enh; E010,H3K4me1_Enh; E011,H3K4me1_Enh; E012,H3K4me1_Enh; E013,H3K4me1_Enh; E014,H3K4me1_Enh; E015,H3K4me1_Enh; E016,H3K4me1_Enh; E017,H3K4me1_Enh; E018,H3K4me1_Enh; E019,H3K4me1_Enh; E020,H3K4me1_Enh; E021,H3K4me1_Enh; E022,H3K4me1_Enh; E023,H3K4me1_Enh; E024,H3K4me1_Enh; E025,H3K4me1_Enh; E026,H3K4me1_Enh; E027,H3K4me1_Enh; E028,H3K4me1_Enh; E029,H3K4me1_Enh; E030,H3K4me1_Enh; E031,H3K4me1_Enh; E032,H3K4me1_Enh; E034,H3K4me1_Enh; E035,H3K4me1_Enh; E036,H3K4me1_Enh; E039,H3K4me1_Enh; E040,H3K4me1_Enh; E041,H3K4me1_Enh; E042,H3K4me1_Enh; E043,H3K4me1_Enh; E046,H3K4me1_Enh; E047,H3K4me1_Enh; E048,H3K4me1_Enh; E049,H3K4me1_Enh; E050,H3K4me1_Enh; E051,H3K4me1_Enh; E055,H3K4me1_Enh; E056,H3K4me1_Enh; E057,H3K4me1_Enh; E058,H3K4me1_Enh; E061,H3K4me1_Enh; E063,H3K4me1_Enh; E066,H3K4me1_Enh; E071,H3K4me1_Enh; E075,H3K4me1_Enh; E077,H3K4me1_Enh; E078,H3K4me1_Enh; E079,H3K4me1_Enh; E080,H3K4me1_Enh; E084,H3K4me1_Enh; E085,H3K4me1_Enh; E086,H3K4me1_Enh; E088,H3K4me1_Enh; E089,H3K4me1_Enh; E092,H3K4me1_Enh; E093,H3K4me1_Enh; E096,H3K4me1_Enh; E097,H3K4me1_Enh; E099,H3K4me1_Enh; E101,H3K4me1_Enh; E102,H3K4me1_Enh; E104,H3K4me1_Enh; E106,H3K4me1_Enh; E110,H3K4me1_Enh; E111,H3K4me1_Enh; E113,H3K4me1_Enh; E114,H3K4me1_Enh; E116,H3K4me1_Enh; E118,H3K4me1_Enh; E119,H3K4me1_Enh; E122,H3K4me1_Enh; E123,H3K4me1_Enh; E124,H3K4me1_Enh; E125,H3K4me1_Enh; E127,H3K4me1_Enh; E128,H3K4me1_Enh; E001,H3K4me3_Pro; E002,H3K4me3_Pro; E003,H3K4me3_Pro; E006,H3K4me3_Pro; E008,H3K4me3_Pro; E010,H3K4me3_Pro; E011,H3K4me3_Pro; E012,H3K4me3_Pro; E013,H3K4me3_Pro; E014,H3K4me3_Pro; E015,H3K4me3_Pro; E016,H3K4me3_Pro; E017,H3K4me3_Pro; E018,H3K4me3_Pro; E019,H3K4me3_Pro; E020,H3K4me3_Pro; E021,H3K4me3_Pro; E022,H3K4me3_Pro; E023,H3K4me3_Pro; E024,H3K4me3_Pro; E025,H3K4me3_Pro; E026,H3K4me3_Pro; E027,H3K4me3_Pro; E028,H3K4me3_Pro; E030,H3K4me3_Pro; E032,H3K4me3_Pro; E034,H3K4me3_Pro; E035,H3K4me3_Pro; E036,H3K4me3_Pro; E041,H3K4me3_Pro; E046,H3K4me3_Pro; E049,H3K4me3_Pro; E050,H3K4me3_Pro; E051,H3K4me3_Pro; E055,H3K4me3_Pro; E056,H3K4me3_Pro; E057,H3K4me3_Pro; E058,H3K4me3_Pro; E061,H3K4me3_Pro; E062,H3K4me3_Pro; E063,H3K4me3_Pro; E066,H3K4me3_Pro; E067,H3K4me3_Pro; E068,H3K4me3_Pro; E069,H3K4me3_Pro; E071,H3K4me3_Pro; E072,H3K4me3_Pro; E073,H3K4me3_Pro; E075,H3K4me3_Pro; E077,H3K4me3_Pro; E078,H3K4me3_Pro; E079,H3K4me3_Pro; E080,H3K4me3_Pro; E084,H3K4me3_Pro; E085,H3K4me3_Pro; E086,H3K4me3_Pro; E088,H3K4me3_Pro; E089,H3K4me3_Pro; E092,H3K4me3_Pro; E096,H3K4me3_Pro; E100,H3K4me3_Pro; E101,H3K4me3_Pro; E102,H3K4me3_Pro; E103,H3K4me3_Pro; E104,H3K4me3_Pro; E105,H3K4me3_Pro; E107,H3K4me3_Pro; E108,H3K4me3_Pro; E110,H3K4me3_Pro; E111,H3K4me3_Pro; E114,H3K4me3_Pro; E116,H3K4me3_Pro; E118,H3K4me3_Pro; E119,H3K4me3_Pro; E122,H3K4me3_Pro; E124,H3K4me3_Pro; E127,H3K4me3_Pro; E128,H3K4me3_Pro; E001,H3K9ac_Pro; E003,H3K9ac_Pro; E006,H3K9ac_Pro; E008,H3K9ac_Pro; E015,H3K9ac_Pro; E017,H3K9ac_Pro; E019,H3K9ac_Pro; E020,H3K9ac_Pro; E023,H3K9ac_Pro; E025,H3K9ac_Pro; E026,H3K9ac_Pro; E027,H3K9ac_Pro; E038,H3K9ac_Pro; E049,H3K9ac_Pro; E063,H3K9ac_Pro; E066,H3K9ac_Pro; E072,H3K9ac_Pro; E075,H3K9ac_Pro; E076,H3K9ac_Pro; E086,H3K9ac_Pro; E088,H3K9ac_Pro; E101,H3K9ac_Pro; E102,H3K9ac_Pro; E103,H3K9ac_Pro; E110,H3K9ac_Pro; E111,H3K9ac_Pro; E114,H3K9ac_Pro; E116,H3K9ac_Pro; E118,H3K9ac_Pro; E119,H3K9ac_Pro; E122,H3K9ac_Pro; E124,H3K9ac_Pro; E127,H3K9ac_Pro; E128,H3K9ac_Pro; E006,H3K27ac_Enh; E012,H3K27ac_Enh; E013,H3K27ac_Enh; E014,H3K27ac_Enh; E017,H3K27ac_Enh; E026,H3K27ac_Enh; E032,H3K27ac_Enh; [illegible]	ESC, ESDR, LNG, IPSC, FAT, STRM, BRST, BLD, SKIN, BRN, GI, ADRL, KID, MUS, LIV, VAS	ESC, ESDR, IPSC, BLD, BRN, GI, LNG, MUS, PLCNT, OVRY	E003;E007;E008;E017;E021;E028;E032;E051;E057;E080;E085;E086;E088;E089;E092;E119;E122	.	ERalpha-a, PTF1-beta, RAR
rs10486485	7	26,900,356	Transcript	INTRONIC		15.88	11.52	2.00						3849	16707	H3K9me1, Histone, Histone 3 Lysine 9 mono-methylation H3K4me1, Histone, Histone 3 Lysine 4 Mono-Methylation H3K27me3, Histone, Histone 3 Lysine 27 Tri-Methylation	.	.	E014,H3K4me1_Enh; E018,H3K4me1_Enh; E019,H3K4me1_Enh; E024,H3K4me1_Enh; E025,H3K4me1_Enh; E027,H3K4me1_Enh; E028,H3K4me1_Enh; E029,H3K4me1_Enh; E030,H3K4me1_Enh; E031,H3K4me1_Enh; E032,H3K4me1_Enh; E035,H3K4me1_Enh; E036,H3K4me1_Enh; E037,H3K4me1_Enh; E040,H3K4me1_Enh; E044,H3K4me1_Enh; E050,H3K4me1_Enh; E051,H3K4me1_Enh; E057,H3K4me1_Enh; E058,H3K4me1_Enh; E061,H3K4me1_Enh; E063,H3K4me1_Enh; E066,H3K4me1_Enh; E075,H3K4me1_Enh; E077,H3K4me1_Enh; E078,H3K4me1_Enh; E084,H3K4me1_Enh; E085,H3K4me1_Enh; E088,H3K4me1_Enh; E101,H3K4me1_Enh; E102,H3K4me1_Enh; E110,H3K4me1_Enh; E111,H3K4me1_Enh; E114,H3K4me1_Enh; E117,H3K4me1_Enh; E119,H3K4me1_Enh; E124,H3K4me1_Enh; E127,H3K4me1_Enh; E128,H3K4me1_Enh; E028,H3K4me3_Pro; E040,H3K4me3_Pro; E071,H3K4me3_Pro; E078,H3K4me3_Pro; E101,H3K4me3_Pro; E110,H3K4me3_Pro; E111,H3K4me3_Pro; E049,H3K9ac_Pro; E063,H3K9ac_Pro; E076,H3K9ac_Pro; E088,H3K9ac_Pro; E101,H3K9ac_Pro; E102,H3K9ac_Pro; E110,H3K9ac_Pro; E119,H3K9ac_Pro; E127,H3K9ac_Pro; E058,H3K27ac_Enh; E063,H3K27ac_Enh; E075,H3K27ac_Enh; E078,H3K27ac_Enh; E101,H3K27ac_Enh; E102,H3K27ac_Enh; E111,H3K27ac_Enh; E119,H3K27ac_Enh; E127,H3K27ac_Enh	.	.	E004;E090	.	.
rs3801803	7	26,901,740	Transcript	INTRONIC		7.24	10.28	7.12			1	1	37.5219	2465	18091	H3K4me1, Histone, Histone 3 Lysine 4 Mono-Methylation H3K27me3, Histone, Histone 3 Lysine 27 Tri-Methylation	E030,7_Enh; E031,7_Enh; E036,7_Enh; E050,7_Enh; E051,7_Enh; E116,1_TssA; E119,1_TssA; E124,7_Enh; E127,7_Enh	E025,12_TxEnhW; E028,12_TxEnhW; E030,12_TxEnhW; E031,12_TxEnhW; E035,12_TxEnhW; E036,12_TxEnhW; E051,12_TxEnhW; E058,12_TxEnhW; E078,12_TxEnhW; E107,19_DNase; E119,12_TxEnhW; E124,12_TxEnhW; E127,12_TxEnhW	E001,H3K4me1_Enh; E014,H3K4me1_Enh; E025,H3K4me1_Enh; E026,H3K4me1_Enh; E027,H3K4me1_Enh; E028,H3K4me1_Enh; E030,H3K4me1_Enh; E031,H3K4me1_Enh; E035,H3K4me1_Enh; E036,H3K4me1_Enh; E037,H3K4me1_Enh; E038,H3K4me1_Enh; E043,H3K4me1_Enh; E044,H3K4me1_Enh; E047,H3K4me1_Enh; E048,H3K4me1_Enh; E049,H3K4me1_Enh; E050,H3K4me1_Enh; E051,H3K4me1_Enh; E052,H3K4me1_Enh; E057,H3K4me1_Enh; E058,H3K4me1_Enh; E061,H3K4me1_Enh; E063,H3K4me1_Enh; E075,H3K4me1_Enh; E077,H3K4me1_Enh; E078,H3K4me1_Enh; E084,H3K4me1_Enh; E085,H3K4me1_Enh; E088,H3K4me1_Enh; E099,H3K4me1_Enh; E101,H3K4me1_Enh; E102,H3K4me1_Enh; E110,H3K4me1_Enh; E114,H3K4me1_Enh; E115,H3K4me1_Enh; E117,H3K4me1_Enh; E122,H3K4me1_Enh; E124,H3K4me1_Enh; E127,H3K4me1_Enh; E025,H3K4me3_Pro; E028,H3K4me3_Pro; E040,H3K4me3_Pro; E051,H3K4me3_Pro; E058,H3K4me3_Pro; E071,H3K4me3_Pro; E072,H3K4me3_Pro; E076,H3K4me3_Pro; E078,H3K4me3_Pro; E088,H3K4me3_Pro; E102,H3K4me3_Pro; E110,H3K4me3_Pro; E111,H3K4me3_Pro; E114,H3K4me3_Pro; E116,H3K4me3_Pro; E119,H3K4me3_Pro; E124,H3K4me3_Pro; E127,H3K4me3_Pro; E025,H3K9ac_Pro; E063,H3K9ac_Pro; E066,H3K9ac_Pro; E076,H3K9ac_Pro; E077,H3K9ac_Pro; E086,H3K9ac_Pro; E088,H3K9ac_Pro; E102,H3K9ac_Pro; E110,H3K9ac_Pro; E127,H3K9ac_Pro; E058,H3K27ac_Enh; E063,H3K27ac_Enh; E074,H3K27ac_Enh; E076,H3K27ac_Enh; E078,H3K27ac_Enh; E080,H3K27ac_Enh; E101,H3K27ac_Enh; E102,H3K27ac_Enh; E111,H3K27ac_Enh; E119,H3K27ac_Enh; E127,H3K27ac_Enh	BLD, BRST	BLD, SKIN	E028;E057;E090	MAFK	.
rs73069539	7	26,903,661	RegulatoryFeature	REGULATORY	0.44	52.20	15.88	121.80	3	0.02	4	4	44.4919	544	20012	H3K9ac, Histone, Histone 3 Lysine 9 Acetylation DNase1, Open Chromatin, DNase1 Hypersensitive Site E2F6, Transcription Factor, E2F6 Transcription Factor Binding H3K4me3, Histone, Histone 3 Lysine 4 Tri-Methylation H3K4me2, Histone, Histone 3 Lysine 4 Di-Methylation H3K27me3, Histone, Histone 3 Lysine 27 Tri-Methylation H2AZ, Histone, Histone 2A variant Z H3K27ac, Histone, Histone 3 Lysine 27 Acetylation H4K91ac, Histone, Histone 4 Lysine 91 Acetylation Promoter Associated, Regulatory Feature, Promoter like regulatory feature PolII, Polymerase, RNA Polymerase II	E001,1_TssA; E002,1_TssA; E003,11_BivFlnk; E004,1_TssA; E005,2_TssAFlnk; E006,1_TssA; E007,1_TssA; E008,1_TssA; E009,1_TssA; E010,1_TssA; E011,1_TssA; E012,10_TssBiv; E013,1_TssA; E014,10_TssBiv; E015,1_TssA; E016,10_TssBiv; E017,1_TssA; E018,1_TssA; E019,10_TssBiv; E020,10_TssBiv; E021,1_TssA; E022,1_TssA; E023,1_TssA; E024,1_TssA; E025,1_TssA; E026,1_TssA; E027,1_TssA; E028,1_TssA; E029,1_TssA; E030,1_TssA; E031,2_TssAFlnk; E032,1_TssA; E033,2_TssAFlnk; E034,1_TssA; E035,2_TssAFlnk; E036,1_TssA; E037,1_TssA; E038,1_TssA; E039,1_TssA; E040,1_TssA; E041,2_TssAFlnk; E042,2_TssAFlnk; E043,2_TssAFlnk; E044,1_TssA; E045,1_TssA; E046,1_TssA; E047,2_TssAFlnk; E048,1_TssA; E049,2_TssAFlnk; E050,2_TssAFlnk; E051,1_TssA; E052,1_TssA; E053,10_TssBiv; E055,1_TssA; E056,1_TssA; E057,1_TssA; E058,1_TssA; E059,10_TssBiv; E061,1_TssA; E062,2_TssAFlnk; E063,2_TssAFlnk; E065,1_TssA; E066,1_TssA; E067,1_TssA; E068,10_TssBiv; E069,1_TssA; E070,10_TssBiv; E071,1_TssA; E072,1_TssA; E073,1_TssA; E074,1_TssA; E075,1_TssA; E076,10_TssBiv; E077,1_TssA; E078,1_TssA; E079,1_TssA; E080,1_TssA; E082,10_TssBiv; [illegible]	E001,3_PromD1; E002,2_PromU; E003,2_PromU; E004,2_PromU; E005,3_PromD1; E006,3_PromD1; E007,3_PromD1; E008,3_PromD1; E009,3_PromD1; E010,3_PromD1; E011,1_TssA; E012,2_PromU; E013,3_PromD1; E014,1_TssA; E015,3_PromD1; E016,1_TssA; E017,3_PromD1; E018,1_TssA; E019,1_TssA; E020,2_PromU; E021,1_TssA; E022,3_PromD1; E023,3_PromD1; E024,2_PromU; E025,3_PromD1; E026,3_PromD1; E027,3_PromD1; E028,3_PromD1; E029,3_PromD1; E030,3_PromD1; E031,3_PromD1; E032,3_PromD1; E033,3_PromD1; E034,3_PromD1; E035,3_PromD1; E036,3_PromD1; E037,3_PromD1; E038,3_PromD1; E039,3_PromD1; E040,3_PromD1; E041,3_PromD1; E042,3_PromD1; E043,3_PromD1; E044,3_PromD1; E045,3_PromD1; E046,3_PromD1; E047,3_PromD1; E048,3_PromD1; E049,3_PromD1; E050,3_PromD1; E051,3_PromD1; E052,3_PromD1; E053,3_PromD1; E054,2_PromU; E055,3_PromD1; E056,3_PromD1; E057,3_PromD1; E058,3_PromD1; E059,3_PromD1; E061,3_PromD1; E062,3_PromD1; E063,3_PromD1; E065,3_PromD1; E066,3_PromD1; E067,3_PromD1; E068,3_PromD1; E069,3_PromD1; E070,3_PromD1; E071,3_PromD1; E072,3_PromD1; E073,3_PromD1; E074,3_PromD1; E075,3_PromD1; E076,3_PromD1; E077,3_PromD1; E078,3_PromD1; E079,3_PromD1; E080,3_PromD1; E081,2_PromU; E082,2_PromU; E083,2_PromU; E084,3_PromD1; E085,3_PromD1; E086,3_PromD1; E087,3_PromD1; E088,3_PromD1; E089,3_PromD1; E090,3_PromD1; E091,3_PromD1; E092,3_PromD1; E093,3_PromD1; E094,3_PromD1; E095,3_PromD1; E096,3_PromD1; E097,3_PromD1; E098,3_PromD1; E099,3_PromD1; E100,3_PromD1; E101,3_PromD1; E102,3_PromD1; E103,3_PromD1; E104,3_PromD1; E105,3_PromD1; E106,3_PromD1; E107,2_PromU; E108,3_PromD1; E109,3_PromD1; E110,3_PromD1; E111,3_PromD1; E112,3_PromD1; E113,3_PromD1; E114,3_PromD1; E115,3_PromD1; E116,3_PromD1; E117,3_PromD1; E118,3_PromD1; E119,3_PromD1; E120,3_PromD1; E121,3_PromD1; E122,3_PromD1; E123,3_PromD1; E124,3_PromD1; E125,3_PromD1; E126,3_PromD1; E127,3_PromD1; E128,3_PromD1; E129,3_PromD1	E001,H3K4me3_Pro; E002,H3K4me3_Pro; E003,H3K4me3_Pro; E004,H3K4me3_Pro; E005,H3K4me3_Pro; E006,H3K4me3_Pro; E007,H3K4me3_Pro; E008,H3K4me3_Pro; E009,H3K4me3_Pro; E010,H3K4me3_Pro; E011,H3K4me3_Pro; E012,H3K4me3_Pro; E013,H3K4me3_Pro; E014,H3K4me3_Pro; E015,H3K4me3_Pro; E016,H3K4me3_Pro; E017,H3K4me3_Pro; E018,H3K4me3_Pro; E019,H3K4me3_Pro; E020,H3K4me3_Pro; E021,H3K4me3_Pro; E022,H3K4me3_Pro; E023,H3K4me3_Pro; E024,H3K4me3_Pro; E025,H3K4me3_Pro; E026,H3K4me3_Pro; E027,H3K4me3_Pro; E028,H3K4me3_Pro; E029,H3K4me3_Pro; E030,H3K4me3_Pro; E031,H3K4me3_Pro; E032,H3K4me3_Pro; E033,H3K4me3_Pro; E034,H3K4me3_Pro; E035,H3K4me3_Pro; E036,H3K4me3_Pro; E037,H3K4me3_Pro; E038,H3K4me3_Pro; E039,H3K4me3_Pro; E040,H3K4me3_Pro; E041,H3K4me3_Pro; E042,H3K4me3_Pro; E043,H3K4me3_Pro; E044,H3K4me3_Pro; E045,H3K4me3_Pro; E046,H3K4me3_Pro; E047,H3K4me3_Pro; E048,H3K4me3_Pro; E049,H3K4me3_Pro; E050,H3K4me3_Pro; E051,H3K4me3_Pro; E052,H3K4me3_Pro; E053,H3K4me3_Pro; E054,H3K4me3_Pro; E055,H3K4me3_Pro; E056,H3K4me3_Pro; E057,H3K4me3_Pro; E058,H3K4me3_Pro; E059,H3K4me3_Pro; E061,H3K4me3_Pro; E062,H3K4me3_Pro; E063,H3K4me3_Pro; E065,H3K4me3_Pro; E066,H3K4me3_Pro; E067,H3K4me3_Pro; E068,H3K4me3_Pro; E069,H3K4me3_Pro; E070,H3K4me3_Pro; E071,H3K4me3_Pro; E072,H3K4me3_Pro; E073,H3K4me3_Pro; E074,H3K4me3_Pro; E075,H3K4me3_Pro; E076,H3K4me3_Pro; E077,H3K4me3_Pro; E078,H3K4me3_Pro; E079,H3K4me3_Pro; E080,H3K4me3_Pro; E081,H3K4me3_Pro; E082,H3K4me3_Pro; E084,H3K4me3_Pro; E085,H3K4me3_Pro; E086,H3K4me3_Pro; E087,H3K4me3_Pro; E088,H3K4me3_Pro; E089,H3K4me3_Pro; E090,H3K4me3_Pro; E091,H3K4me3_Pro; E092,H3K4me3_Pro; E093,H3K4me3_Pro; E094,H3K4me3_Pro; E095,H3K4me3_Pro; E096,H3K4me3_Pro; E097,H3K4me3_Pro; E098,H3K4me3_Pro; E099,H3K4me3_Pro; E100,H3K4me3_Pro; E101,H3K4me3_Pro; E102,H3K4me3_Pro; E103,H3K4me3_Pro; E104,H3K4me3_Pro; E105,H3K4me3_Pro; E106,H3K4me3_Pro; E107,H3K4me3_Pro; E108,H3K4me3_Pro; E109,H3K4me3_Pro; E110,H3K4me3_Pro; E111,H3K4me3_Pro; E112,H3K4me3_Pro; E113,H3K4me3_Pro; E114,H3K4me3_Pro; E115,H3K4me3_Pro; E116,H3K4me3_Pro; E117,H3K4me3_Pro; E118,H3K4me3_Pro; E119,H3K4me3_Pro; E120,H3K4me3_Pro; E121,H3K4me3_Pro; E122,H3K4me3_Pro; E123,H3K4me3_Pro; E124,H3K4me3_Pro; E125,H3K4me3_Pro; E126,H3K4me3_Pro; E127,H3K4me3_Pro; E128,H3K4me3_Pro; E129,H3K4me3_Pro; E001,H3K9ac_Pro; E003,H3K9ac_Pro; E004,H3K9ac_Pro; E007,H3K9ac_Pro; E008,H3K9ac_Pro; E011,H3K9ac_Pro; E014,H3K9ac_Pro; E015,H3K9ac_Pro; E016,H3K9ac_Pro; E017,H3K9ac_Pro; E019,H3K9ac_Pro; E020,H3K9ac_Pro; E023,H3K9ac_Pro; E025,H3K9ac_Pro; E026,H3K9ac_Pro; E027,H3K9ac_Pro; E038,H3K9ac_Pro; E047,H3K9ac_Pro; E049,H3K9ac_Pro; E052,H3K9ac_Pro; E062,H3K9ac_Pro; E063,H3K9ac_Pro; E066,H3K9ac_Pro; E067,H3K9ac_Pro; E068,H3K9ac_Pro; E069,H3K9ac_Pro; E072,H3K9ac_Pro; E073,H3K9ac_Pro; E074,H3K9ac_Pro; E075,H3K9ac_Pro; E076,H3K9ac_Pro; E077,H3K9ac_Pro; E083,H3K9ac_Pro; E086,H3K9ac_Pro; E087,H3K9ac_Pro; E088,H3K9ac_Pro; E101,H3K9ac_Pro; E102,H3K9ac_Pro; E103,H3K9ac_Pro; E107,H3K9ac_Pro; E108,H3K9ac_Pro; E110,H3K9ac_Pro; E111,H3K9ac_Pro; E114,H3K9ac_Pro; E115,H3K9ac_Pro; E116,H3K9ac_Pro; E117,H3K9ac_Pro; E118,H3K9ac_Pro; E119,H3K9ac_Pro; E120,H3K9ac_Pro; E121,H3K9ac_Pro; E122,H3K9ac_Pro; E123,H3K9ac_Pro; E124,H3K9ac_Pro; E125,H3K9ac_Pro; E126,H3K9ac_Pro; E127,H3K9ac_Pro; E128,H3K9ac_Pro; E005,H3K4me1_Enh; E007,H3K4me1_Enh; E009,H3K4me1_Enh; E010,H3K4me1_Enh; E012,H3K4me1_Enh; E014,H3K4me1_Enh; E015,H3K4me1_Enh; E016,H3K4me1_Enh; E018,H3K4me1_Enh; E019,H3K4me1_Enh; E020,H3K4me1_Enh; E030,H3K4me1_Enh; E031,H3K4me1_Enh; E033,H3K4me1_Enh; E034,H3K4me1_Enh; E035,H3K4me1_Enh; E036,H3K4me1_Enh; E037,H3K4me1_Enh; E038,H3K4me1_Enh; E039,H3K4me1_Enh; [illegible]	ESC, ESDR, LNG, IPSC, FAT, STRM, BRST, BLD, MUS, BRN, SKIN, VAS, LIV, GI, ADRL, KID, PANC, PLCNT, THYM, HRT, OVRY, SPLN, CRVX, BONE	HRT	E003;E006;E007;E008;E017;E028;E032;E033;E034;E046;E050;E051;E055;E056;E057;E059;E080;E083;E084;E086;E088;E089;E090;E091;E092;E093;E097;E098;E109;E114;E116;E117;E118;E119;E120;E121;E122;E123;E124;E125;E126;E128	EGR1, TAF1	AP-1, BCL, E2F, EWSR1-FLI1, Egr-1, Ets, GR, Klf4, Klf7, MOVO-B, PU.1, Pax-5, SETDB1, SP1, SPIB, STAT, TATA, ZNF263
rs17323934	7	26,904,330	RegulatoryFeature	REGULATORY	1.78	15.72	4.00	30.00			52	86	448.4156	33	20681	DNase1, Open Chromatin, DNase1 Hypersensitive Site HEY1, Transcription Factor, HEY1 Transcription Factor Binding H3K4me3, Histone, Histone 3 Lysine 4 Tri-Methylation ELF1, Transcription Factor, ELF1 Transcription Factor Binding Nfe2, Transcription Factor, Nfe2 TF binding H3K27ac, Histone, Histone 3 Lysine 27 Acetylation Ini1, Transcription Factor, Ini1 Transcription Factor Binding H3K36ac, Histone, Histone 3 Lysine 36 Acetylation H3K18ac, Histone, Histone 3 Lysine 18 Acetylation TAF1, Transcription Factor, TAF1 Transcription Factor Binding THAP1, Transcription Factor, THAP1 Transcription Factor Binding Max, Transcription Factor, Max TF binding PolII, Polymerase, RNA Polymerase II Cfos, Transcription Factor, Cfos TF binding H2BK5ac, Histone, Histone 2B Lysine 5 Acetylation H2BK120ac, Histone, Histone 2B Lysine 120 Acetylation H3K9ac, Histone, Histone 3 Lysine 9 Acetylation FAIRE, Open Chromatin, Formaldehyde-Assisted Isolation of Regulatory Elements ATF3, Transcription Factor, ATF3 Transcription Factor Binding E2F6, Transcription Factor, E2F6 Transcription Factor Binding Yy1, Transcription Factor, Yy1 Transcription Factor Binding Ap2gamma, Transcription Factor, Ap2gamma Transcription Factor Binding USF1, Transcription Factor, USF1 Transcription Factor Binding FOSL2, Transcription Factor, FOSL2 Transcription Factor Binding H3K4me2, Histone, Histone 3 Lysine 4 Di-Methylation H3K27me3, Histone, Histone 3 Lysine 27 Tri-Methylation H2AZ, Histone, Histone 2A variant Z H4K91ac, Histone, Histone 4 Lysine 91 Acetylation Promoter Associated, Regulatory Feature, Promoter like regulatory feature	E001,1_TssA; E002,1_TssA; E003,1_TssA; E004,1_TssA; E005,1_TssA; E006,1_TssA; E007,1_TssA; E008,1_TssA; E009,1_TssA; E010,1_TssA; E011,1_TssA; E012,10_TssBiv; E013,1_TssA; E014,1_TssA; E015,1_TssA; E016,1_TssA; E017,1_TssA; E018,1_TssA; E019,1_TssA; E020,1_TssA; E021,1_TssA; E022,1_TssA; E023,1_TssA; E024,1_TssA; E025,1_TssA; E026,1_TssA; E027,1_TssA; E028,1_TssA; E029,1_TssA; E030,1_TssA; E031,1_TssA; E032,1_TssA; E033,1_TssA; E034,11_BivFlnk; E035,1_TssA; E036,1_TssA; E037,1_TssA; E038,1_TssA; E039,2_TssAFlnk; E040,1_TssA; E041,1_TssA; E042,3_TxFlnk; E043,2_TssAFlnk; E044,1_TssA; E045,2_TssAFlnk; E046,1_TssA; E047,1_TssA; E048,1_TssA; E049,1_TssA; E050,1_TssA; E051,1_TssA; E052,1_TssA; E053,10_TssBiv; E054,10_TssBiv; E055,1_TssA; E056,1_TssA; E057,1_TssA; E058,1_TssA; E059,1_TssA; E061,1_TssA; E062,2_TssAFlnk; E063,1_TssA; E065,1_TssA; E066,1_TssA; E067,1_TssA; E068,10_TssBiv; E069,1_TssA; E070,10_TssBiv; E071,1_TssA; E072,1_TssA; E073,1_TssA; E074,1_TssA; E075,1_TssA; E076,1_TssA; E077,1_TssA; E078,1_TssA; E079,1_TssA; E080,1_TssA; E081,12_EnhBiv; E082,10_TssBiv; E083,12_EnhBiv; E084,1_TssA; E085,1_TssA; E086,1_TssA; E087,1_TssA; E088,10_TssBiv; E089,10_TssBiv; [illegible]	E001,1_TssA; E002,1_TssA; E003,1_TssA; E004,1_TssA; E005,1_TssA; E006,1_TssA; E007,1_TssA; E008,1_TssA; E009,1_TssA; E010,1_TssA; E011,1_TssA; E012,1_TssA; E013,1_TssA; E014,1_TssA; E015,1_TssA; E016,1_TssA; E017,1_TssA; E018,1_TssA; E019,1_TssA; E020,1_TssA; E021,1_TssA; E022,1_TssA; E023,1_TssA; E024,1_TssA; E025,1_TssA; E026,1_TssA; E027,1_TssA; E028,1_TssA; E029,1_TssA; E030,1_TssA; E031,1_TssA; E032,1_TssA; E033,1_TssA; E034,2_PromU; E035,1_TssA; E036,1_TssA; E037,2_PromU; E038,2_PromU; E039,3_PromD1; E040,2_PromU; E041,2_PromU; E042,3_PromD1; E043,2_PromU; E044,3_PromD1; E045,2_PromU; E046,2_PromU; E047,2_PromU; E048,2_PromU; E049,1_TssA; E050,1_TssA; E051,1_TssA; E052,1_TssA; E053,1_TssA; E054,2_PromU; E055,2_PromU; E056,1_TssA; E057,1_TssA; E058,1_TssA; E059,1_TssA; E061,1_TssA; E062,2_PromU; E063,1_TssA; E065,1_TssA; E066,1_TssA; E067,1_TssA; E068,1_TssA; E069,1_TssA; E070,1_TssA; E071,1_TssA; E072,1_TssA; E073,1_TssA; E074,1_TssA; E075,1_TssA; E076,1_TssA; E077,1_TssA; E078,1_TssA; E079,1_TssA; E080,1_TssA; E081,1_TssA; E082,2_PromU; E083,1_TssA; E084,1_TssA; E085,1_TssA; E086,1_TssA; E087,1_TssA; E088,1_TssA; E089,1_TssA; E090,1_TssA; E091,1_TssA; E092,1_TssA; E093,1_TssA; E094,1_TssA; E095,1_TssA; E096,1_TssA; E097,1_TssA; E098,1_TssA; E099,1_TssA; E100,1_TssA; E101,1_TssA; E102,1_TssA; E103,1_TssA; E104,1_TssA; E105,1_TssA; E106,1_TssA; E107,1_TssA; E108,1_TssA; E109,1_TssA; E110,1_TssA; E111,1_TssA; E112,2_PromU; E113,2_PromU; E114,1_TssA; E115,1_TssA; E116,2_PromU; E117,1_TssA; E118,1_TssA; E119,1_TssA; E120,1_TssA; E121,1_TssA; E122,1_TssA; E123,1_TssA; E124,1_TssA; E125,1_TssA; E126,1_TssA; E127,1_TssA; E128,1_TssA; E129,1_TssA	E001,H3K4me3_Pro; E002,H3K4me3_Pro; E003,H3K4me3_Pro; E004,H3K4me3_Pro; E005,H3K4me3_Pro; E006,H3K4me3_Pro; E007,H3K4me3_Pro; E008,H3K4me3_Pro; E009,H3K4me3_Pro; E010,H3K4me3_Pro; E011,H3K4me3_Pro; E012,H3K4me3_Pro; E013,H3K4me3_Pro; E014,H3K4me3_Pro; E015,H3K4me3_Pro; E016,H3K4me3_Pro; E017,H3K4me3_Pro; E018,H3K4me3_Pro; E019,H3K4me3_Pro; E020,H3K4me3_Pro; E021,H3K4me3_Pro; E022,H3K4me3_Pro; E023,H3K4me3_Pro; E024,H3K4me3_Pro; E025,H3K4me3_Pro; E026,H3K4me3_Pro; E027,H3K4me3_Pro; E028,H3K4me3_Pro; E029,H3K4me3_Pro; E030,H3K4me3_Pro; E031,H3K4me3_Pro; E032,H3K4me3_Pro; E033,H3K4me3_Pro; E034,H3K4me3_Pro; E035,H3K4me3_Pro; E036,H3K4me3_Pro; E037,H3K4me3_Pro; E038,H3K4me3_Pro; E039,H3K4me3_Pro; E040,H3K4me3_Pro; E041,H3K4me3_Pro; E042,H3K4me3_Pro; E043,H3K4me3_Pro; E044,H3K4me3_Pro; E045,H3K4me3_Pro; E046,H3K4me3_Pro; E047,H3K4me3_Pro; E048,H3K4me3_Pro; E049,H3K4me3_Pro; E050,H3K4me3_Pro; E051,H3K4me3_Pro; E052,H3K4me3_Pro; E053,H3K4me3_Pro; E054,H3K4me3_Pro; E055,H3K4me3_Pro; E056,H3K4me3_Pro; E057,H3K4me3_Pro; E058,H3K4me3_Pro; E059,H3K4me3_Pro; E061,H3K4me3_Pro; E062,H3K4me3_Pro; E063,H3K4me3_Pro; E065,H3K4me3_Pro; E066,H3K4me3_Pro; E067,H3K4me3_Pro; E068,H3K4me3_Pro; E069,H3K4me3_Pro; E070,H3K4me3_Pro; E071,H3K4me3_Pro; E072,H3K4me3_Pro; E073,H3K4me3_Pro; E074,H3K4me3_Pro; E075,H3K4me3_Pro; E076,H3K4me3_Pro; E077,H3K4me3_Pro; E078,H3K4me3_Pro; E079,H3K4me3_Pro; E080,H3K4me3_Pro; E081,H3K4me3_Pro; E082,H3K4me3_Pro; E083,H3K4me3_Pro; E084,H3K4me3_Pro; E085,H3K4me3_Pro; E086,H3K4me3_Pro; E087,H3K4me3_Pro; E088,H3K4me3_Pro; E089,H3K4me3_Pro; E090,H3K4me3_Pro; E091,H3K4me3_Pro; E092,H3K4me3_Pro; E093,H3K4me3_Pro; E094,H3K4me3_Pro; E095,H3K4me3_Pro; E096,H3K4me3_Pro; E097,H3K4me3_Pro; E098,H3K4me3_Pro; E099,H3K4me3_Pro; E100,H3K4me3_Pro; E101,H3K4me3_Pro; E102,H3K4me3_Pro; E103,H3K4me3_Pro; E104,H3K4me3_Pro; E105,H3K4me3_Pro; E106,H3K4me3_Pro; E107,H3K4me3_Pro; E108,H3K4me3_Pro; E109,H3K4me3_Pro; E110,H3K4me3_Pro; E111,H3K4me3_Pro; E112,H3K4me3_Pro; E113,H3K4me3_Pro; E114,H3K4me3_Pro; E115,H3K4me3_Pro; E116,H3K4me3_Pro; E117,H3K4me3_Pro; E118,H3K4me3_Pro; E119,H3K4me3_Pro; E120,H3K4me3_Pro; E121,H3K4me3_Pro; E122,H3K4me3_Pro; E123,H3K4me3_Pro; E124,H3K4me3_Pro; E125,H3K4me3_Pro; E126,H3K4me3_Pro; E127,H3K4me3_Pro; E128,H3K4me3_Pro; E129,H3K4me3_Pro; E001,H3K9ac_Pro; E003,H3K9ac_Pro; E004,H3K9ac_Pro; E006,H3K9ac_Pro; E007,H3K9ac_Pro; E008,H3K9ac_Pro; E011,H3K9ac_Pro; E014,H3K9ac_Pro; E015,H3K9ac_Pro; E016,H3K9ac_Pro; E017,H3K9ac_Pro; E018,H3K9ac_Pro; E019,H3K9ac_Pro; E020,H3K9ac_Pro; E023,H3K9ac_Pro; E025,H3K9ac_Pro; E026,H3K9ac_Pro; E027,H3K9ac_Pro; E038,H3K9ac_Pro; E047,H3K9ac_Pro; E049,H3K9ac_Pro; E052,H3K9ac_Pro; E062,H3K9ac_Pro; E063,H3K9ac_Pro; E066,H3K9ac_Pro; E067,H3K9ac_Pro; E068,H3K9ac_Pro; E069,H3K9ac_Pro; E072,H3K9ac_Pro; E073,H3K9ac_Pro; E074,H3K9ac_Pro; E075,H3K9ac_Pro; E076,H3K9ac_Pro; E077,H3K9ac_Pro; E083,H3K9ac_Pro; E086,H3K9ac_Pro; E087,H3K9ac_Pro; E088,H3K9ac_Pro; E101,H3K9ac_Pro; E102,H3K9ac_Pro; E103,H3K9ac_Pro; E107,H3K9ac_Pro; E108,H3K9ac_Pro; E110,H3K9ac_Pro; E111,H3K9ac_Pro; E114,H3K9ac_Pro; E115,H3K9ac_Pro; E116,H3K9ac_Pro; E117,H3K9ac_Pro; E118,H3K9ac_Pro; E119,H3K9ac_Pro; E120,H3K9ac_Pro; E121,H3K9ac_Pro; E122,H3K9ac_Pro; E123,H3K9ac_Pro; E124,H3K9ac_Pro; E125,H3K9ac_Pro; E126,H3K9ac_Pro; E127,H3K9ac_Pro; E128,H3K9ac_Pro; E003,H3K27ac_Enh; E004,H3K27ac_Enh; E005,H3K27ac_Enh; E006,H3K27ac_Enh; E008,H3K27ac_Enh; E012,H3K27ac_Enh; E013,H3K27ac_Enh; E014,H3K27ac_Enh; E015,H3K27ac_Enh; E016,H3K27ac_Enh; E017,H3K27ac_Enh; E020,H3K27ac_Enh; E022,H3K27ac_Enh; E026,H3K27ac_Enh; E029,H3K27ac_Enh; E032,H3K27ac_Enh; E034,H3K27ac_Enh; [illegible]	ESC, ESDR, LNG, IPSC, FAT, STRM, BRST, BLD, MUS, BRN, SKIN, VAS, LIV, GI, ADRL, KID, PANC, PLCNT, THYM, HRT, OVRY, SPLN, CRVX, BONE	BRN, HRT	E003;E004;E005;E006;E007;E008;E017;E021;E022;E028;E029;E032;E033;E034;E046;E050;E051;E055;E056;E057;E059;E080;E081;E082;E083;E084;E085;E086;E088;E089;E090;E091;E092;E093;E094;E097;E098;E100;E109;E114;E116;E117;E118;E119;E120;E121;E122;E123;E124;E125;E126;E127;E128	ELF1, NFE2, NRF1, SP1, TBP, USF1, AP2GAMMA, BAF155, BAF170, USF2, RFX5, SREBP1, CCNT2, HMGN3, IRF1, NRSF, POL2B, PU1, POL2, STAT3, MAX, KAP1	.

rs73069540	7	26,904,770	RegulatoryFeature	REGULATORY	0.44	120.88	10.40	76.16			11	11	62.3526	38	21121		E001,1_TssA; E002,1_TssA; E003,1_TssA; E004,1_TssA; E005,1_TssA; E007,1_TssA; E008,1_TssA; E009,1_TssA; E010,1_TssA; E011,1_TssA; E012,10_TssBiv; E013,1_TssA; E014,1_TssA; E015,1_TssA; E016,1_TssA; E017,1_TssA; E018,1_TssA; E019,1_TssA; E020,1_TssA; E021,1_TssA; E022,1_TssA; E023,1_TssA; E024,1_TssA; E025,1_TssA; E026,1_TssA; E027,1_TssA; E028,1_TssA; E029,1_TssA; E030,2_TssAFlnk; E031,2_TssAFlnk; E032,1_TssA; E033,2_TssAFlnk; E034,11_BivFlnk; E035,1_TssA; E036,2_TssAFlnk; E037,1_TssA; E038,1_TssA; E039,2_TssAFlnk; E040,2_TssAFlnk; E041,2_TssAFlnk; E042,3_TxFlnk; E043,11_BivFlnk; E044,2_TssAFlnk; E045,2_TssAFlnk; E046,1_TssA; E047,2_TssAFlnk; E048,1_TssA; E049,1_TssA; E050,2_TssAFlnk; E051,2_TssAFlnk; E052,1_TssA; E053,11_BivFlnk; E054,10_TssBiv; E055,1_TssA; E056,1_TssA; E057,1_TssA; E058,1_TssA; E059,1_TssA; E061,2_TssAFlnk; E062,2_TssAFlnk; E063,1_TssA; E065,1_TssA; E066,1_TssA; E067,1_TssA; E068,10_TssBiv; E069,1_TssA; E070,10_TssBiv; E071,1_TssA; E072,1_TssA; E073,1_TssA; E074,1_TssA; E075,1_TssA; E076,1_TssA; E077,1_TssA; E078,1_TssA; E079,1_TssA; E080,1_TssA; E081,11_BivFlnk; E082,10_TssBiv; E083,11_BivFlnk; E084,1_TssA;	E001,1_TssA; E002,1_TssA; E003,1_TssA; E004,1_TssA; E005,1_TssA; E006,2_PromU; E007,1_TssA; E008,1_TssA; E009,1_TssA; E010,1_TssA; E011,1_TssA; E012,1_TssA; E013,2_PromU; E014,1_TssA; E015,1_TssA; E016,1_TssA; E017,1_TssA; E018,1_TssA; E019,1_TssA; E020,1_TssA; E021,1_TssA; E022,1_TssA; E023,1_TssA; E024,1_TssA; E025,1_TssA; E026,1_TssA; E027,1_TssA; E028,1_TssA; E029,2_PromU; E030,2_PromU; E031,2_PromU; E032,2_PromU; E033,2_PromU; E034,2_PromU; E035,2_PromU; E036,2_PromU; E037,2_PromU; E038,2_PromU; E039,2_PromU; E040,2_PromU; E041,2_PromU; E042,3_PromD1; E043,2_PromU; E044,2_PromU; E045,2_PromU; E046,2_PromU; E047,2_PromU; E048,2_PromU; E049,1_TssA; E050,2_PromU; E051,2_PromU; E052,1_TssA; E053,2_PromU; E054,1_TssA; E055,1_TssA; E056,1_TssA; E057,1_TssA; E058,1_TssA; E059,2_PromU; E061,1_TssA; E062,2_PromU; E063,2_PromU; E065,1_TssA; E066,2_PromU; E067,1_TssA; E068,1_TssA; E069,1_TssA; E070,1_TssA; E071,2_PromU; E072,1_TssA; E073,2_PromU; E074,1_TssA; E075,2_PromU; E076,2_PromU; E077,1_TssA; E078,3_PromD1; E079,2_PromU; E080,2_PromU; E081,1_TssA; E082,2_PromU; E083,1_TssA; E084,1_TssA; E085,1_TssA; E086,1_TssA; E087,1_TssA; E088,2_PromU; E089,2_PromU; E090,1_TssA; E091,2_PromU; E092,2_PromU; E093,2_PromU; E094,2_PromU; E095,2_PromU; E096,2_PromU; E097,1_TssA; E098,1_TssA; E099,1_TssA; E100,2_PromU; E101,3_PromD1; E102,2_PromU; E103,2_PromU; E104,2_PromU; E105,1_TssA; E106,2_PromU; E107,2_PromU; E108,2_PromU; E109,2_PromU; E110,2_PromU; E111,2_PromU; E112,2_PromU; E113,2_PromU; E114,2_PromU; E115,2_PromU; E116,2_PromU; E117,1_TssA; E118,2_PromU; E119,2_PromU; E120,1_TssA; E121,1_TssA; E122,1_TssA; E123,2_PromU; E124,2_PromU; E125,1_TssA; E126,1_TssA; E127,2_PromU; E128,2_PromU; E129,1_TssA	E001,H3K4me3_Pro; E002,H3K4me3_Pro; E003,H3K4me3_Pro; E004,H3K4me3_Pro; E005,H3K4me3_Pro; E006,H3K4me3_Pro; E007,H3K4me3_Pro; E008,H3K4me3_Pro; E009,H3K4me3_Pro; E010,H3K4me3_Pro; E011,H3K4me3_Pro; E012,H3K4me3_Pro; E013,H3K4me3_Pro; E014,H3K4me3_Pro; E015,H3K4me3_Pro; E016,H3K4me3_Pro; E017,H3K4me3_Pro; E018,H3K4me3_Pro; E019,H3K4me3_Pro; E020,H3K4me3_Pro; E021,H3K4me3_Pro; E022,H3K4me3_Pro; E023,H3K4me3_Pro; E024,H3K4me3_Pro; E025,H3K4me3_Pro; E026,H3K4me3_Pro; E027,H3K4me3_Pro; E028,H3K4me3_Pro; E029,H3K4me3_Pro; E030,H3K4me3_Pro; E031,H3K4me3_Pro; E032,H3K4me3_Pro; E033,H3K4me3_Pro; E034,H3K4me3_Pro; E035,H3K4me3_Pro; E036,H3K4me3_Pro; E037,H3K4me3_Pro; E038,H3K4me3_Pro; E039,H3K4me3_Pro; E040,H3K4me3_Pro; E041,H3K4me3_Pro; E042,H3K4me3_Pro; E043,H3K4me3_Pro; E044,H3K4me3_Pro; E045,H3K4me3_Pro; E046,H3K4me3_Pro; E047,H3K4me3_Pro; E048,H3K4me3_Pro; E049,H3K4me3_Pro; E050,H3K4me3_Pro; E051,H3K4me3_Pro; E052,H3K4me3_Pro; E053,H3K4me3_Pro; E054,H3K4me3_Pro; E055,H3K4me3_Pro; E056,H3K4me3_Pro; E057,H3K4me3_Pro; E058,H3K4me3_Pro; E059,H3K4me3_Pro; E061,H3K4me3_Pro; E062,H3K4me3_Pro; E063,H3K4me3_Pro; E065,H3K4me3_Pro; E066,H3K4me3_Pro; E067,H3K4me3_Pro; E068,H3K4me3_Pro; E069,H3K4me3_Pro; E070,H3K4me3_Pro; E071,H3K4me3_Pro; E072,H3K4me3_Pro; E073,H3K4me3_Pro; E074,H3K4me3_Pro; E075,H3K4me3_Pro; E076,H3K4me3_Pro; E077,H3K4me3_Pro; E078,H3K4me3_Pro; E079,H3K4me3_Pro; E080,H3K4me3_Pro; E081,H3K4me3_Pro; E082,H3K4me3_Pro; E083,H3K4me3_Pro; E084,H3K4me3_Pro; E085,H3K4me3_Pro; E086,H3K4me3_Pro; E087,H3K4me3_Pro; E088,H3K4me3_Pro; E089,H3K4me3_Pro; E090,H3K4me3_Pro; E091,H3K4me3_Pro; E092,H3K4me3_Pro; E093,H3K4me3_Pro; E094,H3K4me3_Pro; E095,H3K4me3_Pro; E096,H3K4me3_Pro; E097,H3K4me3_Pro; E098,H3K4me3_Pro; E099,H3K4me3_Pro; E100,H3K4me3_Pro; E101,H3K4me3_Pro; E102,H3K4me3_Pro; E103,H3K4me3_Pro; E104,H3K4me3_Pro; E105,H3K4me3_Pro; E106,H3K4me3_Pro; E107,H3K4me3_Pro; E108,H3K4me3_Pro; E109,H3K4me3_Pro; E110,H3K4me3_Pro; E111,H3K4me3_Pro; E112,H3K4me3_Pro; E113,H3K4me3_Pro; E114,H3K4me3_Pro; E115,H3K4me3_Pro; E116,H3K4me3_Pro; E117,H3K4me3_Pro; E118,H3K4me3_Pro; E119,H3K4me3_Pro; E120,H3K4me3_Pro; E121,H3K4me3_Pro; E122,H3K4me3_Pro; E123,H3K4me3_Pro; E124,H3K4me3_Pro; E125,H3K4me3_Pro; E126,H3K4me3_Pro; E127,H3K4me3_Pro; E128,H3K4me3_Pro; E129,H3K4me3_Pro; E001,H3K9ac_Pro; E003,H3K9ac_Pro; E004,H3K9ac_Pro; E006,H3K9ac_Pro; E007,H3K9ac_Pro; E008,H3K9ac_Pro; E011,H3K9ac_Pro; E014,H3K9ac_Pro; E015,H3K9ac_Pro; E016,H3K9ac_Pro; E017,H3K9ac_Pro; E018,H3K9ac_Pro; E019,H3K9ac_Pro; E020,H3K9ac_Pro; E023,H3K9ac_Pro; E025,H3K9ac_Pro; E026,H3K9ac_Pro; E027,H3K9ac_Pro; E038,H3K9ac_Pro; E049,H3K9ac_Pro; E052,H3K9ac_Pro; E062,H3K9ac_Pro; E063,H3K9ac_Pro; E066,H3K9ac_Pro; E067,H3K9ac_Pro; E068,H3K9ac_Pro; E072,H3K9ac_Pro; E073,H3K9ac_Pro; E074,H3K9ac_Pro; E075,H3K9ac_Pro; E076,H3K9ac_Pro; E077,H3K9ac_Pro; E083,H3K9ac_Pro; E086,H3K9ac_Pro; E087,H3K9ac_Pro; E088,H3K9ac_Pro; E101,H3K9ac_Pro; E102,H3K9ac_Pro; E103,H3K9ac_Pro; E107,H3K9ac_Pro; E108,H3K9ac_Pro; E110,H3K9ac_Pro; E111,H3K9ac_Pro; E114,H3K9ac_Pro; E115,H3K9ac_Pro; E116,H3K9ac_Pro; E117,H3K9ac_Pro; E118,H3K9ac_Pro; E119,H3K9ac_Pro; E120,H3K9ac_Pro; E121,H3K9ac_Pro; E122,H3K9ac_Pro; E123,H3K9ac_Pro; E124,H3K9ac_Pro; E125,H3K9ac_Pro; E126,H3K9ac_Pro; E127,H3K9ac_Pro; E128,H3K9ac_Pro; E003,H3K27ac_Enh; E004,H3K27ac_Enh; E005,H3K27ac_Enh; E006,H3K27ac_Enh; E008,H3K27ac_Enh; E011,H3K27ac_Enh; E012,H3K27ac_Enh; E013,H3K27ac_Enh; E014,H3K27ac_Enh; E015,H3K27ac_Enh; E016,H3K27ac_Enh; E017,H3K27ac_Enh; E019,H3K27ac_Enh; E020,H3K27ac_Enh; E022,H3K27ac_Enh; E026,H3K27ac_Enh; E029,H3K27ac_Enh; E032,H3K27ac_Enh; E034,H3K27ac_Enh; [illegible]	ESC, ESDR, LNG, IPSC, FAT, STRM, BRST, BLD, MUS, BRN, SKIN, VAS, LIV, GI, ADRL, KID, PANC, PLCNT, THYM, HRT, OVRY, SPLN, CRVX, BONE	.	E003;E004;E006;E007;E008;E017;E021;E022;E028;E029;E032;E033;E034;E046;E050;E051;E055;E056;E057;E059;E080;E081;E082;E083;E084;E085;E086;E088;E089;E090;E091;E092;E093;E097;E098;E100;E109;E114;E116;E117;E118;E119;E120;E121;E122;E123;E124;E125;E126;E127;E128	POL2B, E2F6, THAP1, HAE2F1, POL2	Pax-4
rs73069541	7	26,904,928	RegulatoryFeature	REGULATORY		82.12	13.24	40.24			3	3	62.3526	196	21279		E001,1_TssA; E002,1_TssA; E003,1_TssA; E008,1_TssA; E009,1_TssA; E010,1_TssA; E011,1_TssA; E012,10_TssBiv; E014,1_TssA; E015,1_TssA; E016,1_TssA; E017,1_TssA; E018,2_TssAFlnk; E019,2_TssAFlnk; E020,1_TssA; E023,1_TssA; E025,2_TssAFlnk; E026,2_TssAFlnk; E027,1_TssA; E028,1_TssA; E029,2_TssAFlnk; E030,2_TssAFlnk; E031,2_TssAFlnk; E032,2_TssAFlnk; E033,2_TssAFlnk; E034,2_TssAFlnk; E035,2_TssAFlnk; E036,2_TssAFlnk; E037,2_TssAFlnk; E038,1_TssA; E039,2_TssAFlnk; E040,2_TssAFlnk; E041,2_TssAFlnk; E042,2_TssAFlnk; E043,11_BivFlnk; E044,2_TssAFlnk; E045,2_TssAFlnk; E046,1_TssA; E047,2_TssAFlnk; E048,2_TssAFlnk; E049,2_TssAFlnk; E050,2_TssAFlnk; E051,2_TssAFlnk; E052,1_TssA; E053,11_BivFlnk; E054,10_TssBiv; E055,1_TssA; E056,1_TssA; E057,1_TssA; E058,2_TssAFlnk; E059,1_TssA; E061,2_TssAFlnk; E062,1_TssA; E063,2_TssAFlnk; E065,1_TssA; E066,1_TssA; E067,1_TssA; E068,10_TssBiv; E069,1_TssA; E070,10_TssBiv; E071,1_TssA; E072,1_TssA; E073,1_TssA; E074,1_TssA; E075,1_TssA; E076,1_TssA; E077,2_TssAFlnk; E078,1_TssA; E080,2_TssAFlnk; E081,11_BivFlnk; E082,10_TssBiv; E083,10_TssBiv; [illegible]	E001,2_PromU; E002,2_PromU; E003,2_PromU; E004,2_PromU; E005,1_TssA; E006,2_PromU; E007,22_PromP; E008,2_PromU; E009,2_PromU; E010,2_PromU; E011,2_PromU; E012,2_PromU; E013,2_PromU; E014,2_PromU; E015,2_PromU; E016,2_PromU; E017,2_PromU; E018,2_PromU; E019,2_PromU; E020,2_PromU; E021,1_TssA; E022,1_TssA; E023,2_PromU; E024,1_TssA; E025,2_PromU; E026,2_PromU; E027,2_PromU; E028,2_PromU; E029,2_PromU; E030,2_PromU; E031,2_PromU; E032,2_PromU; E033,2_PromU; E034,2_PromU; E035,2_PromU; E036,2_PromU; E037,2_PromU; E038,2_PromU; E039,2_PromU; E040,2_PromU; E041,2_PromU; E042,2_PromU; E043,2_PromU; E044,2_PromU; E045,2_PromU; E046,2_PromU; E047,2_PromU; E048,2_PromU; E049,2_PromU; E050,2_PromU; E051,2_PromU; E052,2_PromU; E053,2_PromU; E054,2_PromU; E055,2_PromU; E056,2_PromU; E057,2_PromU; E058,2_PromU; E059,2_PromU; E061,2_PromU; E062,2_PromU; E063,2_PromU; E065,1_TssA; E066,2_PromU; E067,2_PromU; E068,2_PromU; E069,2_PromU; E070,2_PromU; E071,2_PromU; E072,2_PromU; E073,2_PromU; E074,2_PromU; E075,2_PromU; E076,2_PromU; E077,2_PromU; E078,2_PromU; E079,2_PromU; E080,2_PromU; E081,2_PromU; E082,2_PromU; E083,2_PromU; E084,2_PromU; E085,2_PromU; E086,2_PromU; E087,2_PromU; E088,2_PromU; E089,2_PromU; E090,2_PromU; E091,2_PromU; E092,2_PromU; E093,2_PromU; E094,1_TssA; E095,2_PromU; E096,2_PromU; E097,2_PromU; E098,1_TssA; E099,2_PromU; E100,2_PromU; E101,2_PromU; E102,2_PromU; E103,2_PromU; E104,1_TssA; E105,1_TssA; E106,2_PromU; E107,2_PromU; E108,2_PromU; E109,2_PromU; E110,2_PromU; E111,2_PromU; E112,2_PromU; E113,2_PromU; E114,2_PromU; E115,2_PromU; E116,2_PromU; E117,2_PromU; E118,2_PromU; E119,2_PromU; E120,2_PromU; E121,2_PromU; E122,2_PromU; E123,2_PromU; E124,2_PromU; E125,2_PromU; E126,2_PromU; E127,2_PromU; E128,2_PromU; E129,2_PromU	E001,H3K4me3_Pro; E002,H3K4me3_Pro; E003,H3K4me3_Pro; E004,H3K4me3_Pro; E005,H3K4me3_Pro; E008,H3K4me3_Pro; E009,H3K4me3_Pro; E010,H3K4me3_Pro; E011,H3K4me3_Pro; E012,H3K4me3_Pro; E013,H3K4me3_Pro; E014,H3K4me3_Pro; E015,H3K4me3_Pro; E016,H3K4me3_Pro; E017,H3K4me3_Pro; E018,H3K4me3_Pro; E019,H3K4me3_Pro; E020,H3K4me3_Pro; E021,H3K4me3_Pro; E022,H3K4me3_Pro; E023,H3K4me3_Pro; E025,H3K4me3_Pro; E026,H3K4me3_Pro; E027,H3K4me3_Pro; E028,H3K4me3_Pro; E029,H3K4me3_Pro; E030,H3K4me3_Pro; E031,H3K4me3_Pro; E032,H3K4me3_Pro; E033,H3K4me3_Pro; E034,H3K4me3_Pro; E035,H3K4me3_Pro; E036,H3K4me3_Pro; E037,H3K4me3_Pro; E038,H3K4me3_Pro; E039,H3K4me3_Pro; E040,H3K4me3_Pro; E041,H3K4me3_Pro; E042,H3K4me3_Pro; E043,H3K4me3_Pro; E044,H3K4me3_Pro; E045,H3K4me3_Pro; E046,H3K4me3_Pro; E047,H3K4me3_Pro; E048,H3K4me3_Pro; E049,H3K4me3_Pro; E050,H3K4me3_Pro; E051,H3K4me3_Pro; E052,H3K4me3_Pro; E053,H3K4me3_Pro; E054,H3K4me3_Pro; E055,H3K4me3_Pro; E056,H3K4me3_Pro; E057,H3K4me3_Pro; E058,H3K4me3_Pro; E059,H3K4me3_Pro; E061,H3K4me3_Pro; E062,H3K4me3_Pro; E063,H3K4me3_Pro; E065,H3K4me3_Pro; E066,H3K4me3_Pro; E067,H3K4me3_Pro; E068,H3K4me3_Pro; E069,H3K4me3_Pro; E070,H3K4me3_Pro; E071,H3K4me3_Pro; E072,H3K4me3_Pro; E073,H3K4me3_Pro; E074,H3K4me3_Pro; E075,H3K4me3_Pro; E076,H3K4me3_Pro; E077,H3K4me3_Pro; E078,H3K4me3_Pro; E079,H3K4me3_Pro; E080,H3K4me3_Pro; E081,H3K4me3_Pro; E082,H3K4me3_Pro; E083,H3K4me3_Pro; E084,H3K4me3_Pro; E085,H3K4me3_Pro; E086,H3K4me3_Pro; E087,H3K4me3_Pro; E088,H3K4me3_Pro; E089,H3K4me3_Pro; E090,H3K4me3_Pro; E091,H3K4me3_Pro; E092,H3K4me3_Pro; E093,H3K4me3_Pro; E094,H3K4me3_Pro; E095,H3K4me3_Pro; E096,H3K4me3_Pro; E097,H3K4me3_Pro; E099,H3K4me3_Pro; E100,H3K4me3_Pro; E101,H3K4me3_Pro; E102,H3K4me3_Pro; E103,H3K4me3_Pro; E104,H3K4me3_Pro; E105,H3K4me3_Pro; E106,H3K4me3_Pro; E107,H3K4me3_Pro; E108,H3K4me3_Pro; E109,H3K4me3_Pro; E110,H3K4me3_Pro; E111,H3K4me3_Pro; E112,H3K4me3_Pro; E113,H3K4me3_Pro; E114,H3K4me3_Pro; E115,H3K4me3_Pro; E116,H3K4me3_Pro; E117,H3K4me3_Pro; E118,H3K4me3_Pro; E119,H3K4me3_Pro; E120,H3K4me3_Pro; E121,H3K4me3_Pro; E122,H3K4me3_Pro; E123,H3K4me3_Pro; E124,H3K4me3_Pro; E125,H3K4me3_Pro; E126,H3K4me3_Pro; E127,H3K4me3_Pro; E128,H3K4me3_Pro; E129,H3K4me3_Pro; E001,H3K9ac_Pro; E003,H3K9ac_Pro; E004,H3K9ac_Pro; E006,H3K9ac_Pro; E008,H3K9ac_Pro; E011,H3K9ac_Pro; E014,H3K9ac_Pro; E017,H3K9ac_Pro; E018,H3K9ac_Pro; E019,H3K9ac_Pro; E020,H3K9ac_Pro; E023,H3K9ac_Pro; E025,H3K9ac_Pro; E026,H3K9ac_Pro; E027,H3K9ac_Pro; E038,H3K9ac_Pro; E049,H3K9ac_Pro; E052,H3K9ac_Pro; E063,H3K9ac_Pro; E066,H3K9ac_Pro; E068,H3K9ac_Pro; E072,H3K9ac_Pro; E073,H3K9ac_Pro; E074,H3K9ac_Pro; E075,H3K9ac_Pro; E076,H3K9ac_Pro; E077,H3K9ac_Pro; E083,H3K9ac_Pro; E086,H3K9ac_Pro; E087,H3K9ac_Pro; E088,H3K9ac_Pro; E101,H3K9ac_Pro; E102,H3K9ac_Pro; E103,H3K9ac_Pro; E107,H3K9ac_Pro; E108,H3K9ac_Pro; E110,H3K9ac_Pro; E111,H3K9ac_Pro; E114,H3K9ac_Pro; E115,H3K9ac_Pro; E116,H3K9ac_Pro; E117,H3K9ac_Pro; E118,H3K9ac_Pro; E119,H3K9ac_Pro; E120,H3K9ac_Pro; E121,H3K9ac_Pro; E122,H3K9ac_Pro; E123,H3K9ac_Pro; E124,H3K9ac_Pro; E125,H3K9ac_Pro; E126,H3K9ac_Pro; E127,H3K9ac_Pro; E128,H3K9ac_Pro; E003,H3K27ac_Enh; E005,H3K27ac_Enh; E008,H3K27ac_Enh; E012,H3K27ac_Enh; E013,H3K27ac_Enh; E014,H3K27ac_Enh; E015,H3K27ac_Enh; E017,H3K27ac_Enh; E019,H3K27ac_Enh; E020,H3K27ac_Enh; E022,H3K27ac_Enh; E026,H3K27ac_Enh; E029,H3K27ac_Enh; E032,H3K27ac_Enh; E034,H3K27ac_Enh; E037,H3K27ac_Enh; E038,H3K27ac_Enh; E040,H3K27ac_Enh; E041,H3K27ac_Enh; E042,H3K27ac_Enh; E043,H3K27ac_Enh; E044,H3K27ac_Enh; E046,H3K27ac_Enh; E047,H3K27ac_Enh; E048,H3K27ac_Enh; E049,H3K27ac_Enh; E050,H3K27ac_Enh; E055,H3K27ac_Enh; [illegible]	ESC, ESDR, LNG, IPSC, FAT, STRM, BRST, BLD, MUS, BRN, SKIN, VAS, LIV, GI, ADRL, HRT, KID, PANC, PLCNT, THYM, OVRY, CRVX, BONE	.	E006;E008;E028;E029;E032;E033;E046;E051;E084;E086;E088;E089;E090;E092;E123	.	Pbx-1
rs12540388	7	26,905,731	Intergenic	UPSTREAM		5.00	4.92	5.00						999	22083		E057,7_Enh	E010,19_DNase; E014,19_DNase; E028,19_DNase; E030,19_DNase; E031,19_DNase; E032,19_DNase; E033,19_DNase; E034,19_DNase; E035,19_DNase; E036,19_DNase; E038,19_DNase; E039,19_DNase; E040,19_DNase; E041,19_DNase; E042,19_DNase; E043,19_DNase; E044,19_DNase; E045,19_DNase; E047,19_DNase; E048,19_DNase; E049,19_DNase; E050,19_DNase; E051,19_DNase; E058,19_DNase; E065,19_DNase; E066,19_DNase; E069,22_PromP; E076,19_DNase; E077,19_DNase; E086,19_DNase; E102,19_DNase; E110,19_DNase; E112,19_DNase; E116,19_DNase; E117,19_DNase; E118,19_DNase; E119,19_DNase; E124,19_DNase; E127,19_DNase	E001,H3K9ac_Pro; E008,H3K9ac_Pro; E020,H3K9ac_Pro; E023,H3K9ac_Pro; E038,H3K9ac_Pro; E063,H3K9ac_Pro; E066,H3K9ac_Pro; E068,H3K9ac_Pro; E072,H3K9ac_Pro; E075,H3K9ac_Pro; E077,H3K9ac_Pro; E101,H3K9ac_Pro; E102,H3K9ac_Pro; E108,H3K9ac_Pro; E117,H3K9ac_Pro; E127,H3K9ac_Pro; E003,H3K27ac_Enh; E034,H3K27ac_Enh; E043,H3K27ac_Enh; E047,H3K27ac_Enh; E049,H3K27ac_Enh; E058,H3K27ac_Enh; E075,H3K27ac_Enh; E076,H3K27ac_Enh; E106,H3K27ac_Enh; E119,H3K27ac_Enh; E129,H3K27ac_Enh; E005,H3K4me3_Pro; E014,H3K4me3_Pro; E019,H3K4me3_Pro; E028,H3K4me3_Pro; E030,H3K4me3_Pro; E032,H3K4me3_Pro; E038,H3K4me3_Pro; E040,H3K4me3_Pro; E045,H3K4me3_Pro; E047,H3K4me3_Pro; E068,H3K4me3_Pro; E072,H3K4me3_Pro; E073,H3K4me3_Pro; E074,H3K4me3_Pro; E076,H3K4me3_Pro; E077,H3K4me3_Pro; E079,H3K4me3_Pro; E088,H3K4me3_Pro; E095,H3K4me3_Pro; E097,H3K4me3_Pro; E102,H3K4me3_Pro; E106,H3K4me3_Pro; E110,H3K4me3_Pro; E111,H3K4me3_Pro; E113,H3K4me3_Pro; E117,H3K4me3_Pro; E018,H3K4me1_Enh; E028,H3K4me1_Enh; E032,H3K4me1_Enh; E033,H3K4me1_Enh; E039,H3K4me1_Enh; E041,H3K4me1_Enh; E048,H3K4me1_Enh; E057,H3K4me1_Enh; E058,H3K4me1_Enh; E073,H3K4me1_Enh; E081,H3K4me1_Enh; E088,H3K4me1_Enh; E099,H3K4me1_Enh; E117,H3K4me1_Enh; E119,H3K4me1_Enh; E125,H3K4me1_Enh	.	SKIN	E032;E088	.	DMRT4
rs2391362	7	26,907,123	Intergenic	UPSTREAM		1.00	0.84	1.00						127736	128664		.	.	E007,H3K9ac_Pro; E038,H3K9ac_Pro; E068,H3K9ac_Pro	.	.	.	.	GCNF, LBP-1
rs76438067	7	26,911,904	Transcript	INTRONIC		3.00	1.00	1.92						122955	133445		.	.	.	.	.	.	.	Mrg, NRSF, Tgif1, VDR

#abbreviation description

Epigenome IDs

Epigenome ID (EID)	Group	Mnemonic	Description
E017	IMR90	LNG.IMR90	IMR90 fetal lung fibroblasts Cell Line
E002	ESC	ESC.WA7	ES-WA7 Cells
E008	ESC	ESC.H9	H9 Cells
E001	ESC	ESC.I3	ES-I3 Cells
E015	ESC	ESC.HUES6	HUES6 Cells
E014	ESC	ESC.HUES48	HUES48 Cells
E016	ESC	ESC.HUES64	HUES64 Cells
E003	ESC	ESC.H1	H1 Cells
E024	ESC	ESC.4STAR	ES-UCSF4 Cells
E020	iPSC	IPSC.20B	iPS-20b Cells
E019	iPSC	IPSC.18	iPS-18 Cells
E018	iPSC	IPSC.15b	iPS-15b Cells
E021	iPSC	IPSC.DF.6.9	iPS DF 6.9 Cells
E022	iPSC	IPSC.DF.19.11	iPS DF 19.11 Cells
E007	ES-deriv	ESDR.H1.NEUR.PROG	H1 Derived Neuronal Progenitor Cultured Cells
E009	ES-deriv	ESDR.H9.NEUR.PROG	H9 Derived Neuronal Progenitor Cultured Cells
E010	ES-deriv	ESDR.H9.NEUR	H9 Derived Neuron Cultured Cells
E013	ES-deriv	ESDR.CD56.MESO	hESC Derived CD56+ Mesoderm Cultured Cells
E012	ES-deriv	ESDR.CD56.ECTO	hESC Derived CD56+ Ectoderm Cultured Cells
E011	ES-deriv	ESDR.CD184.ENDO	hESC Derived CD184+ Endoderm Cultured Cells
E004	ES-deriv	ESDR.H1.BMP4.MESO	H1 BMP4 Derived Mesendoderm Cultured Cells
E005	ES-deriv	ESDR.H1.BMP4.TROP	H1 BMP4 Derived Trophoblast Cultured Cells
E006	ES-deriv	ESDR.H1.MSC	H1 Derived Mesenchymal Stem Cells
E062	Blood & T-cell	BLD.PER.MONUC.PC	Primary mononuclear cells from peripheral blood
E034	Blood & T-cell	BLD.CD3.PPC	Primary T cells from peripheral blood
E045	Blood & T-cell	BLD.CD4.CD25I.CD127.TMEMPC	Primary T cells effector/memory enriched from peripheral blood
E033	Blood & T-cell	BLD.CD3.CPC	Primary T cells from cord blood
E044	Blood & T-cell	BLD.CD4.CD25.CD127M.TREGPC	Primary T regulatory cells from peripheral blood
E043	Blood & T-cell	BLD.CD4.CD25M.TPC	Primary T helper cells from peripheral blood
E039	Blood & T-cell	BLD.CD4.CD25M.CD45RA.NPC	Primary T helper naive cells from peripheral blood
E041	Blood & T-cell	BLD.CD4.CD25M.IL17M.PL.TPC	Primary T helper cells PMA-I stimulated
E042	Blood & T-cell	BLD.CD4.CD25M.IL17P.PL.TPC	Primary T helper 17 cells PMA-I stimulated
E040	Blood & T-cell	BLD.CD4.CD25M.CD45RO.MPC	Primary T helper memory cells from peripheral blood 1
E037	Blood & T-cell	BLD.CD4.MPC	Primary T helper memory cells from peripheral blood 2
E048	Blood & T-cell	BLD.CD8.MPC	Primary T CD8+ memory cells from peripheral blood
E038	Blood & T-cell	BLD.CD4.NPC	Primary T helper naive cells from peripheral blood
E047	Blood & T-cell	BLD.CD8.NPC	Primary T CD8+ naive cells from peripheral blood
E029	HSC & B-cell	BLD.CD14.PC	Primary monocytes from peripheral blood
E031	HSC & B-cell	BLD.CD19.CPC	Primary B cells from cord blood
E035	HSC & B-cell	BLD.CD34.PC	Primary hematopoietic stem cells
E051	HSC & B-cell	BLD.MOB.CD34.PC.M	Primary hematopoietic stem cells G-CSF-mobilized Male
E050	HSC & B-cell	BLD.MOB.CD34.PC.F	Primary hematopoietic stem cells G-CSF-mobilized Female
E036	HSC & B-cell	BLD.CD34.CC	Primary hematopoietic stem cells short term culture
E032	HSC & B-cell	BLD.CD19.PPC	Primary B cells from peripheral blood
E046	HSC & B-cell	BLD.CD56.PC	Primary Natural Killer cells from peripheral blood
E030	HSC & B-cell	BLD.CD15.PC	Primary neutrophils from peripheral blood
E026	Mesench	STRM.MRW.MSC	Bone Marrow Derived Cultured Mesenchymal Stem Cells
E049	Mesench	STRM.CHON.MRW.DR.MSC	Mesenchymal Stem Cell Derived Chondrocyte Cultured Cells
E025	Mesench	FAT.ADIP.DR.MSC	Adipose Derived Mesenchymal Stem Cell Cultured Cells
E023	Mesench	FAT.MSC.DR.ADIP	Mesenchymal Stem Cell Derived Adipocyte Cultured Cells
E052	Myosat	MUS.SAT	Muscle Satellite Cultured Cells
E055	Epithelial	SKIN.PEN.FRSK.FIB.01	Foreskin Fibroblast Primary Cells skin01
E056	Epithelial	SKIN.PEN.FRSK.FIB.02	Foreskin Fibroblast Primary Cells skin02
E059	Epithelial	SKIN.PEN.FRSK.MEL.01	Foreskin Melanocyte Primary Cells skin01
E061	Epithelial	SKIN.PEN.FRSK.MEL.03	Foreskin Melanocyte Primary Cells skin03
E057	Epithelial	SKIN.PEN.FRSK.KER.02	Foreskin Keratinocyte Primary Cells skin02
E058	Epithelial	SKIN.PEN.FRSK.KER.03	Foreskin Keratinocyte Primary Cells skin03
E028	Epithelial	BRST.HMEC.35	Breast variant Human Mammary Epithelial Cells (vHMEC)
E027	Epithelial	BRST.MYO	Breast Myoepithelial Primary Cells
E054	Neurosph	BRN.GANGEM.DR.NRSPHR	Ganglion Eminence derived primary cultured neurospheres
E053	Neurosph	BRN.CRTX.DR.NRSPHR	Cortex derived primary cultured neurospheres
E112	Thymus	THYM	Thymus
E093	Thymus	THYM.FET	Fetal Thymus

E071	Brain	BRN.HIPP.MID	Brain Hippocampus Middle
E074	Brain	BRN.SUB.NIG	Brain Substantia Nigra
E068	Brain	BRN.ANT.CAUD	Brain Anterior Caudate
E069	Brain	BRN.CING.GYR	Brain Cingulate Gyrus
E072	Brain	BRN.INF.TMP	Brain Inferior Temporal Lobe
E067	Brain	BRN.ANG.GYR	Brain Angular Gyrus
E073	Brain	BRN.DL.PRFRNTL.CRTX	Brain_Dorsolateral_Prefrontal_Cortex
E070	Brain	BRN.GRM.MTRX	Brain Germinal Matrix
E082	Brain	BRN.FET.F	Fetal Brain Female
E081	Brain	BRN.FET.M	Fetal Brain Male
E063	Adipose	FAT.ADIP.NUC	Adipose Nuclei
E100	Muscle	MUS.PSOAS	Psoas Muscle
E108	Muscle	MUS.SKLT.F	Skeletal Muscle Female
E107	Muscle	MUS.SKLT.M	Skeletal Muscle Male
E089	Muscle	MUS.TRNK.FET	Fetal Muscle Trunk
E090	Muscle	MUS.LEG.FET	Fetal Muscle Leg
E083	Heart	HRT.FET	Fetal Heart
E104	Heart	HRT.ATR.R	Right Atrium
E095	Heart	HRT.VENT.L	Left Ventricle
E105	Heart	HRT.VNT.R	Right Ventricle
E065	Heart	VAS.AOR	Aorta
E078	Sm. Muscle	GI.DUO.SM.MUS	Duodenum Smooth Muscle
E076	Sm. Muscle	GI.CLN.SM.MUS	Colon Smooth Muscle
E103	Sm. Muscle	GI.RECT.SM.MUS	Rectal Smooth Muscle
E111	Sm. Muscle	GI.STMC.MUS	Stomach Smooth Muscle
E092	Digestive	GI.STMC.FET	Fetal Stomach
E085	Digestive	GI.S.INT.FET	Fetal Intestine Small
E084	Digestive	GI.L.INT.FET	Fetal Intestine Large
E109	Digestive	GI.S.INT	Small Intestine
E106	Digestive	GI.CLN.SIG	Sigmoid Colon
E075	Digestive	GI.CLN.MUC	Colonic Mucosa
E101	Digestive	GI.RECT.MUC.29	Rectal Mucosa Donor 29
E102	Digestive	GI.RECT.MUC.31	Rectal Mucosa Donor 31
E110	Digestive	GI.STMC.MUC	Stomach Mucosa
E077	Digestive	GI.DUO.MUC	Duodenum Mucosa
E079	Digestive	GI.ESO	Esophagus
E094	Digestive	GI.STMC.GAST	Gastric
E099	Other	PLCNT.AMN	Placenta Amnion
E086	Other	KID.FET	Fetal Kidney
E088	Other	LNG.FET	Fetal Lung
E097	Other	OVRY	Ovary
E087	Other	PANC.ISLT	Pancreatic Islets
E080	Other	ADRL.GLND.FET	Fetal Adrenal Gland
E091	Other	PLCNT.FET	Placenta
E066	Other	LIV.ADLT	Liver
E098	Other	PANC	Pancreas
E096	Other	LNG	Lung
E113	Other	SPLN	Spleen
E114	ENCODE2012	LNG.A549.ETOH002.CNCR	A549 EtOH 0.02pct Lung Carcinoma Cell Line
E115	ENCODE2012	BLD.DND41.CNCR	Dnd41 TCell Leukemia Cell Line
E116	ENCODE2012	BLD.GM12878	GM12878 Lymphoblastoid Cells
E117	ENCODE2012	CRVX.HELAS3.CNCR	HeLa-S3 Cervical Carcinoma Cell Line
E118	ENCODE2012	LIV.HEPG2.CNCR	HepG2 Hepatocellular Carcinoma Cell Line
E119	ENCODE2012	BRST.HMEC	HMEC Mammary Epithelial Primary Cells
E120	ENCODE2012	MUS.HSMM	HSMM Skeletal Muscle Myoblasts Cells
E121	ENCODE2012	MUS.HSMMT	HSMM cell derived Skeletal Muscle Myotubes Cells
E122	ENCODE2012	VAS.HUVEC	HUVEC Umbilical Vein Endothelial Primary Cells
E123	ENCODE2012	BLD.K562.CNCR	K562 Leukemia Cells
E124	ENCODE2012	BLD.CD14.MONO	Monocytes-CD14+ RO01746 Primary Cells
E125	ENCODE2012	BRN.NHA	NH-A Astrocytes Primary Cells
E126	ENCODE2012	SKIN.NHDFAD	NHDF-Ad Adult Dermal Fibroblast Primary Cells
E127	ENCODE2012	SKIN.NHEK	NHEK-Epidermal Keratinocyte Primary Cells
E128	ENCODE2012	LNG.NHLF	NHLF Lung Fibroblast Primary Cells
E129	ENCODE2012	BONE.OSTEO	Osteoblast Primary Cells

chromatin core models

Core 15-state model **(5 marks, 127 epigenomes)**

State No	Mnemonic	Description
1	TssA	Active TSS
2	TssAFlnk	Flanking Active TSS
3	TxFlnk	Transcr. at gene 5' and 3'
4	Tx	Strong transcription
5	TxWk	Weak transcription
6	EnhG	Genic enhancers
7	Enh	Enhancers
8	ZNF/Rpts	ZNF genes & repeats
9	Het	Heterochromatin
10	TssBiv	Bivalent/Poised TSS
11	BivFlnk	Flanking Bivalent TSS/Enh
12	EnhBiv	Bivalent Enhancer
13	ReprPC	Repressed PolyComb
14	ReprPCWk	Weak Repressed PolyComb
15	Quies	Quiescent/Low

Chromatin state model based on imputed data (25 state, 12 marks, 127 epigenomes)

State No	Mnemonic	Description
1	TssA	Active TSS
2	PromU	Promoter Upstream TSS
3	PromD1	Promoter Downstream TSS 1
4	PromD2	Promoter Downstream TSS 2
5	Tx5�	Transcribed - 5' preferential
6	Tx	Strong transcription
7	Tx3�	Transcribed - 3' preferential
8	TxWk	Weak transcription
9	TxReg	Transcribed & regulatory (Prom/Enh)
10	TxEnh5�	Transcribed 5' preferential and Enh
11	TxEnh3�	Transcribed 3' preferential and Enh
12	TxEnhW	Transcribed and Weak Enhancer
13	EnhA1	Active Enhancer 1
14	EnhA2	Active Enhancer 2
15	EnhAF	Active Enhancer Flank
16	EnhW1	Weak Enhancer 1
17	EnhW2	Weak Enhancer 2
18	EnhAc	Primary H3K27ac possible Enhancer
19	DNase	Primary DNase
20	ZNF/Rpts	ZNF genes & repeats
21	Het	Heterochromatin
22	PromP	Poised Promoter
23	PromBiv	Bivalent Promoter
24	ReprPC	Repressed Polycomb
25	Quies	Quiescent/Low

SNP: SNP rs ID; **CHR:** Chromosome; **BP:** position (NCBI build 37/hg19 coordinates) of SNP marker; **AnnoType**: 'CodingTranscript', 'Intergenic', 'MotifFeature', 'NonCodingTranscript', 'RegulatoryFeature', 'Transcript'; **Consequence**: '3PRIME_UTR', '5PRIME_UTR', 'DOWNSTREAM', 'INTERGENIC', 'INTRONIC', 'NON_SYNONYMOUS', 'SYNONYMOUS', 'REGULATORY', 'STOP_GAINED', 'STOP_LOST', 'SPLICE_SITE', 'CANONCAL_SPLICE', 'UPSTREAM', 'NONCODING_CHANGE'; **EncExp**: maximum ENCODE expression value; **EncH3K27Ac**: maximum ENCODE H3K27 acetylation level; **EncH3K4Me1**: maximum ENCODE H3K4 methylation level; **EncH3K4Me3**: maximum ENCODE H3K4 trimethylation level; **tOverlapMotifs**: number of overlapping predicted transcription factor motifs; **motifDist**: Reference minus alternate allele difference in nucleotide frequency within an predicted overlapping motif; **TFBS**: number of different overlapping Chromatin immunoprecipitation (ChIP) transcription factor binding sites; **TFBSPeaks**: number of overlapping ChIP transcription factor binding site peaks summed over different cell types/tissue; **TFBSPeaksMax**: maximum value of overlapping ChIP transcription factor binding site peaks across cell types/tissue; **minDistTSS**: distance to closest transcribed sequence start (TSS); **minDistTSE**: distance to closest transcribed sequence end (TSE); **regulatory features**: regulatory features from the Ensembl Regulation database, e.g. histone modification sites, open chromatin or transcription factor binding sites); **Chromatin States**: chromatin 15-state core model learned on five core chromatin marks: shown are the 'Epigenome ID' and the predicted chromatin state (state number and mnemonic); **Chromatin States Imputed**: chromatin 25-state core model learned on 12 core chromatin marks: shown are the 'Epigenome ID' and the predicted chromatin state (state number and mnemonic); **Chromatin Marks**: shown are the 'Epigenome ID' and the chromatin marks H3K4me1 and H3K27ac as enhancers and H3K4me3 and H3K9ac as promotors; **Promoter histone marks**: cell type/tissue in which promoter chromatin mark was found; **Enhancer histone marks**: cell type/tissue in which enhancer chromatin mark was found; **DNAse**: 'Epigenome ID' in which DNAse hypersensitivity site was found; **Proteins bound by ChIP (ENCODE)**: DNA-associated proteins identified through ChIP-sequencing by the ENCODE[6] project; **Regulatory motifs altered:** shown are regulatory motifs altered by the variant.

Supplementary Table 9. Prevalence of different CVD grades in the study population (panel A-C, see Supplementary Table 1) according to the CEAP classification.

CVD grade	Total (n=2,269)	Males (n=641; 28.3%)	Females (n=1,628; 71.7%)
C2	383 (16.9%)	85 (13.3%)	298 (18.3%)
C3	1,488 (65.6%)	378 (59.0%)	1110 (68.2%)
C4	398 (17.5%)	178 (27.8%)	220 (13.5%)

Supplementary Table 10. Characteristics of medical history of CVD patients (panel A-C, Supplementary Table 1).

	All patients (n=2,269)	SD (range)	Males (n=641)	SD (range)	Females (n=1,628)	SD (range)
Age at ascertainment	55.4 years	11.1 years (21-82)	55.3 years	11.6 years (22-80)	55.5 years	11.0 years (21-82)
Age at onset	32.3 years	12.0 years (5-86)	36.2 years	13.0 years (5-86)	30.8 years	11.2 years (6-72)
Duration of disease	23.1 years	12.9 years (0-75)	19.0 years	12.0 years (0-59)	24.7 years	12.9 years (0-75)
BMI	26.7	4.8 (14-66)	27.4	3.7 (20-53)	26.5	5.1 (14-66)

Given are overall and sex-specific means, standard deviations (SD) and ranges. Duration of disease was calculated as the difference between age at onset and age at ascertainment.

Supplementary Table 11. Personal characteristics of healthy control individuals (panel A-C, Supplementary Table 1).

	Controls (n=4,374)	SD (range)	Males (n=2,167)	SD (range)	Females (n=2,207)	SD (range)
Age at ascertainment	49.4 years	15.9 years (18-90)	50.2 years	15.6 years (18-81)	48.6 years	16.2 years (18-90)
BMI*	25.9	4.5 (17-61)	26.4	4.0 (17-61)	25.4	4.9 (17-58)

Given are the overall and sex-specific means, standard deviations (SD) and ranges. Age at ascertainment was available for all controls from the CAU Kiel and PopGen (4,374 controls; panels A-C in **Supplementary Table 1**).

*BMI was available only for the controls of panel A and B from the CAU Kiel and PopGen (3,153 controls; 1,622 males and 1,531 females).

Supplementary Figures

Supplementary Figure 1. Principal component analysis of QCed CVD discovery panel (panel A, Supplementary Table 1). (a) All GWAS samples are plotted on the first two principles components, colored by cases (Var: cyan) and controls (Con: green), respectively. The outlier detection procedure, as implemented in EIGENSTRAT[7] using default parameters (sigma=6.0, maxiter=5, topk=10), revealed no population outliers. **(b)** Distribution of cases and controls along the first ten principal components stratified by phenotype. **(c)** Principal component analysis of combined genetic data from GWAS samples and 26 reference populations from the 1000 Genomes Project (http://www.internationalgenome.org/category/population/). GWAS samples (grey crosses) are anchored near the CEU population, with values smaller than the median plus/minus five times the interquartile range (median ±5×IQR; red square) of GWAS samples for the first two principles components. Super population code: AFR, African; AMR, Ad Mixed American, EAS, East Asian, EUR, European; SAS, South Asian; Population code: CHB, Han Chinese in Bejing, China; JPT, Japanese in Tokyo, Japan; CHS, Southern Han Chinese; CDX, Chinese Dai in Xishuangbanna, China; KHV, Kinh in Ho Chi Minh City, Vietnam; CEU, Utah Residents (CEPH) with Northern and Western Ancestry; TSI, Toscani in Italia; FIN, Finnish in Finland; GBR, British in England and Scotland; IBS, Iberian Population in Spain; YRI, Yoruba in Ibadan, Nigeria; LWK, Luhya in Webuye, Kenya; GWD, Gambia; MSL, Mende in Sierra Leone; ESN, Esan in Nigeria; ASW, Americans of African Ancestry in SW USA; ACB, African Caribbeans in Barbados; MXL, Mexican Ancestry from Los Angeles USA; PUR, Puerto Ricans from Puerto Rico; CLM, Colombians from Medellin, Colombia; PEL, Peruvians from Lima, Peru; GIH, Gujarati Indian from Houston, Texas; PJL, Punjabi from Lahore, Pakistan; BEB, Bengali from Bangladesh; STU, Sri Lankan Tamil from the UK; ITU, Indian Telugu from the UK.

(a)

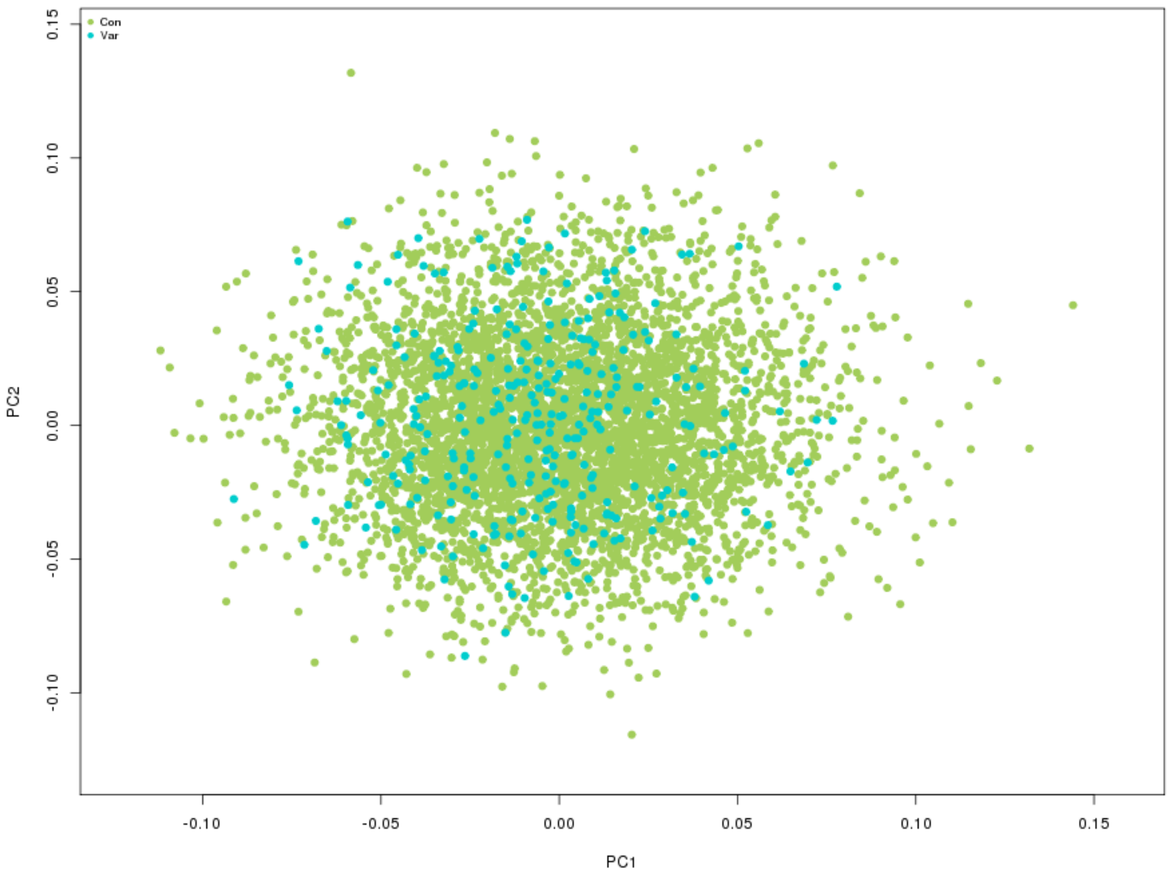

(b)

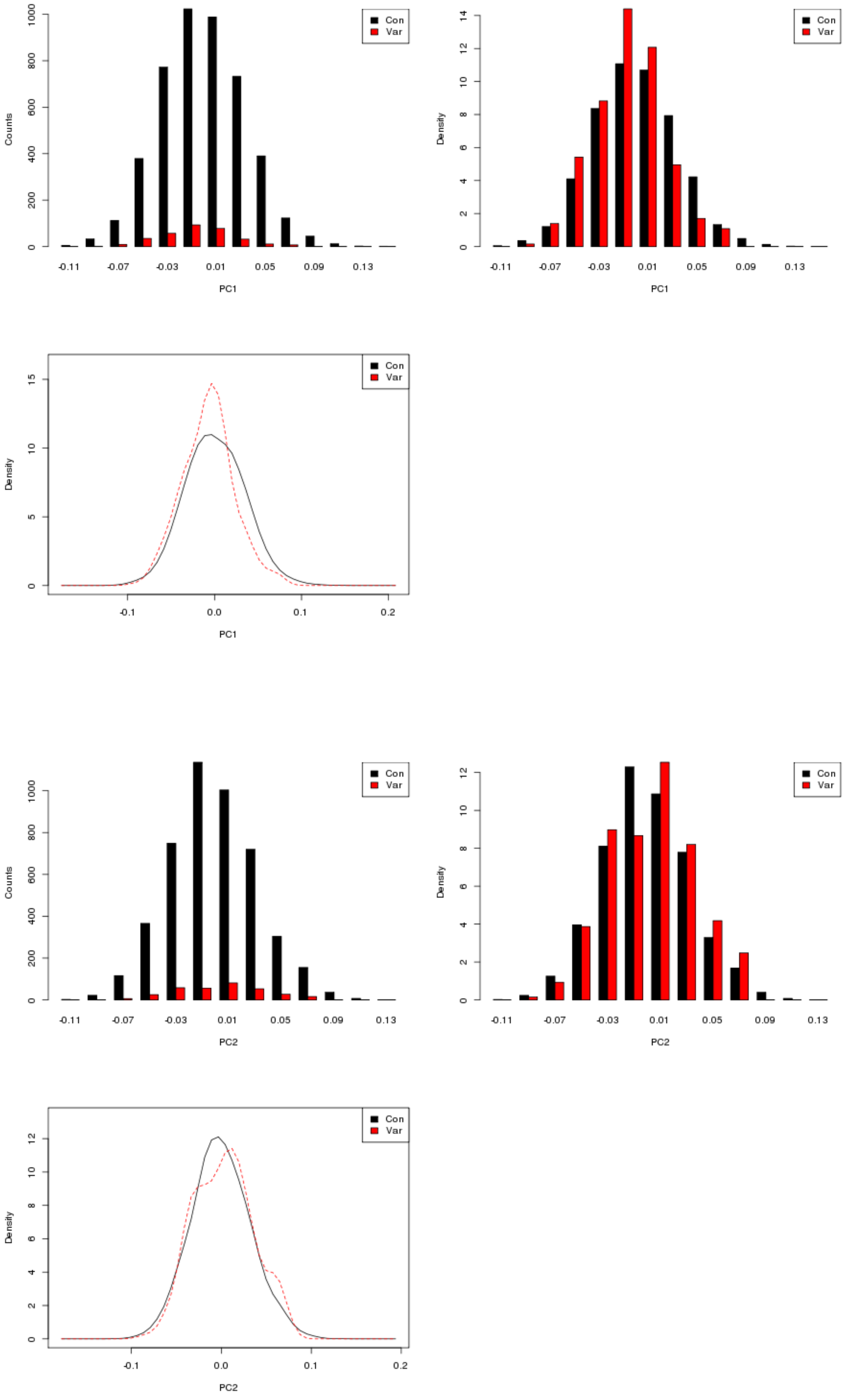

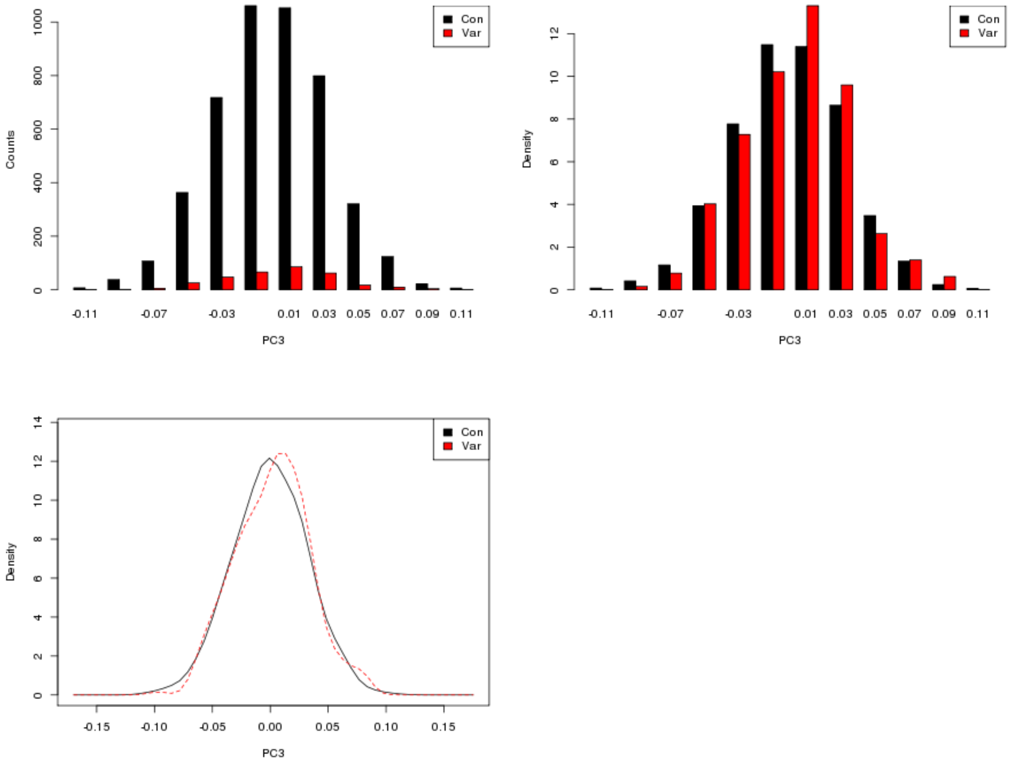
Con
Var
Counts
0
200
400
600
800
1000
-0.11
-0.07
-0.03
0.01
0.03
0.05
0.07
0.09
0.11
PC3
Density
0
2
4
6
8
10
12
14
-0.15
-0.10
-0.05
0.00
0.05
0.10
0.15

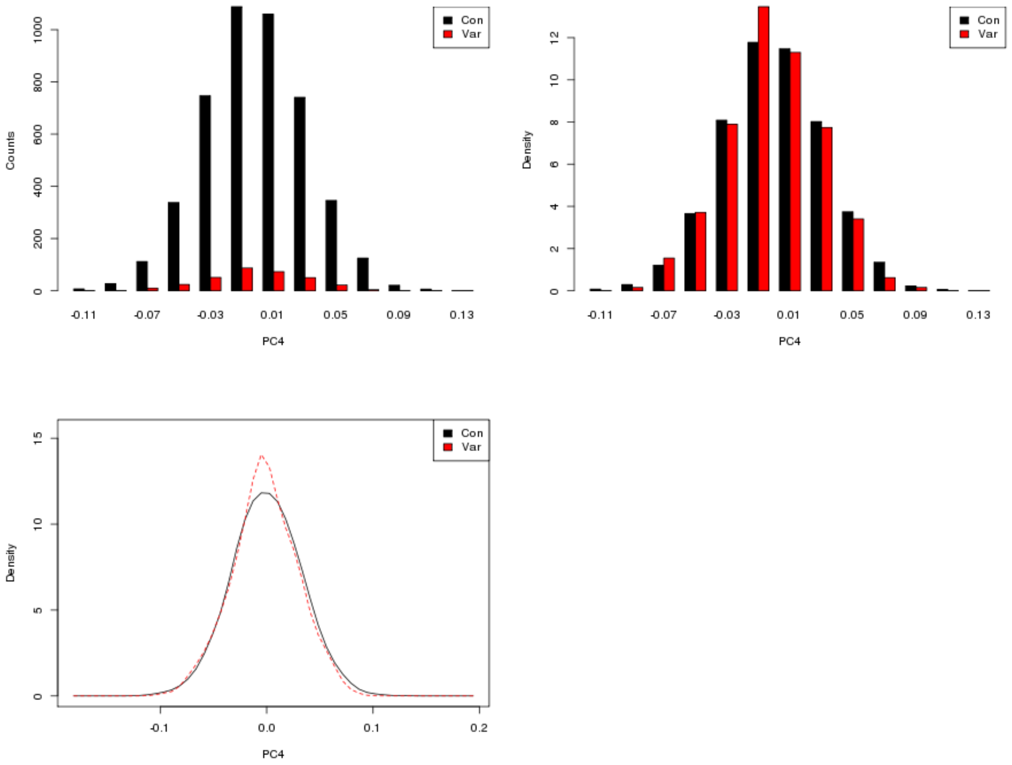
Con
Var
Counts
0
200
400
600
800
1000
-0.11
-0.07
-0.03
0.01
0.05
0.09
0.13
PC4
Density
0
2
4
6
8
10
12
5
15
-0.1
0.0
0.1
0.2

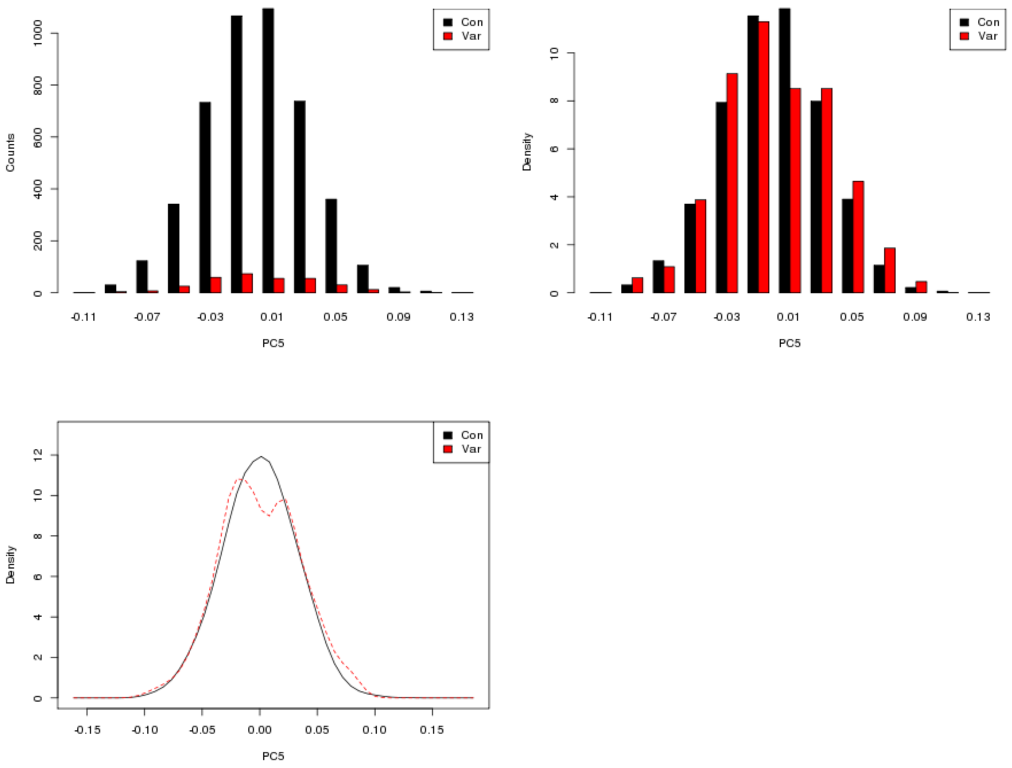
Con
Var
Counts
Density
0
200
400
600
800
1000
2
4
6
8
10
12
-0.11
-0.07
-0.03
0.01
0.05
0.09
0.13
-0.15
-0.10
-0.05
0.00
0.05
0.10
0.15
PC5

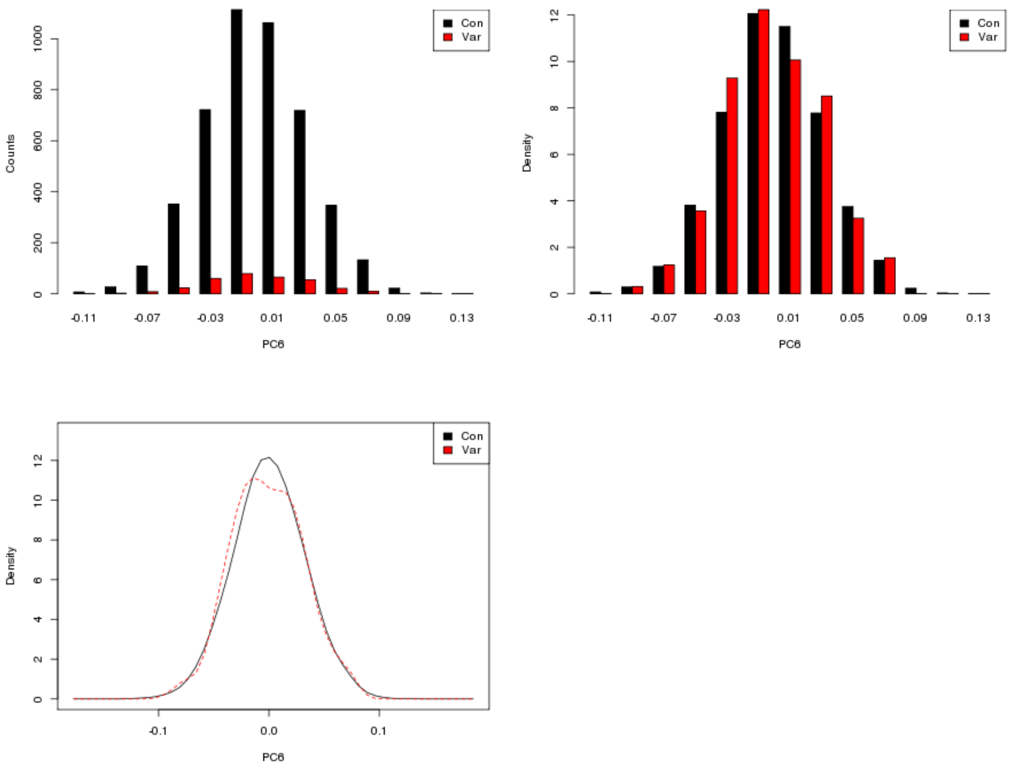
Con
Var
Counts
Density
0
200
400
600
800
1000
2
4
6
8
10
12
-0.11
-0.07
-0.03
0.01
0.05
0.09
0.13
-0.1
0.0
0.1
PC6

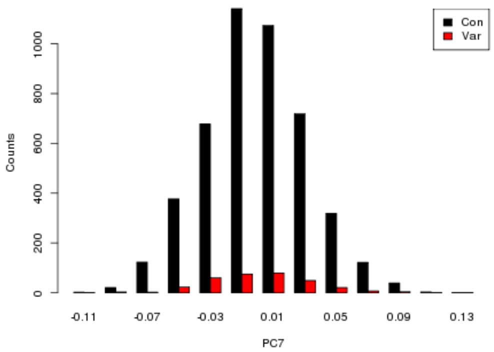
Con
Var
Counts
PC7

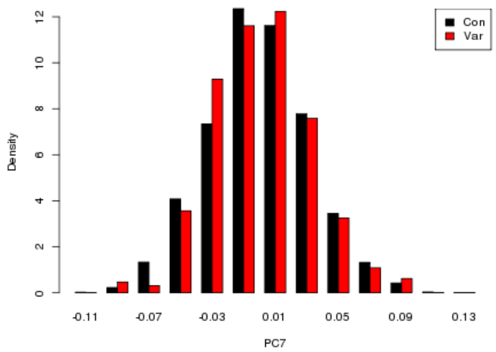
Con
Var
Density
PC7

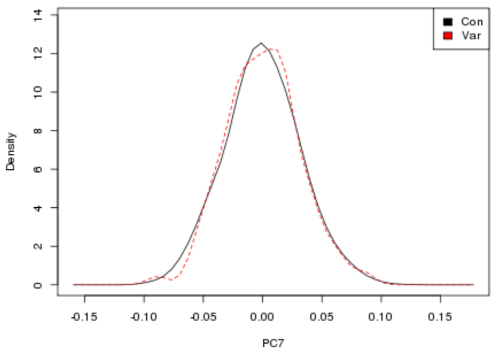
Con
Var
Density
PC7

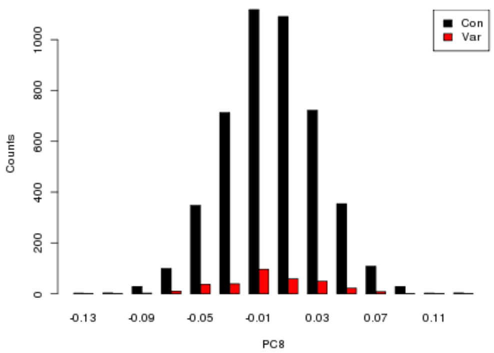
Con
Var
Counts
PC8

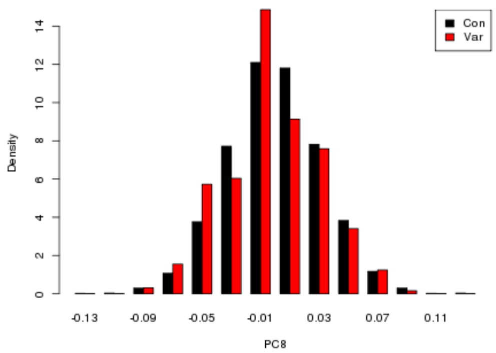
Con
Var
Density
PC8

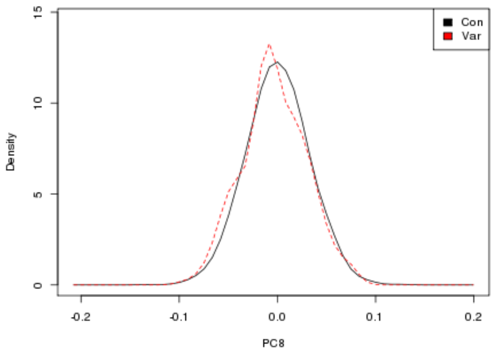
Con
Var
Density
PC8

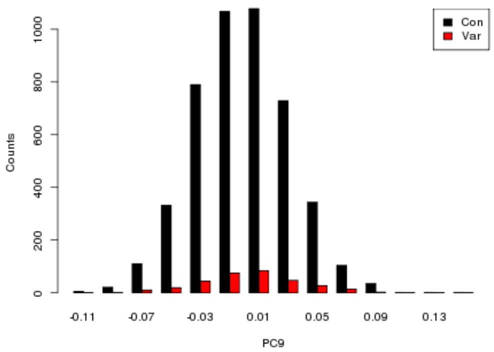
Con
Var
Counts
0
200
400
600
800
1000
-0.11
-0.07
-0.03
0.01
0.05
0.09
0.13
PC9

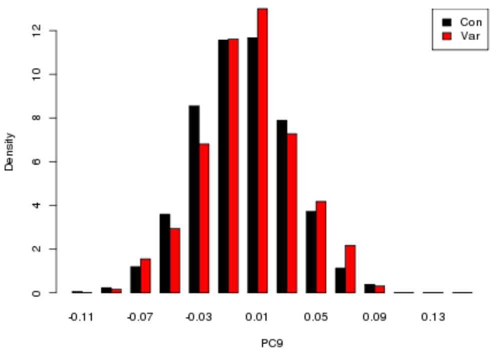
Con
Var
Density
0
2
4
6
8
10
12
-0.11
-0.07
-0.03
0.01
0.05
0.09
0.13
PC9

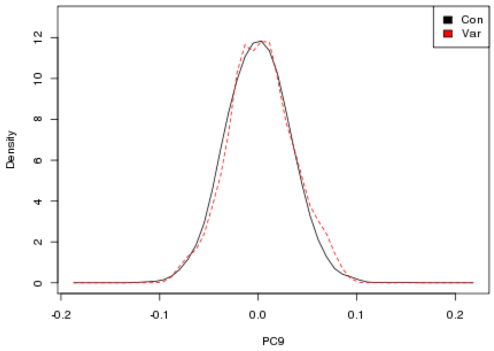
Con
Var
Density
0
2
4
6
8
10
12
-0.2
-0.1
0.0
0.1
0.2
PC9

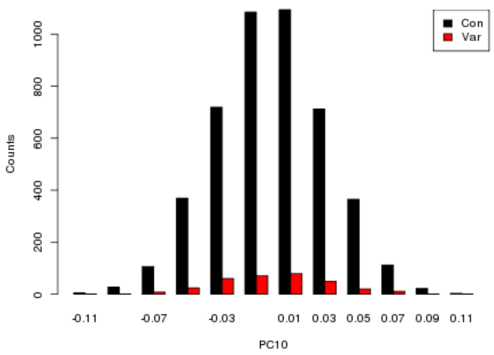
Con
Var
Counts
0
200
400
600
800
1000
-0.11
-0.07
-0.03
0.01
0.03
0.05
0.07
0.09
0.11
PC10

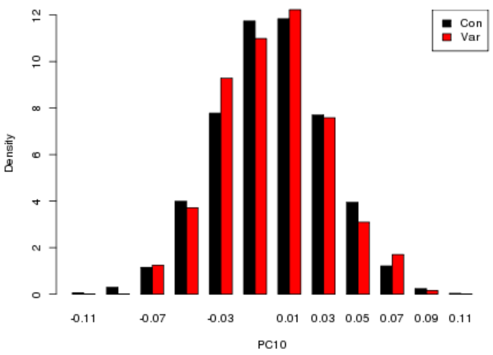
Con
Var
Density
0
2
4
6
8
10
12
-0.11
-0.07
-0.03
0.01
0.03
0.05
0.07
0.09
0.11
PC10

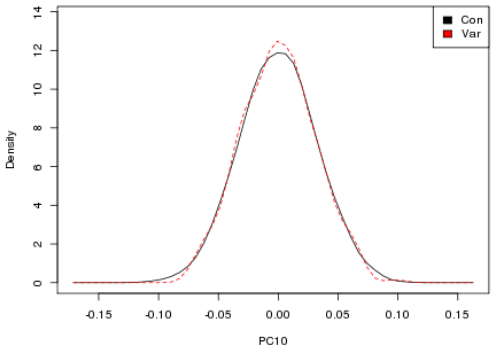
Con
Var
Density
0
2
4
6
8
10
12
14
-0.15
-0.10
-0.05
0.00
0.05
0.10
0.15
PC10

c)

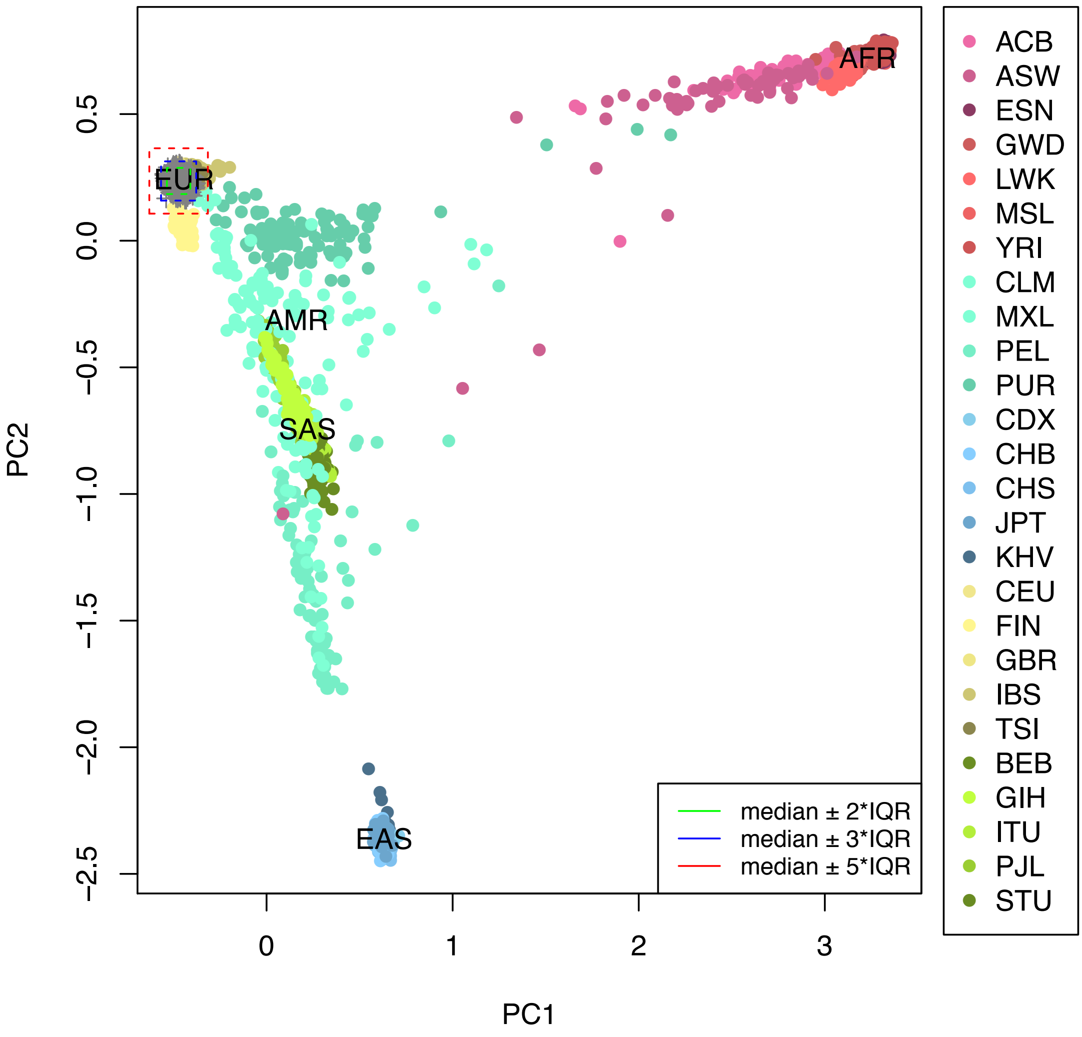

PC2
0.5
0.0
−0.5
−1.0
−1.5
−2.0
−2.5
PC1
0
1
2
3
EUR
AFR
AMR
SAS
EAS
median ± 2*IQR
median ± 3*IQR
median ± 5*IQR
ACB
ASW
ESN
GWD
LWK
MSL
YRI
CLM
MXL
PEL
PUR
CDX
CHB
CHS
JPT
KHV
CEU
FIN
GBR
IBS
TSI
BEB
GIH
ITU
PJL
STU

Supplementary Figure 2. Manhattan plot of association statistics for CVD discovery panel (panel A in Supplementary Table 1). Red horizontal line indicates the genome-wide significance threshold of 5×10^{-8}. The genome-wide significant locus did not replicate at $P<0.05$ in an independent German panel of 1,258 CVD cases and 1,925 control individuals (panel B, **Supplementary Table 1).**

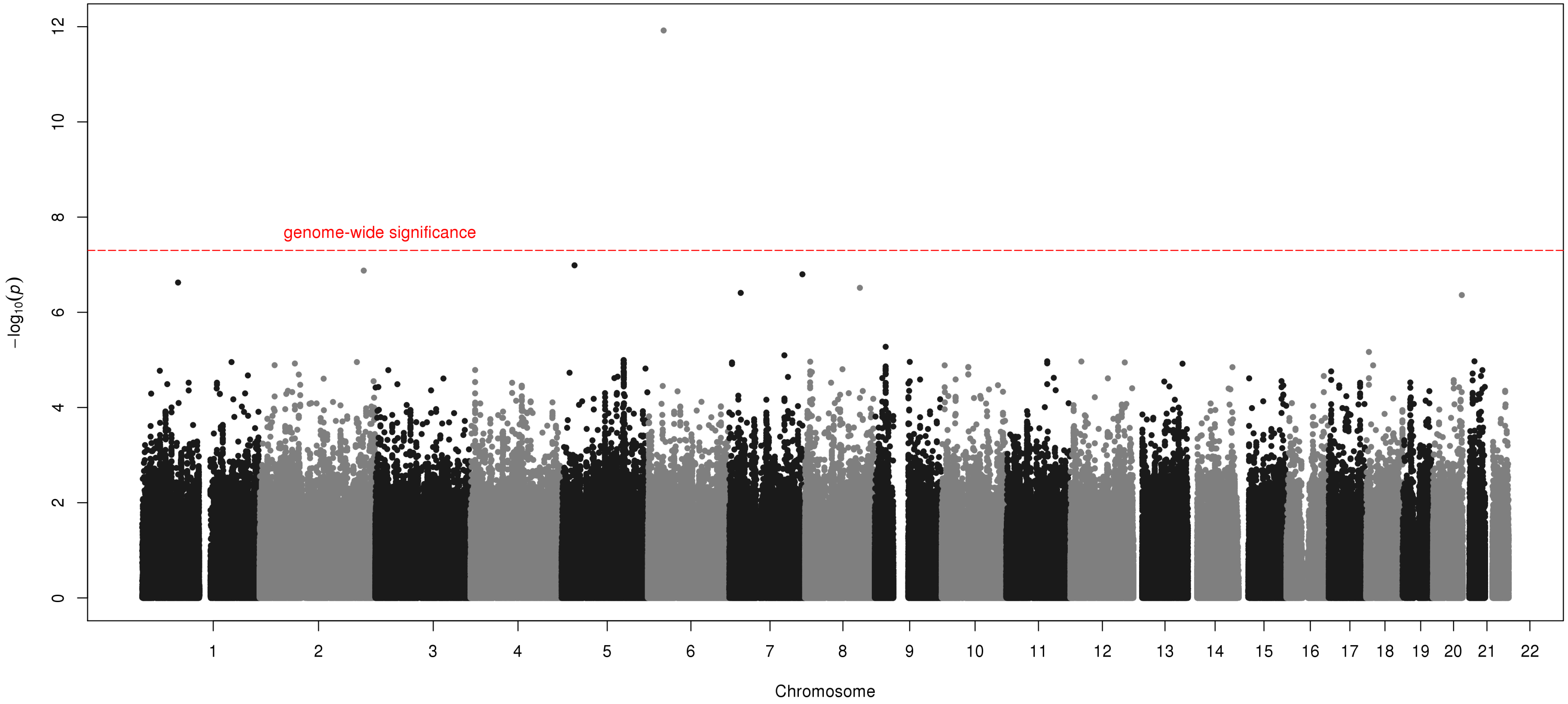

Supplementary Figure 3. eQTL box plots generated with the GTEx[8] online tool and GTEx analysis release V6. (a) SNP rs17278665 is a significant eQTL (FDR ≤ 5%) for *EFEMP1* with decreased expression associated with the minor allele in sun exposed skin of the lower leg (P=2.25×10^{-6}).[8] SNP rs17278665 is also an eQTL for *EFEMP1* in lung tissue (P=3.45×10^{-7}, no box plot data available from the respective study)[9] (**Supplementary Table 6**). **(b)** SNP rs2030136 is a significant eQTL (FDR ≤ 5%) for *HOXA5* with increased expression associated with the minor allele in thyroid tissue (P=4.70×10^{-7}).[8] SNP rs2030136 is also an eQTL for *SKAP2* in lymphoblastoid cell lines (P=8.30×10^{-9}, no box plot data available from the respective study)[10] and whole blood (P=4.99×10^{-141}, no box plot data available from the respective study)[11] (**Supplementary Table 6**).

(a)

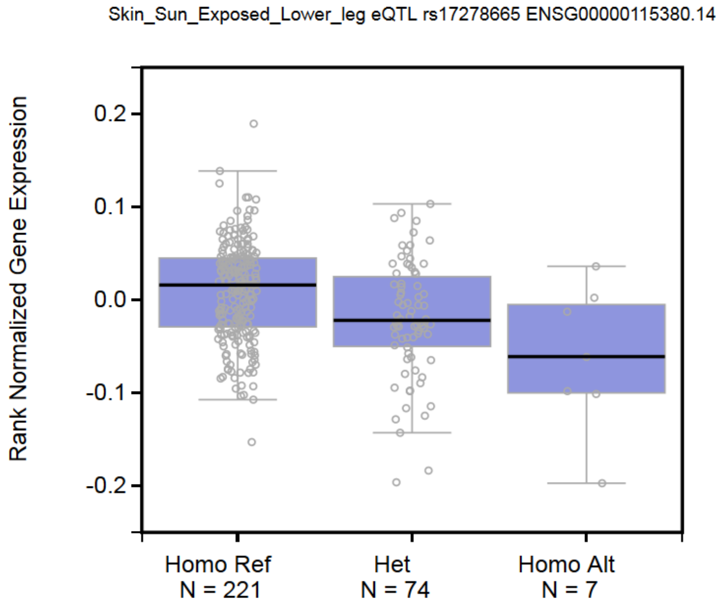

(b)

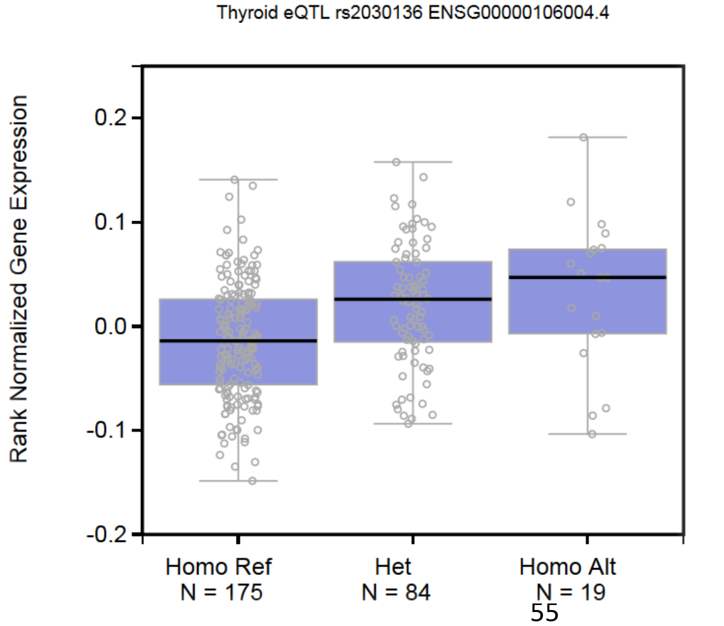

Supplementary Figure 4. Quantile-quantile (Q-Q) plot for the CVD discovery panel (panel A, Supplementary Table 1). Only markers that passed the quality criteria were used for plotting. The 2.5th and 97.5th centiles of the distribution under random sampling and the null hypothesis form the 95% concentration band. The genomic inflation factor λ is defined as the ratio of the medians of the sample χ^2 test statistics and the 1-d.f. χ^2 distribution (0.455)[12]. Since λ inferred from 1,934,349 SNPs showed moderate inflation (λ=1.156), genomic control correction was applied to results by correction of χ^2 test statistics using factor λ and multiplying SNP standard errors by the square root of the inflation factor, thus minimizing artificial differences in allele frequencies due to different genotyping platforms.

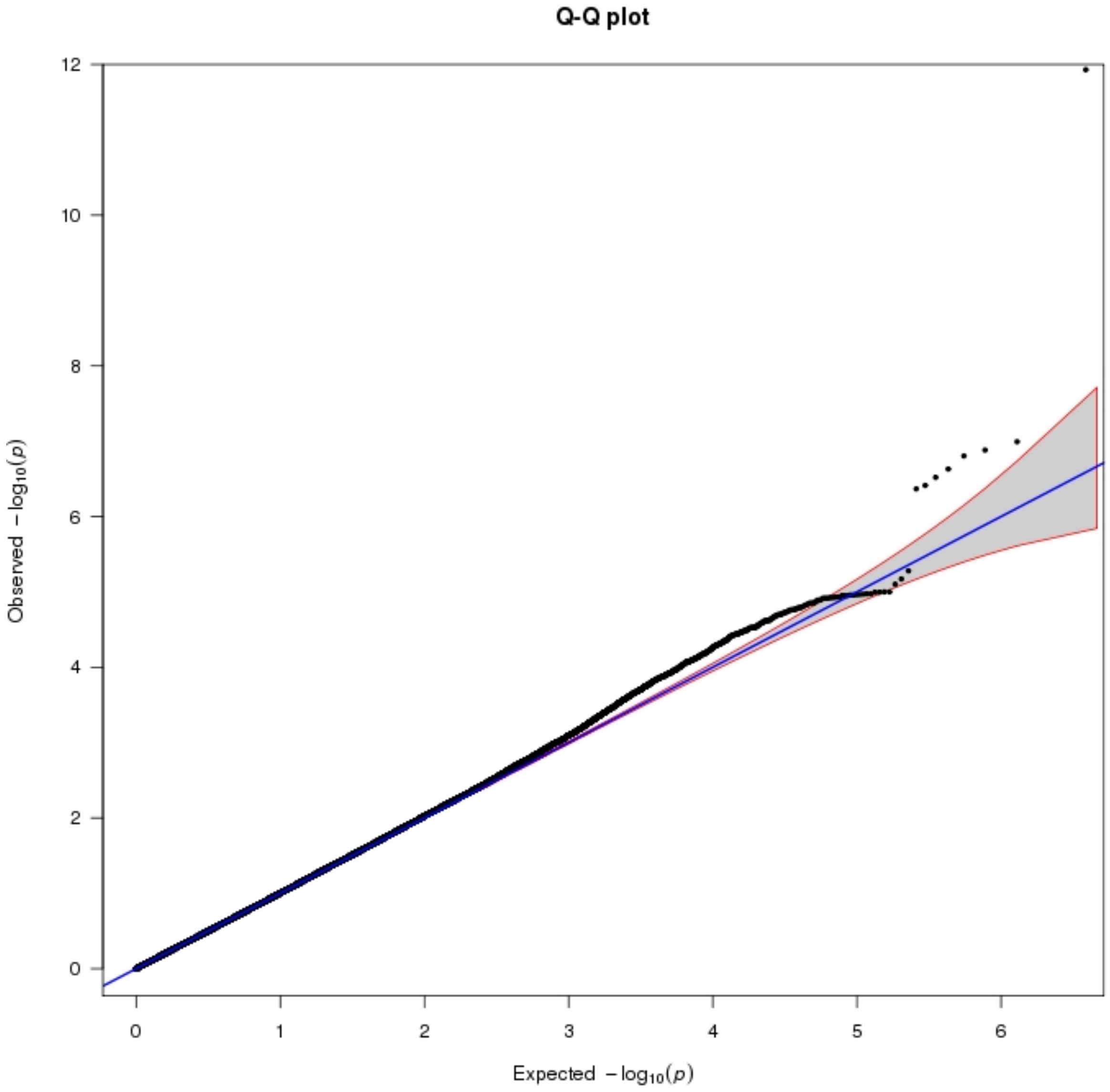

References

1. Kircher M, Witten DM, Jain P, O'Roak BJ, Cooper GM, Shendure J. A general framework for estimating the relative pathogenicity of human genetic variants. *Nat Genet*. 2014;46:310-5.
2. Shihab HA, Rogers MF, Gough J, Mort M, Cooper DN, Day IN, et al. An integrative approach to predicting the functional effects of non-coding and coding sequence variation. *Bioinformatics*. 2015;31:1536-43.
3. Schwarz JM, Cooper DN, Schuelke M, Seelow D. MutationTaster2: mutation prediction for the deep-sequencing age. *Nat Methods*. 2014;11:361-2.
4. Ward LD, Kellis M. HaploReg: a resource for exploring chromatin states, conservation, and regulatory motif alterations within sets of genetically linked variants. *Nucleic Acids Res*. 2012;40:D930-4.
5. Reese MG, Eeckman FH, Kulp D, Haussler D. Improved splice site detection in Genie. *J Comput Biol*. 1997;4:311-23.
6. Birney E, Stamatoyannopoulos JA, Dutta A, Guigo R, Gingeras TR, Margulies EH, et al. Identification and analysis of functional elements in 1% of the human genome by the ENCODE pilot project. *Nature*. 2007;447:799-816.
7. Price AL, Patterson NJ, Plenge RM, Weinblatt ME, Shadick NA, Reich D. Principal components analysis corrects for stratification in genome-wide association studies. *Nat Genet*. 2006;38:904-9.
8. GTeX-Consortium. Human genomics. The Genotype-Tissue Expression (GTEx) pilot analysis: multitissue gene regulation in humans. *Science*. 2015;348:648-60.
9. Hao K, Bosse Y, Nickle DC, Pare PD, Postma DS, Laviolette M, et al. Lung eQTLs to help reveal the molecular underpinnings of asthma. *PLoS Genet*. 2012;8:e1003029.
10. Lappalainen T, Sammeth M, Friedlander MR, t Hoen PA, Monlong J, Rivas MA, et al. Transcriptome and genome sequencing uncovers functional variation in humans. *Nature*. 2013;501:506-11.
11. Westra HJ, Peters MJ, Esko T, Yaghootkar H, Schurmann C, Kettunen J, et al. Systematic identification of trans eQTLs as putative drivers of known disease associations. *Nat Genet*. 2013;45:1238-43.
12. Devlin B, Roeder K. Genomic control for association studies. *Biometrics*. 1999;55:997-1004.
